# Enantio‐ and Regioconvergent Nickel‐Catalyzed C(sp^3^)−C(sp^3^) Cross‐Coupling of Allylic Electrophiles Steered by a Silyl Group

**DOI:** 10.1002/anie.202102233

**Published:** 2021-05-05

**Authors:** Nektarios Kranidiotis‐Hisatomi, Hong Yi, Martin Oestreich

**Affiliations:** ^1^ Institut für Chemie Technische Universität Berlin Strasse des 17. Juni 115 10623 Berlin Germany

**Keywords:** cross-coupling, nickel, radical reactions, silicon, synthetic methods

## Abstract

A two‐step sequence for the enantio‐ and diastereoselective synthesis of exclusively alkyl‐substituted acyclic allylic systems with a stereocenter in the allylic position is reported. The asymmetric induction and the site selectivity are controlled in an enantio‐ and regioconvergent nickel‐catalyzed C(sp^3^)−C(sp^3^) cross‐coupling of regioisomeric mixtures of racemic α‐/γ‐silylated allylic halides and primary alkylzinc reagents. The silyl group steers the allylic displacement towards the formation of the vinylsilane regioisomer, and the resulting C(sp^2^)−Si bond serves as a linchpin for the installation of various C(sp^3^) substituents in a subsequent step.

## Graphical abstract

A nickel‐catalyzed cross‐coupling of regioisomeric mixtures of racemic silylated allylic halides and primary alkylzinc reagents enables the enantio‐ and regioconvergent synthesis of vinylsilanes with an allylic stereocenter. The silyl group controls the regiochemical outcome and is at the same time a placeholder for another alkyl substituent that can be installed in a subsequent cross‐coupling.

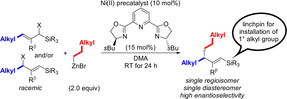

Enantioselective nickel catalysis involving radical intermediates is already a key technology for forging C(sp^3^)−C(sp^3^) bonds from racemic alkyl electrophiles in an enantioconvergent fashion.^[1]^ A broad range of zinc‐based nucleophiles and various electrophilic coupling partners can be used for that purpose, thereby enabling an impressive number of otherwise challenging bond formations.^[2]^ This field has largely been shaped by Fu, and it was also his laboratory to recently disclose the synthesis of α‐chiral silanes from racemic α‐bromo‐substituted alkylsilanes (Scheme 1, top left).^[3]^ With our interest in silicon chemistry, we had developed a similar method employing α‐silylated alkyl iodides and reported our protocol at exactly the same time (Scheme 1, top right).^[4]^ The next step for us was to investigate the related C(sp^3^)−C(sp^3^) cross‐coupling of the corresponding racemic silylated allylic systems (Scheme 1, bottom).^[5]^

**Scheme 1:**
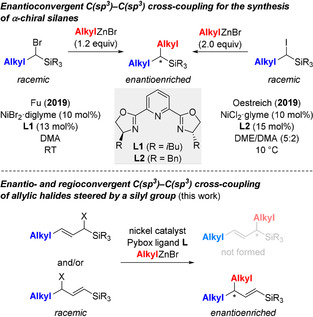
Enantioconvergent C(sp^3^)−C(sp^3^) cross‐coupling reactions of silylated alkyl and allyl electrophiles, respectively. diglyme=1‐methoxy‐2‐(2‐methoxyethoxy)ethane, glyme=DME=1,2‐dimethoxyethane, DMA=*N*,*N*‐dimethylacetamide.

Son and Fu had accomplished a Negishi‐type reaction for a diverse set of allylic chlorides in the presence of NiBr_2_⋅glyme and Pybox ligand **L3** (R=CH_2_Bn; see gray box in Scheme 1).^[2c]^ The regioselectivity was consistently high for 2° and 3° alkyl as well as electron‐withdrawing groups as one and a methyl substituent as the other in the α and γ positions of the allyl unit (not shown) but was modest with two 1° alkyl groups (Scheme 2, top). With the high regiocontrol for *tert*‐butyl/methyl and the known steering effect of silyl groups in transition‐metal‐catalyzed allylic displacements,[^6^, ^7^] we anticipated that an enantioconvergent cross‐coupling of regioisomeric mixtures of silylated allylic halides would regioselectively yield vinylsilanes with a stereogenic carbon atom in the allylic position (Scheme 1, bottom).^[8]^ The silyl group attached to a C(sp^2^) carbon atom could then be a placeholder for another 1° alkyl group,^[9]^ thereby providing a two‐step regioselective access to exclusively alkyl‐substituted acyclic allylic systems with excellent diastereo‐ and high enantiocontrol (Scheme 2, bottom).^[10]^

**Scheme 2:**
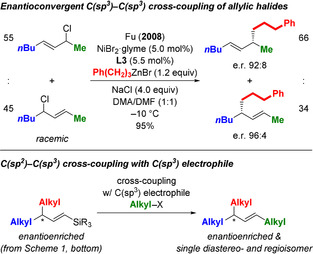
Fu's nickel‐catalyzed enantioconvergent C(sp^3^)−C(sp^3^) cross‐coupling reaction of a 1,3‐dialkyl‐substituted allylic chloride and our planned indirect approach. DMF=*N*,*N*‐dimethylformamide.

For the optimization of the reaction conditions, we chose silylated allylic bromide *rac*‐**1 a** as a mixture of regioisomers (α:γ=53:47)^[11]^ and 2.0 equiv of primary alkylzinc bromide **2 a** as model substrates (Table 1). Upon variation of the reaction parameters, we found that NiBr_2_⋅diglyme as precatalyst and Pybox ligand **L4** (R=(*S*)‐*s*Bu; see gray box in Scheme 1) as the chiral ligand in DMA can afford the C(sp^3^)−C(sp^3^) coupling product **3 aa** regioselectively in 80 % yield with a superb *E*/*Z* ratio of >98:2 and a high enantiomeric ratio of 92:8 (entry 1). Other nickel precatalysts and Pybox ligands as well as different solvents were also examined yet with no improvement (see Table S1 in the Supporting Information). Temperatures lower than room temperature had no significant effect on enantioselectivity but resulted in substantially decreased yields (entries 2 and 3). Changing the leaving group in *rac*‐**1 a** from bromide to chloride (α:γ=0:100) led to lower yield and a slightly lower enantiomeric ratio (entry 4). The corresponding acetate (α:γ=0:100) did not react (entry 5). Less alkylzinc reagent **2 a** decreased the yield without affecting the enantioselectivity (entries 6 and 7). A lower catalyst loading can be employed with only a small loss in yield and enantioselectivity (entry 8).

**Table 1:** Selected examples of the optimization.^[a]^ 
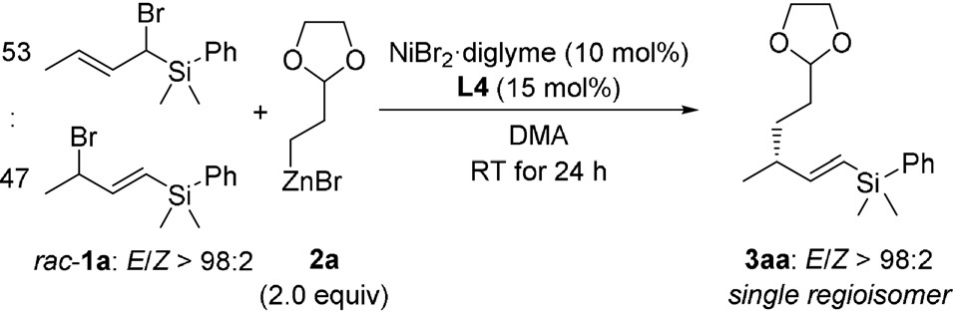

Entry	Deviation from the standard conditions	Yield [%]^[b]^	e.r.^[c]^
1	None	88 (80)^[d]^	92:8
2	0 °C instead of RT	75	92:8
3	−10 °C instead of RT	37	93:7
4	Allylic chloride instead of *rac*‐**1 a**	64	89:11
5	Allylic acetate instead of *rac*‐**1 a**	4	–
6	1.5 equiv of **2 a**	74	92:8
7	1.2 equiv of **2 a**	69	92:8
8	5.0 mol % of NiBr_2_⋅diglyme and 7.5 mol % of **L4**	77	91:9

[a] All reactions were performed on a 0.10 mmol scale. [b] Determined by GLC analysis with tetracosane as an internal standard. [c] Determined by HPLC analysis on a chiral stationary phase. [d] Isolated yield after purification by flash chromatography on silica gel.

With the optimized conditions established, we tested various primary alkylzinc reagents **2 b**–**j** with allylic bromides *rac*‐**1 a** as the coupling partner (Scheme 3). Secondary alkylzinc halides such as cyclohexylzinc bromide and iodide did not react, only yielding trace amounts of the homocoupled allylic bromide (not shown). Functional groups include another acetal (as in **2 b**), an ether as well as a silyl ether (as in **2 c** and **2 j**), a phenyl group (as in **2 d**), a nitrile (as in **2 e**), an ester (as in **2 f**) and an alkenyl group (as in **2 i**). Unfunctionalized alkyl groups (as in **2 g** and **2 h**) were also suitable for this reaction. Yields were generally good, regio‐ and diastereocontrol excellent, and enantioselectivities moderate to good. The absolute configuration of product **3 ah** had already been assigned.^[8]^ By comparison of the sign of optical rotation with the reported value, we were able to establish the absolute configuration of the obtained cross‐coupling products as *R*.

**Scheme 3:**
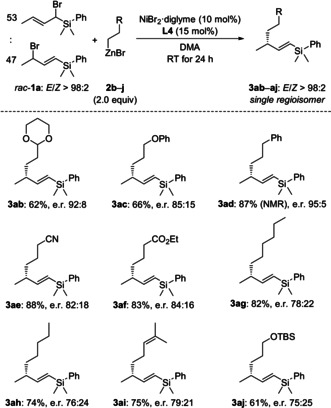
Scope I: Variation of the primary alkylzinc bromide.

Variation of the substitution pattern at the silicon atom was examined next (Scheme 4). Replacement of the Me_2_PhSi group with the more sterically hindered MePh_2_Si and *t*BuPh_2_Si groups as in *rac*‐**4 a** and *rac*‐**5 a**, respectively was not detrimental to yield and level of enantioselection. The simplest triorganosilyl group Me_3_Si as in *rac*‐**6 a** could also be installed, and the high enantiomeric ratio was retained. The same applied to the synthetically valuable BnMe_2_Si group as in *rac*‐**7 a** (see below for further processing of **11 aa**). With MePh_2_Si and BnMe_2_Si as silyl groups, we then investigated further substituents of the allyl unit. An *n*‐propyl and an *n*‐butyl instead of the methyl group could be installed as R^1^, and both the yield and the enantioselectivity were high. However, allylic bromides with a methyl group in the β‐position (not shown) were not chemically stable and could neither be purified by flash chromatography on silica gel nor isolated after distillation.

**Scheme 4:**
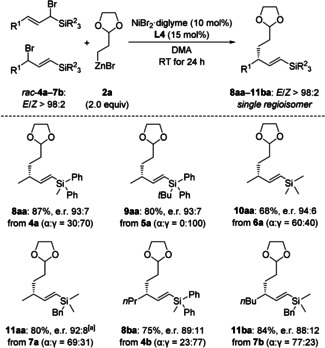
Scope II: Variation of the silylated allylic bromide. [a] A reaction on a 1.30 mmol scale afforded **11 aa** in 73 % isolated yield and with e.r. 92:8.

As a consequence thereof, we returned to chemically more robust silylated allylic chlorides (cf. Table 1, entry 4). To our delight, β‐methyl‐substituted *rac*‐**12 c** was stable during purification by conventional flash chromatography on silica gel. Moreover, the cross‐coupling of regioisomerically pure *rac*‐**12 c** and alkylzinc reagent **2 a** afforded product **8 ca** under the standard setup in good yield and with high enantioselectivity (68 % and e.r. 98:2; not shown). We did a brief reassessment of the reaction conditions and could further increase the yield to 80 % and the enantiomeric ratio to 99:1 when using NiI_2_ instead of NiBr_2_⋅diglyme (see Table S2 in the Supporting Information for details). With the modified conditions in hand, we used **2 a**–**g** in the reaction of *rac*‐**12 c** as the coupling partner (Scheme 5, top). As expected, the functional‐group tolerance was excellent (cf. Scheme 4), and isolated yields were good throughout. The enantiomeric ratios were very high, reaching 99:1 for **8 cd**. A longer alkyl chain instead of a methyl group at the allyl fragment as in *rac*‐**12 d** was also compatible with the setup, furnishing **8 da** in 90 % yield and with an enantiomeric ratio of 97:3 (Scheme 5, bottom).

**Scheme 5:**
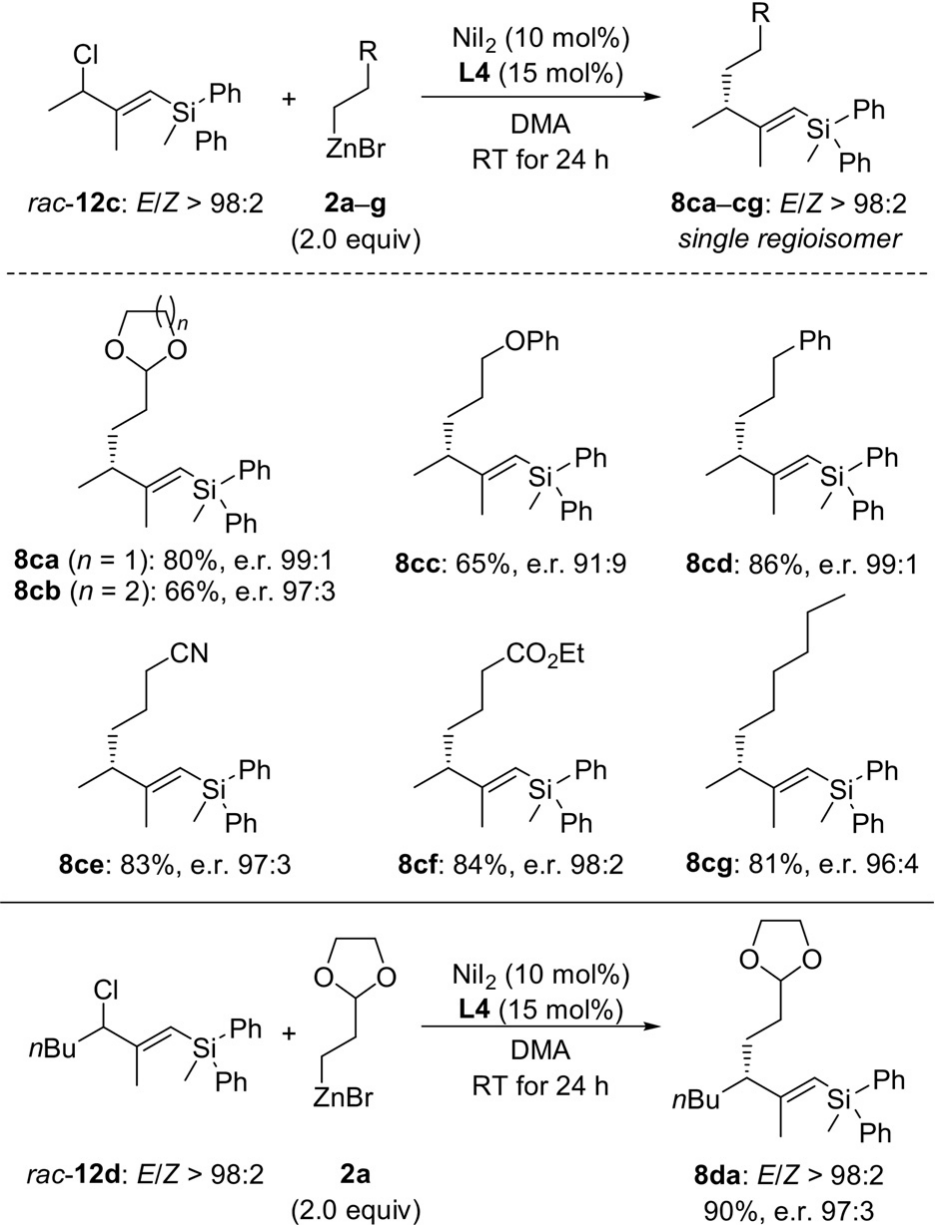
Scopes III and IV: Variation of the alkylzinc bromide (top) and the silylated allylic chloride (bottom).

The value of the present method lies in its regioconvergence. Such regioselectivity had not been achieved with allylic substrates decorated with two 1° alkyl substituents yet.[^2c^, ^10^] The controlling element is the silyl group which is at the same time a handle for the installation of another 1° alkyl group. Tsubouchi and co‐workers developed a copper‐promoted protocol for the cross‐coupling of BnMe_2_Si‐substituted vinylsilanes and alkyl electrophiles.^[9]^ To demonstrate the potential synthetic utility of our chiral vinylsilanes, we had included the BnMe_2_Si‐substituted allylic bromides *rac*‐**7 a** and *rac*‐**7 b** into our scope. The resulting products **11 aa** and **11 ba** were applied to the C(sp^2^)−C(sp^3^) cross‐coupling with two different C(sp^3^)−X coupling partners **13 a** and **13 b** (Scheme 6). These reactions proceeded in good yields to produce **14 aaa**–**bab** as single regioisomers and diastereomers without any erosion of the enantiomeric excess. The expected absolute configuration of product **14 aab** is in accordance with the literature.^[2c]^

**Scheme 6:**
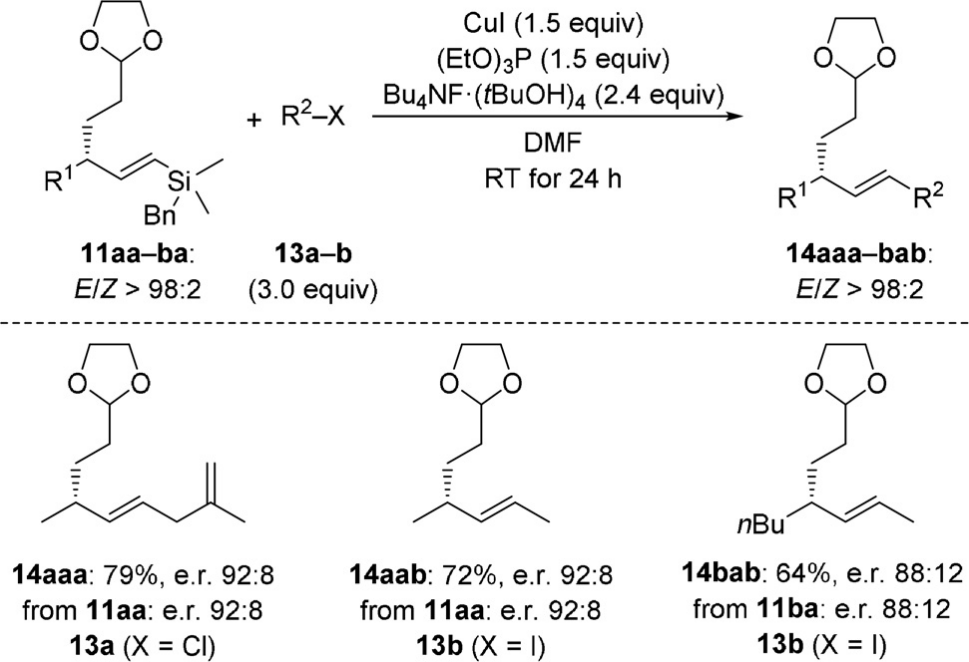
C(sp^2^)−C(sp^3^) cross‐coupling of vinylsilanes with an allylic stereocenter and C(sp^3^) electrophiles.

In summary, we developed an enantioconvergent nickel‐catalyzed C(sp^3^)−C(sp^3^) cross‐coupling of regioisomeric mixtures of racemic α‐/γ‐silylated allylic halides and primary alkylzinc reagents. The regioselectivity is governed by the silyl group[^6^, ^7^] which steers the bond formation away from the silicon‐substituted carbon atom. The resulting chiral *E*‐configured vinylsilanes can be subsequently coupled with carbon electrophiles such as allyl and alkyl halides. By this two‐step sequence, 1,3‐dialkyl‐substituted acyclic allylic systems with a stereocenter in the allylic position become available in enantio‐ and diastereoselective manner.

## Conflict of interest

The authors declare no conflict of interest.

## Supporting information

As a service to our authors and readers, this journal provides supporting information supplied by the authors. Such materials are peer reviewed and may be re‐organized for online delivery, but are not copy‐edited or typeset. Technical support issues arising from supporting information (other than missing files) should be addressed to the authors.

Supplementary
