# Supplementary material for: Enantio‐ and Regioconvergent Nickel‐Catalyzed C(sp3)−C(sp3) Cross‐Coupling of Allylic Electrophiles Steered by a Silyl Group
Source: Angew Chem Int Ed Engl. 2021 May 5;60(24):13652–5. doi: 10.1002/anie.202102233 (PMC8251714; doi:10.1002/anie.202102233)
Supplement: Supplementary file 1 — Supplementary [file ANIE-60-13652-s001.pdf]

Angewandte Chemie
Eine Zeitschrift der Gesellschaft Deutscher Chemiker

Supporting Information

Enantio- and Regioconvergent Nickel-Catalyzed C(sp^3)−C(sp^3) Cross-Coupling of Allylic Electrophiles Steered by a Silyl Group

*Nektarios Kranidiotis-Hisatomi, Hong Yi, and Martin Oestreich**

anie_202102233_sm_miscellaneous_information.pdf

Table of Contents

1. General Information S4

2. Optimization Study S6

2.1. General Procedure for the Optimization of Reaction Conditions (GP1) S6

3. Experimental Details for the Preparation of Alkylzinc Reagents S9

3.1. General Procedure for the Preparation of Alkylzinc Reagents (GP2) S9

4. Experimental Details for the Preparation of Silylated Allylic Halides (Br, Cl) and Acetate S10

4.1. General Procedure for the Preparation of Silylated Allylbromides (Me_2PhSi, $MePh_2Si$ and *t*$BuPh_2Si$ Substituents) (GP3) S10

4.2. General Procedure for the Preparation of Silylated Allylbromides (Me_3Si Substituent) (GP4) S11

4.3. General Procedure for the Preparation of Silylated Allylbromides ($BnMe_2Si$ Substituent) (GP5) S12

4.4. General Procedure for the Preparation of Silylated Allylchlorides (Me_2PhSi and $MePh_2Si$ Substituents) (GP6) S13

4.5. General Procedure for the Preparation of Silylated Allylacetates (Me_2PhSi Substituent) (GP7) S14

4.6. Characterization Data of the Silylated Allylic Halides (Br, Cl) and Acetate .. S15

5. Experimental Details for the Nickel-Catalyzed Cross-Coupling S20

5.1. General Procedure for the Nickel-Catalyzed Cross-Coupling of Silylated Allylbromides (GP8) S20

5.2. General Procedure for the Nickel-Catalyzed Cross-Coupling of Silylated Allylchlorides (GP9) S20

5.3. Characterization Data of the Chiral Vinylsilanes S22

6. Experimental Details for the Copper-Mediated Cross-Coupling S34

6.1. General Procedure for the Copper-Mediated Cross-Coupling of Alkenylbenzyldimethylsilanes (GP10) S34

6.2. Characterization Data of the Chiral Alkyl-Substituted Alkenes S35

7. Scale-up Experiment S37

8. Assignment of Absolute Configuration S38

9. HPLC Traces S39

10. GLC Traces S64

11. NMR Spectra S66

12. References S177

1. General Information

Reagents and Solvents

Standard reagents and solvents were obtained from *ABCR*, *Acros Organics*, *Alfa Aesar*, *Carbolution*, *Merck*, *Sigma-Aldrich*, *or Tokyo Chemical Industry* (*TCI*). All solvents were dried and purified following standard procedures. Technical grade solvents for extraction or chromatography (cyclohexane, ethyl acetate, diethyl ether, dichloromethane, tetrahydrofuran (THF), *n*-hexane, *n*-pentane, and *tert*-butyl methyl ether) were freshly distilled prior to use. Unless otherwise noted, all commercially available reagents were used as received, including $NiBr_2 \cdot$diglyme (*Sigma-Aldrich*), NiI_2 (*Alfa Aesar*), and dimethylacetamide (DMA; 99.5%, extra dry over molecular sieves, AcroSeal®, *Acros Organics*). Brine refers to a saturated solution of NaCl in deionized water.

Reactions

All reactions were performed in flame-dried glassware using conventional Schlenk techniques under a static pressure of nitrogen, unless otherwise stated. Liquids and solutions were transferred with syringes.

Chromatography

Analytical thin layer chromatography (TLC) was performed *Macherey-Nagel* Alugram Xtra SIL G/UV_{254} silica gel 60 pre-coated aluminum-backed plates (200 μm layer thickness). Product spots were visualized under UV light (λ_{max}= 254 nm) and/or by staining with a ceric ammonium molybdate solution.

Flash column chromatography was performed on silica gel 60 (40–63 μm, 230–400 mesh, ASTM) by *Grace* using the indicated solvents.

Analytical gas liquid chromatography (GLC) was performed on a *Varian* 430-GC gas chromatograph equipped with a *Varian* Factor Four Capillary column (30 m × 0.25 mm, 0.25 μm film thickness) using the following program: N_2 carrier gas, injection temperature 250 °C, detector temperature 250 °C, flow rate: 4 mL/min; temperature program: start temperature 40 °C, heating rate 20 °C/min, end temperature 250 °C for 10 min, unless otherwise stated.

Analytical high performance liquid chromatography (HPLC) was performed on an *Agilent Technologies* 1290 Infinity instrument equipped with a chiral stationary phase using *Daicel* Chiralcel columns, using *n*-heptane/isopropanol or acetonitrile/water mixtures as the mobile phase.

Spectroscopy

Nuclear magnetic resonance (NMR) spectra were recorded in $CDCl_3$, or C_6D_6 on a *Bruker* AV 400, or AV 500 instrument with the deuterated solvent acting as an internal deuterium lock. ^{1}H and ^{13}C NMR spectra are referenced to the residual protic solvent resonances and the deuterated solvent carbon resonances, respectively ($CHCl_3$: δ = 7.26 ppm for ^{1}H NMR and $CDCl_3$: δ = 77.16 ppm for ^{13}C NMR, C_6D_5H: δ = 7.16 ppm for ^{1}H NMR and C_6D_6: δ = 128.06 ppm for ^{13}C NMR). NMR assignments were performed using COSY, HMQC, HMBC and NOESY experiments. The ^{29}Si nuclei is referenced in compliance with the unified scale for NMR chemical shifts as recommended by the IUPAC stating the chemical shift relative to Me_4Si.[S1] Data are reported as follows: chemical shift, multiplicity (s = singlet, d = doublet, t = triplet, q = quartet, qui = quintet, m = multiplet, and combinations thereof), coupling constants (Hz), and integration.

Infrared (IR) spectra were recorded from neat samples on an *Agilent Technologies* Cary 630 FTIR spectrometer equipped with a diamond ATR unit. Selected absorption maxima are reported in wavenumbers (cm^{-1}).

Mass Spectrometry

High resolution mass spectra (HRMS) were recorded on a *Thermo Scientific* LTQ Orbitrap XL by *the Analytical Facility* at the *Institut für Chemie*, *Technische Universität Berlin* using atmospheric-pressure chemical ionization (APCI), electron impact ionization (EI) or liquid injection field desorption/ionization (LIFDI) methods.

Optical Rotation

Optical rotations were measured on a *Schmidt & Haensch* Polartronic H532 polarimeter. $[a]_{\lambda}^{T}$ values are reported in 10^{-1} (° cm^2 g^{-1}); with the concentration *c* in g/100 mL, the temperature T, and the wavelength λ indicated.

2. Optimization Study

2.1. General Procedure for the Optimization of Reaction Conditions (GP1)

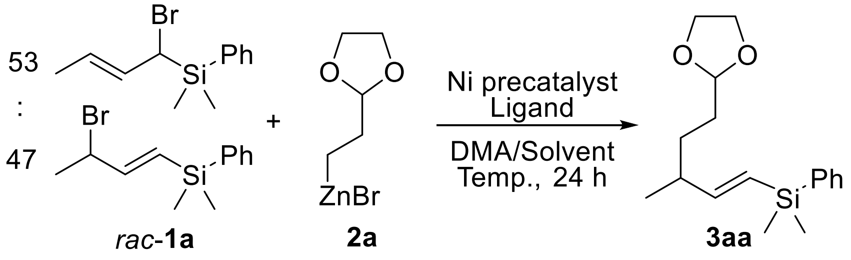

Scheme S1. Model reaction.

A flame-dried Schlenk tube equipped with a magnetic stir bar was charged with the 53:47 mixture of (2*E*)-(1-bromobut-2-en-1-yl)dimethyl(phenyl)silane and (1*E*)-(3-bromobut-1-en-1-yl) dimethyl(phenyl)silane (**1a**, 26.9 mg, 0.1 mmol, 1.0 equiv), followed by a nickel catalyst and a chiral ligand. The reaction tube was capped with a septum and wrapped with teflon tape. Then, the tube was placed under high vacuum on a Schlenk line for 20 min and backfilled with N_2 (3 times), followed by the addition of anhydrous DMA/solvent (1.0 mL). After stirring for 30 min at room temperature, the alkylzinc bromide reagent **2a** (0.2 mmol, 2.0 equiv) was added dropwise and stirred at ~800 rpm for 24 h. After completion of the reaction, tetracosane (internal standard) was added and stirred for 30 minutes. The reaction mixture was then subjected to GLC analysis after a short pipette filtration over silica gel. The enantiomeric ratio was determined by HPLC analysis on a chiral stationary phase.

Table S1. Optimization of reaction conditions for silylated allylbromide *rac*-**1a**[a]

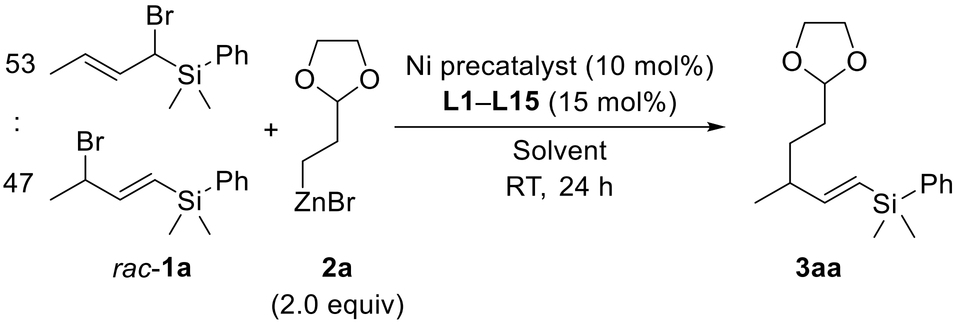

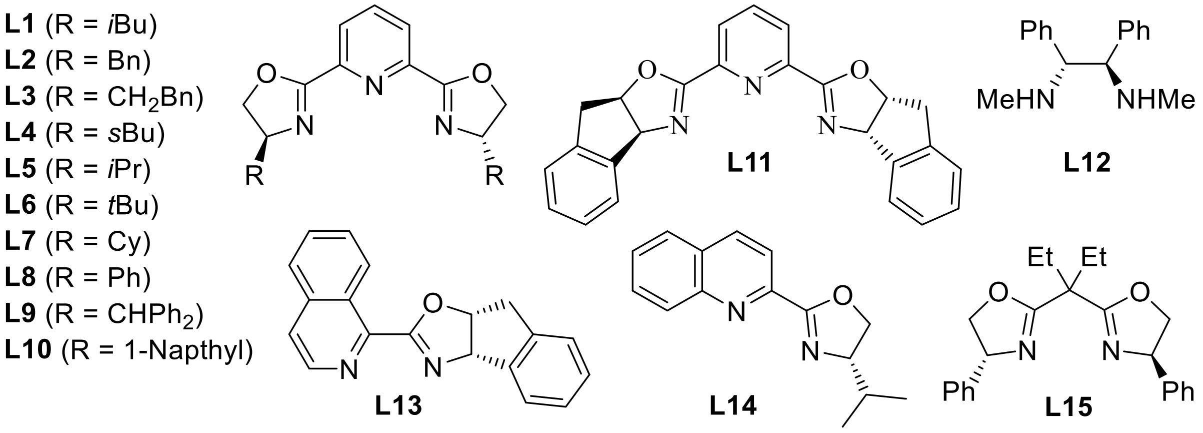

Entry	Nickel precatalyst	Solvent	Ligand	Yield [%][b]	e.r.[c]
1	$NiBr_2$·diglyme	DMA	**L1**	5	—
2	$NiBr_2$·diglyme	DMA	**L2**	65	87:13
3	$NiBr_2$·diglyme	DMA	**L3**	30	76:24
4	$NiBr_2$·diglyme	DMA	**L4**	88	92:8
5	$NiBr_2$·diglyme	DMA	**L5**	71	90:10
6	$NiBr_2$·diglyme	DMA	**L6**	19	61:39
7	$NiBr_2$·diglyme	DMA	**L7**	63	90:10
8	$NiBr_2$·diglyme	DMA	**L8**	14	85:15
9	$NiBr_2$·diglyme	DMA	**L9**	7	—
10	$NiBr_2$·diglyme	DMA	**L10**	39	83:17
11	$NiBr_2$·diglyme	DMA	**L11**	36	87:13
12	$NiBr_2$·diglyme	DMA	**L12**	3	—
13	$NiBr_2$·diglyme	DMA	**L13**	6	—
14	$NiBr_2$·diglyme	DMA	**L14**	20	50:50
15	$NiBr_2$·diglyme	DMA	**L15**	8	—
16	$NiBr_2$·glyme	DMA	**L4**	72	91:9
17	$NiCl_2$·glyme	DMA	**L4**	71	91:9
18	NiI_2	DMA	**L4**	74	91:9
19	$Ni(acac)_2$	DMA	**L4**	15	89:11
20	$Ni(cod)_2$	DMA	**L4**	58	91:9
21	$NiBr_2$·diglyme	DMA:THF (1:1)	**L4**	70	91:9
22	$NiBr_2$·diglyme	DMA:DME (1:1)	**L4**	69	91:9
23	$NiBr_2$·diglyme	DMA:1,4-Dioxane (1:1)	**L4**	70	90:10
24	$NiBr_2$·diglyme	DMA:DMF (1:1)	**L4**	68	91:9

25	$NiBr_2$·diglyme	DMA:Diglyme (1:1)	**L4**	71	90:10
26	$NiBr_2$·diglyme	DMA:NMP (1:1)	**L4**	73	91:9
27	$NiBr_2$·diglyme	DMA:DMPU (1:1)	**L4**	69	91:9

[a] All reactions were performed on a 0.10 mmol scale. [b] Determined by GLC analysis with tetracosane as an internal standard. [c] Determined by HPLC analysis on a chiral stationary phase. DMA = *N*,*N*-dimethylacetamide, diglyme = 1-methoxy-2-(2-methoxyethoxy)ethane, glyme = DME = 1,2-dimethoxyethane, acac = acetylacetonate, cod = cyclooctadiene, DMF = *N*,*N*-Dimethylformamide, NMP = 1-methylpyrrolidin-2-one, DMPU = 1,3-Dimethyl-1,3-diazinan-2-one.

Table S2. Optimization of reaction conditions for silylated allylchloride *rac*-**12c**[a]

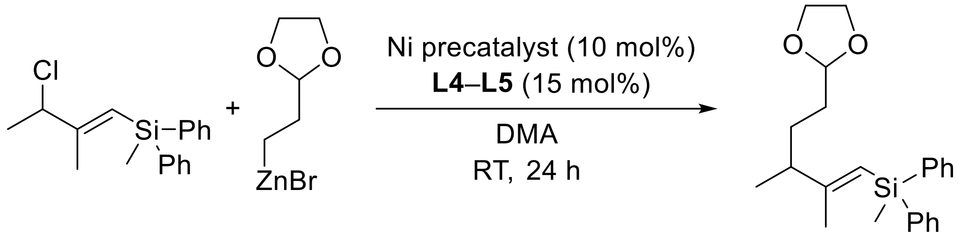

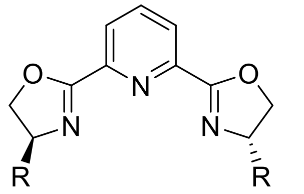

L4 (R = *s*Bu)
L5 (R = *i*Pr)

Entry	Nickel precatalyst	Ligand	Yield [%][b]	e.r.[c]
1	$NiBr_2$·diglyme	**L4**	68	98:2
2	$NiBr_2$·glyme	**L4**	75	98:2
3	$NiCl_2$·glyme	**L4**	75	98:2
4	$NiCl_2$·glyme	**L5**	58	98:2
5	NiI_2	**L4**	80	99:1
6	NiI_2	**L5**	52	97:3

[a] All reactions were performed on a 0.10 mmol scale. [b] Isolated yield after purification by flash chromatography on silica gel. [c] Determined by HPLC analysis on a chiral stationary phase.

3. Experimental Details for the Preparation of Alkylzinc Reagents

3.1. General Procedure for the Preparation of Alkylzinc Reagents (GP2)[S2]

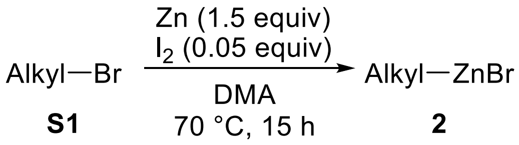

Scheme S2. Preparation of alkylzinc reagents.

A flame-dried Schlenk flask charged with a magnetic stir bar and zinc dust powder (980.9 mg, 15.0 mmol, 1.5 equiv) was sealed with a septum cap and heated at 70 °C under high vacuum on a Schlenk line for 1 h. After refilling the flask with N_2 and allowing to cool to room temperature, iodine (126.9 mg, 0.5 mmol, 0.05 equiv) in DMA (3 mL) was added under N_2, and the suspension was stirred until the solution turned colourless. In turn, the alkyl bromide **S1** (10.0 mmol, 1.0 equiv) in DMA (10.0 mL) was added under N_2 via syringe and the reaction was stirred at 70 °C for 15 h. Next, the reaction mixture was cooled to room temperature and was filtered with a syringe filter (pore size: 0.45 μM, PTFE). A sample of the solution was titrated with a known amount of iodine in THF (1.0 mL).

4. Experimental Details for the Preparation of Silylated Allylic Halides (Br, Cl) and Acetate

The yields have not been optimized.

4.1. General Procedure for the Preparation of Silylated Allylbromides (Me_2PhSi, $MePh_2Si$ and tBuPh$_2$Si Substituents) (GP3)

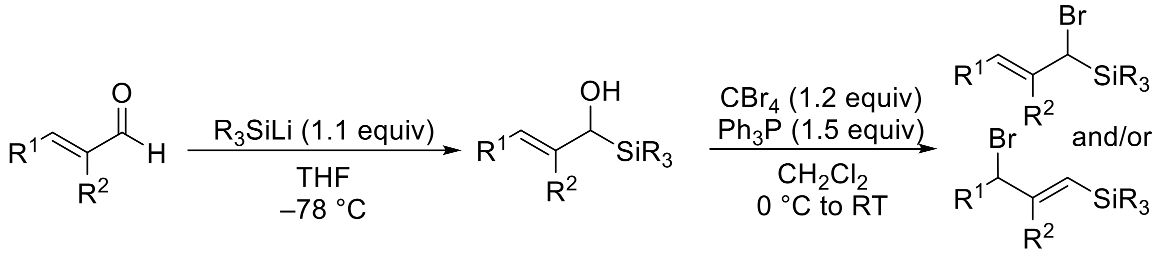

S2 *rac*-**S3**–**S5** *rac*-**1** and *rac*-**4**–**5**

Scheme S3. Preparation of *rac*-silylated allylbromides (Me_2PhSi, $MePh_2Si$ and tBuPh$_2$Si substituents).

Step One:[S3] A flame-dried round-bottom flask equipped with a magnetic stir bar was charged with R_3SiCl (7.4 mmol, 1.1 equiv), THF (18.5 mL) and was sealed with a rubber septum cap. The reaction was stirred under N_2 at room temperature for 10 min. Then, small pellets of Li metal (36.9 mmol, 5.5 equiv) were added portionwise and the suspension was furtherly and vigorously stirred for 16 h (note: after ~30 min of stirring, the reaction mixture turned dark brown). The solution of R_3SiLi (7.4 mmol, 1.1 equiv) was transferred to a flask and cooled to –78 °C. The α,β-unsaturated aldehyde **S2** (6.7 mmol, 1.0 equiv) in THF (5.6 mL) was then added dropwise via syringe.[S4] The reaction mixture was stirred at –78 °C before being quenched by addition into saturated aqueous NH_4Cl (90 mL) and extracted with diethyl ether. The combined extracts were dried ($MgSO_4$), filtrated and evaporated. Then, a quick purification of the residue by flash column chromatography on silica gel using cyclohexane/*tert*-butyl methyl ether or cyclohexane/ethyl acetate to afford the α-hydroxy allylsilane **S3**–**S5**.

Step Two:[S5] To a solution of the corresponding α-hydroxy allylsilane **S3**–**S5** (1.0 equiv) in dichloromethane (0.97 M), CBr_4 (1.2 equiv) was added slowly. After stirring for 10 min, a solution of Ph_3P (1.5 equiv) in dichloromethane (1.1 M) was added dropwise at 0 °C. Then, the reaction mixture was allowed to warm to room temperature and monitored by TLC. After reaction completion, the solvent was evaporated under vacuum and the residue was purified by column chromatography to give the desired product **1** and **4**–**5**.

4.2. General Procedure for the Preparation of Silylated Allylbromides (Me_3Si Substituent) (GP4)

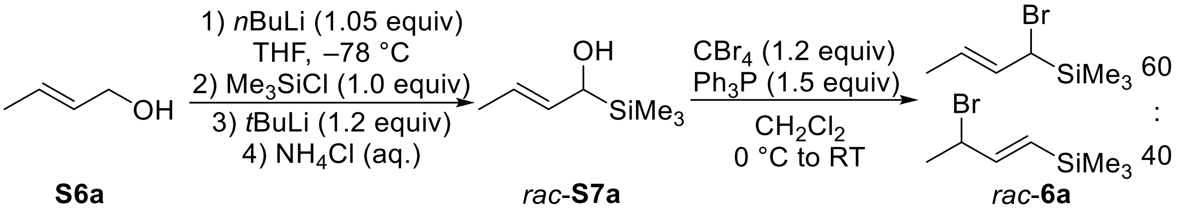

S6a *rac*-**S7a** *rac*-**6a**

Scheme S4. Preparation of *rac*-silylated allylbromides (Me_3Si substituent).

Step One:[S6] At –78 °C, to a stirred solution of (*E*)-2-propenol (**S6a**,1.0 g, 13.8 mmol, 1.0 equiv) in THF (10.4 mL) under N_2 was added *n*BuLi (5.8 mL of a 2.5 M solution in *n*-hexane, 14.43 mmol, 1.05 equiv) dropwise via a syringe. Upon complete addition, the reaction mixture was stirred for 1 h. Next, TMSCl (1.75 mL, 13.8 mmol, 1.0 equiv) was added dropwise via a syringe. Following this addition, the reaction mixture was stirred for 2.5 h and then *t*BuLi (8.8 mL of a 1.9 M solution in *n*-pentane, 16.7 mmol, 1.2 equiv) was added dropwise via a syringe. After stirring for additional 2 h, the reaction was quenched by the addition of saturated aqueous NH_4Cl and then diluted with diethyl ether. The aqueous phase was extracted with diethyl ether and the combined organic phases were washed with brine, dried ($MgSO_4$), filtrated and evaporated. The residue was quickly purified by silica gel column chromatography to afford α-hydroxy allylsilane **S7a**.

Step Two:[S5] To a solution of the corresponding α-hydroxy allylsilane **S7a** (1.0 equiv) in dichloromethane (0.97 M), CBr_4 (1.2 equiv) was added slowly. After stirring for 10 min, a solution of Ph_3P (1.5 equiv) in dichloromethane (1.1 M) was added dropwise at 0 °C. Then, the reaction mixture was allowed to warm to room temperature and monitored by TLC. After reaction completion, the solvent was evaporated under vacuum and the residue was purified by column chromatography to give the desired product **6a**.

4.3. General Procedure for the Preparation of Silylated Allylbromides (BnMe2Si Substituent) (GP5)

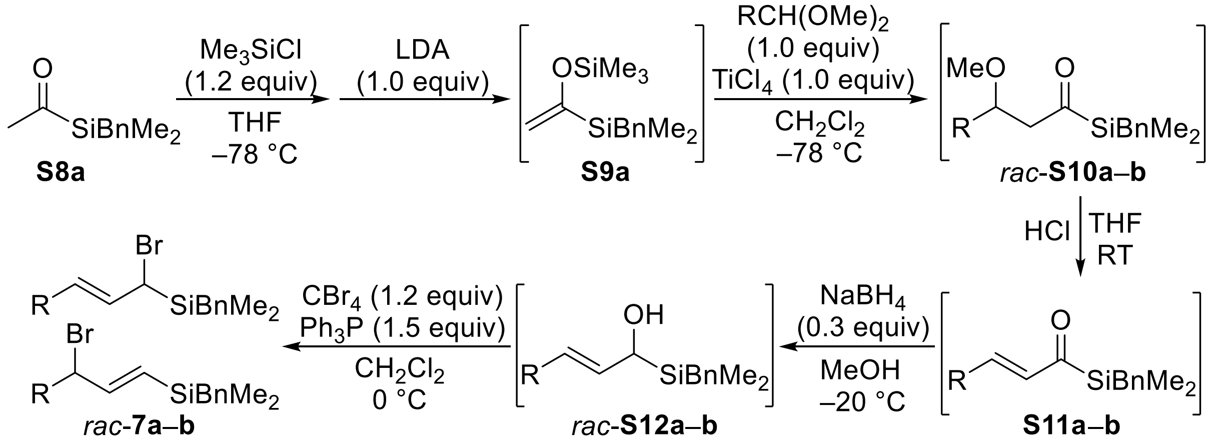

Scheme S5. Preparation of *rac*-silylated allylbromides (BnMe2Si substituent).

Step One:[S7] A flame-dried round-bottom flask equipped with a magnetic stir bar was charged with 33.0 ml of dry THF and diisopropylamine (0.78 ml, 5.5 mmol, 1.1 equiv) cooled to –78 °C and *n*BuLi (2.5 M solution in *n*-hexane, 2.1 ml, 5.25 mmol, 1.05 equiv) was added dropwisely. After 30 min, a solution of acylsilane **S8a** (961.7 mg, 5.0 mmol, 1.0 equiv) (prepared according to the literature)[S8] in 9.0 mL THF was added. After 30 min, a solution of Me_3SiCl (0.76 mL, 6.0 mmol, 1.2 equiv) was added. The reaction mixture was stirred 30 min and then allowed to warm to room temperature. Brine was then added, and aqueous layer was extracted with diethyl ether. The combined organic layers were washed with brine, dried (Na_2SO_4), filtered, and concentrated. The residue was used without further purification in the next step.

Step Two:[S9] To a stirred solution of titanium tetrachloride (0.55 mL, 5.0 mmol, 1.0 equiv) in dichloromethane (13.5 mL) at –78°C, a solution of 1,1-dimethyl acetal (5.0 mmol, 1.0 equiv) in dichloromethane (2.0 mL) was added slowly. Then, crude acylsilane silyl enol ether **S9a** in dichloromethane (3.0 mL) was added. After stirring for 2 h, methanol (2.0 mL) was added and the reaction mixture was allowed to warm to room temperature and then poured into saturated aqueous $NaHCO_3$. The aqueous layer was extracted with *n*-hexane. The combined organic layers were washed with brine, dried (Na_2SO_4), filtered, and concentrated. The residue was used without further purification in the next step.

Step Three:[S9] To a stirred solution of crude β-methoxyacylsilane **S10** in THF (45.0 mL) at room temperature, an aqueous solution of HCl (1.0 M solution in H_2O, 12.5 mmol, 12.5 mL, 2.5 equiv) was added slowly. The resulting reaction mixture was stirred at room temperature for 18 h. After completion, brine was added. The aqueous layer was extracted with diethyl ether and the combined organic layers were washed with saturated aqueous $NaHCO_3$ and brine, dried (Na_2SO_4), filtered, and concentrated. The residue was used without further purification in the next step.

Step Four: To a solution of crude acylsilane **S11** in MeOH (10.0 mL) was added $NaBH_4$ (56.7 mg, 1.5 mmol, 0.3 equiv) at –20 °C. The reaction mixture was stirred at –20 °C for 2 h, quenched with saturated aqueous NH_4Cl, and extracted with AcOEt. The combined organic layers were washed with brine, dried (Na_2SO_4), filtered, and concentrated. The residue was used without further purification in the next step.

Step Five:[S5] To a solution of the corresponding crude α-hydroxy allylsilane **S12** in dichloromethane (5.0 mL), CBr_4 (2.0 g, 6.0 mmol, 1.2 equiv) was added slowly. After stirring for 10 min, a solution of Ph_3P (2.0 g, 7.5 mmol, 1.5 equiv) in dichloromethane (4.5 mL) was added dropwise at 0 °C. After stirring for 2 h, the solvent was evaporated under vacuum and the residue was purified by column chromatography using cyclohexane to give the desired product **7**.

4.4. General Procedure for the Preparation of Silylated Allylchlorides (Me_2PhSi and $MePh_2Si$ Substituents) (GP6)

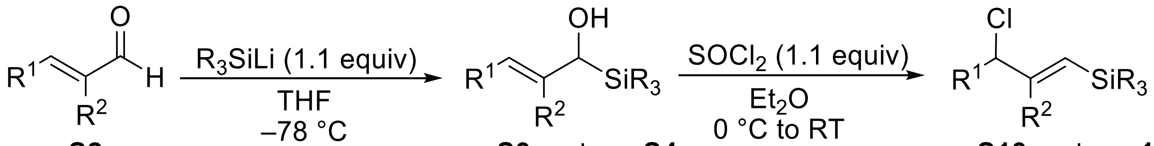

S2 *rac*-**S3** and *rac*-**S4** *rac*-**S13** and *rac*-**12**

Scheme S6. Preparation of *rac*-silylated allylchlorides (Me_2PhSi, $MePh_2Si$ substituents).

Step One:[S3] A flame-dried round-bottom flask equipped with a magnetic stir bar was charged with R_3SiCl (2.2 mmol, 1.1 equiv), THF (5.5 mL) and was sealed with a rubber septum cap. The reaction was stirred under N_2 at room temperature for 10 min. Then, small pellets of Li metal (11.0 mmol, 5.5 equiv) were added portionwise and the suspension was furtherly and vigorously stirred for 16 h (note: after ~30 min of stirring, the reaction mixture had turned dark brown). The solution of R_3SiLi (2.2 mmol, 1.1 equiv) was transferred to a flask and cooled to –78 °C. The α,β-unsaturated aldehyde **S2** (2.0 mmol, 1.0 equiv) in THF (1.7 mL) was then added dropwise via syringe.[S4] The reaction mixture was stirred at –78 °C for 1 h before being quenched by addition into saturated aqueous NH_4Cl (27 mL) and extracted with diethyl ether. The combined extracts were dried ($MgSO_4$), filtrated, and evaporated. Then, a quick purification of the residue by flash column chromatography on silica gel using cyclohexane/*tert*-butyl methyl ether (9:1) to afford the α-hydroxy allylsilane **S3** and **S4**.

Step Two:[S10] To a solution of the corresponding α-hydroxy allylsilane (1.0 equiv) in diethyl ether (0.4 M), thionyl chloride (1.1 equiv) was added dropwise at 0 °C. The reaction mixture was stirred at room temperature for 1 h. After reaction completion, the solvent and volatiles were evaporated under vacuum and the residue was purified by column chromatography to afford the γ-chloro vinylsilane **S13** and **12**.

4.5. General Procedure for the Preparation of Silylated Allylacetates (Me_2PhSi Substituent) (GP7)

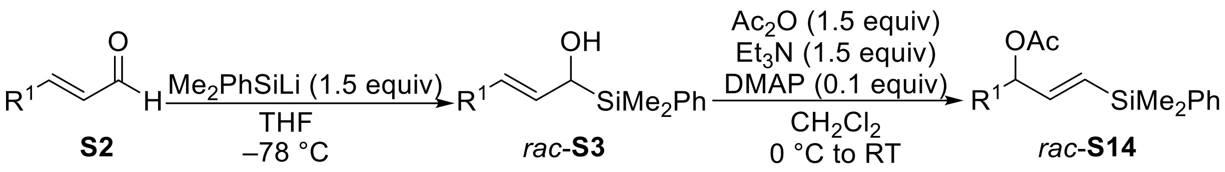

Scheme S7. Preparation of *rac*-silylated allylacetates (Me_2PhSi substituent).

Step One:[S3] A flame-dried round-bottom flask equipped with a magnetic stir bar was charged with $Me_2PhSiCl$ (0.37 mL, 2.2 mmol, 1.1 equiv), THF (5.5 mL) and was sealed with a rubber septum cap. The reaction was stirred under N_2 at room temperature for 10 min. Then, small pellets of Li metal (76.3 mg, 11.0 mmol, 5.5 equiv) were added portionwise and the suspension was further and vigorously stirred for 16 h (note: after ~30 min of stirring, the reaction mixture had turned dark brown). The solution of $Me_2PhSiLi$ (2.2 mmol, 1.1 equiv) was transferred to a flask and cooled to –78 °C. The α,β-unsaturated aldehyde **S2** (2.0 mmol, 1.0 equiv) in THF (1.7 mL) was then added dropwise via syringe.[S4] The reaction mixture was stirred at –78 °C for 1 h before being quenched by addition into saturated aqueous NH_4Cl (27 mL) and extracted with diethyl ether. The combined extracts were dried ($MgSO_4$), filtrated and evaporated. Then, a quick purification of the residue by flash column chromatography on silica gel using cyclohexane/*tert*-butyl methyl ether (9:1) to afford the α-hydroxy allylsilane **S3**.

Step Two:[S11] A solution of the corresponding α-hydroxy allylsilane **S3** (1.0 equiv) in dichloromethane (0.1 M) was cooled to 0 °C. Next, Et_3N (1.5 equiv) was added followed by Ac_2O (1.5 equiv) and 4-dimethylaminopyridine (0.1 equiv). The reaction was stirred at room temperature for 2 h. The reaction mixture was washed by aqueous HCl (2 N), $NaHCO_3$ and brine. The organic phase was dried ($MgSO_4$), filtrated, and evaporated. Then, a purification of the residue by flash column chromatography on silica gel to afford the γ-acetoxy vinylsilane **S14**.

4.6. Characterization Data of the Silylated Allylic Halides (Br, Cl) and Acetate

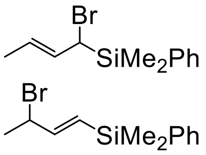

α:γ = 53:47
1a
$C_{12}H_{17}BrSi$
M = 268.03 g/mol

***rac*-(2*E*)-(1-Bromobut-2-en-1-yl)dimethyl(phenyl)silane** and ***rac*-(1*E*)-(3-bromobut-2-en-1-yl)dimethyl(phenyl)silane** (**1a**): Prepared from (*E*)-but-2-enal (**S2a**, 0.55 mL, 6.7 mmol, 1.0 equiv) according to **GP3**. Purification by flash column chromatography on silica gel using cyclohexane afforded **1a** as a yellow oil (1.35 g, 75% yield, α:γ = 53:47).

R_f = 0.74 (cyclohexane/ethyl acetate = 20/1). Major α-bromo-diastereomer **^{1}H NMR** (500 MHz, $CDCl_3$): δ 0.42 (s, 3H), 0.45 (s, 3H), 1.67 (d, *J* = 4.8 Hz, 3H), 3.99 (d, *J* = 9.2 Hz, 1H), 5.57–5.59 (m, 2H), 7.35–7.39 (m, 3H), 7.54–7.56 (m, 2H) ppm. **^{13}C NMR** (126 MHz, $CDCl_3$): δ –4.8, –4.4, 17.5, 42.9, 127.6, 128.6, 129.1, 129.5, 134.2, 138.1 ppm. **^{29}Si DEPT NMR** ppm (99 MHz, $CDCl_3$): δ –2.53. Minor γ-bromo-diastereomer **^{1}H NMR** (500 MHz, $CDCl_3$): δ 0.36 (s, 6H), 1.78 (d, *J* = 6.7 Hz, 3H), 4.66 (qui, *J* = 7.7, 6.7 Hz, 1H), 5.95 (dd, J = 18.4, 0.8 Hz, 1H), 6.26 (dd, *J* = 18.4, 7.7 Hz, 1H), 7.35–7.39 (m, 3H), 7.50–7.52 (m, 2H) ppm. **^{13}C NMR** (126 MHz, $CDCl_3$): δ –2.9, –2.8, 25.0, 50.1, 127.8, 128.6, 129.1, 133.8, 148.5 ppm. **^{29}Si DEPT NMR** ppm (99 MHz, $CDCl_3$): δ –11.02. **IR (ATR)**: $\tilde{\nu}$/cm^{-1} = 3016, 2957, 1426, 1248, 1113, 961, 823, 777, 730, 696. **HRMS (EI)** for $C_6H_{12}BrSi^+$ $[M\text{-}C_6H_5]^+$: calculated 190.9892, found 190.9017.

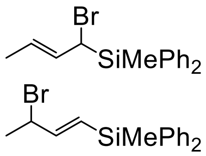

α:γ = 30:70
4a
$C_{17}H_{19}BrSi$
M = 330.04 g/mol

***rac*-(2*E*)-(1-Bromobut-2-en-1-yl)(methyl)diphenylsilane** and ***rac*-(1*E*)-(3-bromobut-2-en-1-yl)(methyl)diphenylsilane** (**4a**): Prepared from (*E*)-but-2-enal (**S2a**, 0.55 mL, 6.7 mmol, 1.0 equiv) according to **GP3**. Purification by flash column chromatography on silica gel using cyclohexane afforded **4a** as a yellow oil (1.95 g, 88% yield, α:γ = 30:70).

R_f = 0.71 (cyclohexane/ethyl acetate = 20/1). Major γ-bromo-diastereomer **^{1}H NMR** (500 MHz, $CDCl_3$): δ 0.67 (s, 3H), 1.82 (d, *J* = 6.8 Hz, 3H), 4.73 (qui, *J* = 7.5, 7.0 Hz, 1H), 6.17 (dd, J = 18.3, 0.5 Hz, 1H), 6.32 (dd, *J* = 18.3, 7.5 Hz, 1H), 7.37–7.43 (m, 6H), 7.53–7.55 (m, 4H) ppm. **^{13}C NMR** (126 MHz, $CDCl_3$): δ –3.9, 25.0, 50.7, 126.6, 127.9, 129.5, 134.8, 135.8, 150.5 ppm. **^{29}Si DEPT NMR** ppm (99 MHz, $CDCl_3$): δ –14.93. Minor α-bromo-diastereomer **^{1}H NMR** (500 MHz, $CDCl_3$): δ 0.75 (s, 3H), 1.69 (d, *J* = 5.0 Hz, 3H), 4.36 (d, *J* = 8.2 Hz, 1H), 5.68–5.69 (m, 2H), 7.37–7.43 (m, 6H), 7.57–7.59 (m, 2H), 7.64–7.66 (m, 2H) ppm. **^{13}C NMR** (126 MHz, $CDCl_3$): δ –5.2, 17.7, 41.1, 127.8, 128.9, 129.7, 129.8, 135.2, 136.4 ppm. **^{29}Si DEPT NMR** ppm (99 MHz, $CDCl_3$): δ –9.42. **IR (ATR)**: $\tilde{\nu}$/cm^{-1} = 3067, 3011, 2960, 1427, 1109, 784, 720, 694. **HRMS (EI)** for $C_{16}H_{16}BrSi^+$ $[M\text{-}CH_3]^+$: calculated 315.0205, found 315.0199.

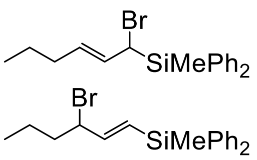

α:γ = 23:77
4b
$C_{19}H_{23}BrSi$
M = 358.08 g/mol

***rac*-(2*E*)-(1-Bromohex-1-en-1-yl)(methyl)diphenylsilane** and ***rac*-(1*E*)-(3-bromohex-1-en-1-yl)(methyl)diphenylsilane** (**4b**): Prepared from (*E*)-hex-2-enal (**S2b**, 0.78 mL, 6.7 mmol, 1.0 equiv) according to **GP3**. Purification by flash column chromatography on silica gel using cyclohexane afforded **4b** as a pale yellow oil (0.97 g, 60% yield, α:γ = 23:77).

R_f = 0.78 (cyclohexane/ethyl acetate = 20/1). Major γ-bromo-diastereomer **^{1}H NMR** (500 MHz, $CDCl_3$): δ 0.64 (s, 3H), 0.96 (t, *J* = 7.3 Hz, 3H), 1.37-1.51 (m, 2H), 1.81-2.00 (m, 2H), 4.54 (q, *J* = 16.3, 6.4 Hz, 1H), 6.13 (d, J = 18.5 Hz, 1H), 6.21 (dd, *J* = 18.5, 7.9 Hz, 1H), 7.33–7.40 (m, 4H), 7.50–7.52 (m, 6H) ppm. **^{13}C NMR** (126 MHz, $CDCl_3$): δ –3.9, 13.4, 21.0, 40.4, 57.2, 127.7, 127.9, 129.4, 134.8, 135.9, 149.6 ppm. **^{29}Si DEPT NMR** (99 MHz, $CDCl_3$): δ –15.01 ppm. Minor α-bromo-diastereomer **^{1}H NMR** (500 MHz, $CDCl_3$): δ 0.72 (s, 3H), 0.80 (t, *J* = 7.3 Hz, 3H), 1.28–1.33 (m, 2H), 1.89–2.00 (m, 2H), 4.33–4.35 (m, 1H), 5.61–5.64 (m, 2H) ppm. Signals of phenyl groups are overlapping with signals of major diastereomer. **^{13}C NMR** (126 MHz, $CDCl_3$): δ –5.3, 13.5, 22.3, 34.3, 41.2, 127.8, 129.7, 134.7, 135.2 ppm. **^{29}Si DEPT NMR** (99 MHz, $CDCl_3$): δ –9.44 ppm. **IR (ATR)**: $\tilde{v}$/cm^{-1} = 3067, 2957, 1426, 1250, 1109, 985, 785, 719, 695. **HRMS (LIFDI)** for $C_{15}H_{20}BrSi^+$ [M-CH_3]$^+$: calculated 343.0518, found 343.1029.

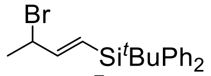

5a
$C_{20}H_{25}BrSi$
M = 372.09 g/mol

***rac*-(1*E*)-(3-Bromobut-1-en-1-yl)(*tert*-butyl)diphenylsilane** (**5a**): Prepared from (*E*)-but-2-enal (**S2a**, 0.55 mL, 6.7 mmol, 1.0 equiv) according to **GP3**. Purification by flash column chromatography on silica gel using cyclohexane afforded **5a** as a yellow oil (2.12 g, 85% yield).

R_f = 0.80 (cyclohexane/ethyl acetate = 20/1). **^{1}H NMR** (500 MHz, $CDCl_3$): δ 1.08 (s, 9H), 1.79 (d, *J* = 7.1 Hz, 3H), 4.71–4.76 (m, 1H), 6.23–6.24 (m, 2H), 7.34–7.42 (m, 6H), 7.57–7.61 (m, 4H) ppm. **^{29}Si DEPT NMR** ppm (99 MHz, $CDCl_3$): δ –9.77. **IR (ATR)**: $\tilde{v}$/cm^{-1} = 3047, 2927, 2855, 1427, 1103, 1000, 819, 773, 737, 697. **HRMS (EI)** for $C_{16}H_{16}BrSi^+$ [M-C_4H_9]$^+$: calculated 315.0199, found 315.0191.

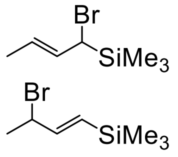

α:γ = 60:40
6a
$C_7H_{15}BrSi$
M = 206.01 g/mol

***rac*-(2*E*)-(1-Bromobut-2-en-1-yl)trimethylsilane and *rac*-(1*E*)-(3-bromobut-2-en-1-yl)trimethylsilane** (**6a**): Prepared from (*E*)-but-2-enal (**S2a**, 0.55 mL, 6.7 mmol, 1.0 equiv) according to **GP4**. Purification by flash column chromatography on silica gel using cyclohexane afforded **6a** as a yellow oil (0.99 g, 72% yield, α:γ = 60:40).

R_f = 0.80 (cyclohexane/ethyl acetate = 20/1). Major α-bromo-diastereomer **^{1}H NMR** (500 MHz, $CDCl_3$): δ 0.11 (s, 6H), 1.71 (d, *J* = 5.6 Hz, 3H), 3.82 (d, *J* = 10.4 Hz, 1H), 5.56–5.66 (m, 2H) ppm. **^{13}C NMR** (126 MHz, $CDCl_3$): δ –3.1, 17.7, 44.5, 128.2, 129.6 ppm. **^{29}Si DEPT NMR** (99 MHz, $CDCl_3$): δ 4.32 ppm. Major γ-bromo-diastereomer **^{1}H NMR** (500 MHz, $CDCl_3$): δ 0.07 (s, 6H), 1.76 (d, *J* = 6.8 Hz, 3H), 4.62 (qui, *J* = 7.3, 6.8 Hz, 1H), 5.81 (d, *J* = 18.5 Hz, 1H), 6.16 (dd, *J* =

18.5, 7.3 Hz, 1H) ppm. **^{13}C NMR** (126 MHz, $CDCl_3$): δ –1.5, 25.2, 51.4, 130.8, 146.9 ppm. **^{29}Si DEPT NMR** (99 MHz, $CDCl_3$): δ –6.65 ppm. **IR (ATR)**: ṽ/cm^{-1} = 2955, 1447, 1248, 1171, 961, 835, 744, 669. **HRMS (EI)** for $C_7H_{16}BrSi^+$ $[M+H]^+$: calculated 207.0205, found 207.0263.

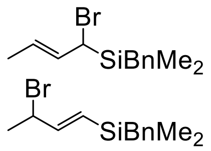

α:γ = 69:31
7a
$C_{13}H_{19}BrSi$
M = 282.04 g/mol

***rac*-(2*E*)-Benzyl(1-bromobut-2-en-1-yl)dimethylsilane** and ***rac*-(1*E*)-benzyl(1-bromobut-2-en-1-yl)dimethylsilane** (**7a**): Prepared from 1-(benzyldimethylsilyl)ethan-1-one (**S6a**, 961.6 mg, 5.0 mmol, 1.0 equiv) according to **GP5**. Purification by flash column chromatography on silica gel using cyclohexane afforded **7a** as a yellow oil (708.2 mg, 50% yield, α:γ = 69:31).

R_f = 0.66 (cyclohexane/ethyl acetate = 20/1). Major α-bromo-diastereomer **^{1}H NMR** (500 MHz, $CDCl_3$): δ 0.06 (s, 3H), 0.12 (s, 3H), 1.73 (dd, *J* = 6.8, 0.9 Hz, 3H), 2.20 (d, *J* = 13.6 Hz, 1H), 2.28 (d, *J* = 13.6 Hz, 1H), 3.84 (d, *J* = 9.9 Hz, 1H), 5.54–5.67 (m, 2H), 6.98–7.25 (m, 5H) ppm. **^{13}C NMR** (126 MHz, $CDCl_3$): δ –5.2, –5.0, 17.8, 23.6, 42.8, 124.3, 128.3, 128.3, 128.8, 129.1, 139.0 ppm. **^{29}Si DEPT NMR** (99 MHz, $CDCl_3$): δ 3.65 ppm. Minor γ-bromo-diastereomer **^{1}H NMR** (500 MHz, $CDCl_3$): δ 0.07 (s, 6H), 1.75 (d, *J* = 6.7 Hz, 3H), 2.14 (s, 2H), 4.58–4.65 (m, 1H), 5.77 (dd, J = 18.6, 0.8 Hz, 1H), 6.13 (dd, *J* = 18.6, 7.6 Hz, 1H), 6.98–7.25 (m, 5H) ppm. **^{13}C NMR** (126 MHz, $CDCl_3$): δ –3.6, –3.6, 25.1, 25.8, 51.2, 124.1, 128.1, 128.1, 128.7, 139.5, 148.1 ppm. **^{29}Si DEPT NMR** (99 MHz, $CDCl_3$): δ –6.40 ppm. **IR (ATR)**: ṽ/cm^{-1} = 3059, 2956, 1598, 1491, 1450, 1249, 1205, 829, 758, 696. **HRMS (APCI)** for $C_{13}H_{20}BrSi^+$ $[M+H]^+$: calculated 283.0518, found 283.0517.

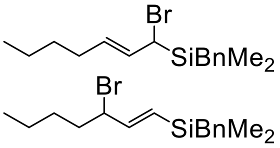

α:γ = 77:23
7b
$C_{16}H_{25}BrSi$
M = 324.09 g/mol

***rac*-(2*E*)-Benzyl(1-bromohept-2-en-1-yl)dimethylsilane** and ***rac*-(1*E*)-benzyl(3-bromohept-1-en-1-yl)dimethylsilane** (**7b**): Prepared from 1-(benzyldimethylsilyl)ethan-1-one (**S6a**, 961.6 mg, 5.0 mmol, 1.0 equiv) according to **GP5**. Purification by flash column chromatography on silica gel using cyclohexane afforded **7b** as a yellow oil (487.5 mg, 30% yield, α:γ = 77:23).

R_f = 0.69 (cyclohexane/ethyl acetate = 20/1). Major α-bromo-diastereomer **^{1}H NMR** (500 MHz, $CDCl_3$): δ 0.06 (s, 3H), 0.12 (s, 3H), 0.89 (t, *J* = 7.0 Hz, 3H), 1.29–1.37 (m, 4H), 2.02–2.06 (m, 2H), 2.20 (d, *J* = 13.3 Hz, 1H), 2.28 (d, *J* = 13.3 Hz, 1H), 3.84 (d, *J* = 10.5 Hz, 1H), 5.52–5.65 (m, 2H), 7.04–7.05 (m, 2H), 7.19–7.26 (m, 3H) ppm. **^{13}C NMR** (126 MHz, $CDCl_3$): δ –5.1, –5.0, 13.9, 22.2, 23.6, 31.3, 31.9, 42.8, 124.4, 128.0, 128.3, 128.3, 134.2, 139.0 ppm. **^{29}Si DEPT NMR** (99 MHz, $CDCl_3$): δ 3.64 ppm. Minor γ-bromo-diastereomer **^{1}H NMR** (500 MHz, $CDCl_3$): δ 0.07 (s, 6H), 0.91 (t, *J* = 7.1, 3H), 1.29–1.37 (m, 6H), 2.14 (s, 2H), 4.40–4.44 (m, 1H), 5.76 (dd, J = 18.6, 0.93 Hz, 1H), 6.05 (dd, *J* = 18.5, 8.6 Hz, 1H), 6.98–7.00 (m, 2H), 7.07–7.11 (m, 3H) ppm. **^{13}C NMR** (126 MHz, $CDCl_3$): δ –3.6, –3.5, 23.9, 22.1, 25.8, 29.8, 57.9, 124.1, 128.2, 128.3, 129.9, 147.1 ppm. **^{29}Si DEPT NMR** (99 MHz, $CDCl_3$): δ –6.50 ppm. Missing signals are overlapping with signals of major diastereomer. **IR**

(ATR): ṽ/cm^{-1} = 2956, 2926, 1599, 1492, 1452, 1249, 1206, 1156, 834, 698. **HRMS (APCI)** for $C_{16}H_{26}BrSi^{+}$ [M+H]$^{+}$: calculated 325.0987, found 325.0989.

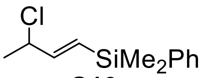

S13a

$C_{12}H_{17}ClSi$

M = 224.08 g/mol

***rac*-(1*E*)-(3-Chlorobut-1-en-1-yl)dimethyl(phenyl)silane** (**S13a**): Prepared from (*E*)-but-2-enal (**S2a**, 0.17 mL, 2.0 mmol, 1.0 equiv) according to **GP6**. Purification by flash column chromatography on silica gel using cyclohexane afforded **S13a** as a yellow oil (300 mg, 60% yield).

R$_f$ = 0.80 (cyclohexane/ethyl acetate = 20/1). **^{1}H NMR** (500 MHz, $CDCl_3$): δ 0.36 (s, 6H), 1.59 (d, *J* = 6.6 Hz, 3H), 4.52 (qui, *J* = 6.7 Hz, 1H), 5.99 (dd, *J* = 18.4, 1.0 Hz, 1H), 6.14 (dd, *J* = 18.4, 6.4 Hz, 1H), 7.34–7.37 (m, 6H), 7.49–7.51 ppm. **^{13}C NMR** (126 MHz, $CDCl_3$): δ –2.8, –2.7, 24.6, 59.6, 127.7, 127.9, 129.2, 133.8, 138.1, 148.0 ppm. **^{29}Si DEPT NMR** (99 MHz, $CDCl_3$): δ –10.92 ppm. **IR (ATR)**: ṽ/cm^{-1} = 3067, 2956, 1249, 1114, 984, 824, 786, 729, 697. **HRMS (APCI)** for $C_6H_{12}ClSi^{+}$ [M-C_6H_5]$^{+}$: calculated 147.0397, found 147.0394.

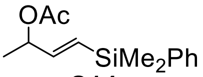

S14a

$C_{14}H_{20}O_2Si$

M = 248.12 g/mol

***rac*-(1*E*)-(3-Acetoxy-1-en-1-yl)dimethyl(phenyl)silane** (**S14a**): Prepared from (*E*)-but-2-enal (**S2a**, 0.17 mL, 2.0 mmol, 1.0 equiv) according to **GP7**. Purification by flash column chromatography on silica gel using cyclohexane/ethyl acetate = 40/1 afforded **S14a** as a yellow oil (360 mg, 60% yield).

R$_f$ = 0.44 (cyclohexane/ethyl acetate = 20/1). **^{1}H NMR** (500 MHz, $CDCl_3$): δ 0.32 (s, 3H), 0.33 (s, 3H), 1.66 (d, *J* = 5.2 Hz, 3H), 2.01 (s, 3H), 5.31–5.32 (m, 1H), 5.43–5.45 (m, 2H), 7.34–7.39 (m, 3H), 7.50–7.52 (m, 2H) ppm. **^{13}C NMR** (126 MHz, $CDCl_3$): δ –5.5, –5.3, 17.8, 21.1, 69.7, 125.2, 127.2, 127.7, 129.5, 134.2, 135.7, 170.7 ppm. **^{29}Si DEPT NMR** (99 MHz, $CDCl_3$): δ –4.57 ppm. **IR (ATR)**: ṽ/cm^{-1} = 3023, 2960, 1736, 1427, 1366, 1226, 114, 1013, 962, 807, 773, 733, 697. **HRMS (APCI)** for $C_{14}H_{21}O_2Si^{+}$ [M-C_6H_5]$^{+}$: calculated 249.1311, found 249.1308.

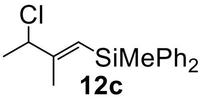

12c

$C_{18}H_{21}ClSi$

M = 300.11 g/mol

***rac*-(1*E*)-(3-Chloro-2-methylbut-1-en-1-yl)(methyl)diphenylsilane** (**10a**): Prepared from (*E*)-2-methylbut-2-enal (**S2c**, 424 mg, 1.5 mmol, 1.0 equiv) according to **GP6**. Purification by flash column chromatography on silica gel using cyclohexane afforded **10a** as a colourless oil (316 mg, 70% yield).

R$_f$ = 0.80 (cyclohexane/ethyl acetate = 20/1). **^{1}H NMR** (500 MHz, $CDCl_3$): δ 0.67 (s, 3H), 1.36 (d, *J* = 6.8 Hz, 3H), 1.75 (s, *J* = 0.8 Hz, 1H), 4.61 (q, *J* = 14.2, 6.8 Hz, 1H), 5.89 (s, 1H), 7.34–7.38 (m, 6H), 7.52–7.54 (m, 4H) ppm. **^{13}C NMR** (126 MHz, $CDCl_3$): δ –2.4, 17.5, 23.8, 64.9, 122.5, 127.9, 129.2, 134.6, 136.9, 156.8 ppm. **^{29}Si DEPT NMR** (99 MHz, $CDCl_3$): δ –18.38 ppm. **IR (ATR)**: ṽ/cm^{-1} = 3066, 2974, 1609, 1375, 1107, 1059, 788, 720, 695. **HRMS (LIFDI)** for $C_{18}H_{22}ClSi^{+}$ [M+H]$^{+}$: calculated 301.1179, found 301.1179.

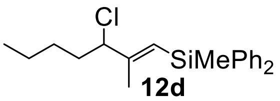

12d

$C_{21}H_{27}ClSi$

M = 342.16 g/mol

***rac*-(1*E*)-(3-Chloro-2-methylhept-1-en-1-yl)(methyl)diphenyl silane** (**10b**): Prepared from (*E*)-2-methylhept-2-enal (**S2d**, 252.4 mg, 2.0 mmol, 1.0 equiv) according to **GP6**. Purification by flash column chromatography on silica gel using cyclohexane afforded **10b** as a colourless oil (357 mg, 52% yield).

R_f = 0.76 (cyclohexane/ethyl acetate = 20/1). **^{1}H NMR** (500 MHz, $CDCl_3$): δ 0.68 (s, 3H), 0.93 (t, *J* = 6.9 Hz, 3H), 1.33–1.45 (m, 4H), 1.71 (d, *J* = 0.7 Hz, 3H), 1.84–1.89 (m, 2H), 4.43 (t, *J* = 7.4 Hz, 1H), 5.87 (s, 1H), 7.34–7.39 (m, 6H), 7.52–7.55 (m, 4H) ppm. **^{13}C NMR** (126 MHz, $CDCl_3$): δ –2.4, 14.0, 16.9, 22.2, 29.1, 36.4, 70.4, 124.0, 127.9, 129.2, 129.2, 134.6, 134.6, 136.9, 137.0, 155.8 ppm. **^{29}Si DEPT NMR** (99 MHz, $CDCl_3$): δ –18.47 ppm. **IR (ATR)**: $\tilde{\nu}$/cm^{-1} = 3067, 2957, 1599, 1426, 1249, 1108, 787, 722, 696. **HRMS (APCI)** for $C_{21}H_{28}ClSi^+$ $[M-C_6H_5]^+$: calculated 265.1179, found 265.1171.

5. Experimental Details for the Nickel-Catalyzed Cross-Coupling

5.1. General Procedure for the Nickel-Catalyzed Cross-Coupling of Silylated Allylbromides (GP8)

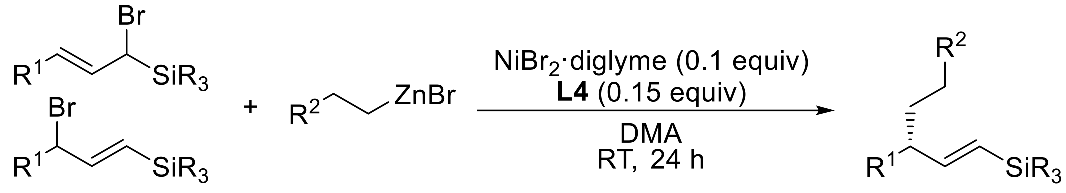

rac-**1** and *rac*-**4**–**7** **2** (2.0 equiv) **3** and **8**–**11**

Scheme S8. Preparation of chiral vinylsilanes.

A flame-dried Schlenk tube equipped with a magnetic stir bar was charged with silylated allylbromide **1** and **4**–**7** as mixture of regioisomers at the α- and γ- position (0.10 mmol, 1.0 equiv), followed by $NiBr_2$·diglyme (3.5 mg, 0.01 mmol, 10.0 mol%) and (*S*,*S*)-*s*Bu-Pybox (**L4**, 4.9 mg, 0.015 mmol, 15.0 mol%). The reaction tube was capped with a septum and wrapped with teflon tape. Then, the tube was placed under high vacuum on a Schlenk line for 20 min and backfilled with N_2 (3 times), followed by the addition of anhydrous DMA (1.0 mL). After stirring for 30 min at room temperature, the reaction mixture appeared orange. Next, the corresponding alkylzinc bromide reagent **2** (0.20 mmol, 2.0 equiv) was added dropwise, leading to a dark brown/black reaction mixture and stirred at ~800 rpm for 24 h. After completion of the reaction, the reaction mixture was directly transferred to a column of silica gel without additional work-up.

5.2. General Procedure for the Nickel-Catalyzed Cross-Coupling of Silylated Allylchlorides (GP9)

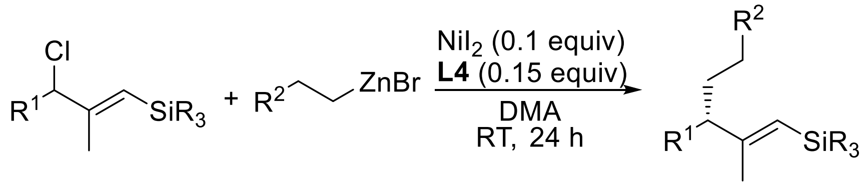

rac-**12** **2** (2.0 equiv) **5**

Scheme S9. Preparation of chiral β-methyl-substituted vinylsilanes.

A flame-dried Schlenk tube equipped with a magnetic stir bar was charged with the silylated allylchloride **12** (0.10 mmol, 1.0 equiv), followed by NiI_2 (3.1 mg, 0.01 mmol, 10.0 mol%) and (*S*,*S*)-*s*Bu-Pybox (**L4**, 4.9 mg, 0.015 mmol, 15.0 mol%). The reaction tube was capped with a septum and wrapped with teflon tape. Then, the tube was placed under high vacuum on a Schlenk line for 20 min and backfilled with N_2 (3 times),

followed by the addition of anhydrous DMA (1.0 mL). After stirring for 30 min at room temperature, the reaction mixture appeared orange. Next, the corresponding alkylzinc bromide reagent **2** (0.20 mmol, 2.0 equiv) was added dropwise, leading to a dark brown/black reaction mixture and stirred at ~800 rpm for 24 h. After completion of the reaction, the reaction mixture was directly transferred to a column of silica gel without additional work-up.

5.3. Characterization Data of the Chiral Vinylsilanes

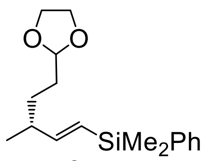

3aa

$C_{17}H_{26}O_2Si$
M = 290.17 g/mol

(–)-(3*R*,1*E*)-(5-(1,3-Dioxolan-2-yl)-3-methylpent-1-en-1-yl) dimethyl(phenyl)silane (**3aa**): Prepared from (*E*)-(1-bromobut-2-en-1-yl)dimethyl(phenyl)silane and (*E*)-(3-bromobut-2-en-1-yl)dimethyl(phenyl)silane (**1a**, 26.9 mg, 0.1 mmol) according to **GP8** with (2-(1,3-dioxolan-2-yl)ethyl)zinc(II) bromide (**2a**, 0.2 mmol) at rt for 24 h. Purification by flash column chromatography on silica gel using cyclohexane/ethyl acetate = 30/1 afforded **3aa** as a yellow oil (23.1 mg, 80% yield).

R$_f$ = 0.29 (cyclohexane/ethyl acetate = 20/1). **^{1}H NMR** (500 MHz, $CDCl_3$): δ 0.31 (s, 6H), 1.02 (d, *J* = 6.6 Hz, 3H), 1.42–1.50 (m, 2H), 1.63–1.68 (m, 2H), 2.17–2.25 (m, 1H), 3.83–3.86 (m, 2H), 3.95-3.98 (m, 2H), 4.85 (t, *J* = 4.6 Hz, 1H), 5.73 (dd, *J* = 18.7, 0.9 Hz, 1H), 5.99 (dd, *J* = 18.7, 7.0 Hz, 1H), 7.33–7.36 (m, 3H), 7.50–7.52 (m, 2H) ppm. **^{13}C NMR** (126 MHz, $CDCl_3$): δ –2.2, –2.4, 19.8, 30.5, 31.7, 40.2, 64.8, 104.7, 125.6, 127.7, 128.8, 133.8, 139.3, 154.2 ppm. **^{29}Si DEPT NMR** (99 MHz, $CDCl_3$): δ –11.78 ppm. **IR (ATR)**: ṽ/cm^{-1} = 2953, 2924, 1612, 1427, 1247, 1110, 992, 821, 729, 696. **HRMS (APCI)** for $C_{18}H_{26}O_2Si^+$ [M+H]$^+$: calculated 291.1775, found 291.1778.

Optical rotation: $[\alpha]_D^{20}$ = –8.7 (c 0.96, $CHCl_3$, e.r. 92:8). The enantiomeric ratio of **3aa** was determined by HPLC analysis on a chiral stationary phase (*Daicel* Chiralcel OD-H column, column temperature 20 °C, solvent *n*-heptane:isopropanol = 99.9:0.1, flow rate 0.4 mL/min): t_R = 27.0 min (minor), t_R = 34.6 min (major).

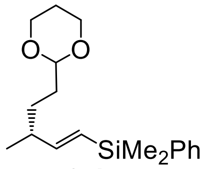

3ab

$C_{18}H_{28}O_2Si$
M = 304.19 g/mol

(–)-(3*R*,1*E*)-(5-(1,3-Dioxan-2-yl)-3-methylpent-1-en-1-yl)dimethyl (phenyl)silane (**3ab**): Prepared from (*E*)-(1-bromobut-2-en-1-yl)dimethyl(phenyl)silane and (*E*)-(3-bromobut-2-en-1-yl)dimethyl(phenyl)silane (**1a**, 26.9 mg, 0.1 mmol) according to **GP8** with (2-(1,3-dioxan-2-yl)ethyl)zinc(II) bromide (**2b**, 0.2 mmol) at rt for 24 h. Purification by flash column chromatography on silica gel using cyclohexane/ethyl acetate = 30/1 afforded **3ab** as a yellow oil (18.8 mg, 62% yield).

R$_f$ = 0.30 (cyclohexane/ethyl acetate = 20/1). **^{1}H NMR** (500 MHz, $CDCl_3$): δ 0.30 (s, 6H), 1.00 (d, *J* = 7.0 Hz, 3H), 1.30–1.36 (m, 2H), 1.38–1.45 (m, 2H), 1.57–1.61 (m, 2H), 2.03–2.11 (m, 1H), 2.12–2.20 (m, 1H), 3.74 (td, *J* = 12.4, 1.6 Hz, 2H), 4.10 (dd, *J* = 11.0, 5.0 Hz, 2H), 4.50 (t, *J* = 5.0 Hz, 1H), 5.71 (dd, *J* = 18.8, 1.1 Hz, 1H), 5.99 (dd, *J* = 18.8, 7.2 Hz, 1H), 7.33–7.35 (m, 3H), 7.49–7.52 (m, 2H) ppm. **^{13}C NMR** (126 MHz, $CDCl_3$): δ –2.4, –2.4, 19.8, 25.8, 30.4, 33.1, 40.2, 66.9, 102.5, 125.4, 127.7, 128.7, 133.8, 139.4, 154.3 ppm. **^{29}Si DEPT NMR** (99 MHz, $CDCl_3$): δ –11.79 ppm. **IR (ATR)**: ṽ/cm^{-1} = 3067, 2954, 2924, 2849, 1613, 1457, 1376, 1247, 1144, 995, 841, 731, 699. **HRMS (APCI)** for $C_{18}H_{29}O_2Si^+$ [M+H]$^+$: calculated 305.1931, found 305.1934.

Optical rotation: $[\alpha]_D^{20}$ = –11.5 (c 0.87, $CHCl_3$, e.r. 92:8). The enantiomeric ratio of **3ab** was determined by HPLC analysis on a chiral stationary phase (*Daicel* Chiralcel OD-H column, column temperature 20 °C, solvent *n*-heptane:isopropanol = 99.9:0.1, flow rate 0.4 mL/min): t_R = 25.4 min (minor), t_R = 38.8 min (major).

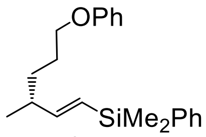

3ac

$C_{21}H_{28}OSi$
M =324.19 g/mol

(–)-(3*R*,1*E*)-Dimethyl(3-methyl-6-phenoxyhex-1-en-1-yl)(phenyl) silane (**3ac**): Prepared from (*E*)-(1-bromobut-2-en-1-yl)dimethyl(phenyl)silane and (*E*)-(3-bromobut-2-en-1-yl)dimethyl(phenyl)silane (**1a**, 26.9 mg, 0.1 mmol) according to **GP8** with (3-phenoxypropyl)zinc(II) bromide (**2c**, 0.2 mmol) at rt for 24 h. Purification by flash column chromatography on silica gel using cyclohexane/diethyl ether = 150/1 afforded **3ac** as a yellow oil (21.5 mg, 66% yield).

$\mathbf{R_f}$ = 0.56 (cyclohexane/diethyl ether = 50/1). **^{1}H NMR** (500 MHz, $CDCl_3$): δ 0.32 (s, 6H), 1.04 (d, *J* = 6.8 Hz, 3H), 1.46–1.53 (m, 2H), 1.75–1.84 (m, 2H), 2.22–2.28 (m, 1H), 3.95 (t, *J* = 6.6 Hz, 2H), 5.75 (dd, *J* = 18.7, 1.2 Hz, 1H), 6.01 (dd, *J* = 18.7, 7.1 Hz, 1H), 6.88–6.95 (m, 3H), 7.26–7.29 (m, 2H), 7.34–7.35 (m, 3H), 7.51–7.53 (m, 2H) ppm. **^{13}C NMR** (126 MHz, $CDCl_3$): δ –2.4, –2.4, 20.0, 27.1, 32.7, 40.2, 67.9, 114.5, 120.5, 125.6, 127.7, 128.8, 129.4, 133.8, 139.3, 154.4, 159.1 ppm. **^{29}Si DEPT NMR** (99 MHz, $CDCl_3$): δ –11.77 ppm. **IR (ATR)**: $\tilde{\nu}$/cm^{-1} = 3067, 2654, 1600, 1496, 1245, 1112, 993, 825, 753, 694. **HRMS (APCI)** for $C_{21}H_{29}OSi^+$ $[M+H]^+$: calculated 325.1988, found 325.1985.

Optical rotation: $[\alpha]_D^{20}$ = –10.8 (c 1.00, $CHCl_3$, e.r. 85:15). The enantiomeric ratio of **3ac** was determined by HPLC analysis on a chiral stationary phase (*Daicel* Chiralcel OD-H column, column temperature 20 °C, solvent *n*-heptane:isopropanol = 99:1, flow rate 0.8 mL/min): t_R = 7.8 min (minor), t_R = 17.8 min (major).

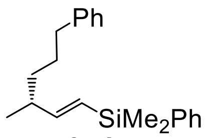

3ad

$C_{21}H_{28}Si$
M =308.20 g/mol

(–)-(3*R*,1*E*)-Dimethyl(3-methyl-6-phenylhex-1-en-1-yl)(phenyl) silane (**3ad**): Prepared from (*E*)-(1-bromobut-2-en-1-yl)dimethyl(phenyl)silane and (*E*)-(3-bromobut-2-en-1-yl)dimethyl(phenyl)silane (**1a**, 26.9 mg, 0.1 mmol) according to **GP8** with (3-phenylpropyl)zinc(II) bromide (**2d**, 0.2 mmol) at rt for 24 h. Purification by flash column chromatography on silica gel using cyclohexane afforded **3ad** as a yellow oil (87% NMR yield, internal standard: mesitylene).

$\mathbf{R_f}$ = 0.29 (cyclohexane). **^{1}H NMR** (500 MHz, $CDCl_3$): δ 0.31 (s, 6H), 0.99 (d, *J* = 7.9 Hz, 3H), 1.32–1.38 (m, 2H), 1.58–1.64 (m, 2H), 2.18–2.23 (m, 1H), 2.58 (t, *J* = 7.7 Hz, 2H), 5.70 (d, *J* = 18.5 Hz, 1H), 5.97 (dd, *J* = 18.5, 7.2 Hz, 1H), 7.14–7.18 (m, 3H), 7.24–7.28 (m, 2H), 7.31–7.34 (m, 3H), 7.50–7.51 (m, 2H) ppm. **^{13}C NMR** (126 MHz, $CDCl_3$): δ –2.4, 19.9, 29.2, 36.0, 40.3, 125.2, 125.6, 127.7, 128.2, 128.4, 128.8, 133.8, 139.4, 142.7, 154.7 ppm. **^{29}Si DEPT NMR** (99 MHz, $CDCl_3$): δ –11.81 ppm. **IR (ATR)**: $\tilde{\nu}$/cm^{-1} = 3065, 2927, 2854, 1613, 1454, 1248, 1112, 992, 824, 731, 698. **HRMS (APCI)** for $C_{21}H_{29}Si^+$ $[M+H]^+$: calculated 309.2039, found 309.2034.

Optical rotation: $[\alpha]_D^{20}$ = –11.2 (c 0.94, $CHCl_3$, e.r. 95:5). The enantiomeric ratio of **3ad** was determined by HPLC analysis on a chiral stationary phase (*Daicel* Chiralcel OD-H column, column temperature 20 °C, solvent *n*-heptane:isopropanol = 90:10, flow rate 0.8 mL/min): t_R = 4.3 min (major), t_R = 5.5 min (minor).

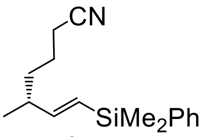

3ae

$C_{16}H_{23}NSi$

M =257.16 g/mol

(–)-(3*R*,1*E*)-(6-Cyano-3-methylhex-1-en-1-yl)dimethyl(phenyl) silane (**3ae**): Prepared from (*E*)-(1-bromobut-2-en-1-yl)dimethyl(phenyl)silane and (*E*)-(3-bromobut-2-en-1-yl)dimethyl(phenyl)silane (**1a**, 26.9 mg, 0.1 mmol) according to **GP8** with (3-cyanopropyl)zinc(II) bromide (**2e**, 0.2 mmol) at rt for 24 h. Purification by flash column chromatography on silica gel using cyclohexane/ethyl acetate = 30/1 afforded **3ae** as a yellow oil (22.7 mg, 88% yield).

R_f = 0.27 (cyclohexane/ethyl acetate = 20/1). **^{1}H NMR** (500 MHz, $CDCl_3$): δ 0.33 (s, 6H), 1.03 (d, *J* = 6.9 Hz, 3H), 1.43–1.49 (m, 2H), 1.60–1.67 (m, 2H), 2.19–2.24 (m, 1H), 2.32 (t, *J* = 7.2 Hz, 2H), 5.74 (d, *J* = 18.8 Hz, 1H), 5.94 (dd, *J* = 18.8, 6.9 Hz, 1H), 7.34–7.36 (m, 3H), 7.50–7.52 (m, 2H) ppm. **^{13}C NMR** (126 MHz, $CDCl_3$): δ –2.5, –2.5, 17.2, 19.9, 23.3, 35.2, 39.9, 76.7, 77.0, 77.3, 119.7, 126.6, 127.7, 128.9, 133.8, 139.0, 153.3 ppm. **^{29}Si DEPT NMR** (99 MHz, $CDCl_3$): δ –11.72 ppm. **IR (ATR)**: $\tilde{v}$/cm^{-1} = 3067, 2956, 2870, 2245, 1614, 1426, 1248, 1111, 993, 824, 732, 700. **HRMS (APCI)** for $C_{16}H_{24}NSi^+$ $[M+H]^+$: calculated 258.1678, found 258.1667.

Optical rotation: $[\alpha]_D^{20}$ = –12.8 (c 0.94, $CHCl_3$, e.r. 82:18). The enantiomeric ratio of **3ae** was determined by HPLC analysis on a chiral stationary phase (*Daicel* Chiralcel AS-H column, column temperature 20 °C, solvent *n*-heptane:isopropanol = 98:2, flow rate 0.4 mL/min): t_R = 13.5 min (miror), t_R = 14.2 min (major).

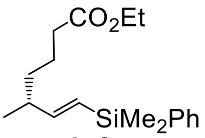

3af

$C_{18}H_{28}O_2Si$

M =304.19 g/mol

(–)-(3*R*,1*E*)-(7-Ethoxy-7-oxo-3-methylhept-1-en-1-yl)dimethyl (phenyl)silane (**3af**): Prepared from (*E*)-(1-bromobut-2-en-1-yl)dimethyl(phenyl)silane and (*E*)-(3-bromobut-2-en-1-yl)dimethyl(phenyl)silane (**1a**, 26.9 mg, 0.1 mmol) according to **GP8** with (4-ethoxy-4-oxobutyl)zinc(II) bromide (**2f**, 0.2 mmol) at rt for 24 h. Purification by flash column chromatography on silica gel using cyclohexane/ethyl acetate = 40/1 afforded **3af** as a yellow oil (25.3 mg, 83% yield).

R_f = 0.48 (cyclohexane/ethyl acetate = 20/1). **^{1}H NMR** (500 MHz, $CDCl_3$): δ 0.31 (s, 6H), 1.00 (d, *J* = 6.6 Hz, 3H), 1.25 (t, *J* = 7.0 Hz, 3H), 1.29–1.38 (m, 3H), 1.62 (qui, *J* = 7.8 Hz, 2H), 2.16–2.22 (m, 1H), 2.28 (t, *J* = 7.7 Hz, 2H), 4.12 (q, *J* = 15.5, 7.1 Hz, 1H), 5.72 (d, *J* = 18.5 Hz, 1H), 5.98 (dd, *J* = 18.5, 7.2 Hz, 1H), 7.34–7.35 (m, 3H), 7.50–7.52 (m, 2H) ppm. **^{13}C NMR** (126 MHz, $CDCl_3$): δ –2.4, –2.4, 14.3, 19.8, 22.8, 34.4, 35.8, 40.1, 60.2, 76.7, 77.0, 77.3, 125.5, 127.7, 128.8, 133.8, 139.3, 154.3, 173.7 ppm. **^{29}Si DEPT NMR** (99 MHz, $CDCl_3$): δ –11.78 ppm. **IR (ATR)**: $\tilde{v}$/cm^{-1} = 3067, 2956, 1735, 1614, 1372, 1247, 1112, 993, 824, 731, 699. **HRMS (APCI)** for $C_{18}H_{29}O_2Si^+$ $[M+H]^+$: calculated 291.1780, found 291.1959.

Optical rotation: $[\alpha]_D^{20}$ = –7.9 (c 0.95, $CHCl_3$, e.r. 84:16). The enantiomeric ratio of **3af** was determined by HPLC analysis on a chiral stationary phase (*Daicel* Chiralcel OD-H column, column temperature 20 °C, solvent *n*-heptane:isopropanol = 99.9:0.1, flow rate 0.4 mL/min): t_R = 27.0 min (major), t_R = 29.1 min (minor).

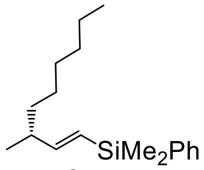

3ag

$C_{18}H_{30}Si$

M =274.21 g/mol

(–)-(3*R*,1*E*)-Dimethyl(3-methylnon-1-en-1-yl)(phenyl)silane (**3ag**): Prepared from (*E*)-(1-bromobut-2-en-1-yl)dimethyl(phenyl)silane and (*E*)-(3-bromobut-2-en-1-yl)dimethyl(phenyl)silane (**1a**, 26.9 mg, 0.1 mmol) according to **GP8** with (1-hexyl)zinc(II) bromide (**2g**, 0.2 mmol) at rt for 24 h. Purification by flash column chromatography on silica gel using cyclohexane afforded **3ag** as a yellow oil (22.5 mg, 82% yield).

R_f = 0.63 (cyclohexane). **^{1}H NMR** (500 MHz, $CDCl_3$): δ 0.31 (s, 6H), 0.88 (t, *J* = 6.7 Hz, 3H), 0.98 (d, *J* = 6.7 Hz, 3H), 1.26 (broad s, 10H), 2.13–2.17 (m, 1H), 5.68 (d, *J* = 18.5 Hz, 1H), 5.99 (dd, *J* = 18.5, 7.3 Hz, 1H), 7.32–7.34 (m, 3H), 7.50–7.52 (m, 2H) ppm. **^{13}C NMR** (126 MHz, $CDCl_3$): δ –2.4, –2.4, 14.1, 19.9, 22.6, 27.2, 29.4, 31.8, 36.4, 40.3, 124.7, 127.6, 128.7, 133.8, 139.5, 155.1 ppm. **^{29}Si DEPT NMR** (99 MHz, $CDCl_3$): δ –11.84 ppm. **IR (ATR)**: $\tilde{\nu}$/cm^{-1} = 3067, 2955, 2923, 2853, 1613, 1457, 1427, 1247, 1112, 991, 824, 729, 698. **HRMS (APCI)** for $C_{18}H_{31}Si^+$ $[M+H]^+$: calculated 275.2190, found 275.2186.

Optical rotation: $[\alpha]_D^{20}$ = –11.0 (c 0.95, $CHCl_3$, e.r. 78:22). The enantiomeric ratio of **3ag** was determined by reversed phase HPLC analysis on a chiral stationary phase (*Daicel* Chiralcel AD-RH column, column temperature 20 °C, solvent acetonitrile:water = 65:35, flow rate 0.3 mL/min): t_R = 37.1 min (major), t_R = 39.5 min (minor).

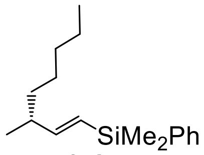

3ah

$C_{17}H_{28}Si$

M =260.20 g/mol

(–)-(3*R*,1*E*)-Dimethyl(3-methyloct-1-en-1-yl)(phenyl)silane (**3ah**): Prepared from (*E*)-(1-bromobut-2-en-1-yl)dimethyl(phenyl)silane and (*E*)-(3-bromobut-2-en-1-yl)dimethyl(phenyl)silane (**1a**, 26.9 mg, 0.1 mmol) according to **GP8** with (1-pentyl)zinc(II) bromide (**2h**, 0.2 mmol) at rt for 24 h. Purification by flash column chromatography on silica gel using *n*-pentane afforded **3ah** as a yellow oil (19.2 mg, 74% yield).

R_f = 0.85 (*n*-pentane). **^{1}H NMR** (500 MHz, $CDCl_3$): δ 0.32 (s, 6H), 0.88 (t, *J* = 6.7 Hz, 3H), 0.99 (d, *J* = 7.2 Hz, 3H), 1.27–1.32 (m, 8H), 2.15–2.19 (m, 1H), 5.69 (dd, *J* = 18.7, 1.36 Hz, 1H), 6.00 (dd, *J* = 18.5, 6.8 Hz, 1H), 7.33–7.36 (m, 3H), 7.51–7.53 (m, 2H) ppm. **^{13}C NMR** (126 MHz, $CDCl_3$): δ –2.4, –2.4, 14.1, 19.9, 22.6, 26.9, 32.0, 36.4, 40.3, 124.7, 127.6, 128.7, 133.8, 139.6, 155.2 ppm. **^{29}Si DEPT NMR** (99 MHz, $CDCl_3$): δ –11.83 ppm. **IR (ATR)**: $\tilde{\nu}$/cm^{-1} = 3067, 2955, 2924, 2854, 1613, 1457, 1427, 1247, 1111, 991, 822, 729, 697. **HRMS (APCI)** for $C_{17}H_{29}Si^+$ $[M+H]^+$: calculated 261.2039, found 261.2033.

Optical rotation: $[\alpha]_D^{20}$ = –11.0 (c 1.11, $CHCl_3$, e.r. 76:24). The enantiomeric ratio of **3ah** was determined by reversed phase HPLC analysis on a chiral stationary phase (*Daicel* Chiralcel AD-RH column, column temperature 20 °C, solvent acetonitrile:water = 65:35, flow rate 0.3 mL/min): t_R = 32.6 min (major), t_R = 35.5 min (minor).

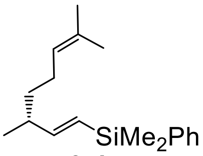

3ai

$C_{18}H_{28}Si$

M =272.20 g/mol

(–)-(3*R*,1*E*)-(3,7-Dimethylocta-1,6-dien-1-yl)dimethyl(phenyl) silane (**3ai**): Prepared from (*E*)-(1-bromobut-2-en-1-yl)dimethyl(phenyl)silane and (*E*)-(3-bromobut-2-en-1-yl)dimethyl(phenyl)silane (**1a**, 26.9 mg, 0.1 mmol) according to **GP8** with (5-(2-methylpent-2-enyl))zinc(II) bromide (**2i**, 0.2 mmol) at rt for 24 h. Purification by flash column chromatography on silica gel using cyclohexane afforded **3ai** as a yellow oil (20.5 mg, 75% yield).

R$_f$ = 0.57 (cyclohexane). **^{1}H NMR** (500 MHz, $CDCl_3$): δ 0.31 (s, 6H), 1.00 (d, *J* = 7.0 Hz, 3H), 1.27–1.39 (m, 2H), 1.58 (s, 3H), 1.68 (s, 3H), 1.96 (q, *J* = 16.6, 7.7 Hz, 2H), 2.14–2.22 (m, 1H), 5.10 (t, *J* = 7.7 Hz, 1H), 5.70 (d, *J* = 18.8 Hz, 1H), 5.99 (dd, *J* = 18.8, 7.0 Hz, 1H), 7.33–7.34 (m, 3H), 7.51–7.53 (m, 2H) ppm. **^{13}C NMR** (126 MHz, $CDCl_3$): δ –2.4, –2.4, 17.6, 19.9, 25.7, 25.8, 36.6, 39.9, 124.7, 125.1, 127.7, 128.8, 131.3, 133.8, 139.5, 154.9 ppm. **^{29}Si DEPT NMR** (99 MHz, $CDCl_3$): δ –11.82 ppm. **IR (ATR)**: ṽ/cm^{-1} = 3067, 2958, 2921, 2853, 1613, 1453, 1248, 1112, 991, 824, 730, 698. **HRMS (APCI)** for $C_{18}H_{29}Si^+$ $[M+H]^+$: calculated 273.2039, found 273.2042.

Optical rotation: $[\alpha]_D^{20}$ = –18.0 (c 0.64, $CHCl_3$, e.r. 79:21). The enantiomeric ratio of **3ai** was determined by reversed phase HPLC analysis on a chiral stationary phase (*Daicel* Chiralcel AD-RH column, column temperature 20 °C, solvent acetonitrile:water = 65:35, flow rate 0.3 mL/min): t_R = 27.0 min (major), t_R = 29.8 min (minor).

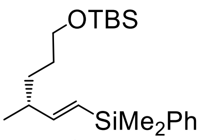

3aj

$C_{21}H_{38}OSi_2$

M =262.25 g/mol

(–)-(3*R*,1*E*)-*tert*-Butyl((6-(dimethyl(phenyl)silyl)-4-methylhex-5-en-1-yl) oxy)dimethylsilane (**3aj**): Prepared from (*E*)-(1-bromobut-2-en-1-yl)dimethyl(phenyl)silane and (*E*)-(3-bromobut-2-en-1-yl)dimethyl(phenyl)silane (**1a**, 26.9 mg, 0.1 mmol) according to **GP8** with ((3-propoxy)(tert-butyl)dimethylsilyl)zinc(II) bromide (**2j**, 0.2 mmol) at rt for 24 h. Purification by flash column chromatography on silica gel using cyclohexane/ethyl acetate = 30/1 afforded **3aj** as a yellow oil (22.1 mg, 61% yield).

R$_f$ = 0.44 (cyclohexane/ethyl acetate = 20/1). **^{1}H NMR** (500 MHz, $CDCl_3$): δ 0.04 (s, 6H), 0.31 (s, 6H), 0.89 (s, 9H), 1.00 (d, *J* = 6.8 Hz, 3H), 1.31–1.36 (m, 2H), 1.48–1.52 (m, 2H), 2.15–2.21 (m, 1H), 6.5 (t, J = Hz, 2H), 5.70 (dd, *J* = 18.6, 1.0 Hz, 1H), 5.99 (dd, *J* = 18.6, 7.0 Hz, 1H), 7.33–7.35 (m, 3H), 7.50–7.52 (m, 2H) ppm. **^{13}C NMR** (126 MHz, $CDCl_3$): δ –5.25, –2.4, –2.4, 18.4, 20.0, 25.6, 26.0, 30.6, 32.5, 32.9, 40.1, 63.4, 76.7, 77.0, 77.2, 125.1, 127.7, 128.8, 133.8, 139.5, 154.8 ppm. **^{29}Si DEPT NMR** (99 MHz, $CDCl_3$): δ –11.84, –18.40 ppm. **IR (ATR)**: ṽ/cm^{-1} = 3067, 2953, 2856, 1614, 1462, 1250, 1098, 835, 775, 730, 698. **HRMS (APCI)** for $C_{28}H_{39}OSi_2^+$ $[M+H]^+$: calculated 363.2534, found 363.2535.

Optical rotation: $[\alpha]_D^{20}$ = –5.4 (c 0.97, $CHCl_3$, e.r. 75:25). The enantiomeric ratio of **3aj** was determined, after deprotection of TBS group with TBAF,[S12] by HPLC analysis on a chiral stationary phase (*Daicel* Chiralcel OD-H column, column temperature 20 °C, solvent *n*-heptane:isopropanol = 99:1, flow rate 0.4 mL/min): t_R = 27.1 min (minor), t_R = 31.1 min (major).

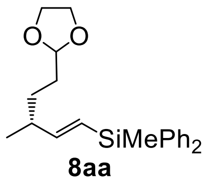

8aa

$C_{22}H_{28}O_2Si$

M = 352.19 g/mol

(–)-(3*R*,1*E*)-(5-(1,3-Dioxolan-2-yl)-3-methylpent-1-en-1-yl)(methyl)diphenylsilane (**8aa**): Prepared from (*E*)-(1-bromobut-2-en-1-yl)(methyl)phenylsilane and (*E*)-(3-bromobut-2-en-1-yl)(methyl)phenylsilane (**4a**, 26.9 mg, 0.1 mmol) according to **GP8** with (2-(1,3-dioxolan-2-yl)ethyl)zinc(II) bromide (**2a**, 0.2 mmol) at rt for 24 h. Purification by flash column chromatography on silica gel using cyclohexane/ethyl acetate = 30/1 afforded **8aa** as a yellow oil (30.6 mg, 87% yield).

R$_f$ = 0.31 (cyclohexane/ethyl acetate = 20/1). **^{1}H NMR** (500 MHz, $CDCl_3$): δ 0.60 (s, 3H), 1.04 (d, *J* = 6.6 Hz, 3H), 1.42–1.52 (m, 2H), 1.65–1.69 (m, 2H), 2.23–2.31 (m, 1H), 3.83–3.88 (m, 2H), 3.92–3.97 (m, 2H), 4.85, (t, *J* = 4.9 Hz, 1H), 5.92 (d, *J* = 18.6 Hz, 1H), 6.05 (dd, *J* = 18.6, 6.7 Hz, 1H), 7.33–7.39 (m, 3H), 7.50–7.52 (m, 2H) ppm. **^{13}C NMR** (126 MHz, $CDCl_3$): δ –3.6, 19.8, 30.4, 31.7, 40.3, 64.8, 104.6, 123.4, 127.7, 129.1, 134.8, 137.1, 156.4 ppm. **^{29}Si DEPT NMR** (99 MHz, $CDCl_3$): δ –15.25 ppm. **IR (ATR)**: $\tilde{\nu}$/cm^{-1} = 3067, 2953, 2924, 2870, 1613, 1427, 1250, 1111, 995, 798, 735, 699. **HRMS (APCI)** for $C_{22}H_{29}O_2Si^+$ [M+H]$^+$: calculated 353.1931, found 353.1937.

Optical rotation: $[\alpha]_D^{20}$ = –9.6 (c 0.95, $CHCl_3$, e.r. 93:7). The enantiomeric ratio of **8aa** was determined by HPLC analysis on a chiral stationary phase (*Daicel* Chiralcel OD-H column, column temperature 20 °C, solvent *n*-heptane:isopropanol = 99.9:0.1, flow rate 0.4 mL/min): t_R = 48.4 min (minor), t_R = 58.8 min (major).

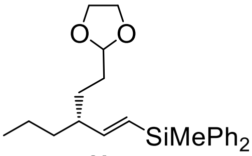

8ba

$C_{24}H_{32}O_2Si$

M = 380.22 g/mol

(+)-(3*R*,1*E*)-(3-(2-(1,3-Dioxolan-2-yl)ethyl)hex-1-en-1-yl)(methyl)diphenylsilane (**8ba**): Prepared from (*E*)-(1-bromohex-1-en-1-yl)(methyl)diphenylsilane and (*E*)-(3-bromohex-1-en-1-yl)(methyl)diphenylsilane (**4b**, 35.9 mg, 0.1 mmol) according to **GP8** with (2-(1,3-dioxolan-2-yl)ethyl)zinc(II) bromide (**2a**, 0.2 mmol) at rt for 24 h. Purification by flash column chromatography on silica gel using cyclohexane/ethyl acetate = 30/1 afforded **8ba** as a yellow oil (28.5 mg, 75% yield).

R$_f$ = 0.30 (cyclohexane/ethyl acetate = 20/1). **^{1}H NMR** (500 MHz, $CDCl_3$): δ 0.59 (s, 3H), 0.88 (t, *J* = 6.7 Hz, 3H), 1.25–1.43 (m, 5H), 1.49–1.54 (m, 1H), 1.58–72 (m, 2H), 2.11–2.17 (m, 1H), 3.82–3.87 (m, 2H), 3.92–3.97 (m, 2H), 4.84, (t, *J* = 4.7 Hz, 1H), 5.88–5.90 (m, 2H), 7.33–7.37 (m, 6H), 7.51–7.52 (m, 4H) ppm. **^{13}C NMR** (126 MHz, $CDCl_3$): δ –3.5, 14.1, 20.3, 28.8, 31.7, 36.9, 46.7, 64.8, 64.8, 104.7, 125.5, 127.7, 129.1, 134.8, 137.1, 155.3 ppm. **^{29}Si DEPT NMR** (99 MHz, $CDCl_3$): δ –15.43 ppm. **IR (ATR)**: $\tilde{\nu}$/cm^{-1} = 3067, 2953, 2925, 2870, 1613, 1427, 1250, 1111, 996, 799, 735, 716. **HRMS (APCI)** for $C_{24}H_{33}O_2Si^+$ [M+H]$^+$: calculated 381.2250, found 381.2241.

Optical rotation: $[\alpha]_D^{20}$ = +0.6 (c 1.00, $CHCl_3$, e.r. 89:11). The enantiomeric ratio of **8ba** was determined by HPLC analysis on a chiral stationary phase (*Daicel* Chiralcel OD-H column, column temperature 20 °C, solvent *n*-heptane:isopropanol = 99.9:0.1, flow rate 0.4 mL/min): t_R = 37.3 min (minor), t_R = 40.2 min (major).

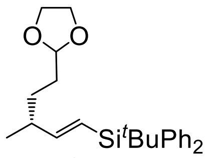

9aa

$C_{25}H_{34}O_2Si$
M = 394.23 g/mol

(–)-(3*R*,1*E*)-(5-(1,3-Dioxolan-2-yl)-3-methylpent-1-en-1-yl)(*tert*-butyl)diphenylsilane (**9aa**): Prepared from (*E*)-(3-bromobut-1-en-1-yl)(*tert*-butyl)diphenylsilane (**5a**, 26.9 mg, 0.1 mmol) according to **GP8** with (2-(1,3-dioxolan-2-yl)ethyl)zinc(II) bromide (**2a**, 0.2 mmol) at rt for 24 h. Purification by flash column chromatography on silica gel using cyclohexane/ethyl acetate = 30/1 afforded **9aa** as a yellow oil (29.1 mg, 80% yield).

R_f = 0.42 (cyclohexane/ethyl acetate = 20/1). **^{1}H NMR** (500 MHz, $CDCl_3$): δ 1.04 (d, *J* = 6.7 Hz, 3H), 1.06 (s, 9H), 1.43–1.47 (m, 2H), 1.65–1.69 (m, 2H), 2.27–2.33 (m, 1H), 3.83–3.86 (m, 2H), 3.94–3.97 (m, 2H), 4.85 (t, *J* = 4.7 Hz, 1H), 5.93-6.03 (m, 2H), 7.32–7.39 (m, 6H), 7.59–7.60 (m, 4H) ppm. **^{13}C NMR** (126 MHz, $CDCl_3$): δ 12.1, 20.0, 27.7, 30.4, 31.8, 40.8, 64.8, 104.7, 121.1, 127.5, 129.0, 135.0, 136.2, 158.0 ppm. **^{29}Si DEPT NMR** (99 MHz, $CDCl_3$): δ –9.62 ppm. **IR (ATR)**: ṽ/cm^{-1} = 2954, 2855, 1614, 1427, 1135, 1106, 998, 820, 739, 701. **HRMS (APCI)** for $C_{25}H_{35}O_2Si^+$ [M+H]$^+$: calculated 395.2406, found 395.2401.

Optical rotation: $[\alpha]_D^{20}$ = –14.6 (c 1.01, $CHCl_3$, e.r. 93:7). The enantiomeric ratio of **9aa** was determined by HPLC analysis on a chiral stationary phase (*Daicel* Chiralcel OD-H column, column temperature 20 °C, solvent *n*-heptane:isopropanol = 99.9:0.1, flow rate 0.5 mL/min): t_R = 28.9 min (minor), t_R = 32.2 min (major).

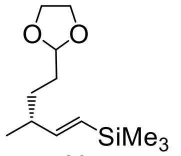

10aa

$C_{12}H_{24}O_2Si$
M = 228.15 g/mol

(*S*)-(3*R*,1*E*)-(5-(1,3-Dioxolan-2-yl)-3-methylpent-1-en-1-yl)trimethylsilane (**10aa**): Prepared from (*E*)-(1-bromobut-2-en-1-yl)trimethylsilane and (*E*)-(3-bromobut-2-en-1-yl)trimethylsilane (**6a**, 26.9 mg, 0.1 mmol) according to **GP8** with (2-(1,3-dioxolan-2-yl)ethyl)zinc(II) bromide (**2a**, 0.2 mmol) at rt for 24 h. Purification by flash column chromatography on silica gel using cyclohexane/ethyl acetate = 30/1 afforded **10aa** as a yellow oil (15.5 mg, 68% yield).

R_f = 0.32 (cyclohexane/ethyl acetate = 20/1). **^{1}H NMR** (500 MHz, $CDCl_3$): δ 0.04 (s, 9H), 0.99 (d, *J* = 6.7 Hz, 3H), 1.39–1.44 (m, 2H), 1.60–1.66 (m, 2H), 2.11–2.17 (m, 1H), 3.83–3.86 (m, 2H), 3.95–3.98 (m, 2H), 4.84 (t, *J* = 4.8 Hz, 1H), 5.58 (dd, *J* = 18.5, 1.0 Hz, 1H), 5.86 (dd, *J* = 18.5, 7.0 Hz, 1H) ppm. **^{13}C NMR** (126 MHz, $CDCl_3$): δ –1.1, 19.9, 30.5, 31.7, 40.1, 64.8, 104.7, 127.9, 152.1 ppm. **^{29}Si DEPT NMR** (99 MHz, $CDCl_3$): δ –7.87 ppm. **IR (ATR)**: ṽ/cm^{-1} = 2953, 2874, 1614, 1246, 1134, 991, 836, 739, 691. **HRMS (APCI)** for $C_{12}H_{25}O_2Si^+$ [M+H]$^+$: calculated 229.1624, found 229.1621.

Optical rotation: $[\alpha]_D^{20}$ = –9.1 (c 1.00, $CHCl_3$, e.r. 94:6). The enantiomeric ratio of **10aa** was determined by HPLC analysis on a chiral stationary phase (*Daicel* Chiralcel OD-H column, column temperature 20 °C, solvent *n*-heptane:isopropanol = 99.9:0.1, flow rate 0.3 mL/min): t_R = 25.3 min (minor), t_R = 33.3 min (major).

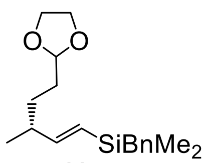

11aa

$C_{18}H_{28}O_2Si$
M = 304.19 g/mol

(–)-(3*R*,1*E*)-(5-(1,3-Dioxolan-2-yl)-3-methylpent-1-en-1-yl)(benzyl) dimethylsilane (**11aa**): Prepared from (*E*)-benzyl(1-bromobut-2-en-1-yl)dimethylsilane and (*E*)-benzyl(1-bromobut-2-en-1-yl)dimethylsilane (**7a**, 28.3 mg, 0.1 mmol) according to **GP8** with (2-(1,3-dioxolan-2-yl)ethyl)zinc(II) bromide (**2a**, 0.2 mmol) at rt for 24 h. Purification by flash column chromatography on silica gel using cyclohexane/ethyl acetate = 30/1 afforded **11aa** as a yellow oil (24.3 mg, 80% yield).

R$_f$ = 0.33 (cyclohexane/ethyl acetate = 20/1). **^{1}H NMR** (500 MHz, CDCl$_3$): δ 0.02 (s, 6H), 0.98 (d, *J* = 6.7 Hz, 3H), 1.39–1.44 (m, 2H), 1.60–1.65 (m, 2H), 2.11 (s, 2H), 2.13–2.18 (m, 1H), 3.84–3.87 (m, 2H), 3.96-3.99 (m, 2H), 4.84 (t, *J* = 4.9 Hz, 1H), 5.55 (dd, *J* = 18.7, 1.1 Hz, 1H), 5.85 (dd, *J* = 18.7, 6.9 Hz, 1H), 6.98–7.00 (m, 2H), 7.04–7.07 (m, 1H), 7.18–7.21 (m, 2H) ppm. **^{13}C NMR** (126 MHz, CDCl$_3$): δ –3.9, 19.9, 26.3, 30.4, 31.7, 40.2, 64.8, 104.7, 123.8, 125.9, 128.0, 128.2, 140.2, 153.5 ppm. **^{29}Si DEPT NMR** (99 MHz, CDCl$_3$): δ –7.24 ppm. **IR (ATR)**: ṽ/cm^{-1} = 3023, 2953, 2876, 1492, 1248, 1144, 1045, 831, 698. **HRMS (APCI)** for $C_{18}H_{29}O_2Si^+$ [M+H]$^+$: calculated 305.1937, found 305.1937.

Optical rotation: $[\alpha]_D^{20}$ = –9.6 (c 1.00, CHCl$_3$, e.r. 92:8). The enantiomeric ratio of **11aa** was determined by HPLC analysis on a chiral stationary phase (*Daicel* Chiralcel OD-H column, column temperature 20 °C, solvent *n*-heptane:isopropanol = 99.9:0.1, flow rate 0.4 mL/min): t$_R$ = 55.0 min (minor), t$_R$ = 57.4 min (major).

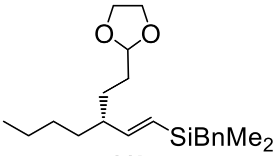

11ba

$C_{21}H_{34}O_2Si$
M = 346.23 g/mol

(–)-(3*R*,1*E*)-(3-(2-(1,3-Dioxolan-2-yl)ethyl)hept-1-en-1-yl) (benzyl)dimethylsilane (**11ba**): Prepared from (*E*)-benzyl(1-bromohept-2-en-1-yl)dimethylsilane and (*E*)-benzyl(3-bromohept-1-en-1-yl)dimethylsilane (**7b**, 165.2 mg, 0.6 mmol) according to **GP8** with (2-(1,3-dioxolan-2-yl)ethyl)zinc(II) bromide (**2a**, 1.2 mmol) at rt for 24 h. Purification by flash column chromatography on silica gel using cyclohexane/ethyl acetate = 30/1 afforded **11ba** as a yellow oil (175.4 mg, 84% yield).

R$_f$ = 0.30 (cyclohexane/ethyl acetate = 20/1). **^{1}H NMR** (500 MHz, CDCl$_3$): δ 0.02 (s, 6H), 0.88 (t, *J* = 7.7 Hz, 3H), 1.17–1.37 (m, 7H), 1.47–1.66 (m, 3H), 1.94–1.99 (m, 1H), 2.11 (s, 2H), 3.83–3.86 (m, 2H), 3.93–3.98 (m, 2H), 4.83 (t, *J* = 4.8 Hz, 1H), 5.52 (dd, *J* = 18.6, 0.99 Hz, 1H), 5.69 (dd, *J* = 18.6, 8.2 Hz, 1H), 6.98–7.00 (m, 2H), 7.04–7.07 (m, 1H), 7.17–7.21 (m, 2H) ppm. **^{13}C NMR** (126 MHz, CDCl$_3$): δ –3.2, –3.1, 14.1, 22.8, 26.4, 28.9, 29.4, 31.8, 34.6, 46.8, 64.9, 64.9, 104.8, 123.9, 127.9, 128.1. 128.3, 140.2, 152.42 ppm. **^{29}Si DEPT NMR** (99 MHz, CDCl$_3$): δ –7.46 ppm. **IR (ATR)**: ṽ/cm^{-1} = 2954, 2926, 1613, 1492, 1248, 1144, 1054, 833, 698. **HRMS (APCI)** for $C_{21}H_{35}O_2Si^+$ [M+H]$^+$: calculated 347.2406, found 347.2394.

Optical rotation: $[\alpha]_D^{20}$ = –0.6 (c 1.00, CHCl$_3$, e.r. 88:12). The enantiomeric ratio of **11ba** was determined by HPLC analysis on a chiral stationary phase (*Daicel* Chiralcel OD-H column, column temperature 20 °C, solvent *n*-heptane:isopropanol = 99.9:0.1, flow rate 0.4 mL/min): t$_R$ = 42.2 min (major), t$_R$ = 48.2 min (minor).

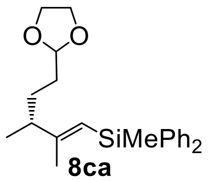

8ca

$C_{23}H_{30}O_2Si$
M = 366.20 g/mol

(–)-(3*R*,1*E*)-(5-(1,3-Dioxolan-2-yl)-2,3-dimethylpent-1-en-1-yl)(methyl)diphenylsilane (8ca): Prepared from (*E*)-(3-chloro-2-methylbut-1-en-1-yl)(methyl)diphenylsilane (**12c**, 30.1 mg, 0.1 mmol) according to **GP9** with (2-(1,3-dioxolan-2-yl)ethyl)zinc(II) bromide (**2a**, 0.2 mmol) at rt for 24 h. Purification by flash column chromatography on silica gel using cyclohexane/ethyl acetate = 30/1 afforded **8ca** as a yellowish oil (29.2 mg, 80% yield).

R_f = 0.35 (cyclohexane/ethyl acetate = 20/1). **^{1}H NMR** (500 MHz, $CDCl_3$): δ 0.63 (s, 3H), 1.07 (d, *J* = 6.8 Hz, 3H), 1.43–1.49 (m, 1H), 1.52–1.56 (m, 1H), 1.59 (d, *J* = 0.6 Hz, 3H), 1.62–1.67 (m, 2H), 2.24–2.31 (m, 1H), 3.84–3.86 (m, 2H), 3.95-3.98 (m, 2H), 4.86 (t, *J* = 4.7 Hz, 1H), 5.55 (s, 1H), 7.32–7.35 (m, 6H), 7.52–7.54 (m, 4H) ppm. **^{13}C NMR** (126 MHz, $CDCl_3$): δ –2.0, 19.2, 19.9, 29.3, 32.1. 45.7, 64.9, 104.7, 118.4, 127.8, 128.9, 134.6, 138.1, 163.0 ppm. **^{29}Si DEPT NMR** (99 MHz, $CDCl_3$): δ –19.03 ppm. **IR (ATR)**: $\tilde{\nu}$/cm^{-1} = 3066, 2955, 2874, 1608, 1427, 1250, 1110, 1042, 790, 731, 700. **HRMS (APCI)** for $C_{23}H_{31}O_2Si^+$ [M+H]$^+$: calculated 367.2093, found 367.2095.

Optical rotation: $[\alpha]_D^{20}$ = –7.6 (c 1.02, $CHCl_3$, e.r. 99:1). The enantiomeric ratio of **8ca** was determined by HPLC analysis on a chiral stationary phase (*Daicel* Chiralcel OD-H column, column temperature 20 °C, solvent *n*-heptane:isopropanol = 99.7:0.3, flow rate 0.4 mL/min): t_R = 22.4 min (minor), t_R = 25.6 min (major).

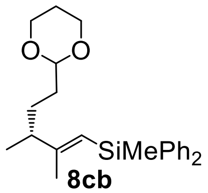

8cb

$C_{24}H_{32}O_2Si$
M = 380.22 g/mol

(–)-(3*R*,1*E*)-(5-(1,3-Dioxan-2-yl)-2,3-dimethylpent-1-en-1-yl)(methyl)diphenylsilane (**8cb**): Prepared from (*E*)-(3-chloro-2-methylbut-1-en-1-yl)(methyl)diphenylsilane (**12c**, 30.1 mg, 0.1 mmol) according to **GP9** with (2-(1,3-dioxan-2-yl)ethyl)zinc(II) bromide (**2b**, 0.2 mmol) at rt for 24 h. Purification by flash column chromatography on silica gel using cyclohexane/ethyl acetate = 30/1 afforded **8cb** as a yellow oil (25.2 mg, 66% yield).

R_f = 0.30 (cyclohexane/ethyl acetate = 20/1). **^{1}H NMR** (500 MHz, $CDCl_3$): δ 0.63 (s, 3H), 1.05 (d, *J* = 7.0 Hz, 3H), 1.31–1.36 (m, 1H), 1.39–1.46 (m, 1H), 1.50–1.54 (m, 1H), 1.56–1.61 (m, 2H), 1.59 (d, *J* = 0.7 Hz, 3H), 2.04–2.13 (m, 1H), 2.22–2.26 (m, 1H), 3.75 (td, *J* = 12.4, 2.3 Hz, 2H), 4.10 (ddd, *J* = 11.5, 4.9, 1.5 Hz, 2H), 4.52 (t, *J* = 5.5 Hz, 1H), 5.77 (s, 1H), 7.32–7.36 (m, 6H), 7.53–7.55 (m, 4H) ppm. **^{13}C NMR** (126 MHz, $CDCl_3$): δ –2.0, 19.2, 19.9, 25.8, 29.2, 33,4, 45.8, 66.9, 102.4, 118.2, 127.7, 128.8, 134.6, 138.1, 163.2 ppm. **^{29}Si DEPT NMR** (99 MHz, $CDCl_3$): δ –19.05 ppm. **IR (ATR)**: $\tilde{\nu}$/cm^{-1} = 3067, 2957, 2850, 1609, 1427, 1144, 1110, 1000, 791, 732, 700. **HRMS (APCI)** for $C_{24}H_{33}O_2Si^+$ [M+H]$^+$: calculated 381.2250, found 381.2253.

Optical rotation: $[\alpha]_D^{20}$ = –2.5 (c 0.91, $CHCl_3$, e.r. 97:3). The enantiomeric ratio of **8cb** was determined by HPLC analysis on a chiral stationary phase (*Daicel* Chiralcel OD-H column, column temperature 20 °C, solvent *n*-heptane:isopropanol = 99.7:0.3, flow rate 0.4 mL/min): t_R = 20.2 min (minor), t_R = 24.2 min (major).

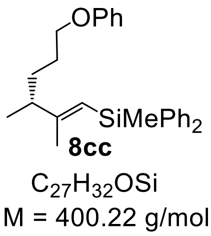

8cc

$C_{27}H_{32}OSi$
M = 400.22 g/mol

(–)-(3*R*,1*E*)-(2,3-Dimethyl-6-phenoxyhex-1-en-1-yl)(methyl) diphenylsilane (8cc): Prepared from (*E*)-(3-chloro-2-methylbut-1-en-1-yl)(methyl)diphenylsilane (**12c**, 30.1 mg, 0.1 mmol) according to **GP9** with (3-phenoxypropyl)zinc(II) bromide (**2c**, 0.2 mmol) at rt for 24 h. Purification by flash column chromatography on silica gel using cyclohexane/diethyl ether = 150/1 afforded **8cc** as a yellowish oil (26.1 mg, 65% yield).

R_f = 0.32 (cyclohexane/diethyl ether = 50/1. **^{1}H NMR** (500 MHz, $CDCl_3$): δ 0.64 (s, 3H), 1.09 (d, *J* = 6.8 Hz, 3H), 1.48–1.57 (m, 2H), 1.61 (d, *J* = 0.6 Hz, 3H), 1.74–1.56 (m, 2H), 2.29–2.36 (m, 1H), 3.96 (t, *J* = 6.3 Hz, 2H), 5.58 (s, 1H), 6.88–6.94 (m, 3H), 7.26–7.29 (m, 2H), 7.33–7.36 (m, 6H), 7.53–7.56 (m, 4H) ppm. **^{13}C NMR** (126 MHz, $CDCl_3$): δ –2.0, 19.2, 20.0, 27.5, 31.6, 45.7, 67.9, 114.5, 118.5, 120.5, 127.8, 128.9, 129.4, 134.6, 138.1, 159.1, 163.1 ppm. **^{29}Si DEPT NMR** (99 MHz, $CDCl_3$): δ –19.02 ppm. **IR (ATR)**: $\tilde{v}$/cm^{-1} = 3067, 2956, 2869, 1600, 1495, 1427, 1245, 1108, 1032, 791, 734, 697. **HRMS (APCI)** for $C_{27}H_{33}OSi^+$ [M+H]$^+$: calculated 401.2301, found 401.2284.

Optical rotation: $[\alpha]_D^{20}$ = –2.9 (c 0.60, $CHCl_3$, e.r. 91:9). The enantiomeric ratio of **8cc** was determined by HPLC analysis on a chiral stationary phase (*Daicel* Chiralcel OD-H column, column temperature 20 °C, solvent *n*-heptane:isopropanol = 99.5:0.5, flow rate 0.6 mL/min): t_R = 17.7 min (minor), t_R = 40.7 min (major).

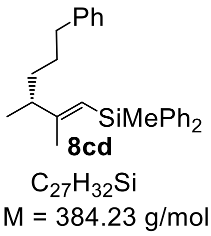

8cd

$C_{27}H_{32}Si$
M = 384.23 g/mol

(–)-(3*R*,1*E*)-(2,3-Dimethyl-6-phenylhex-1-en-1-yl)(methyl) diphenylsilane (8cd): Prepared from (*E*)-(3-chloro-2-methylbut-1-en-1-yl)(methyl)diphenylsilane (**12c**, 30.1 mg, 0.1 mmol) according to **GP9** with (3-phenylpropyl)zinc(II) bromide (**2d**, 0.2 mmol) at rt for 24 h. Purification by flash column chromatography on silica gel using cyclohexane afforded **8cd** as a yellowish oil (33.2 mg, 86% yield).

R_f = 0.69 (cyclohexane/ethyl acetate = 20/1). **^{1}H NMR** (500 MHz, $CDCl_3$): δ 0.64 (s, 3H), 1.05 (d, *J* = 6. Hz, 3H), 1.35–1.40 (m, 1H), 1.45–1.49 (m, 1H), 1.56 (s, 3H), 1.58–1.66 (m, 2H), 2.27–2.34 (m, 1H), 2.56–2.67 (m, 2H), 5.55 (s, 1H), 7.17–7.20 (m, 3H), 7.26–7.29 (m, 2H), 7.32–7.36 (m, 6H), 7.53–7.55 (m, 4H) ppm. **^{13}C NMR** (126 MHz, $CDCl_3$): δ –1.9, 19.1, 20.0, 29.5, 34.8, 36.0, 45.8, 118.2, 125.6, 127.7, 128.2, 128.4, 128.9, 134.5, 138.1, 142.7, 163.4 ppm. **^{29}Si DEPT NMR** (99 MHz, $CDCl_3$): δ –19.04 ppm. **IR (ATR)**: $\tilde{v}$/cm^{-1} = 3065, 2928, 2854, 1607, 1453, 1249, 1109, 791, 732, 698. **HRMS (LIFDI)** for $C_{27}H_{33}O_2Si^+$ [M+H]$^+$: calculated 385.2352, found 385.2364.

Optical rotation: $[\alpha]_D^{20}$ = –11.7 (c 0.97, $CHCl_3$, e.r. 99:1). The enantiomeric ratio of **8cd** was determined by HPLC analysis on a chiral stationary phase (*Daicel* Chiralcel IB column, column temperature 20 °C, solvent *n*-heptane:isopropanol = 100:0, flow rate 0.2 mL/min): t_R = 29.8 min (minor), t_R = 32.2 min (major).

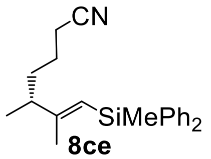

8ce

$C_{22}H_{27}NSi$

M = 333.19 g/mol

(–)-(3*R*,1*E*)-5,6-Dimethyl-7-(methyldiphenylsilyl)hept-6-enenitrile (8ce): Prepared from (*E*)-(3-chloro-2-methylbut-1-en-1-yl)(methyl)diphenylsilane (**12c**, 30.1 mg, 0.1 mmol) according to **GP9** with (3-cyanopropyl)zinc(II) bromide (**2e**, 0.2 mmol) at rt for 24 h. Purification by flash column chromatography on silica gel using cyclohexane/ethyl acetate = 30/1 afforded **8ce** as a yellowish oil (27.6 mg, 83% yield).

R_f = 0.19 (cyclohexane/ethyl acetate = 20/1). **^{1}H NMR** (500 MHz, $CDCl_3$): δ 0.64 (s, 3H), 1.08 (d, *J* = 6.8 Hz, 3H), 1.46–1.50 (m, 1H), 1.53–1.57 (m, 1H), 1.59 (d, *J* = 0.8 Hz, 3H), 1.59–1.66 (m, 2H), 2.27–2.31 (m, 1H), 2.34 (t, *J* = 7.0 Hz, 2H), 5.57 (s, 1H), 7.34–7.37 (m, 6H), 7.52–7.54 (m, 4H) ppm. **^{13}C NMR** (126 MHz, $CDCl_3$): δ –2.1, 17.2, 18.9, 19.9, 23.6, 34.0, 45.3, 119.4, 119.7, 127.8, 129.0, 134.6, 137.8, 162.0 ppm. **^{29}Si DEPT NMR** (99 MHz, $CDCl_3$): δ –19.02 ppm. **IR (ATR)**: ṽ/cm^{-1} = 3067, 2958, 2870, 2245, 1609, 1427, 1109, 731, 734, 700. **HRMS (LIFDI)** for $C_{22}H_{28}NSi^+$ $[M+H]^+$: calculated 334.1991, found 334.1988.

Optical rotation: $[\alpha]_D^{20}$ = –7.2 (c 1.00, $CHCl_3$, e.r. 97:3). The enantiomeric ratio of **8ce** was determined by HPLC analysis on a chiral stationary phase (*Daicel* Chiralcel AS-H column, column temperature 20 °C, solvent *n*-heptane:isopropanol = 99:1, flow rate 0.4 mL/min): t_R = 22.2 min (minor), t_R = 28.7 min (major).

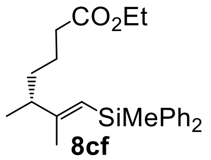

8cf

$C_{24}H_{32}O_2Si$

M = 380.22 g/mol

(–)-Ethyl (3*R*,1*E*)-5,6-dimethyl-7-(methyldiphenylsilyl)hept-6-enoate (8cf): Prepared from (*E*)-(3-chloro-2-methylbut-1-en-1-yl)(methyl)diphenylsilane (**12c**, 30.1 mg, 0.1 mmol) according to **GP9** with (4-ethoxy-4-oxobutyl)zinc(II) bromide (**2f**, 0.2 mmol) at rt for 24 h. Purification by flash column chromatography on silica gel using cyclohexane/ethyl acetate = 40/1 afforded **8cf** as a yellowish oil (32.0 mg, 84% yield).

R_f = 0.48 or 0.27 (cyclohexane/ethyl acetate = 20/1). **^{1}H NMR** (500 MHz, $CDCl_3$): δ 0.56 (s, 3H), 0.98 (d, *J* = 6.9 Hz, 3H), 1.18 (t, *J* = 6.9 Hz, 3H), 1.24–1.30 (m, 1H), 1.34–1.42 (m, 1H), 1.51 (d, *J* = 0.5 Hz, 3H), 1.52–1.57 (m, 2H), 2.18–2.24 (m, 3H), 4.06 (q, *J* = 7.3, 7.1 Hz, 2H), 5.48 (s, 1H), 7.25–7.28 (m, 6H), 7.45–7.48 (m, 4H) ppm. **^{13}C NMR** (126 MHz, $CDCl_3$): δ –2.0, 14.3, 19.1, 19.9, 23.1, 34.4, 35.6, 45.7, 60.2, 118.4, 127.8, 128.9, 134.6, 138.1, 163.0, 173.7 ppm. **^{29}Si DEPT NMR** (99 MHz, $CDCl_3$): δ –19.02 ppm. **IR (ATR)**: ṽ/cm^{-1} = 3067, 2958, 1734, 1609, 1427, 1249, 1109, 791, 732, 700. **HRMS (APCI)** for $C_{24}H_{33}O_2Si^+$ $[M+H]^+$: calculated 381.2250, found 381.2245.

Optical rotation: $[\alpha]_D^{20}$ = –7.8 (c 0.98, $CHCl_3$, e.r. 98:2). The enantiomeric ratio of **8cf** was determined by HPLC analysis on a chiral stationary phase (*Daicel* Chiralcel OD-H column, column temperature 20 °C, solvent *n*-heptane:isopropanol = 99.9:0.1, flow rate 0.4 mL/min): t_R = 40.0 min (major), t_R = 44.8 min (minor).

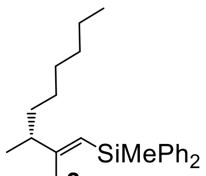

8cg

$C_{24}H_{34}Si$

M =350.64 g/mol

(–)-(3*R*,1*E*)-(2,3-Dimethylnon-1-en-1-yl)(methyl)diphenylsilane (8cg): Prepared from (*E*)-(3-chloro-2-methylbut-1-en-1-yl)(methyl)diphenylsilane (**12c**, 30.1 mg, 0.1 mmol) according to **GP9** with (1-hexyl)zinc(II) bromide (**2g**, 0.2 mmol) at rt for 24 h. Purification by flash column chromatography on silica gel using cyclohexane afforded **8cg** as a yellow oil (28.5 mg, 81% yield).

R_f = 0.50 (cyclohexane). **^{1}H NMR** (500 MHz, $CDCl_3$): δ 0.63 (s, 3H), 0.89 (t, *J* = 7.1 Hz, 3H), 1.04 (d, *J* = 7.1 Hz, 3H), 1.23–1.33 (m, 10H), 1.58 (d, *J* = 0.7 Hz, 3H), 2.23–2.27 (m, 1H), 5.53 (s, 1H), 7.26–7.35 (m, 6H), 7.53–7.56 (m, 4H) ppm. **^{13}C NMR** (126 MHz, $CDCl_3$): δ –1.9, 14.1, 19.2, 20.0, 22.6, 27.6, 29.4, 31.9, 35.3, 45.9, 117.7, 127.7, 128.8, 134.5, 138.3, 163.8 ppm. **^{29}Si DEPT NMR** (99 MHz, $CDCl_3$): δ 12.04 ppm. **IR (ATR)**: $\tilde{\nu}$/cm^{-1} = 3067, 2924, 2853, 1608, 1427, 1258, 1108, 791, 730, 698. **HRMS (APCI)** for $C_{24}H_{35}Si^+$ [M+H]$^+$: calculated 351.2508, found 351.2512.

The spectroscopic data are in accordance with those reported in the literature.[S13]

Optical rotation: $[\alpha]_D^{20}$ = –5.2 (c 1.00, $CHCl_3$, e.r. 96:4). The enantiomeric ratio of **8cg** was determined by reversed phase HPLC analysis on a chiral stationary phase (*Daicel* Chiralcel OJ-RH column, column temperature 20 °C, solvent acetonitrile:water = 65:35, flow rate 0.3 mL/min): t_R = 84.4 min (major), t_R = 107.4 min (minor).

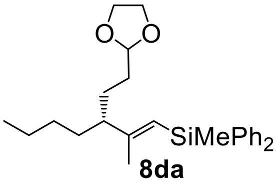

8da

$C_{26}H_{36}O_2Si$

M = 408.25 g/mol

(+)-(3*R*,1*E*)-(3-(2-(1,3-Dioxolan-2-yl)ethyl)-2-methylhept-1-en-1-yl)(methyl)diphenylsilane (8da): Prepared from (*E*)-(3-chloro-2-methylhept-1-en-1-yl)(methyl)diphenylsilane (**12d**, 30.1 mg, 0.1 mmol) according to **GP9** with (2-(1,3-dioxolan-2-yl)ethyl)zinc(II) bromide (**2a**, 0.2 mmol) at rt for 24 h. Purification by flash column chromatography on silica gel using cyclohexane/ethyl acetate = 30/1 afforded **8da** as a yellowish oil (36.7 mg, 90% yield).

R_f = 0.30 (cyclohexane/ethyl acetate = 20/1). **^{1}H NMR** (500 MHz, $CDCl_3$): δ 0.63 (s, 3H), 0.89 (t, *J* = 6.8 Hz, 3H), 1.22–1.39 (m, 7H), 1.42–1.50 (m, 1H), 1.52 (d, *J* = 0.7 Hz, 3H), 1.60–1.65 (m, 2H), 2.11–2.17 (m, 1H), 3.83–3.86 (m, 2H), 3.95-3.98 (m, 2H), 4.86 (t, *J* = 4.8 Hz, 1H), 5.56 (s, 1H), 7.32–7.36 (m, 6H), 7.53–7.56 (m, 4H) ppm. **^{13}C NMR** (126 MHz, $CDCl_3$): δ –2.0, 14.1, 18.2, 22.7, 27.9, 29.8, 32.1, 33.4, 53.3, 64.8, 64.8, 104.6, 120.9, 127.7, 128.9, 134.6, 134.6, 138.1, 138.1, 161.0 ppm. **^{29}Si DEPT NMR** (99 MHz, $CDCl_3$): δ –19.21 ppm. **IR (ATR)**: $\tilde{\nu}$/cm^{-1} = 3066, 2925, 2870, 1608, 1427, 1250, 1109, 791, 732, 699. **HRMS (APCI)** for $C_{26}H_{37}O_2Si^+$ [M+H]$^+$: calculated 409.2563, found 409.2548.

Optical rotation: $[\alpha]_D^{20}$ = +2.3 (c 1.00, $CHCl_3$, e.r. 97:3). The enantiomeric ratio of **8da** was determined by HPLC analysis on a chiral stationary phase (*Daicel* Chiralcel OD-H column, column temperature 20 °C, solvent *n*-heptane:isopropanol = 99.9:0.1, flow rate 0.2 mL/min): t_R = 67.4 min (minor), t_R = 69.9 min (major).

6. Experimental Details for the Copper-Mediated Cross-Coupling

6.1. General Procedure for the Copper-Mediated Cross-Coupling of Alkenylbenzyldimethylsilanes (GP10)[S14]

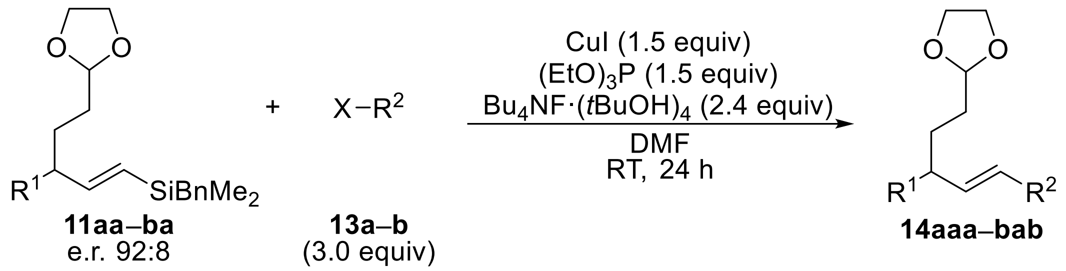

Scheme S10. Preparation of chiral alkyl-substituted acyclic allylic systems.

A base-washed, flame-dried Schlenk tube equipped with a magnetic stir bar was charged with CuI (28.6 mg, 0.15 equiv, 1.5 equiv) and $Bu_4NF\cdot(t\text{BuOH})_4$ (133.9 mg, 0.24 mmol, 2.4 equiv) under N_2. A solution of $(EtO)_3P$ (25.7 µL, 0.15 mmol, 1.5 equiv) in DMF (0.3 mL) and a solution of vinylsilane **11** (84 mg, 0.1 mmol, 1.0 equiv) and halide **13** (0.3 mmol, 3.0 equiv) in DMF (0.7 mL) were successively added to the flask at rt under N_2. The mixture was stirred at room temperature for 24 h. After completion of the reaction, the reaction mixture was directly transferred to a column of silica gel without additional work-up.

6.2. Characterization Data of the Chiral Alkyl-Substituted Alkenes

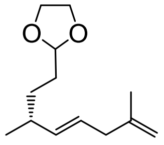

14aaa
$C_{13}H_{22}O_2$
M = 210.16 g/mol

(–)-(2*R*,4*E*)-2-(3,7-Dimethylocta-4,7-dien-1-yl)-1,3-dioxolane (**14aaa**): Prepared from (3*R*,1*E*)-(5-(1,3-dioxolan-2-yl)-3-methylpent-1-en-1-yl)(benzyl)dimethylsilane (**11aa**, 30.4 mg, 0.1 mmol) according to **GP10** with 3-chloro-2-methyl-propene (**13a**, 29.4 µL, 0.3 mmol) at rt for 24 h. Purification by flash column chromatography on silica gel using n-pentane/diethyl ether = 10/1 afforded **14aaa** as a yellow oil (16.6 mg, 79% yield).

R_f = 0.29 (cyclohexane/ethyl acetate = 20/1). **^{1}H NMR** (400 MHz, $CDCl_3$): δ 0.99 (dd, *J* = 6.8, 0.6 Hz, 3H), 1.35–1.45 (m, 2H), 1.60–1.68 (m, 2H), 1.70 (s, 3H), 2.09–2.15 (m, 1H), 2.66 (d, *J* = 6.5 Hz, 2H), 3.82–3.85 (m, 2H), 3.94-3.97 (m, 2H), 4.69 (d, *J* = 8.2 Hz, 2H), 4.83 (t, *J* = 4.6 Hz, 1H), 5.27–5.41 (m, 2H) ppm. **^{13}C NMR** (101 MHz, $CDCl_3$): δ 20.8, 22.4, 31.1, 31.8, 36.7, 41.1, 64.8, 104.7, 110.2, 126.4, 137.6, 145.2 ppm. **IR (ATR)**: ṽ/cm^{-1} = 2953, 2924, 2872, 1648, 1453, 1136, 1037, 969, 886. **HRMS (APCI)** for $C_{13}H_{23}O_2^+$ [M+H]$^+$: calculated 211.1698, found 211.1693.

Optical rotation: $[\alpha]_D^{20}$ = –2.8 (c 0.97, $CHCl_3$, e.r. 92:8). The enantiomeric ratio of **14aaa** was determined by HPLC analysis on a chiral stationary phase (*Daicel* Chiralcel OD-H column, column temperature 20 °C, solvent *n*-heptane:isopropanol = 99.9:0.1, flow rate 0.4 mL/min): t_R = 28.8 min (minor), t_R = 33.8 min (major).

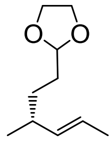

14aab
$C_{10}H_{18}O_2$
M = 170.13 g/mol

(–)-(2*R*,4*E*)-2-(3-Methylhex-4-en-1-yl)-1,3-dioxolane (**14aab**): Prepared from (3*R*,1*E*)-(5-(1,3-dioxolan-2-yl)-3-methylpent-1-en-1-yl)(benzyl)dimethylsilane (**11aa**, 30.4 mg, 0.1 mmol) according to **GP10** with iodomethane (**13b**, 18.7 µL, 0.3 mmol) at rt for 24 h. Purification by flash column chromatography on silica gel using *n*-pentane/diethyl ether = 20/1 afforded **14aab** as a yellow oil (12.2 mg, 72% yield).

R_f = 0.25 (cyclohexane/ethyl acetate = 20/1). **^{1}H NMR** (400 MHz, $CDCl_3$): δ 0.96 (d, *J* = 6.6 Hz, 3H), 1.32–1.42 (m, 2H), 1.56–1.68 (m, 5H), 2.03–2.09 (m, 1H), 3.81–3.86 (m, 2H), 3.91-3.96 (m, 2H), 4.82 (t, *J* = 5.0 Hz, 1H), 5.25 (ddq, *J* = 15.4, 7.8, 1.5 Hz, 1H), 5.37 (ddq, *J* = 15.3, 6.5, 0.7 Hz, 1H) ppm. **^{13}C NMR** (101 MHz, $CDCl_3$): δ 18.1, 21.0, 31.3, 31.9, 36.8, 65.0, 104.9, 123.6, 137.0 ppm. **IR (ATR)**: ṽ/cm^{-1} = 2954, 2926, 2873, 1602, 1456, 1206, 1152, 1042, 967, 820. **HRMS (APCI)** for $C_{10}H_{19}O_2^+$ [M+H]$^+$: calculated 171.1385, found 171.1379.

The spectroscopic data are in accordance with those reported in the literature.[S15]

Optical rotation: $[\alpha]_D^{20}$ = –6.9 (c 0.89, $CHCl_3$, e.r. 92:8). The enantiomeric ratio of **14aab** was determined by chiral GLC analysis (*Varian* CP-Chirasil-Dex CB column (25 m × 0.25 mm, 0.25 µm film thickness), column temperature 60 °C (isothermal), flow rate 1.0 mL/min): t_R = 132.5 min (minor), t_R = 135.4 min (major).

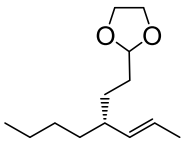

14bab
$C_{13}H_{24}O_2$
M = 212.18 g/mol

(–)-(2*R*,4*E*)-2-(3-(Prop-1-en-1-yl)heptyl)-1,3-dioxolane (**14bab**): Prepared from (*R*)-(3R,1E)-(3-(2-(1,3-dioxolan-2-yl)ethyl)hept-1-en-1-yl)(benzyl)dimethylsilane (**11ba**, 34.6 mg, 0.1 mmol) according to **GP10** with iodomethane (**13b**, 18.7 µL, 0.3 mmol) at rt for 24 h. Purification by flash column chromatography on silica gel using *n*-pentane/diethyl ether = 20/1 afforded **14aac** as a yellow oil (13.6 mg, 64% yield).

R_f = 0.25 (cyclohexane/ethyl acetate = 20/1). **^{1}H NMR** (400 MHz, $CDCl_3$): δ 0.87 (t, *J* = 6.7 Hz, 3H), 1.19–1.31 (m, 6H), 1.44–1.60 (m, 3H), 1.64 (dd, *J* = 6.4, 1.6 Hz, 3H), 1.66–1.72 (m, 1H), 1.85–1.90 (m, 1H), 3.82–3.87 (m, 2H), 3.92-3.97 (m, 2H), 4.82 (t, *J* = 4.8 Hz, 1H), 5.11 (ddq, *J* = 15.4, 8.9, 1.6 Hz, 1H), 5.35 (ddq, *J* = 15.4, 6.5, 0.7 Hz, 1H) ppm. **^{13}C NMR** (101 MHz, $CDCl_3$): δ 14.1, 17.9, 22.8, 29.4, 29.6, 31.8, 35.2, 42.7, 64.8, 64.8, 104.8, 124.9, 135.6 ppm. **IR (ATR)**: $\tilde{\nu}$/cm^{-1} = 2925, 2871, 1454, 1248, 1140, 1039, 966, 852. **HRMS (APCI)** for $C_{13}H_{25}O_2^+$ $[M+H]^+$: calculated 213.1855, found 213.1849.

Optical rotation: $[\alpha]_D^{20}$ = –0.2 (c 1.00, $CHCl_3$, e.r. 88:12). The enantiomeric ratio of **14bab** was determined by chiral GLC analysis (*Sigma-Aldrich* Astec Chiraldex B-DM column (30 m × 0.25 mm, 0.12 µm film thickness), column temperature 80 °C (200 min), ramp of 2 °C/ min to 200 °C (10 min), flow rate 1.0 mL/min): t_R = 220.7 min (major), t_R = 221.5 min (minor).

7. Scale-up Experiment (1.3 mmol)

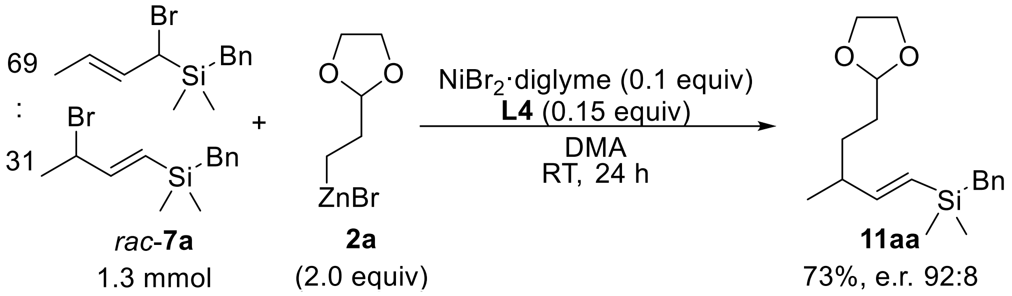

Scheme S11. Scale-up reaction.

A flame-dried Schlenk tube equipped with a magnetic stir bar was charged with the 69:31 mixture of (2*E*)-benzyl(1-bromobut-2-en-1-yl)dimethylsilane and (1*E*)-benzyl(1-bromobut-2-en-1-yl)dimethylsilane (**7a**, 1.3 mmol, 1.0 equiv), followed by $NiBr_2$·diglyme (45.8 mg, 0.13 mmol, 10.0 mol%) and (*S*,*S*)-*s*Bu-Pybox (**L4**, 64.2 mg, 0.195 mmol, 15.0 mol%). The reaction tube was capped with a septum and wrapped with teflon tape. Then, the tube was placed under high vacuum on a Schlenk line for 20 min and backfilled with N_2 (3 times), followed by the addition of anhydrous DMA (13.0 mL). After stirring for 30 min at room temperature, the reaction mixture appeared orange. Next, (2-(1,3-dioxolan-2-yl)ethyl)zinc(II) bromide (**2a**, 2.6 mmol, 2.0 equiv) was added dropwise, leading to a dark brown/black reaction mixture and stirred at ~800 rpm for 24 h. After completion of the reaction, it was diluted with water (5 mL). CH_2Cl_2 (10 mL) was added for extraction, and the resulting suspension was washed with brine (20 mL) and water (20 mL). The aqueous phase was extracted with CH_2Cl_2 (2 × 20 mL). The combined organic phases were dried over anhydrous $MgSO_4$, filtered, and concentrated under reduced pressure. Purification by flash column chromatography on silica gel using cyclohexane/ethyl acetate = 30/1 afforded **11aa** as a yellowish oil (289.0 mg, 73% yield).

8. Assignment of Absolute Configuration

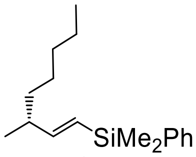

3ah

The found optical rotation of **3ah** is $[\alpha]_D^{20}$ = –11.0 (c 1.11, $CHCl_3$, e.r. 76:24).

The reported optical rotation for (+)-(3*S*,1*E*)-dimethyl-(3-methyl-oct-1-enyl)-phenyl silane[S13] is $[\alpha]_D^{20}$ = +20.6 (c 1.37, $CHCl_3$, e.r. 94:6).

Therefore, the absolute configuration of **3ah** is *R*.

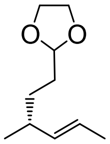

14aab

The found optical rotation of **14aab** is $[\alpha]_D^{20}$ = –6.9 (c 0.89, $CHCl_3$, e.r. 92:8).

The reported optical rotation for (+)-(2*S*,4*E*)-2-(3-methylhex-4-en-1-yl)-1,3-dioxolane[S15] is $[\alpha]_D^{20}$ = +12.6 (c 2.34, $CHCl_3$, e.r. 95:5).

Therefore, the absolute configuration of **14aab** is *R*.

9. HPLC Traces

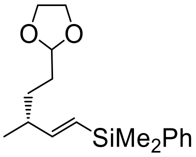

3aa

(3*R*,1*E*)-(5-(1,3-Dioxolan-2-yl)-3-methylpent-1-en-1-yl)dimethyl(phenyl)silane (**3aa**): The enantiomeric ratio of **3aa** was determined by HPLC analysis on a chiral stationary phase (*Daicel* Chiralcel OD-H column, column temperature 20 °C, solvent *n*-heptane:isopropanol = 99.9:0.1, flow rate 0.4 mL/min): t_R = 27.0 min (minor), t_R = 34.6 min (major).

Figure S1. *rac*-(1*E*)-(5-(1,3-Dioxolan-2-yl)-3-methylpent-1-en-1-yl)dimethyl(phenyl) silane (*rac*-**3aa**).

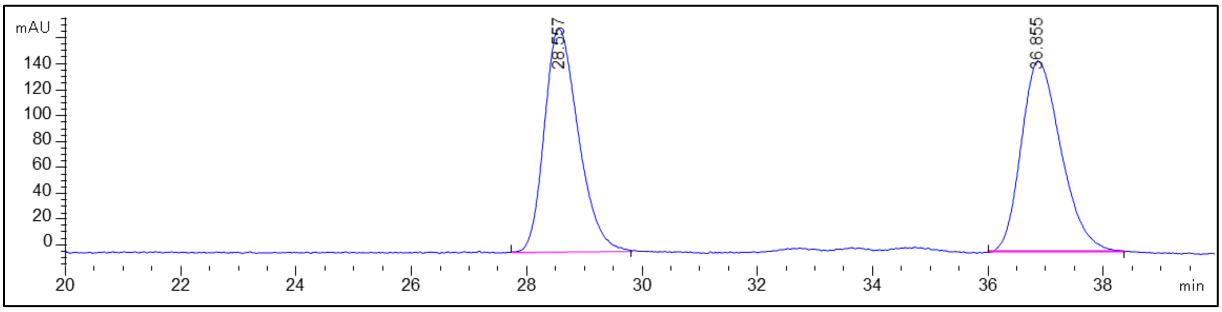

Peak #	RetTime [min]	Type	Width [min]	Area [mAU*s]	Height [mAU]	Area %
1	28.557	VV	0.5271	7102.66650	172.23831	49.8837
2	36.855	VB	0.6042	7135.78955	147.56187	50.1163

Figure S2. (3*R*,1*E*)-(5-(1,3-Dioxolan-2-yl)-3-methylpent-1-en-1-yl)dimethyl(phenyl) silane [(*R*)-**3aa**].

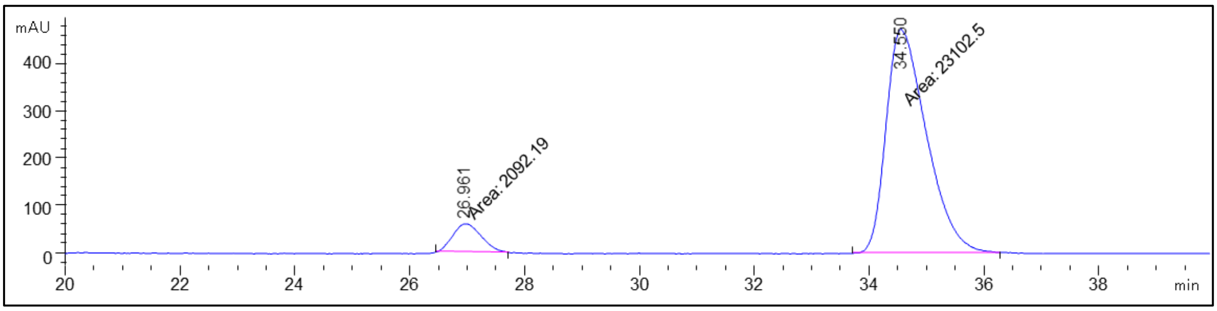

Peak #	RetTime [min]	Type	Width [min]	Area [mAU*s]	Height [mAU]	Area %
1	26.961	MM	0.5815	2092.18652	59.96505	8.3041
2	34.550	MM	0.8136	2.31025e4	473.24792	91.6959

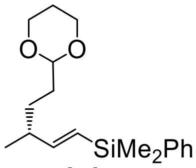

3ab

(3*R*,1*E*)-(5-(1,3-Dioxan-2-yl)-3-methylpent-1-en-1-yl)dimethyl(phenyl)silane (**3ab**): The enantiomeric ratio of **3ab** was determined by HPLC analysis on a chiral stationary phase (*Daicel* Chiralcel OD-H column, column temperature 20 °C, solvent *n*-heptane:isopropanol = 99.9:0.1, flow rate 0.4 mL/min): t_R = 25.4 min (minor), t_R = 38.8 min (major).

Figure S3. *rac*-(1*E*)-(5-(1,3-Dioxan-2-yl)-3-methylpent-1-en-1-yl)dimethyl(phenyl) silane (*rac*-**3ab**).

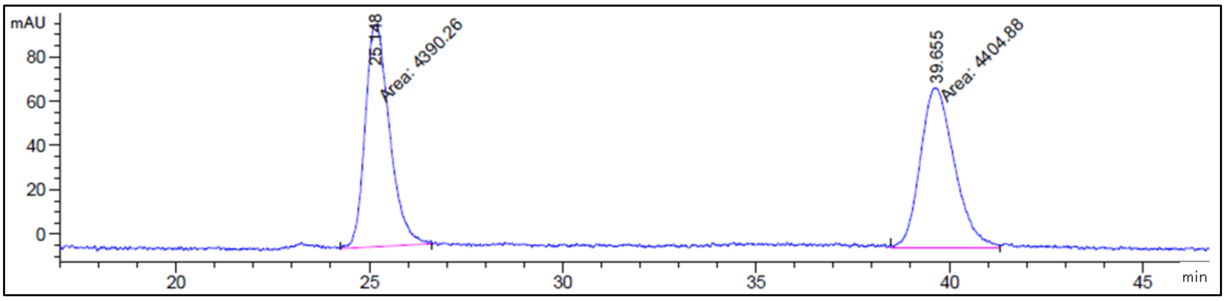

Peak #	RetTime [min]	Type	Width [min]	Area [mAU*s]	Height [mAU]	Area %
1	25.148	MM	0.7312	4390.25684	100.07010	49.9168
2	39.655	MM	1.0218	4404.88330	71.85127	50.0832

Figure S4. (3*R*,1*E*)-(5-(1,3-Dioxan-2-yl)-3-methylpent-1-en-1-yl)dimethyl(phenyl) silane [(*R*)-**3ab**].

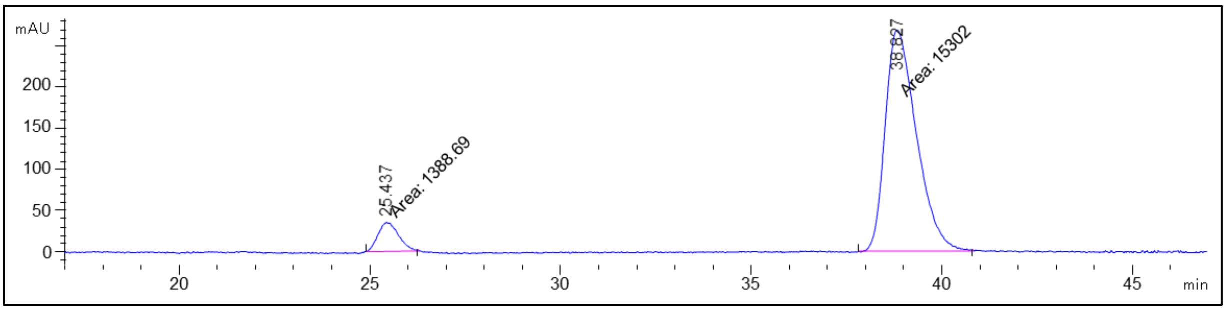

Peak #	RetTime [min]	Type	Width [min]	Area [mAU*s]	Height [mAU]	Area %
1	25.437	MM	0.6440	1388.69141	35.93978	8.3202
2	38.827	MM	0.9509	1.53020e4	268.21466	91.6798

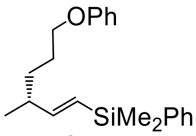

3ac

(3*R*,1*E*)-Dimethyl(3-methyl-6-phenoxyhex-1-en-1-yl)(phenyl)silane (**3ac**): The enantiomeric ratio of **3ac** was determined by HPLC analysis on a chiral stationary phase (*Daicel* Chiralcel OD-H column, column temperature 20 °C, solvent *n*-heptane:isopropanol = 99:1, flow rate 0.8 mL/min): t_R = 7.8 min (minor), t_R = 17.8 min (major).

Figure S5. *rac*-(1*E*)-Dimethyl(3-methyl-6-phenoxyhex-1-en-1-yl)(phenyl)silane (*rac*-**3ac**).

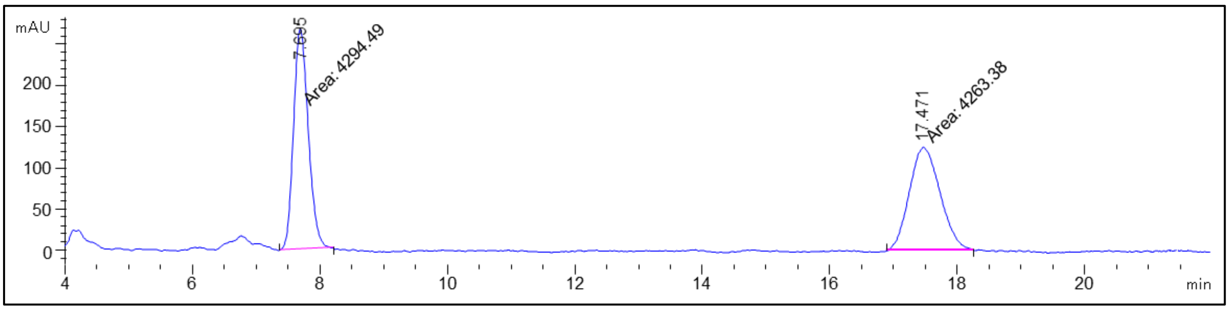

Peak #	RetTime [min]	Type	Width [min]	Area [mAU*s]	Height [mAU]	Area %
1	7.695	MM	0.2682	4294.49170	266.89380	50.1818
2	17.471	MM	0.5649	4263.38037	125.77938	49.8182

Figure S6. (3*R*,1*E*)-Dimethyl(3-methyl-6-phenoxyhex-1-en-1-yl)(phenyl)silane [(*R*)-**3ac**].

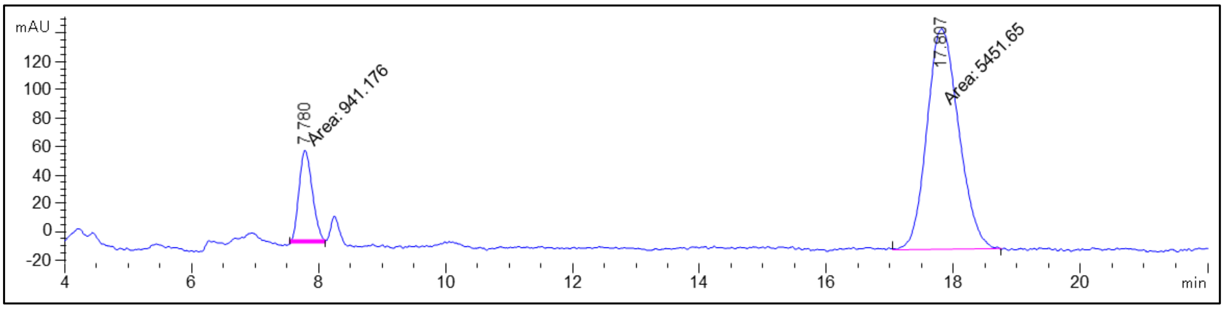

Peak #	RetTime [min]	Type	Width [min]	Area [mAU*s]	Height [mAU]	Area %
1	7.780	MM	0.2429	941.17603	64.58446	14.7224
2	17.807	MM	0.5835	5451.64746	155.70490	85.2776

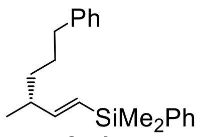

3ad

(3*R*,1*E*)-Dimethyl(3-methyl-6-phenylhex-1-en-1-yl)(phenyl)silane (**3ad**): The enantiomeric ratio of **3ad** was determined by HPLC analysis on a chiral stationary phase (*Daicel* Chiralcel OD-H column, column temperature 20 °C, solvent *n*-heptane:isopropanol = 90:10, flow rate 0.8 mL/min,): t_R = 4.3 min (major), t_R = 5.5 min (minor).

Figure S7. *rac*-(1*E*)-Dimethyl(3-methyl-6-phenylhex-1-en-1-yl)(phenyl)silane (*rac*-**3ad**).

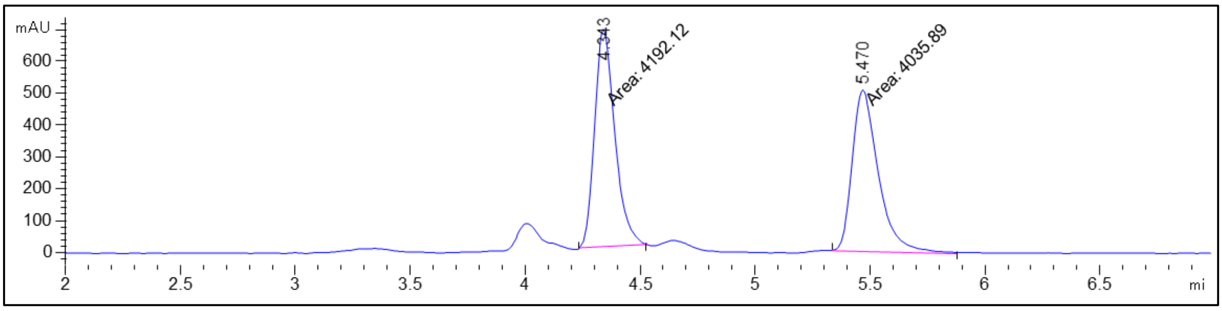

Peak #	RetTime [min]	Type	Width [min]	Area [mAU*s]	Height [mAU]	Area %
1	4.343	MM	0.1021	4192.11963	684.17590	50.9494
2	5.470	MM	0.1325	4035.89331	507.79199	49.0506

Figure S8. (3*R*,1*E*)-Dimethyl(3-methyl-6-phenylhex-1-en-1-yl)(phenyl)silane [(*R*)-**3ad**].

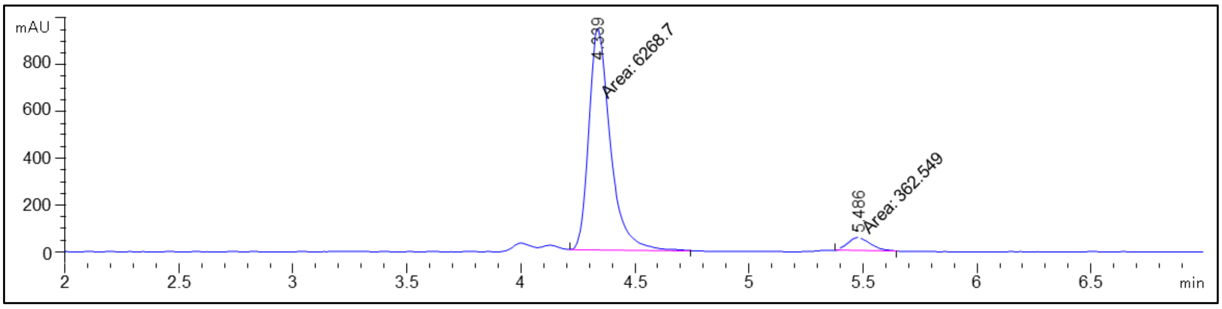

Peak #	RetTime [min]	Type	Width [min]	Area [mAU*s]	Height [mAU]	Area %
1	4.339	MM	0.1098	6268.70020	951.84302	94.5327
2	5.486	MM	0.1140	362.54858	52.99471	5.4673

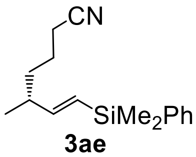

3ae

(3*R*,1*E*)-(6-Cyano-3-methylhex-1-en-1-yl)dimethyl(phenyl)silane (**3ae**): The enantiomeric ratio of **3ae** was determined by HPLC analysis on a chiral stationary phase (*Daicel* Chiralcel AS-H column, column temperature 20 °C, solvent *n*-heptane:isopropanol = 98:2, flow rate 0.4 mL/min): t_R = 13.5 min (miror), t_R = 14.2 min (major).

Figure S9. *rac*-(1*E*)-(6-Cyano-3-methylhex-1-en-1-yl)dimethyl(phenyl)silane (*rac*-**3ae**).

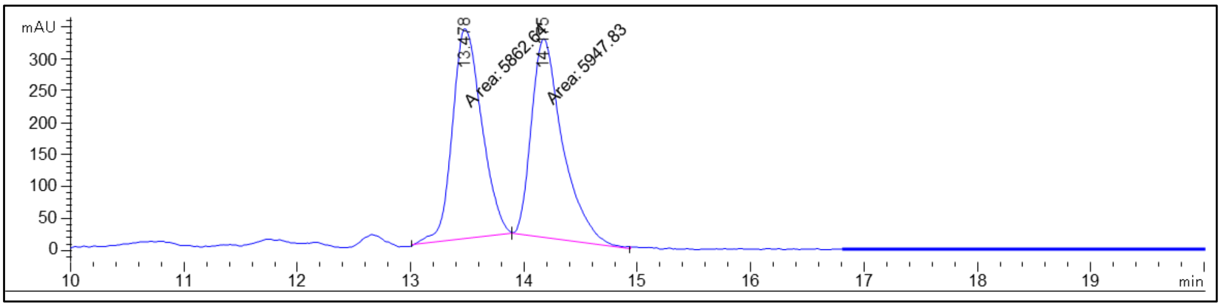

Peak #	RetTime [min]	Type	Width [min]	Area [mAU*s]	Height [mAU]	Area %
1	13.478	MM	0.2980	5862.64355	327.90231	49.6393
2	14.175	MM	0.3194	5947.83350	310.39978	50.3607

Figure S10. (3*R*,1*E*)-(6-Cyano-3-methylhex-1-en-1-yl)dimethyl(phenyl)silane [(*R*)-**3ae**].

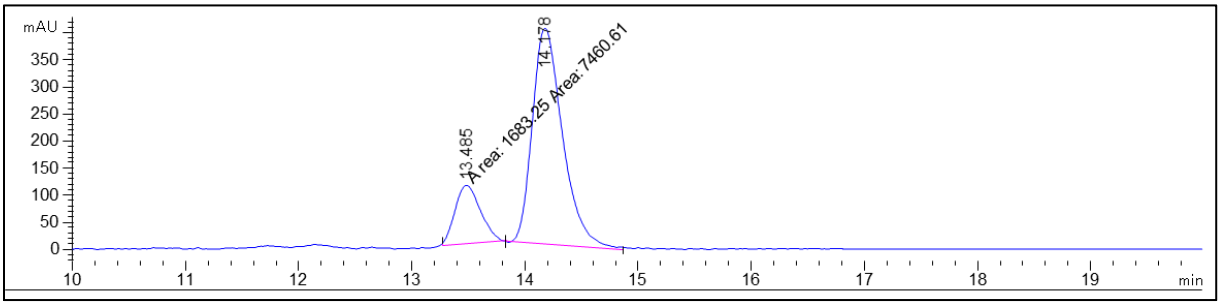

Peak #	RetTime [min]	Type	Width [min]	Area [mAU*s]	Height [mAU]	Area %
1	13.485	MM	0.2611	1683.25085	107.46630	18.4085
2	14.178	MM	0.3118	7460.61084	398.81110	81.5915

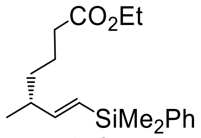

3af

(3*R*,1*E*)-(7-Ethoxy-7-oxo-3-methylhept-1-en-1-yl)dimethyl(phenyl)silane (**3af**): The enantiomeric ratio of **3af** was determined by HPLC analysis on a chiral stationary phase (*Daicel* Chiralcel OD-H column, column temperature 20 °C, solvent *n*-heptane:isopropanol = 99.9:0.1, flow rate 0.4 mL/min): t_R = 27.0 min (major), t_R = 29.1 min (minor).

Figure S11. *rac*-(1*E*)-(7-Ethoxy-7-oxo-3-methylhept-1-en-1-yl)dimethyl(phenyl)silane (*rac*-**3af**).

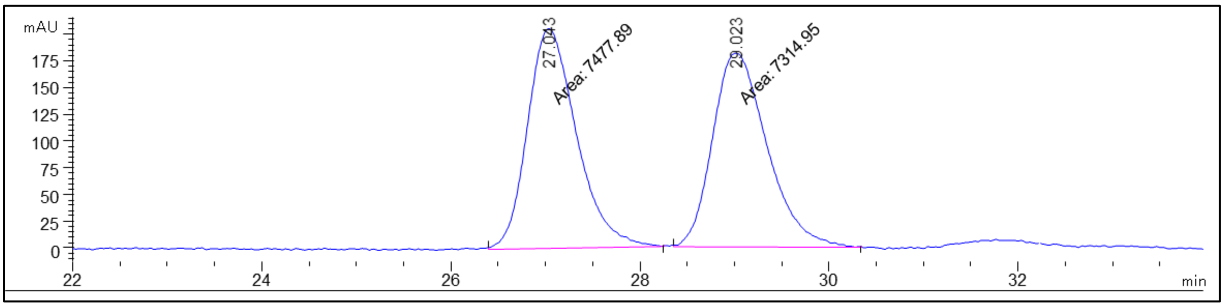

Peak #	RetTime [min]	Type	Width [min]	Area [mAU*s]	Height [mAU]	Area %
1	27.043	MM	0.6057	7477.88916	205.75089	50.5507
2	29.023	MM	0.6718	7314.94775	181.48193	49.4493

Figure S12. (3*R*,1*E*)-(7-Ethoxy-7-oxo-3-methylhept-1-en-1-yl)dimethyl(phenyl)silane [(*R*)-**3af**].

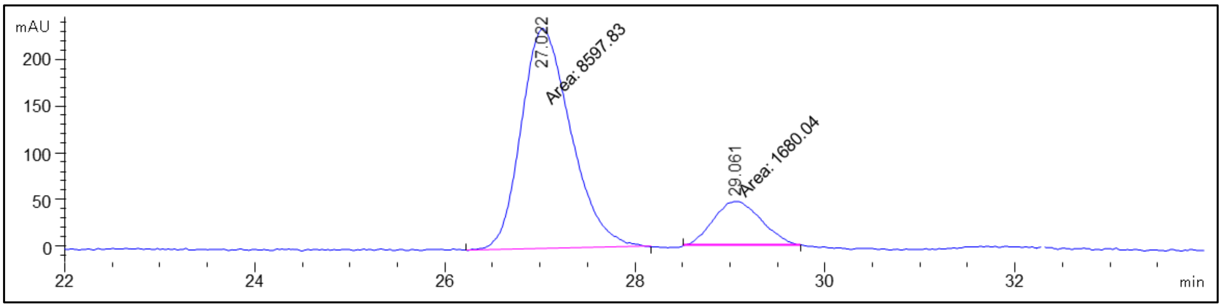

Peak #	RetTime [min]	Type	Width [min]	Area [mAU*s]	Height [mAU]	Area %
1	27.022	MM	0.6072	8597.83398	235.97845	83.6538
2	29.061	MM	0.5925	1680.03906	47.26057	16.3462

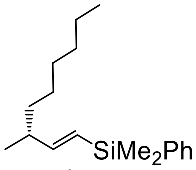

3ag

(3*R*,1*E*)-Dimethyl(3-methylnon-1-en-1-yl)(phenyl)silane (**3ag**): The enantiomeric ratio of **3ag** was determined by reversed phase HPLC analysis on a chiral stationary phase (*Daicel* Chiralcel OD-H column, column temperature 20 °C, solvent acetonitrile:water = 65:35, flow rate 0.3 mL/min): t_R = 37.1 min (major), t_R = 39.5 min (minor).

Figure S13. *rac*-(1*E*)-Dimethyl(3-methylnon-1-en-1-yl)(phenyl)silane (*rac*-**3ag**).

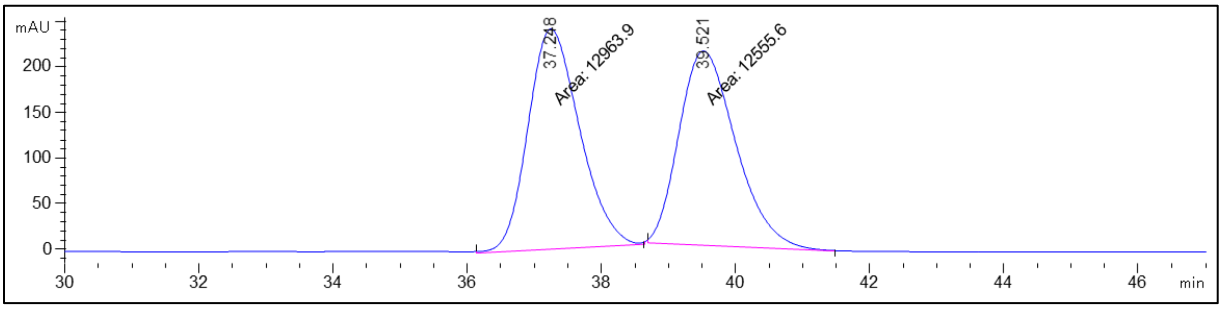

Peak #	RetTime [min]	Type	Width [min]	Area [mAU*s]	Height [mAU]	Area %
1	37.248	MM	0.8931	1.29639e4	241.93779	50.8001
2	39.521	MM	0.9816	1.25556e4	213.19102	49.1999

Figure S14. (3*R*,1*E*)-Dimethyl(3-methylnon-1-en-1-yl)(phenyl)silane [(*R*)-**3ag**].

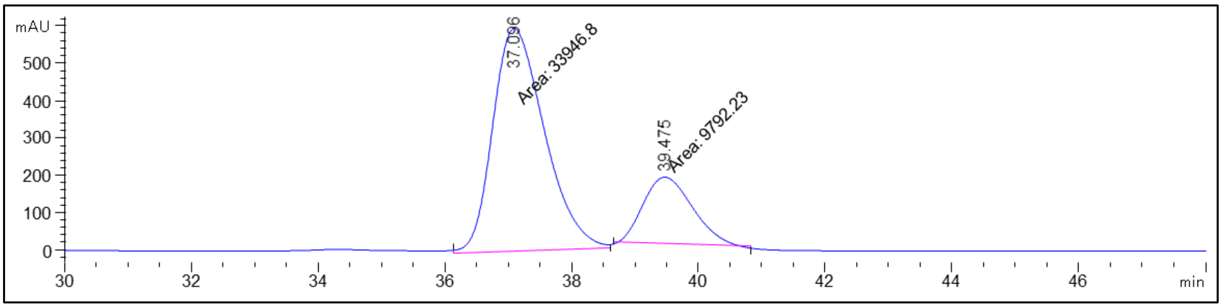

Peak #	RetTime [min]	Type	Width [min]	Area [mAU*s]	Height [mAU]	Area %
1	37.096	MM	0.9486	3.39468e4	596.46613	77.6122
2	39.475	MM	0.9093	9792.22852	179.48058	22.3878

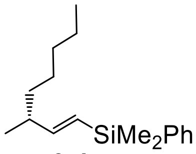

3ah

(3*R*,1*E*)-Dimethyl(3-methyloct-1-en-1-yl)(phenyl)silane (**3ah**): The enantiomeric ratio of **3ag** was determined by reversed phase HPLC analysis on a chiral stationary phase (*Daicel* Chiralcel AD-RH column, column temperature 20 °C, solvent acetonitrile:water = 65:35, flow rate 0.3 mL/min): t_R = 32.6 min (major), t_R = 35.5 min (minor).

Figure S15. *rac*-(1*E*)-Dimethyl(3-methyloct-1-en-1-yl)(phenyl)silane (*rac*-**3ah**).

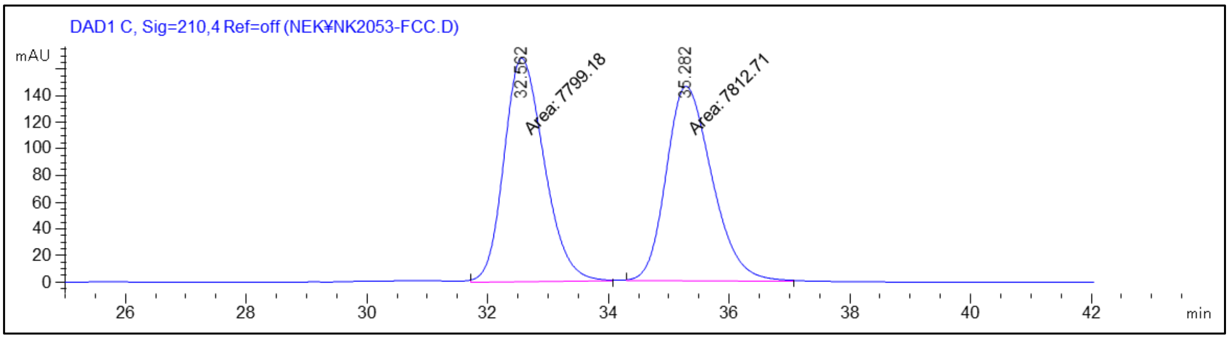

Peak #	RetTime [min]	Type	Width [min]	Area [mAU*s]	Height [mAU]	Area %
1	32.562	MM	0.7729	7799.18066	168.17897	49.9567
2	35.282	MM	0.8862	7812.71436	146.92735	50.0433

Figure S16. (3*R*,1*E*)-Dimethyl(3-methyloct-1-en-1-yl)(phenyl)silane [(*R*)-**3ah**].

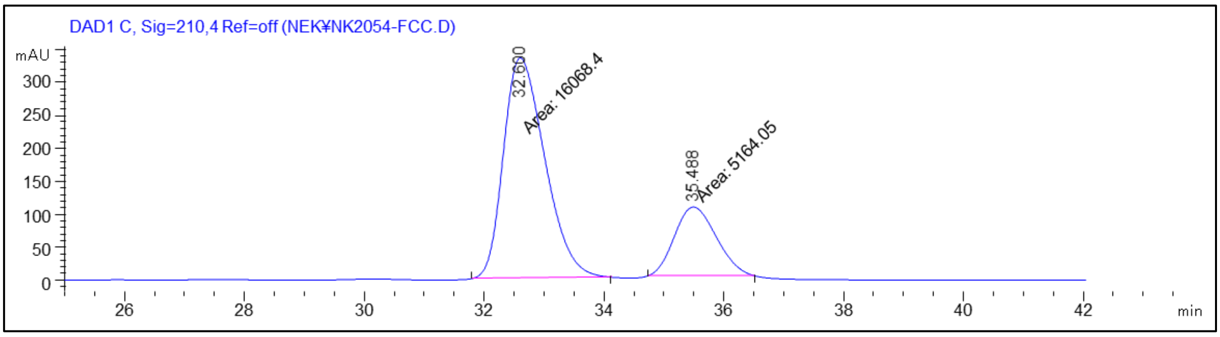

Peak #	RetTime [min]	Type	Width [min]	Area [mAU*s]	Height [mAU]	Area %
1	32.600	MM	0.8007	1.60684e4	334.44995	75.6785
2	35.488	MM	0.8169	5164.05029	105.36227	24.3215

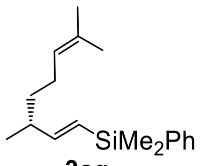

3ag

(3*R*,1*E*)-(3,7-Dimethylocta-1,6-dien-1-yl)dimethyl(phenyl)silane (**3ai**): The enantiomeric ratio of **3ai** was determined by reversed phase HPLC analysis on a chiral stationary phase (*Daicel* Chiralcel AD-RH column, column temperature 20 °C, solvent acetonitrile:water = 65:35, flow rate 0.3 mL/min): t_R = 27.0 min (major), t_R = 29.8 min (minor).

Figure S17. *rac*-(1*E*)-(3,7-Dimethylocta-1,6-dien-1-yl)dimethyl(phenyl)silane (*rac*-**3ai**).

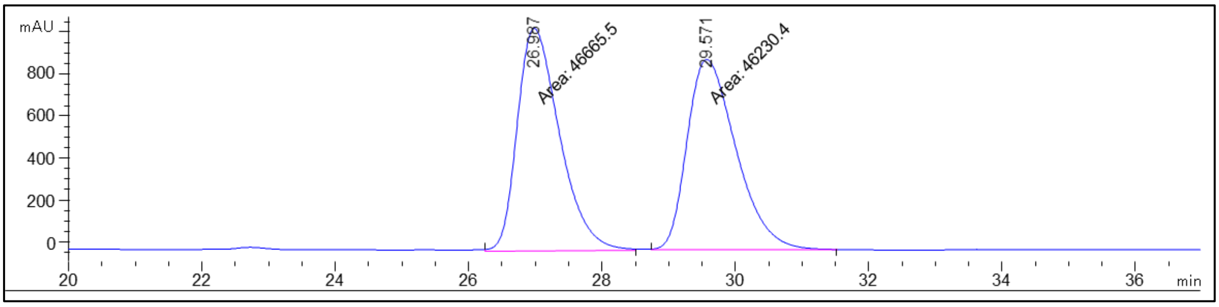

Peak #	RetTime [min]	Type	Width [min]	Area [mAU*s]	Height [mAU]	Area %
1	26.987	MM	0.7349	4.66655e4	1058.30798	50.2342
2	29.571	MM	0.8517	4.62304e4	904.70404	49.7658

Figure S18. (3*R*,1*E*)-(3,7-Dimethylocta-1,6-dien-1-yl)dimethyl(phenyl)silane [(*R*)-**3ai**].

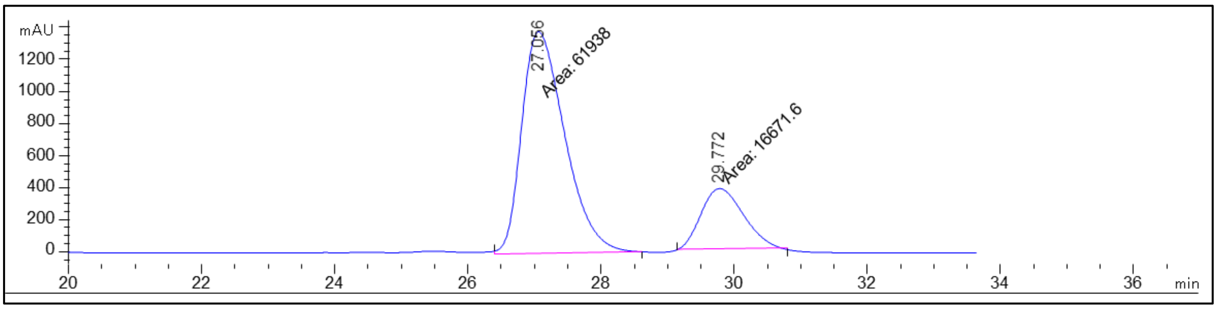

Peak #	RetTime [min]	Type	Width [min]	Area [mAU*s]	Height [mAU]	Area %
1	27.056	MM	0.7499	6.19380e4	1376.54541	78.7919
2	29.772	MM	0.7384	1.66716e4	376.28876	21.2081

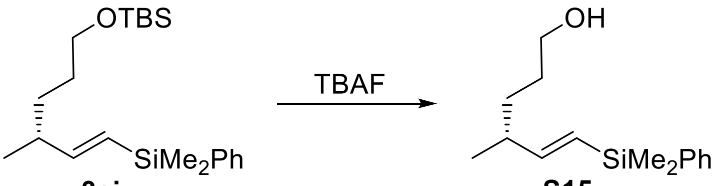

3aj **S15**

Scheme S12. Deprotection of **3aj**.

(3*R*,1*E*)-tert-Butyl((6-(dimethyl(phenyl)silyl)-4-methylhex-5-en-1-yl)oxy)dimethyl silane (**3aj**): The enantiomeric ratio of **3aj** was determined, after deprotection of TBS group with TBAF,[S12] by HPLC analysis on a chiral stationary phase (*Daicel* Chiralcel OD-H column, column temperature 20 °C, solvent *n*-heptane:isopropanol = 99:1, flow rate 0.4 mL/min): t_R = 27.1 min (minor), t_R = 31.1 min (major).

Figure S19. *rac*-(1*E*)-6-(dimethyl(phenyl)silyl)-4-methylhex-5-en-1-ol (*rac*-**S15**).

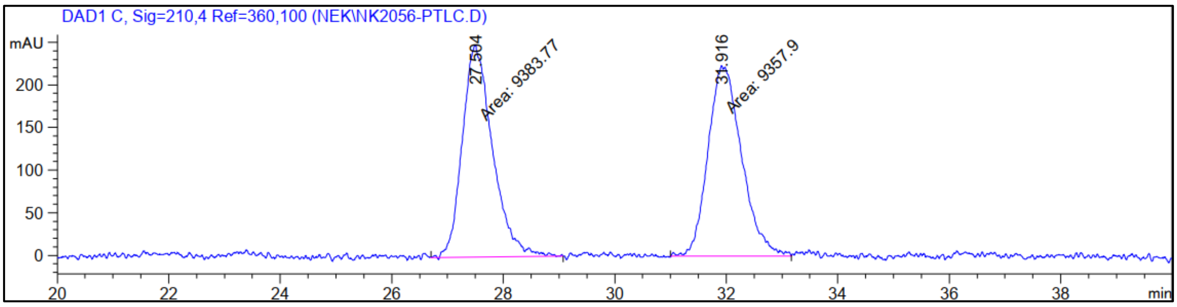

Peak #	RetTime [min]	Type	Width [min]	Area [mAU*s]	Height [mAU]	Area %
1	27.504	MM	0.6285	9383.76758	248.84738	50.0690
2	31.916	MM	0.6974	9357.90137	223.63718	49.9310

Figure S20. (3*R*,1*E*)-6-(dimethyl(phenyl)silyl)-4-methylhex-5-en-1-ol [(*R*)-**S15**].

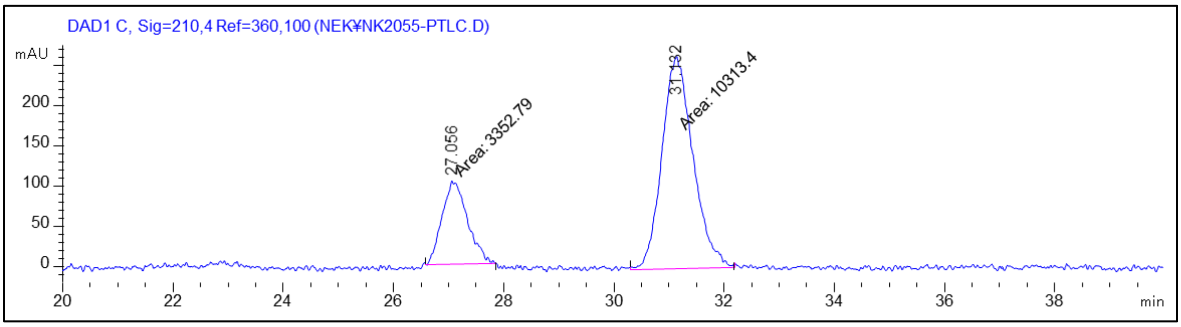

Peak #	RetTime [min]	Type	Width [min]	Area [mAU*s]	Height [mAU]	Area %
1	27.056	MM	0.5378	3352.79077	103.90571	24.5335
2	31.132	MM	0.6528	1.03134e4	263.32666	75.4665

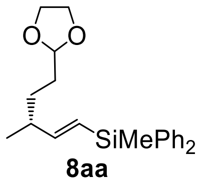

8aa

(3*R*,1*E*)-(5-(1,3-Dioxolan-2-yl)-3-methylpent-1-en-1-yl)(methyl)diphenylsilane (**8aa**): The enantiomeric ratio of **8aa** was determined by HPLC analysis on a chiral stationary phase (*Daicel* Chiralcel OD-H column, column temperature 20 °C, solvent *n*-heptane:isopropanol = 99.9:0.1, flow rate 0.4 mL/min): t_R = 48.4 min (minor), t_R = 58.8 min (minor).

Figure S21. *rac*-(1*E*)-(5-(1,3-Dioxolan-2-yl)-3-methylpent-1-en-1-yl)(methyl) diphenylsilane (*rac*-**8aa**).

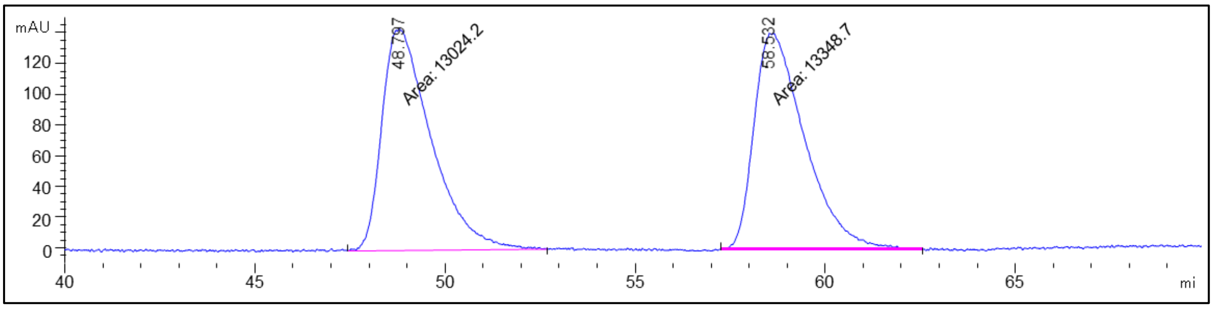

Peak #	RetTime [min]	Type	Width [min]	Area [mAU*s]	Height [mAU]	Area %
1	48.797	MM	1.5070	1.30242e4	144.04210	49.3848
2	58.532	MM	1.5718	1.33487e4	141.54131	50.6152

Figure S22. (3*R*,1*E*)-(5-(1,3-Dioxolan-2-yl)-3-methylpent-1-en-1-yl)(methyl) diphenylsilane [(*R*)-**8aa**].

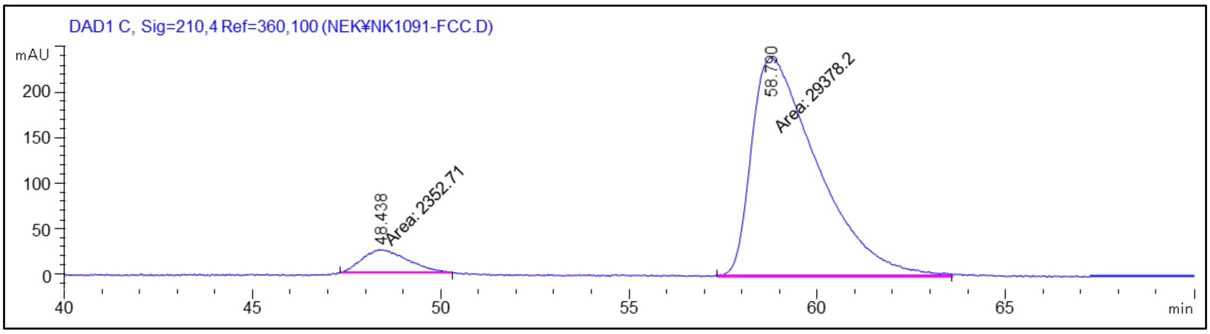

Peak #	RetTime [min]	Type	Width [min]	Area [mAU*s]	Height [mAU]	Area %
1	48.438	MM	1.4785	2352.70874	26.52165	7.4146
2	58.790	MM	2.0308	2.93782e4	241.10405	92.5854

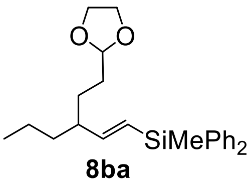

8ba

(3*R*,1*E*)-(3-(2-(1,3-Dioxolan-2-yl)ethyl)hex-1-en-1-yl)(methyl)diphenylsilane (**8ba**): The enantiomeric ratio of **8ba** was determined by HPLC analysis on a chiral stationary phase (*Daicel* Chiralcel OD-H column, column temperature 20 °C, solvent *n*-heptane:isopropanol = 99.9:0.1, flow rate 0.4 mL/min): t_R = 37.3 min (minor), t_R = 40.2 min (major).

Figure S23. *rac*-(1*E*)-(3-(2-(1,3-dioxolan-2-yl)ethyl)hex-1-en-1-yl)(methyl)diphenyl silane (*rac*-**8ba**).

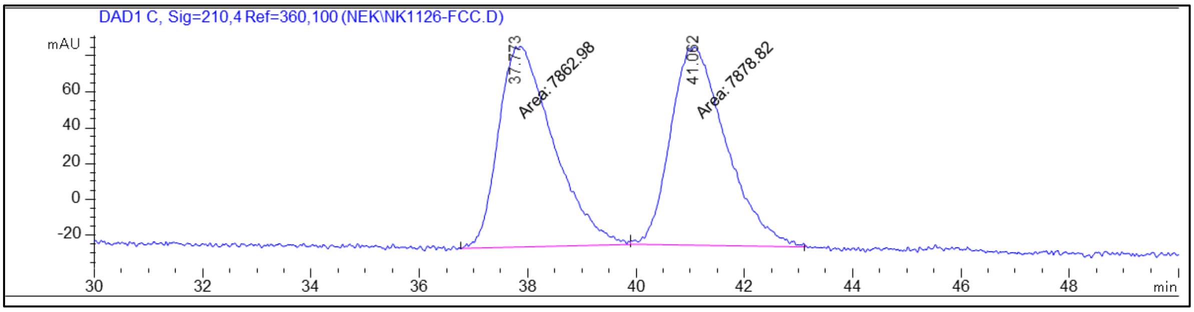

Peak #	RetTime [min]	Type	Width [min]	Area [mAU*s]	Height [mAU]	Area %
1	37.773	MM	1.1731	7862.98389	111.70764	49.9497
2	41.062	MM	1.1897	7878.82080	110.37642	50.0503

Figure S24. (3*R*,1*E*)-(3-(2-(1,3-dioxolan-2-yl)ethyl)hex-1-en-1-yl)(methyl)diphenyl silane [(*R*)-**8ba**].

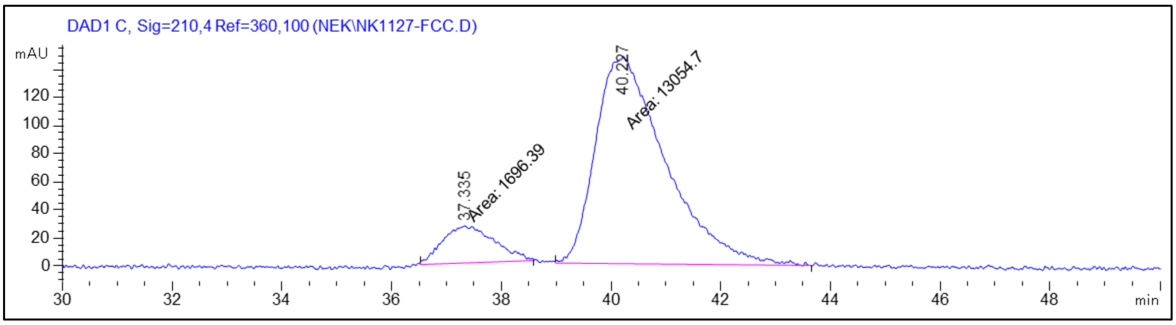

Peak #	RetTime [min]	Type	Width [min]	Area [mAU*s]	Height [mAU]	Area %
1	37.335	MM	1.0834	1696.39148	26.09584	11.4989
2	40.227	MM	1.4701	1.30547e4	147.99866	88.5011

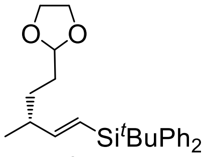

9aa

(3*R*,1*E*)-(5-(1,3-Dioxolan-2-yl)-3-methylpent-1-en-1-yl)(*tert*-butyl)diphenylsilane (**9aa**): The enantiomeric ratio of **9aa** was determined by HPLC analysis on a chiral stationary phase (*Daicel* Chiralcel OD-H column, column temperature 20 °C, solvent *n*-heptane:isopropanol = 99.9:0.1, flow rate 0.5 mL/min): t_R = 28.9 min (minor), t_R = 32.2 min (major).

Figure S25. *rac*-(1*E*)-(5-(1,3-dioxolan-2-yl)-3-methylpent-1-en-1-yl)(*tert*-butyl) diphenylsilane (*rac*-**9aa**).

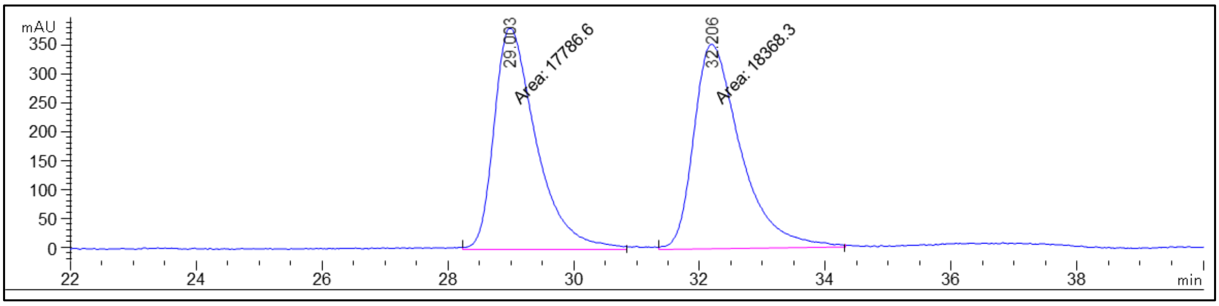

Peak #	RetTime [min]	Type	Width [min]	Area [mAU*s]	Height [mAU]	Area %
1	29.003	MM	0.7778	1.77866e4	381.11948	49.1955
2	32.206	MM	0.8658	1.83683e4	353.59235	50.8045

Figure S26. (3*R*,1*E*)-(5-(1,3-dioxolan-2-yl)-3-methylpent-1-en-1-yl)(*tert*-butyl) diphenylsilane [(*R*)-**9aa**].

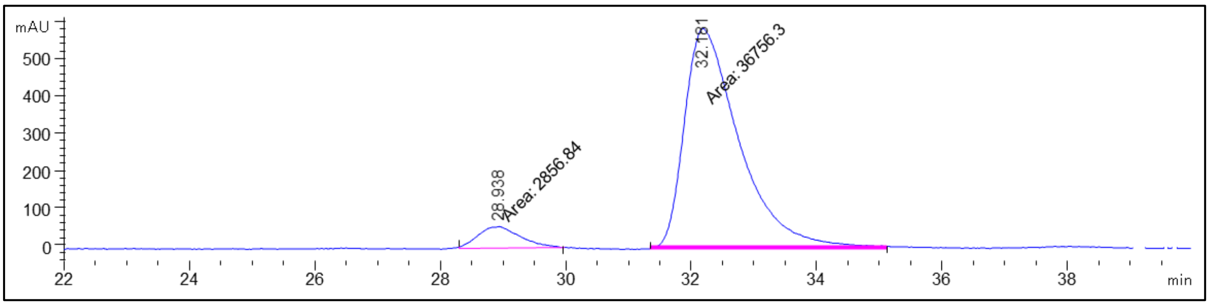

Peak #	RetTime [min]	Type	Width [min]	Area [mAU*s]	Height [mAU]	Area %
1	28.938	MM	0.7997	2856.84302	59.53906	7.2119
2	32.181	MM	1.0336	3.67563e4	592.71918	92.7881

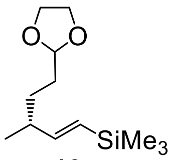

10aa

(3*R*,1*E*)-(5-(1,3-Dioxolan-2-yl)-3-methylpent-1-en-1-yl)trimethylsilane (**10aa**): The enantiomeric ratio of **10aa** was determined by HPLC analysis on a chiral stationary phase (*Daicel* Chiralcel OD-H column, column temperature 20 °C, solvent *n*-heptane:isopropanol = 99.9:0.1, flow rate 0.3 mL/min): t_R = 25.3 min (minor), t_R = 33.3 min (major).

Figure S27. *rac*-(1*E*)-(5-(1,3-Dioxolan-2-yl)-3-methylpent-1-en-1-yl)trimethylsilane (*rac*-**10aa**).

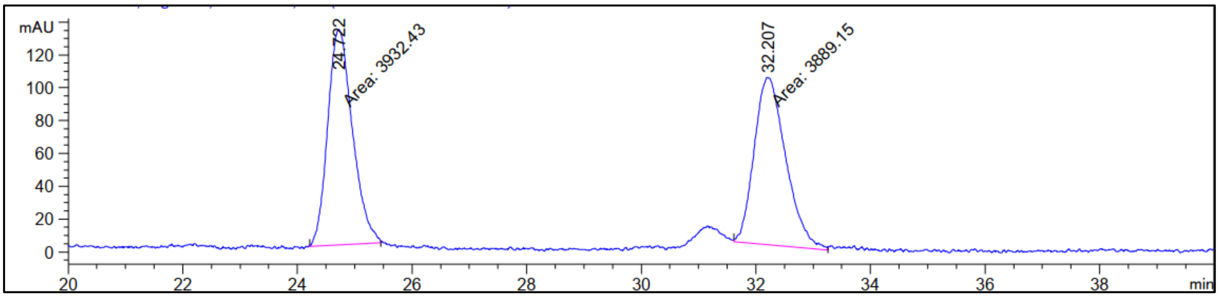

Peak #	RetTime [min]	Type	Width [min]	Area [mAU*s]	Height [mAU]	Area %
1	24.722	MM	0.4996	3932.43188	131.18877	50.2767
2	32.207	MM	0.6374	3889.15137	101.68835	49.7233

Figure S28. (3*R*,1*E*)-(5-(1,3-Dioxolan-2-yl)-3-methylpent-1-en-1-yl)trimethylsilane [(*R*)-**10aa**].

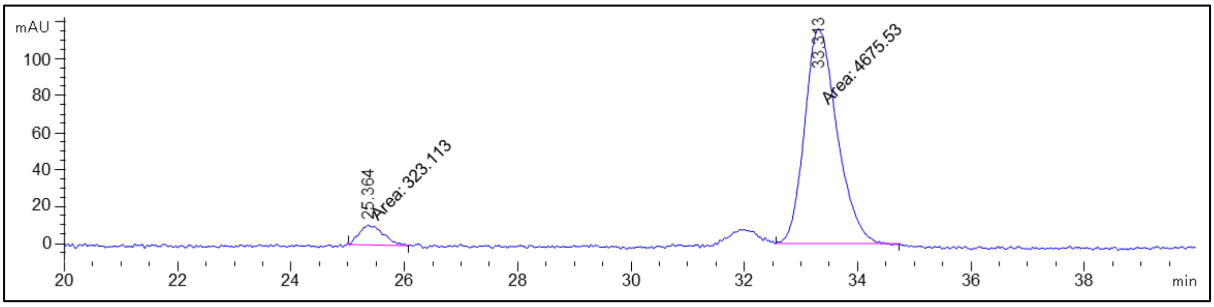

Peak #	RetTime [min]	Type	Width [min]	Area [mAU*s]	Height [mAU]	Area %
1	25.364	MM	0.4915	323.11307	10.95616	6.4640
2	33.313	MM	0.6692	4675.52686	116.44874	93.5360

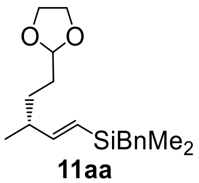

11aa

(3*R*,1*E*)-(5-(1,3-Dioxolan-2-yl)-3-methylpent-1-en-1-yl)(benzyl)dimethylsilane (**11aa**): The enantiomeric ratio of **11aa** was determined by HPLC analysis on a chiral stationary phase (*Daicel* Chiralcel OD-H column, column temperature 20 °C, solvent *n*-heptane:isopropanol = 99.9:0.1, flow rate 0.4 mL/min): t_R = 55.0 min (minor), t_R = 57.4 min (major).

Figure S29. *rac*-(1*E*)-(5-(1,3-Dioxolan-2-yl)-3-methylpent-1-en-1-yl)(benzyl) dimethylsilane (*rac*-**11aa**).

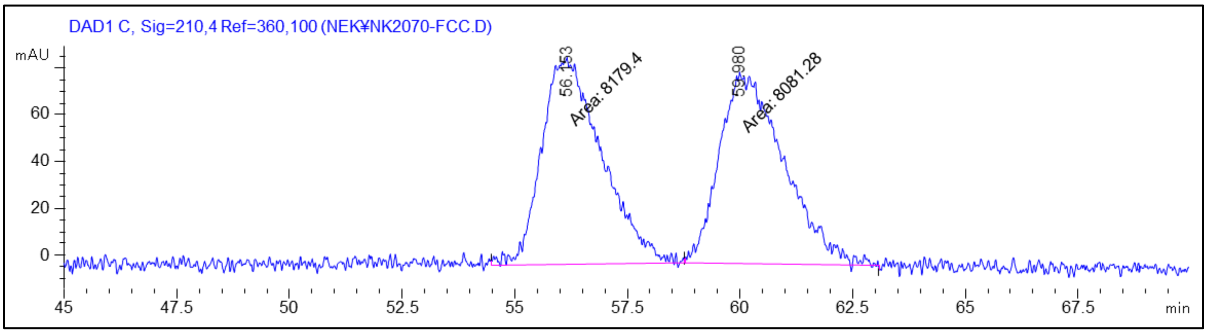

Peak #	RetTime [min]	Type	Width [min]	Area [mAU*s]	Height [mAU]	Area %
1	56.153	MM	1.5449	8179.39502	88.24281	50.3017
2	59.980	MM	1.6481	8081.27783	81.72255	49.6983

Figure S30. (3*R*,1*E*)-(5-(1,3-Dioxolan-2-yl)-3-methylpent-1-en-1-yl)(benzyl) dimethylsilane [(*R*)-**11aa**].

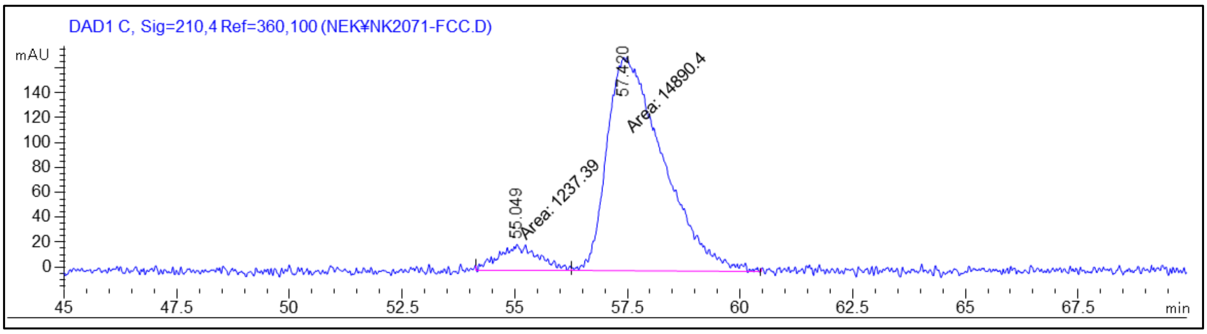

Peak #	RetTime [min]	Type	Width [min]	Area [mAU*s]	Height [mAU]	Area %
1	55.049	MM	0.9839	1237.39136	20.96048	7.6724
2	57.420	MM	1.4479	1.48904e4	171.39729	92.3276

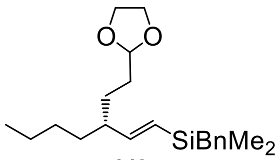

11ba

(3*R*,1*E*)-(3-(2-(1,3-Dioxolan-2-yl)ethyl)hept-1-en-1-yl)(benzyl)dimethylsilane (**11ba**): The enantiomeric ratio of **11ba** was determined by HPLC analysis on a chiral stationary phase (*Daicel* Chiralcel OD-H column, column temperature 20 °C, solvent *n*-heptane:isopropanol = 99.9:0.1, flow rate 0.4 mL/min): t_R = 42.2 min (major), t_R = 48.2 min (minor).

Figure S31. *rac*-(1*E*)-(3-(2-(1,3-Dioxolan-2-yl)ethyl)hept-1-en-1-yl)(benzyl)dimethyl silane (*rac*-**11ba**).

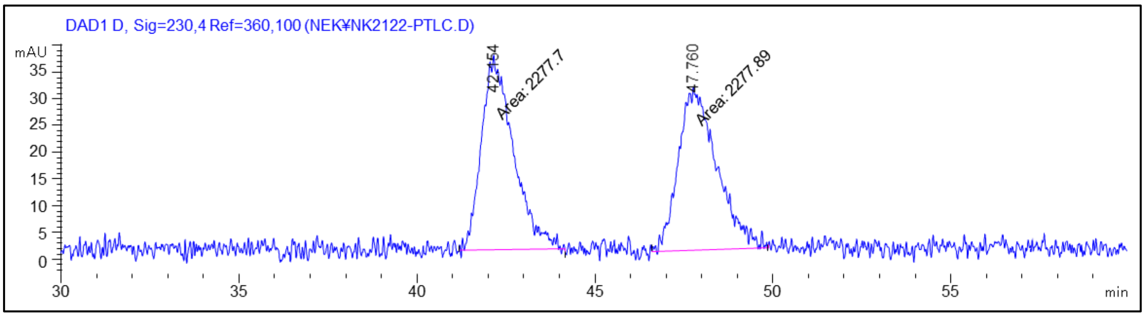

Peak #	RetTime [min]	Type	Width [min]	Area [mAU*s]	Height [mAU]	Area %
1	42.154	MM	1.0398	2277.69873	36.50965	49.9979
2	47.760	MM	1.2453	2277.88940	30.48608	50.0021

Figure S32. (3*R*,1*E*)-(3-(2-(1,3-Dioxolan-2-yl)ethyl)hept-1-en-1-yl)(benzyl) dimethyl silane [(*R*)-**11ba**].

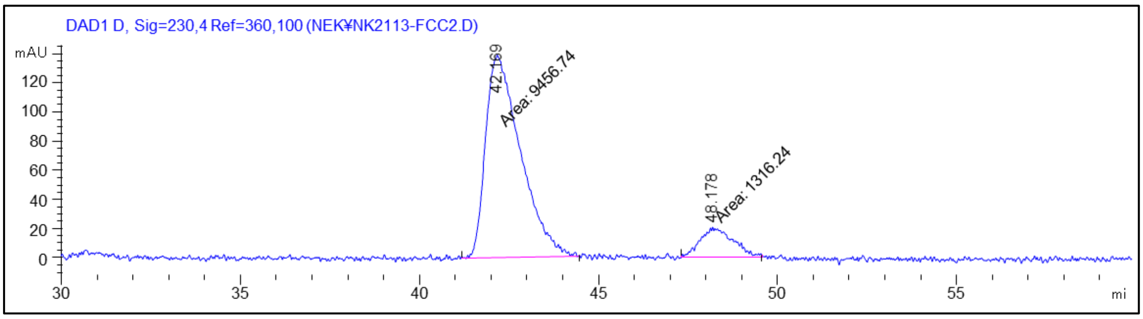

Peak #	RetTime [min]	Type	Width [min]	Area [mAU*s]	Height [mAU]	Area %
1	42.169	MM	1.1322	9456.73730	139.20647	87.7820
2	48.178	MM	1.0491	1316.23730	20.91092	12.2180

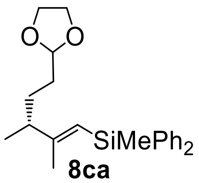

8ca

(3*R*,1*E*)-(5-(1,3-Dioxolan-2-yl)-2,3-dimethylpent-1-en-1-yl)(methyl)diphenylsilane (8ca): The enantiomeric ratio of **8ca** was determined by HPLC analysis on a chiral stationary phase (*Daicel* Chiralcel OD-H column, column temperature 20 °C, solvent *n*-heptane:isopropanol = 99.7:0.3, flow rate 0.4 mL/min): t_R = 22.4 min (minor), t_R = 25.6 min (major).

Figure S33. *rac*-(1*E*)-(5-(1,3-Dioxolan-2-yl)-2,3-dimethylpent-1-en-1-yl)(methyl) diphenylsilane (*rac*-**8ca**).

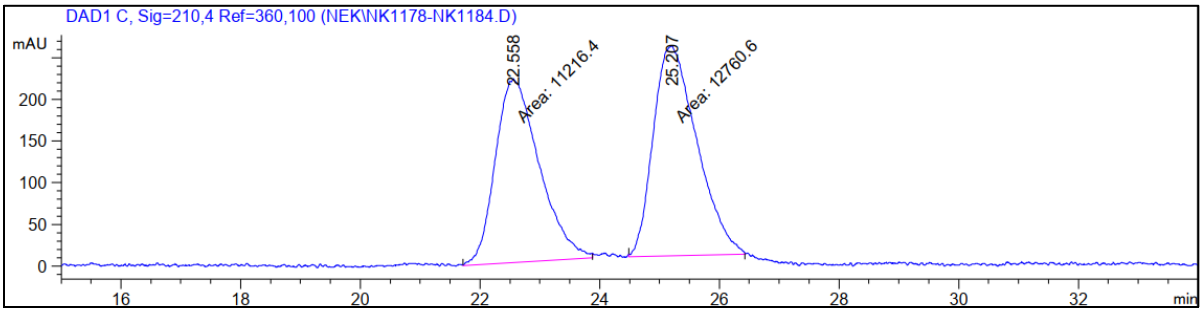

Peak #	RetTime [min]	Type	Width [min]	Area [mAU*s]	Height [mAU]	Area %
1	22.558	MM	0.8511	1.12164e4	219.64064	46.7797
2	25.207	MM	0.8443	1.27606e4	251.90131	53.2203

Figure S34. (3*R*,1*E*)-(5-(1,3-Dioxolan-2-yl)-2,3-dimethylpent-1-en-1-yl)(methyl) diphenylsilane [(*R*)-**8ca**].

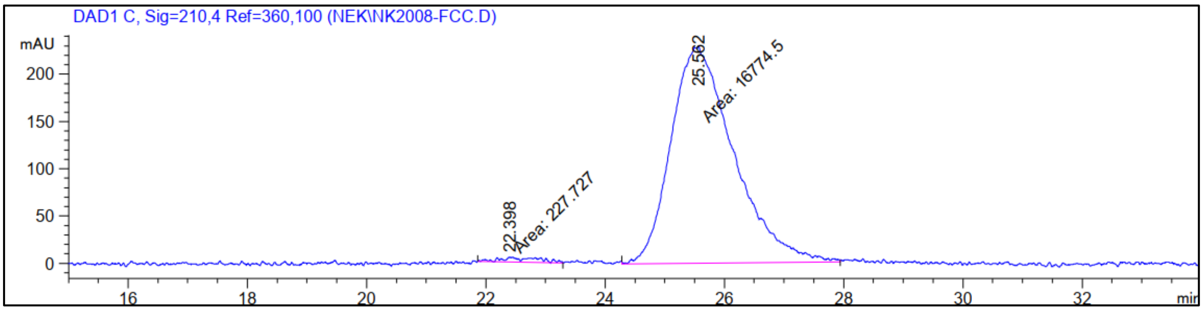

Peak #	RetTime [min]	Type	Width [min]	Area [mAU*s]	Height [mAU]	Area %
1	22.398	MM	0.7269	227.72737	5.22167	1.3394
2	25.562	MM	1.2155	1.67745e4	230.01118	98.6606

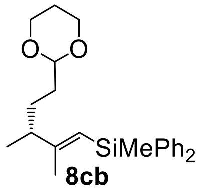
8cb

(3*R*,1*E*)-(5-(1,3-Dioxan-2-yl)-2,3-dimethylpent-1-en-1-yl)(methyl)diphenylsilane (**8cb**): The enantiomeric ratio of **8cb** was determined by HPLC analysis on a chiral stationary phase (*Daicel* Chiralcel OD-H column, column temperature 20 °C, solvent *n*-heptane:isopropanol = 99.7:0.3, flow rate 0.4 mL/min): t_R = 20.2 min (minor), t_R = 24.2 min (major).

Figure S35. *rac*-(1*E*)-(5-(1,3-Dioxan-2-yl)-2,3-dimethylpent-1-en-1-yl)(methyl) diphenylsilane (*rac*-**8cb**).

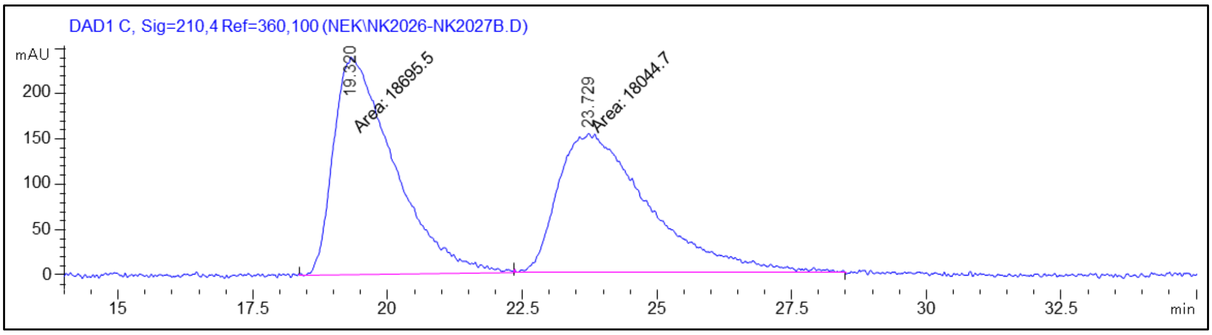

Peak #	RetTime [min]	Type	Width [min]	Area [mAU*s]	Height [mAU]	Area %
1	19.320	MM	1.2981	1.86955e4	240.04247	50.8858
2	23.729	MM	1.9585	1.80447e4	153.56114	49.1142

Figure S36. (3*R*,1*E*)-(5-(1,3-Dioxan-2-yl)-2,3-dimethylpent-1-en-1-yl)(methyl) diphenylsilane [(*R*)-**8cb**].

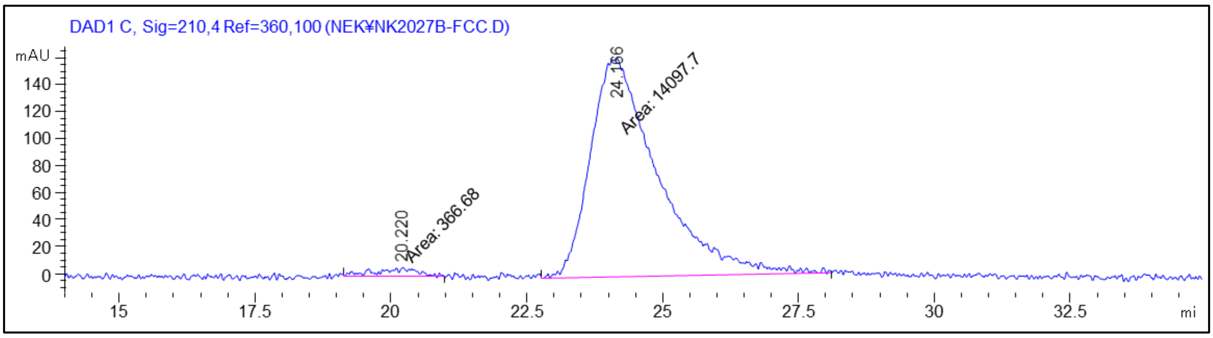

Peak #	RetTime [min]	Type	Width [min]	Area [mAU*s]	Height [mAU]	Area %
1	20.220	MM	0.8717	366.67984	7.01070	2.5351
2	24.166	MM	1.4489	1.40977e4	162.16747	97.4649

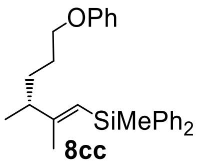

8cc

(3*R*,1*E*)-(2,3-Dimethyl-6-phenoxyhex-1-en-1-yl)(methyl)diphenylsilane (8cc): The enantiomeric ratio of **8cc** was determined by HPLC analysis on a chiral stationary phase (*Daicel* Chiralcel OD-H column, column temperature 20 °C, solvent *n*-heptane:isopropanol = 99.5:0.5, flow rate 0.6 mL/min): t_R = 17.7 min (minor), t_R = 40.7 min (major).

Figure S37. *rac*-(1*E*)-(2,3-Dimethyl-6-phenoxyhex-1-en-1-yl)(methyl)diphenylsilane (*rac*-**8cc**).

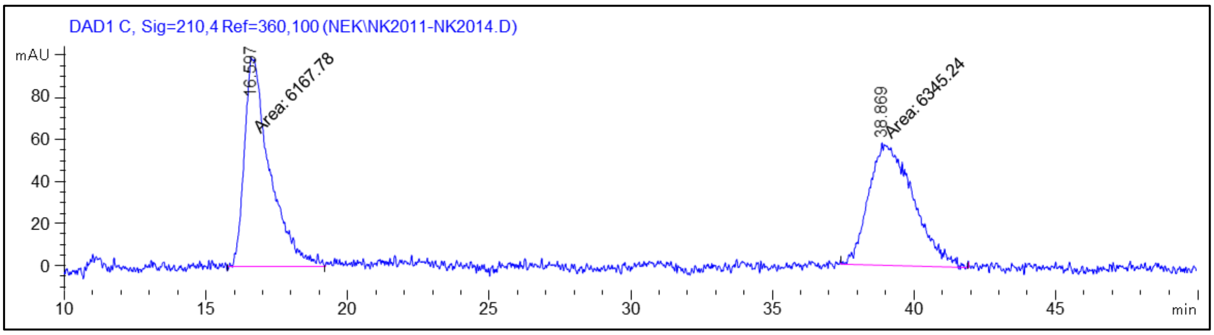

Peak #	RetTime [min]	Type	Width [min]	Area [mAU*s]	Height [mAU]	Area %
1	16.597	MM	1.0391	6167.77832	98.92448	49.2909
2	38.869	MM	1.8162	6345.24365	58.22958	50.7091

Figure S38. (3*R*,1*E*)-(2,3-Dimethyl-6-phenoxyhex-1-en-1-yl)(methyl)diphenylsilane [(*R*)-**8cc**].

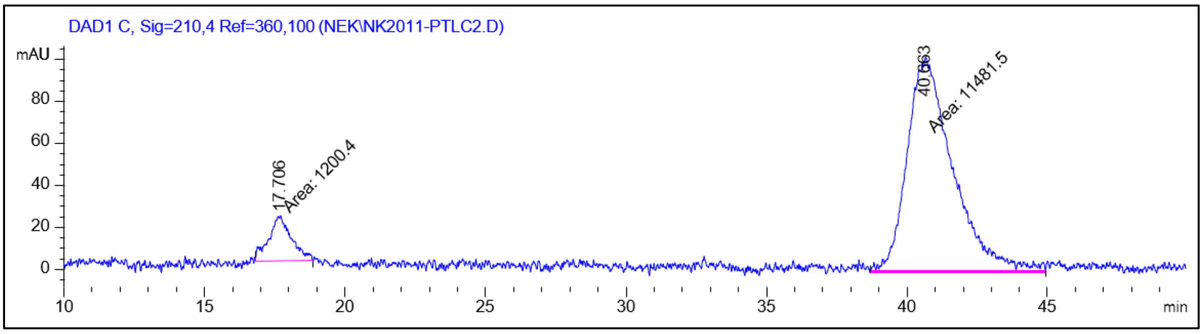

Peak #	RetTime [min]	Type	Width [min]	Area [mAU*s]	Height [mAU]	Area %
1	17.706	MM	0.9267	1200.40198	21.58850	9.4655
2	40.663	MM	1.8658	1.14815e4	102.56123	90.5345

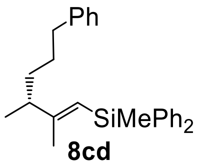

8cd

(3*R*,1*E*)-(2,3-Dimethyl-6-phenylhex-1-en-1-yl)(methyl)diphenylsilane (8cd): The enantiomeric ratio of **8cd** was determined by HPLC analysis on a chiral stationary phase (*Daicel* Chiralcel IB column, column temperature 20 °C, solvent *n*-heptane:isopropanol = 100:0, flow rate 0.2 mL/min): t_R = 29.8 min (minor), t_R = 32.2 min (major).

Figure S39. *rac*-(1*E*)-(2,3-Dimethyl-6-phenoxyhex-1-en-1-yl)(methyl)diphenyl silane (*rac*-**8cd**).

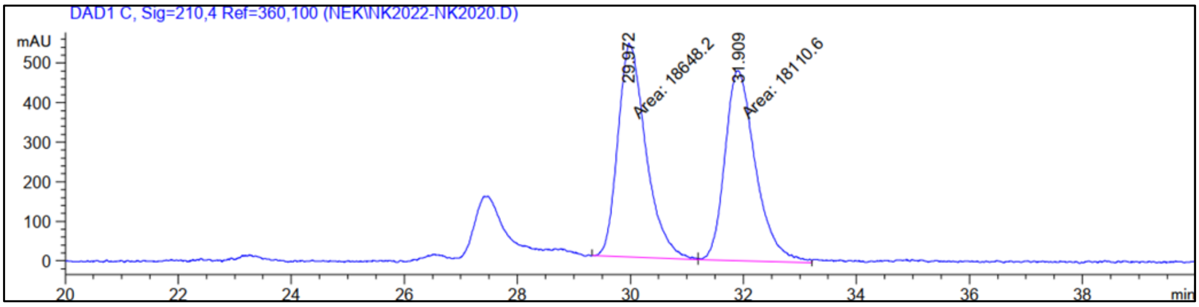

Peak #	RetTime [min]	Type	Width [min]	Area [mAU*s]	Height [mAU]	Area %
1	29.972	MM	0.5746	1.86482e4	540.88324	50.7313
2	31.909	MM	0.6285	1.81106e4	480.28009	49.2687

Figure S40. (3*R*,1*E*)-(2,3-Dimethyl-6-phenoxyhex-1-en-1-yl)(methyl)diphenyl silane [*R*)-**8cd**].

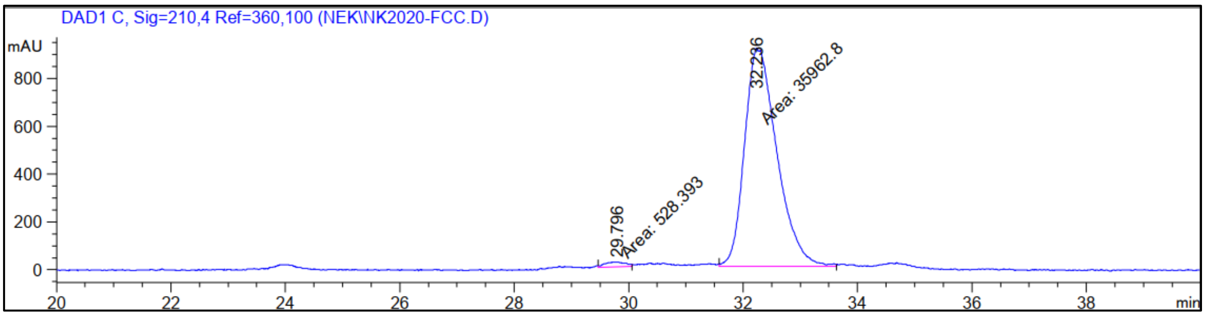

Peak #	RetTime [min]	Type	Width [min]	Area [mAU*s]	Height [mAU]	Area %
1	29.796	MM	0.4261	528.39319	20.66593	1.4480
2	32.236	MM	0.6561	3.59628e4	913.60663	98.5520

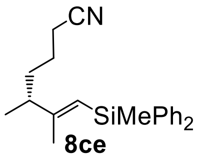

8ce

(3*R*,1*E*)-5,6-Dimethyl-7-(methyldiphenylsilyl)hept-6-enenitrile (8ce): The enantiomeric ratio of **11ae** was determined by HPLC analysis on a chiral stationary phase (*Daicel* Chiralcel AS-H column, column temperature 20 °C, solvent *n*-heptane:isopropanol = 99:1, flow rate 0.4 mL/min): t_R = 22.2 min (minor), t_R = 28.7 min (major).

Figure S41. *rac*-(1*E*)- 5,6-Dimethyl-7-(methyldiphenylsilyl)hept-6-enenitrile (*rac*-**8ce**).

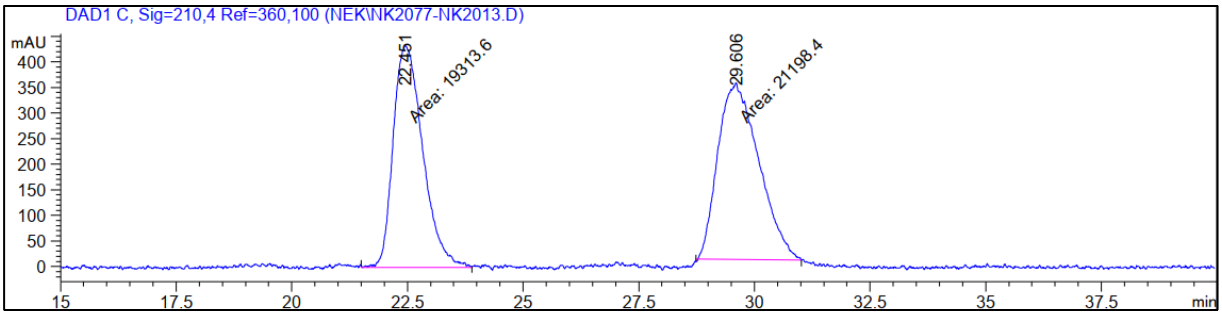

Peak #	RetTime [min]	Type	Width [min]	Area [mAU*s]	Height [mAU]	Area %
1	22.451	MM	0.7394	1.93136e4	435.33670	47.6737
2	29.606	MM	1.0245	2.11984e4	344.87222	52.3263

Figure S42. (3*R*,1*E*)- 5,6-Dimethyl-7-(methyldiphenylsilyl)hept-6-enenitrile [(*R*)-**8ce**].

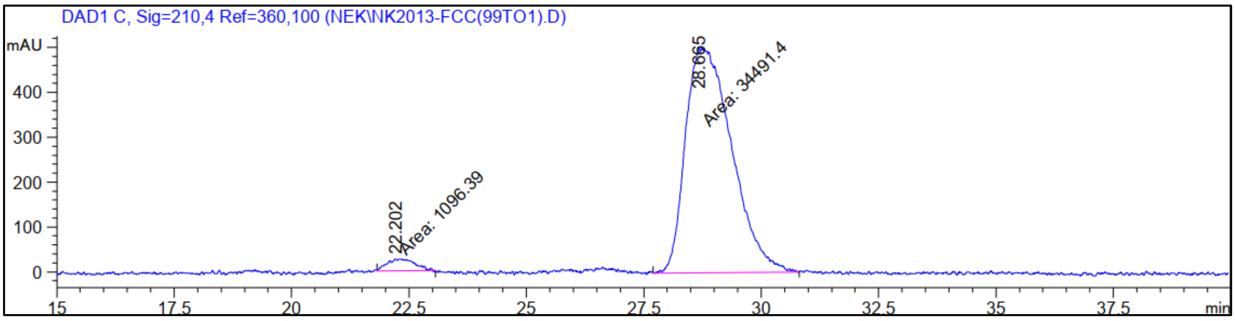

Peak #	RetTime [min]	Type	Width [min]	Area [mAU*s]	Height [mAU]	Area %
1	22.202	MM	0.6813	1096.38745	26.82271	3.0808
2	28.665	MM	1.1433	3.44914e4	502.81821	96.9192

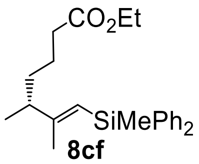

8cf

Ethyl (3*R*,1*E*)-5,6-Dimethyl-7-(methyldiphenylsilyl)hept-6-enoate (8cf): The enantiomeric ratio of **8cf** was determined by HPLC analysis on a chiral stationary phase (*Daicel* Chiralcel OD-H column, column temperature 20 °C, solvent *n*-heptane:isopropanol = 99.9:0.1, flow rate 0.4 mL/min): t_R = 40.0 min (major), t_R = 44.8 min (minor).

Figure S43. *rac*-Ethyl (1*E*)-5,6-dimethyl-7-(methyldiphenylsilyl)hept-6-enoate (*rac*-**8cf**).

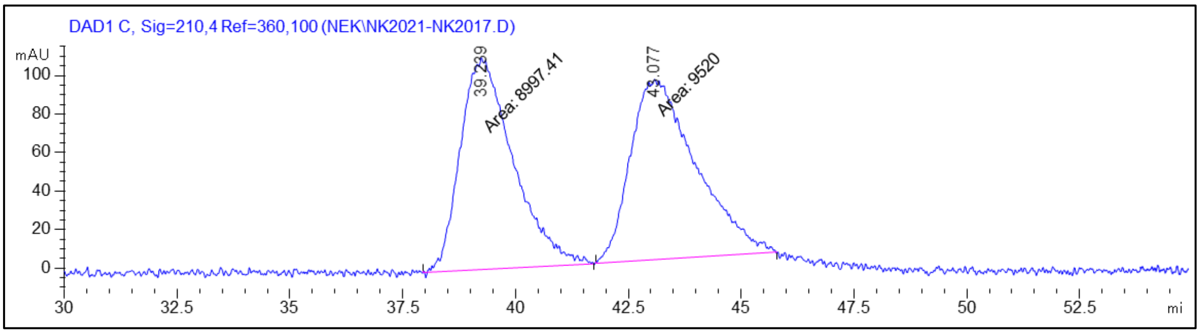

Peak #	RetTime [min]	Type	Width [min]	Area [mAU*s]	Height [mAU]	Area %
1	39.239	MM	1.3599	8997.41211	110.27200	48.5889
2	43.077	MM	1.6918	9520.00488	93.78511	51.4111

Figure S44. Ethyl (3*R*,1*E*)-5,6-dimethyl-7-(methyldiphenylsilyl)hept-6-enoate [(*R*)-**8cf**].

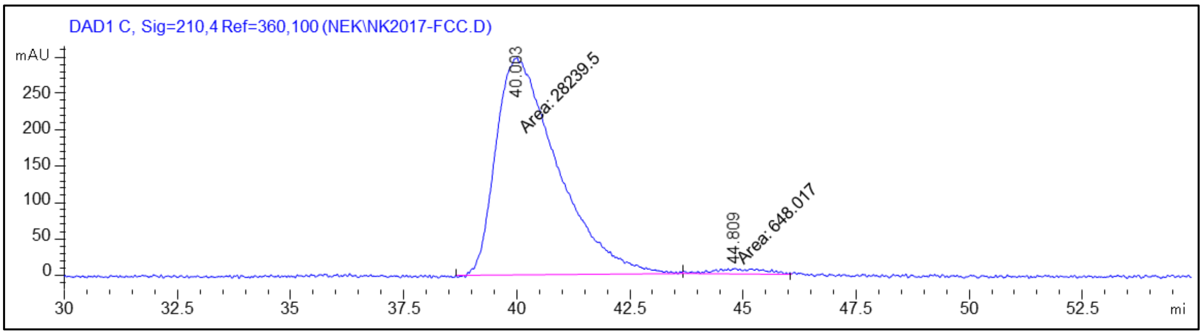

Peak #	RetTime [min]	Type	Width [min]	Area [mAU*s]	Height [mAU]	Area %
1	40.003	MM	1.5765	2.82395e4	298.54410	97.7568
2	44.809	MM	1.3666	648.01715	7.90328	2.2432

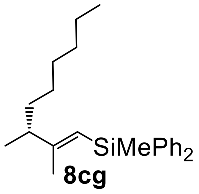

8cg

(3*R*,1*E*)-(2,3-Dimethylnon-1-en-1-yl)(methyl)diphenylsilane (8cg): The enantiomeric ratio of **8cg** was determined by reversed phase HPLC analysis on a chiral stationary phase (*Daicel* Chiralcel OJ-RH column, column temperature 20 °C, solvent acetonitrile:water = 65:35, flow rate 0.3 mL/min): t_R = 84.4 min (major), t_R = 107.4 min (minor). (minor).

Figure S45. *rac*-(1*E*)-(2,3-Dimethylnon-1-en-1-yl)(methyl)diphenylsilane (*rac*-**8cg**).

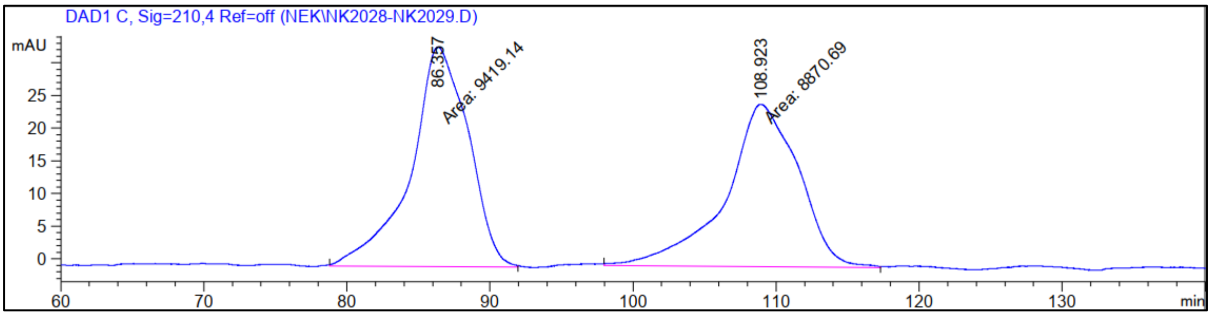

Peak #	RetTime [min]	Type	Width [min]	Area [mAU*s]	Height [mAU]	Area %
1	86.357	MM	4.6683	9419.14160	33.62778	51.4993
2	108.923	MM	5.9438	8870.68848	24.87358	48.5007

Figure S46. (3*R*,1*E*)-(2,3-Dimethylnon-1-en-1-yl)(methyl)diphenylsilane [(*R*)-**8cg**].

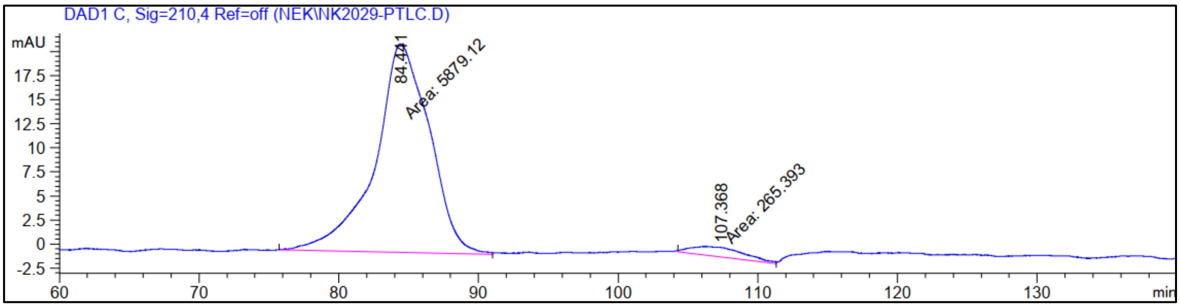

Peak #	RetTime [min]	Type	Width [min]	Area [mAU*s]	Height [mAU]	Area %
1	84.441	MM	4.5263	5879.12207	21.64792	95.6808
2	107.368	MM	4.3549	265.39267	1.01569	4.3192

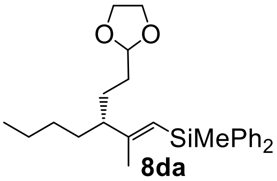

8da

(3*R*,1*E*)-(3-(2-(1,3-Dioxolan-2-yl)ethyl)-2-methylhept-1-en-1-yl)(methyl)diphenyl silane (8da): The enantiomeric ratio of **8da** was determined by HPLC analysis on a chiral stationary phase (*Daicel* Chiralcel OD-H column, column temperature 20 °C, solvent *n*-heptane:isopropanol = 99.9:0.1, flow rate 0.2 mL/min): t_R = 67.4 min (minor), t_R = 69.9 min (major).

Figure S47. *rac*-(1*E*)-(3-(2-(1,3-Dioxolan-2-yl)ethyl)-2-methylhept-1-en-1-yl)(methyl) diphenylsilane (*rac*-**8da**).

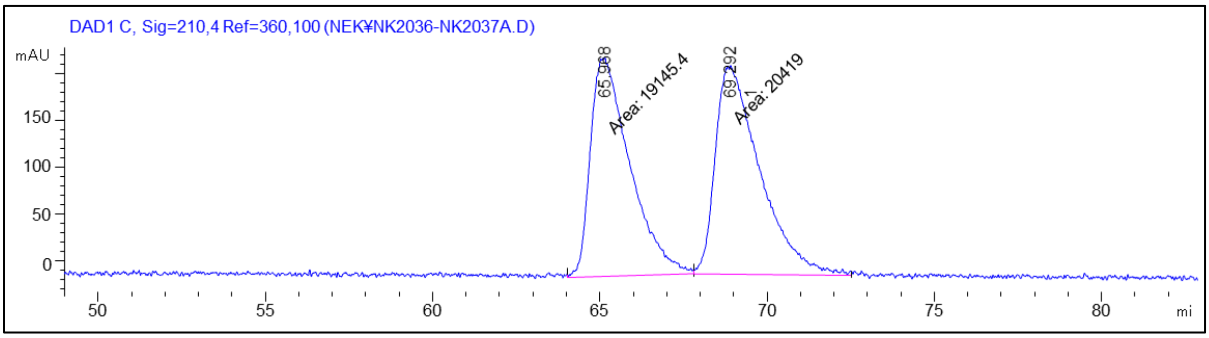

Peak #	RetTime [min]	Type	Width [min]	Area [mAU*s]	Height [mAU]	Area %
1	65.968	MM	1.3657	1.91454e4	233.64494	48.3905
2	69.292	MM	1.5270	2.04190e4	222.87083	51.6095

Figure S48. (3*R*,1*E*)-(3-(2-(1,3-Dioxolan-2-yl)ethyl)-2-methylhept-1-en-1-yl)(methyl) diphenylsilane [(*R*)-**8da**].

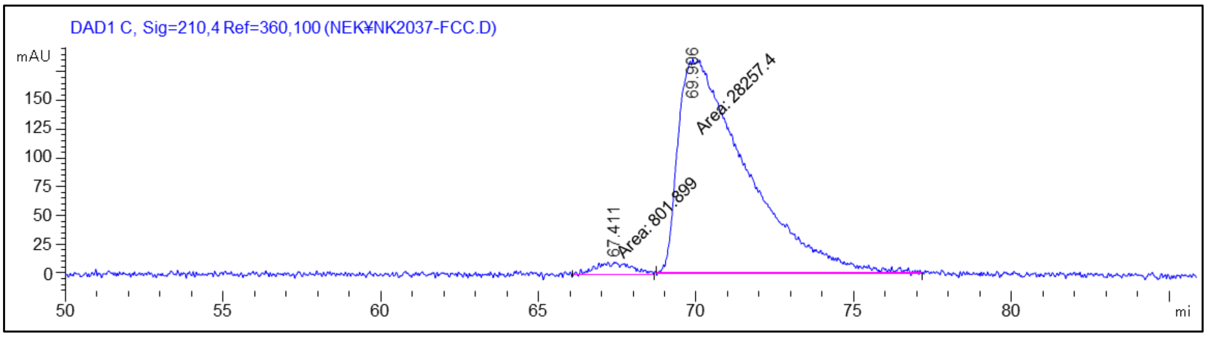

Peak #	RetTime [min]	Type	Width [min]	Area [mAU*s]	Height [mAU]	Area %
1	67.411	MM	1.2471	801.89935	10.71684	2.7595
2	69.906	MM	2.5096	2.82574e4	187.66084	97.2405

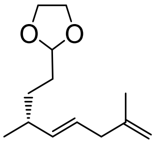

14aaa

(2*R*,4*E*)-2-(3,7-Dimethylocta-4,7-dien-1-yl)-1,3-dioxolane (**14aaa**): The enantiomeric ratio of **14aaa** was determined by HPLC analysis on a chiral stationary phase (*Daicel* Chiralcel OD-H column, column temperature 20 °C, solvent *n*-heptane:isopropanol = 99.9:0.1, flow rate 0.4 mL/min): t_R = 28.8 min (minor), t_R = 33.8 min (major).

Figure S49. *rac*-(4*E*)-2-(3,7-Dimethylocta-4,7-dien-1-yl)-1,3-dioxolane (*rac*-**14aaa**).

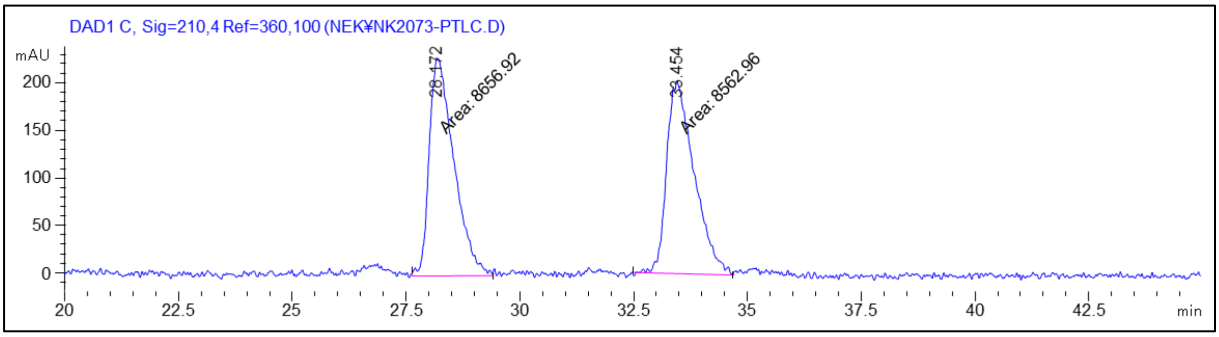

Peak #	RetTime [min]	Type	Width [min]	Area [mAU*s]	Height [mAU]	Area %
1	28.172	MM	0.6296	8656.91504	229.14699	50.2728
2	33.454	MM	0.7025	8562.96191	203.16307	49.7272

Figure S50. (2*R*,4*E*)-2-(3,7-Dimethylocta-4,7-dien-1-yl)-1,3-dioxolane [(*R*)-**14aaa**].

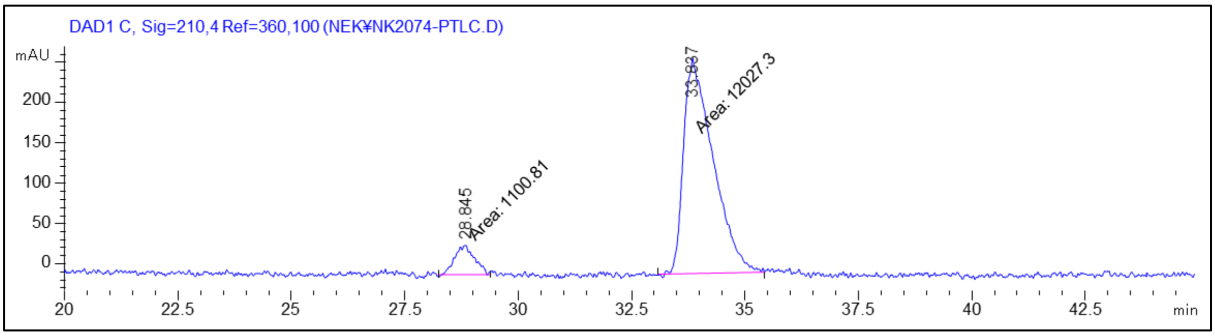

Peak #	RetTime [min]	Type	Width [min]	Area [mAU*s]	Height [mAU]	Area %
1	28.845	MM	0.5032	1100.80688	36.46365	8.3851
2	33.837	MM	0.7510	1.20273e4	266.92227	91.6149

10. GLC Traces

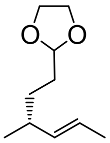

14aab

(2*R*,4*E*)-2-(3-Methylhex-4-en-1-yl)-1,3-dioxolane (**14aab**): The enantiomeric ratio of **14aab** was determined by chiral GLC analysis (*Varian* CP-Chirasil-Dex *CB* column (25 m × 0.25 mm, 0.25 µm film thickness), column temperature 60 °C (isothermal), flow rate 1.0 mL/min): t_R = 132.5 min (minor), t_R = 135.4 min (major).

Figure S51. *rac*-(4*E*)-2-(3-Methylhex-4-en-1-yl)-1,3-dioxolane (*rac*-**14aab**).

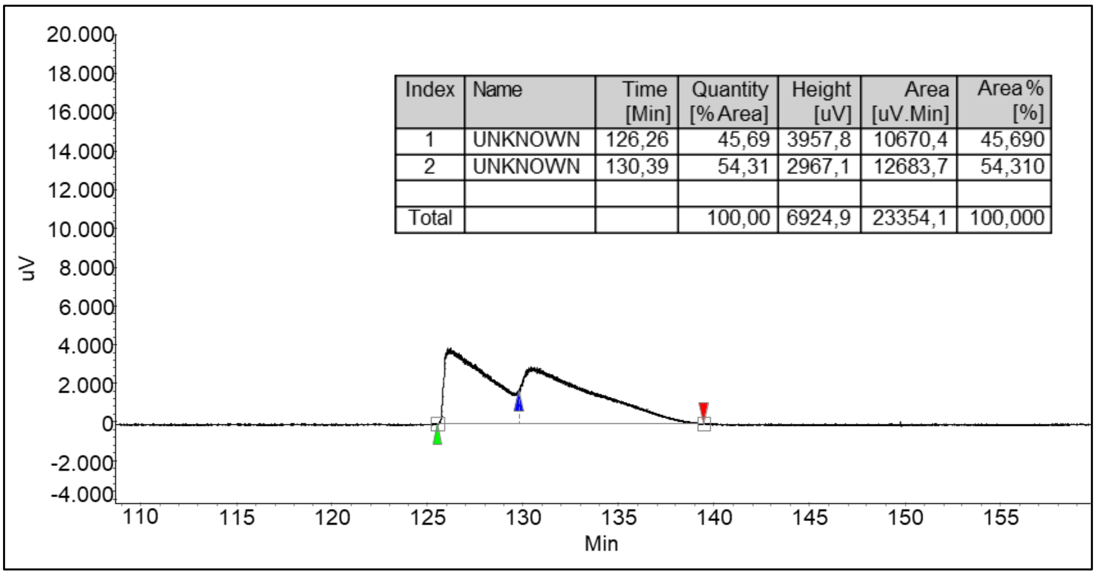

Index	Name	Time [Min]	Quantity [% Area]	Height [uV]	Area [uV.Min]	Area % [%]
1	UNKNOWN	126,26	45,69	3957,8	10670,4	45,690
2	UNKNOWN	130,39	54,31	2967,1	12683,7	54,310
Total			100,00	6924,9	23354,1	100,000

Figure S52. (2*R*,4*E*)-2-(3-Methylhex-4-en-1-yl)-1,3-dioxolane [(*R*)-**14aab**].

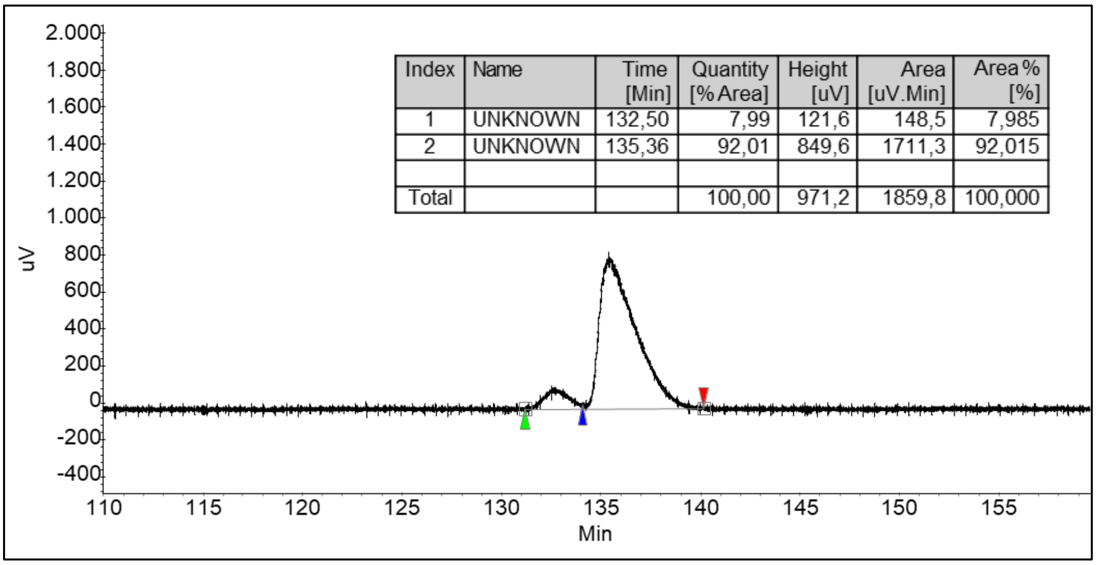

Index	Name	Time [Min]	Quantity [% Area]	Height [uV]	Area [uV.Min]	Area % [%]
1	UNKNOWN	132,50	7,99	121,6	148,5	7,985
2	UNKNOWN	135,36	92,01	849,6	1711,3	92,015
Total			100,00	971,2	1859,8	100,000

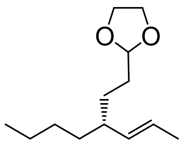

14bab

(2*R*,4*E*)-2-(3-(Prop-1-en-1-yl)heptyl)-1,3-dioxolane (**14bab**): The enantiomeric ratio of **14bab** was determined by chiral GLC analysis (*Sigma-Aldrich* Astec Chiraldex B-DM column (30 m × 0.25 mm, 0.12 µm film thickness), column temperature 80 °C (200 min), ramp of 2 °C/ min to 200 °C (10 min), flow rate 1.0 mL/min): t_R = 220.7 min (major), t_R = 221.5 min (minor).

Figure S53. *rac*-(4*E*)-2-(3-(Prop-1-en-1-yl)heptyl)-1,3-dioxolane (*rac*-**14bab**).

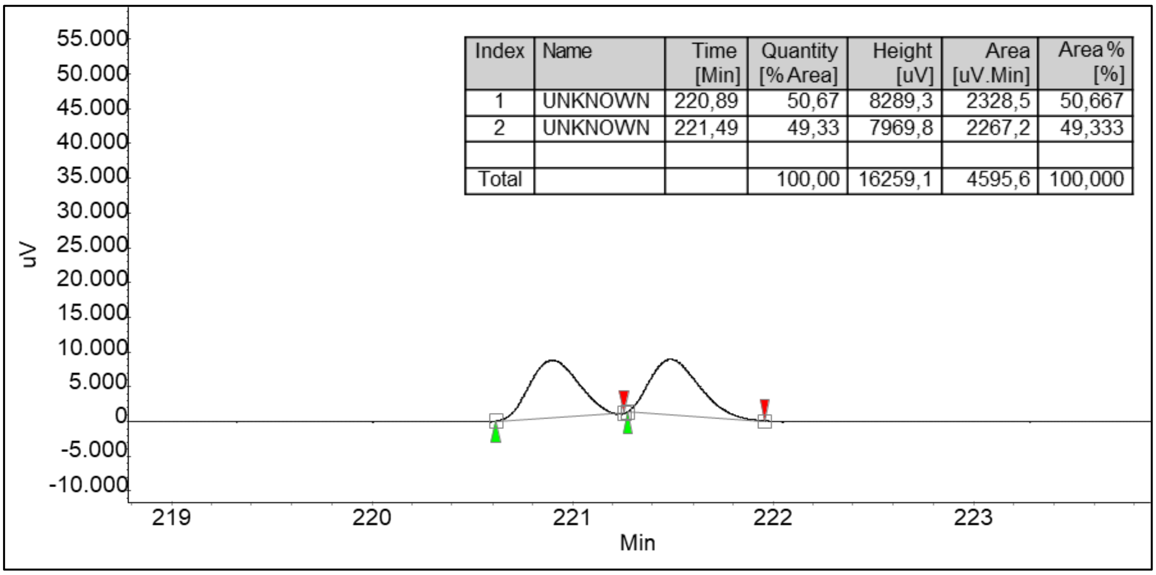

Index	Name	Time [Min]	Quantity [% Area]	Height [uV]	Area [uV.Min]	Area % [%]
1	UNKNOWN	220,89	50,67	8289,3	2328,5	50,667
2	UNKNOWN	221,49	49,33	7969,8	2267,2	49,333
Total			100,00	16259,1	4595,6	100,000

Figure S54. (2*R*,4*E*)-2-(3-(Prop-1-en-1-yl)heptyl)-1,3-dioxolane [(*R*)-**14bab**].

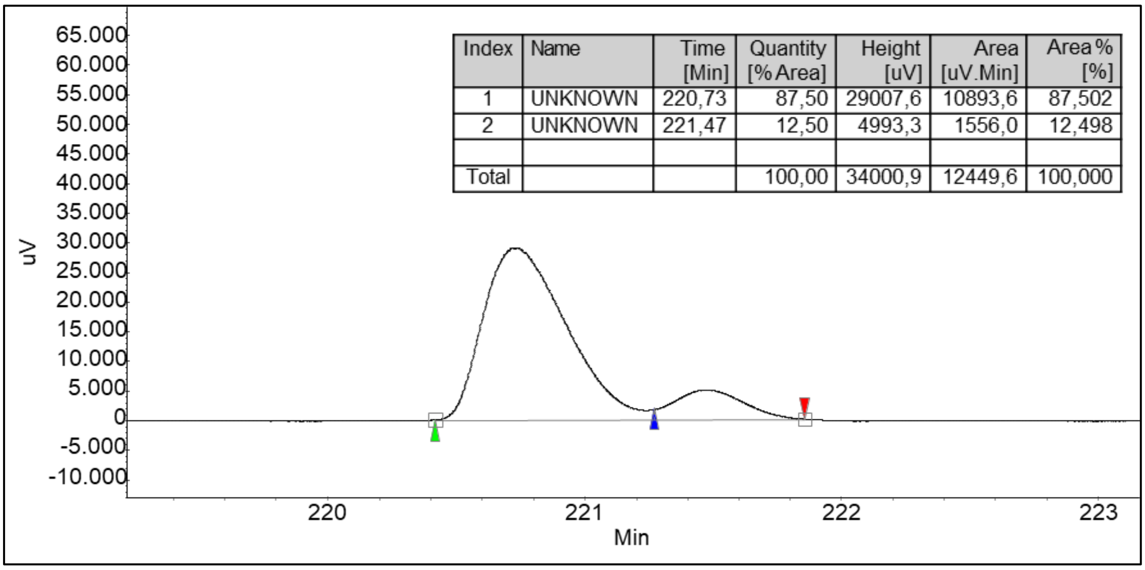

Index	Name	Time [Min]	Quantity [% Area]	Height [uV]	Area [uV.Min]	Area % [%]
1	UNKNOWN	220,73	87,50	29007,6	10893,6	87,502
2	UNKNOWN	221,47	12,50	4993,3	1556,0	12,498
Total			100,00	34000,9	12449,6	100,000

11. NMR Spectra

(2*E*)-(1-Bromobut-2-en-1-yl)dimethyl(phenyl)silane & **(1*E*)-(3-bromobut-2-en-1-yl)dimethyl(phenyl)silane** (**1a**):

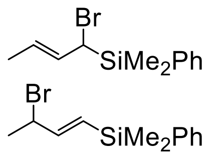

α:γ = 53:47

1a

Figure S55. ^{1}H NMR (500 MHz, $CDCl_3$, 298 K) of **1a**.

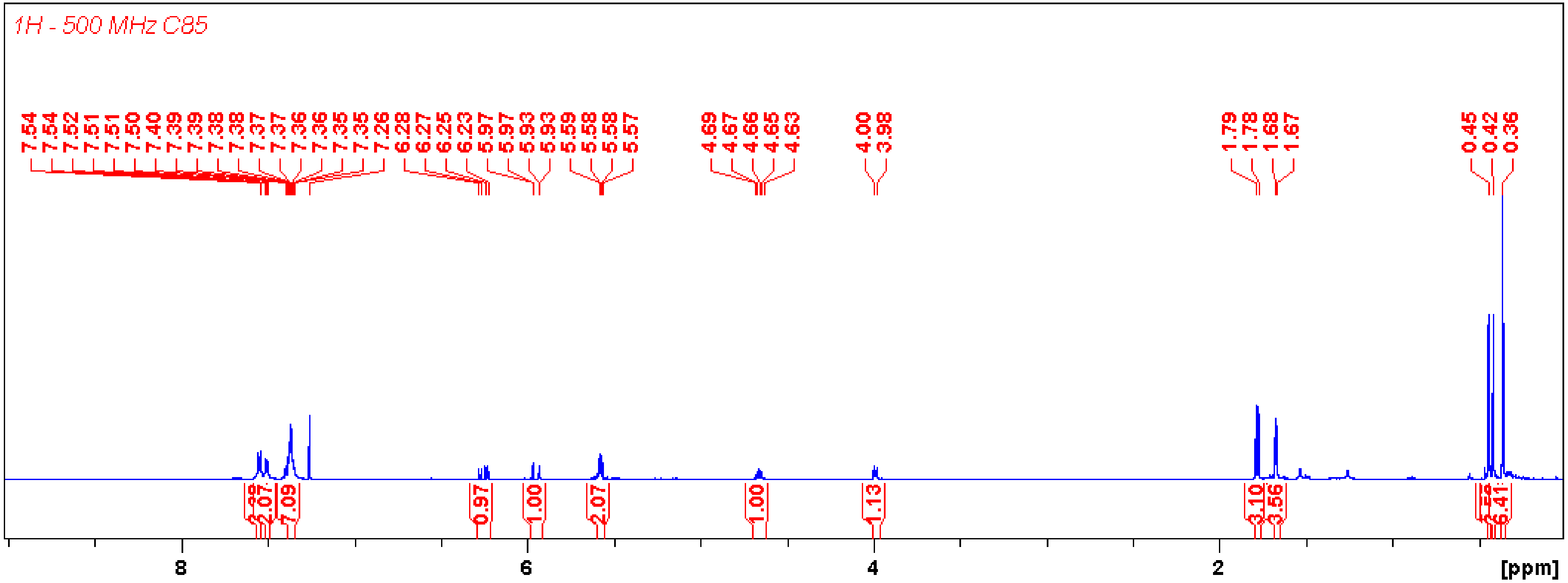

Figure S56. ^{13}C NMR (126 MHz, $CDCl_3$, 298 K) of **1a**.

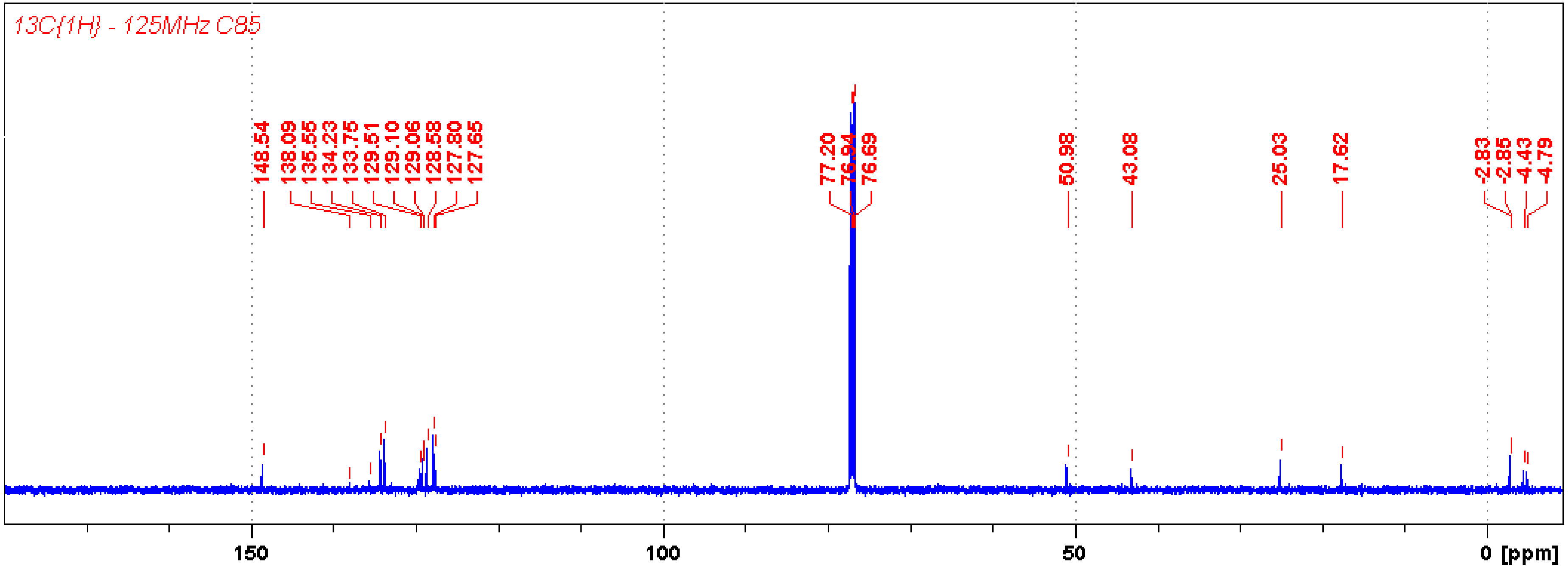

Figure S57. ^{29}Si DEPT NMR (99 MHz, $CDCl_3$, 298 K) of **1a**.

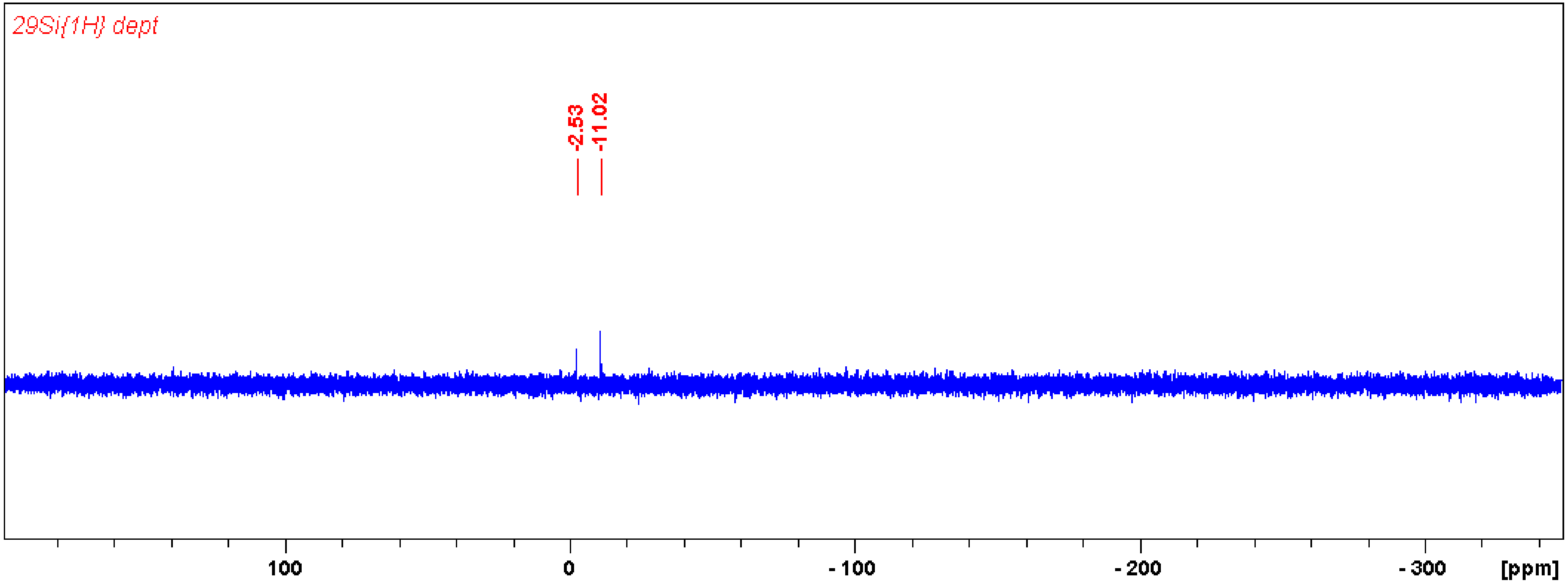

(2*E*)-(1-Bromobut-2-en-1-yl) (methyl) & (1*E*)-(3-bromobut-2-en-1-yl)(methyl)phenylsilane (4a):

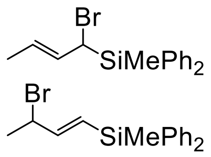

α:γ = 30:70

4a

Figure S58. ^{1}H NMR (500 MHz, $CDCl_3$, 298 K) of **4a**.

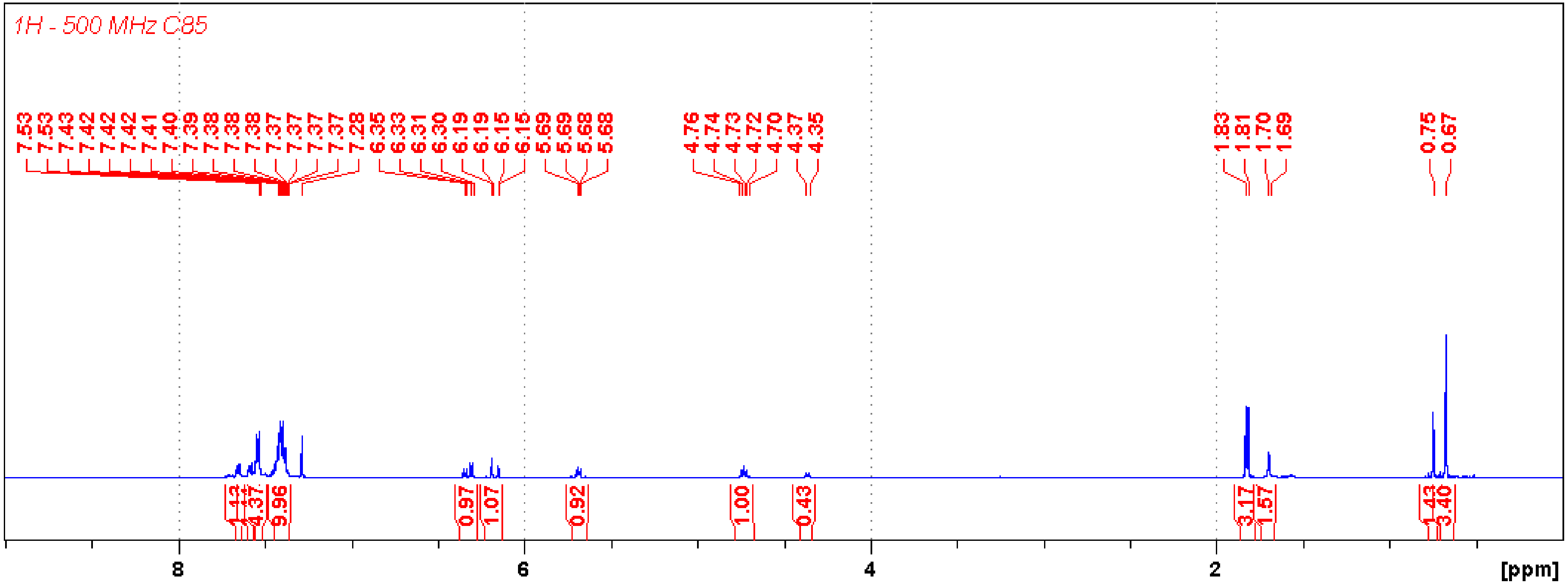

Figure S59. ^{13}C NMR (126 MHz, $CDCl_3$, 298 K) of **4a**.

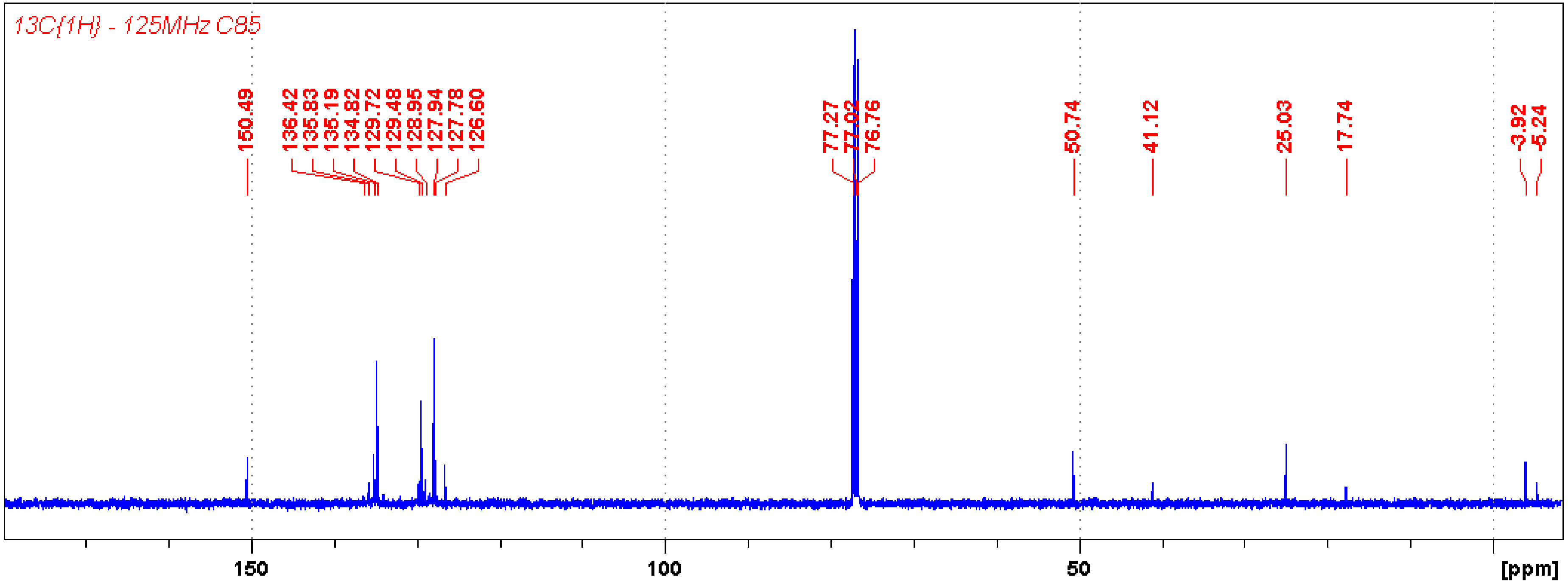

Figure S60. ^{29}Si DEPT NMR (99 MHz, $CDCl_3$, 298 K) of **4a**.

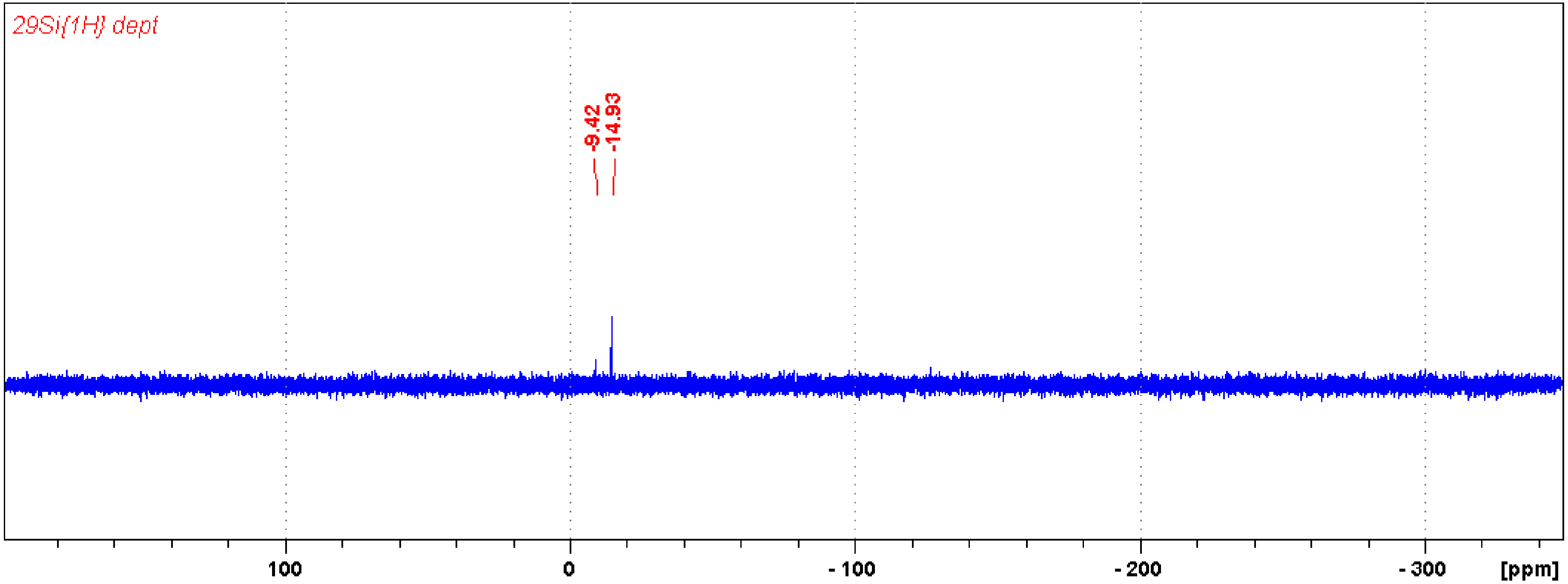

(2*E*)-(1-Bromohex-1-en-1-yl)(methyl)diphenylsilane & **(1*E*)-(3-bromohex-1-en-1-yl)(methyl)diphenylsilane** (**4b**):

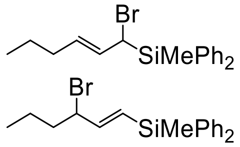

α:γ =23:77

4b

Figure S61. ^{1}H NMR (500 MHz, $CDCl_3$, 298 K) of **4b**.

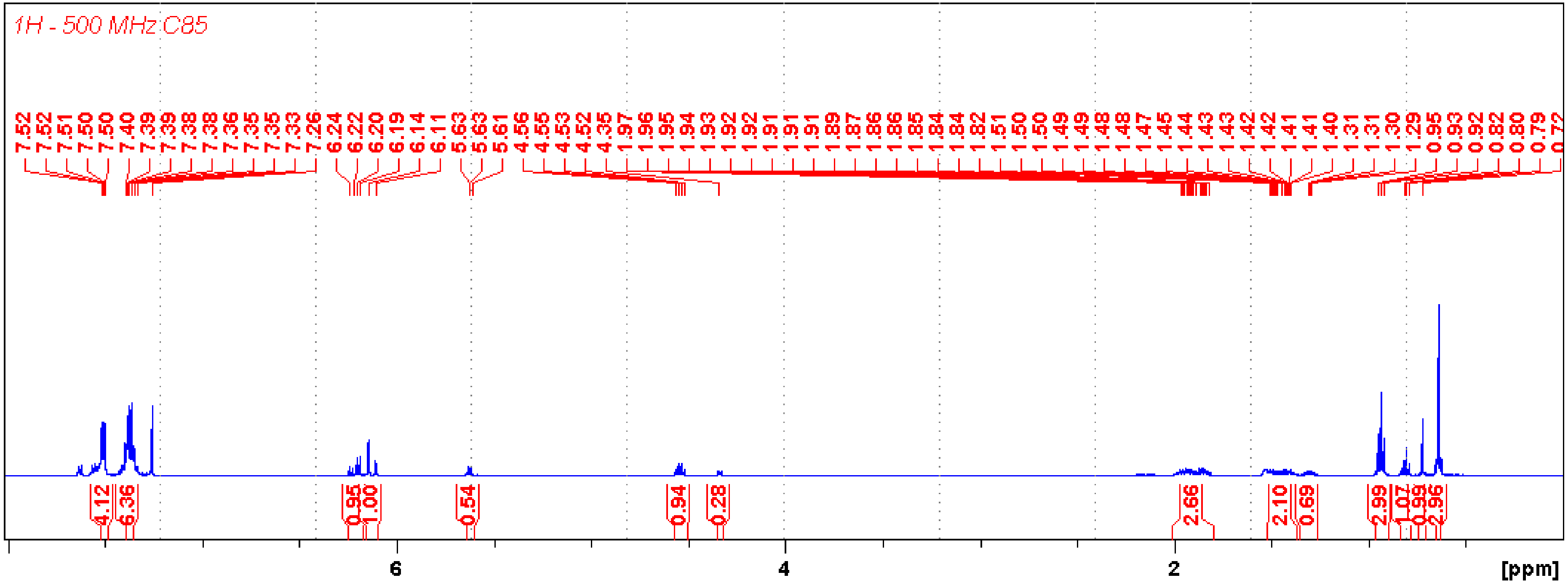

Figure S62. ^{13}C NMR (126 MHz, $CDCl_3$, 298 K) of **4b**.

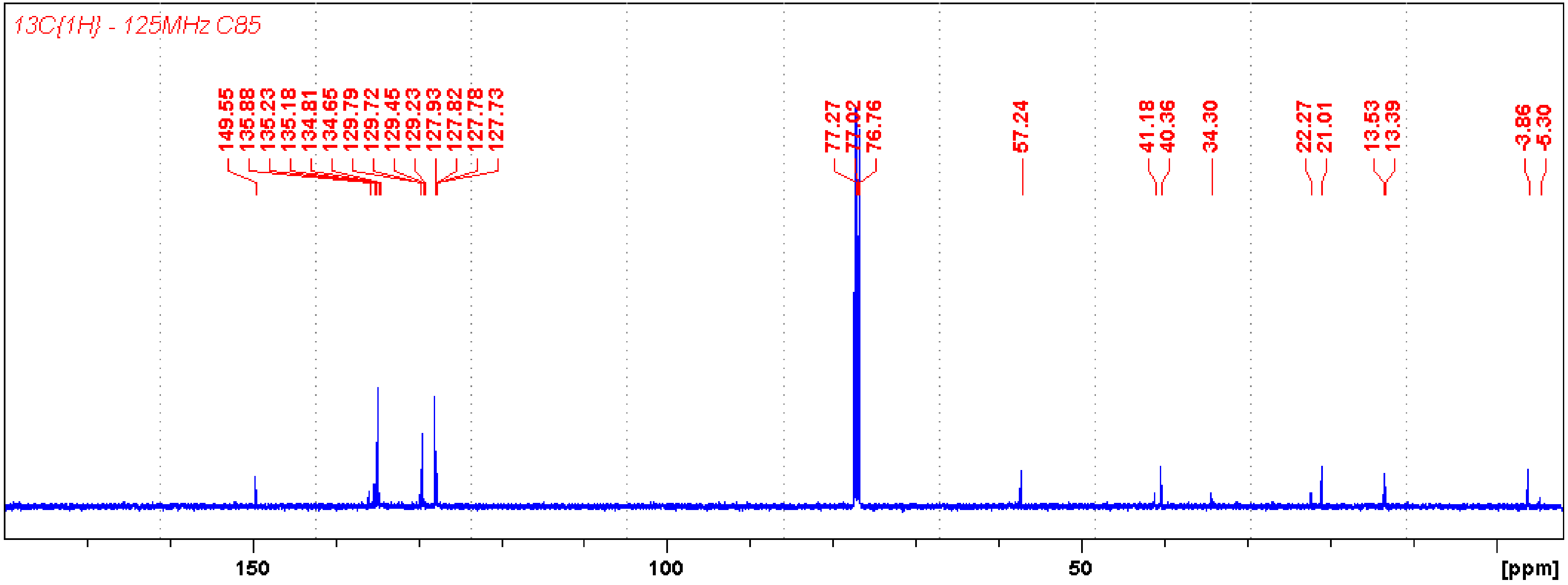

Figure S63. ^{29}Si DEPT NMR (99 MHz, $CDCl_3$, 298 K) of **4b**.

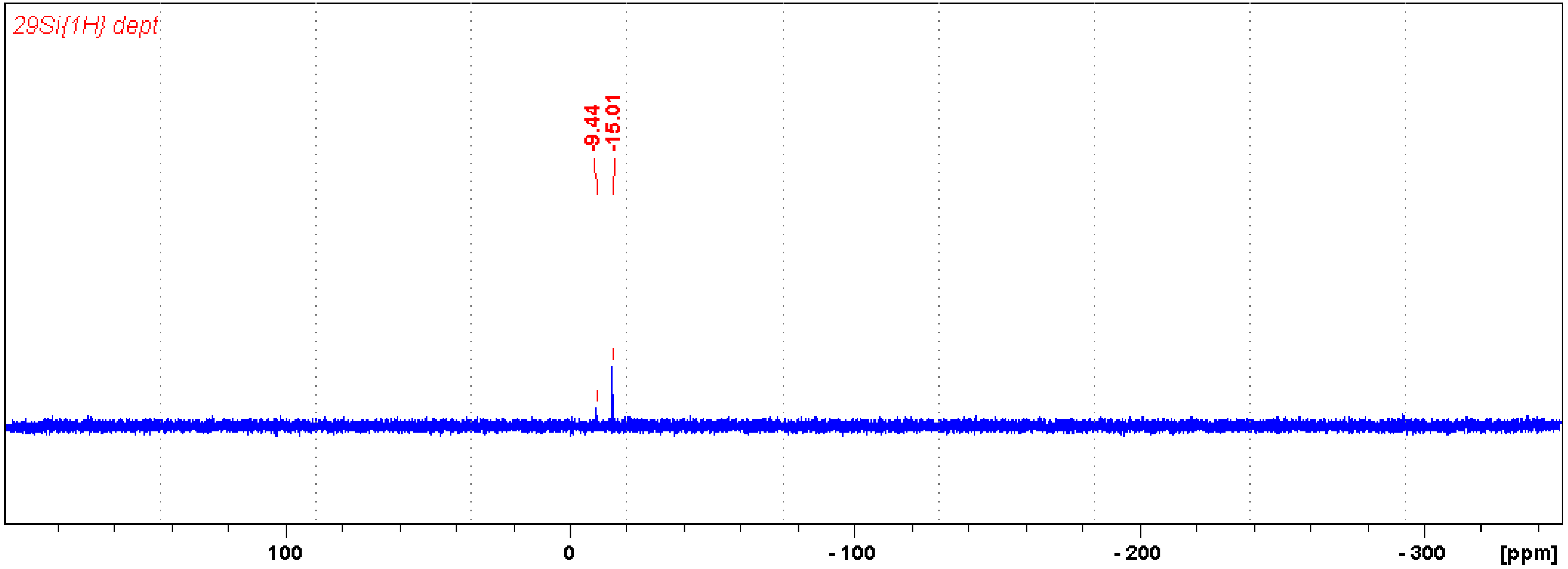

(1*E*)-(3-Bromobut-1-en-1-yl)(*tert*-butyl)diphenylsilane (**5a**):

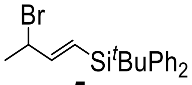

5a

Figure S64. ^{1}H NMR (500 MHz, $CDCl_3$, 298 K) of **5a**.

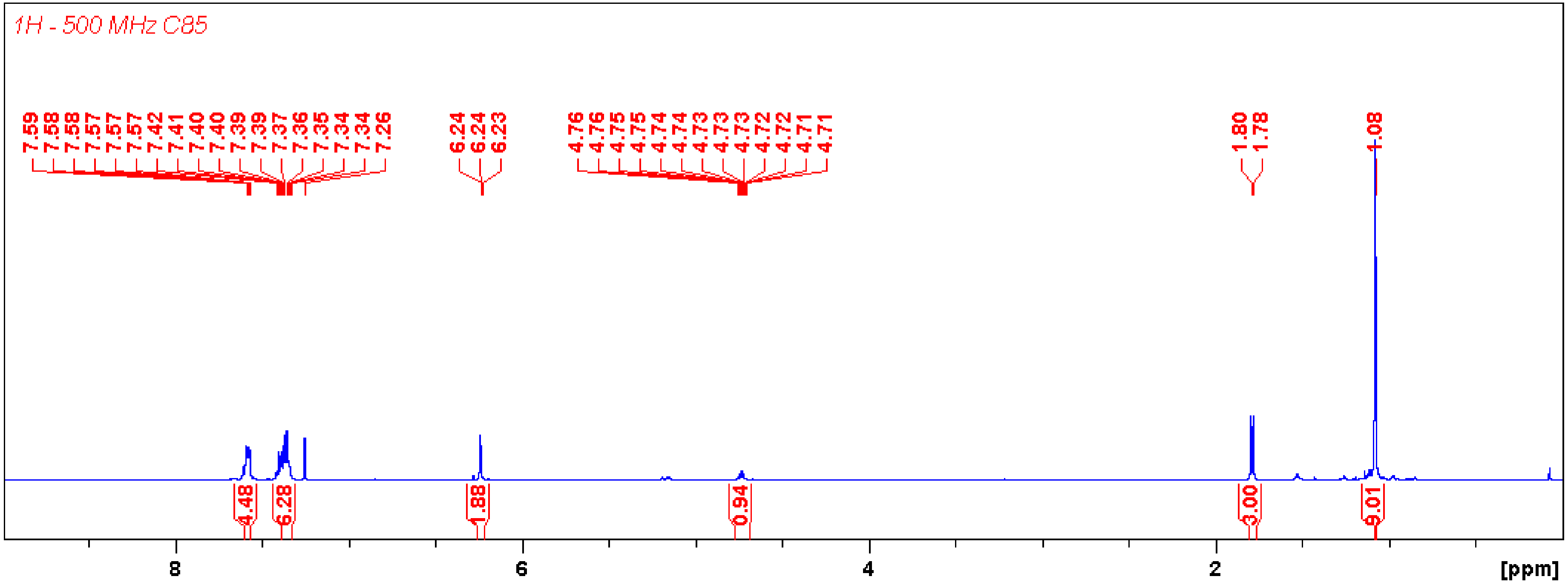

Figure S65. ^{13}C NMR (126 MHz, $CDCl_3$, 298 K) of **5a**.

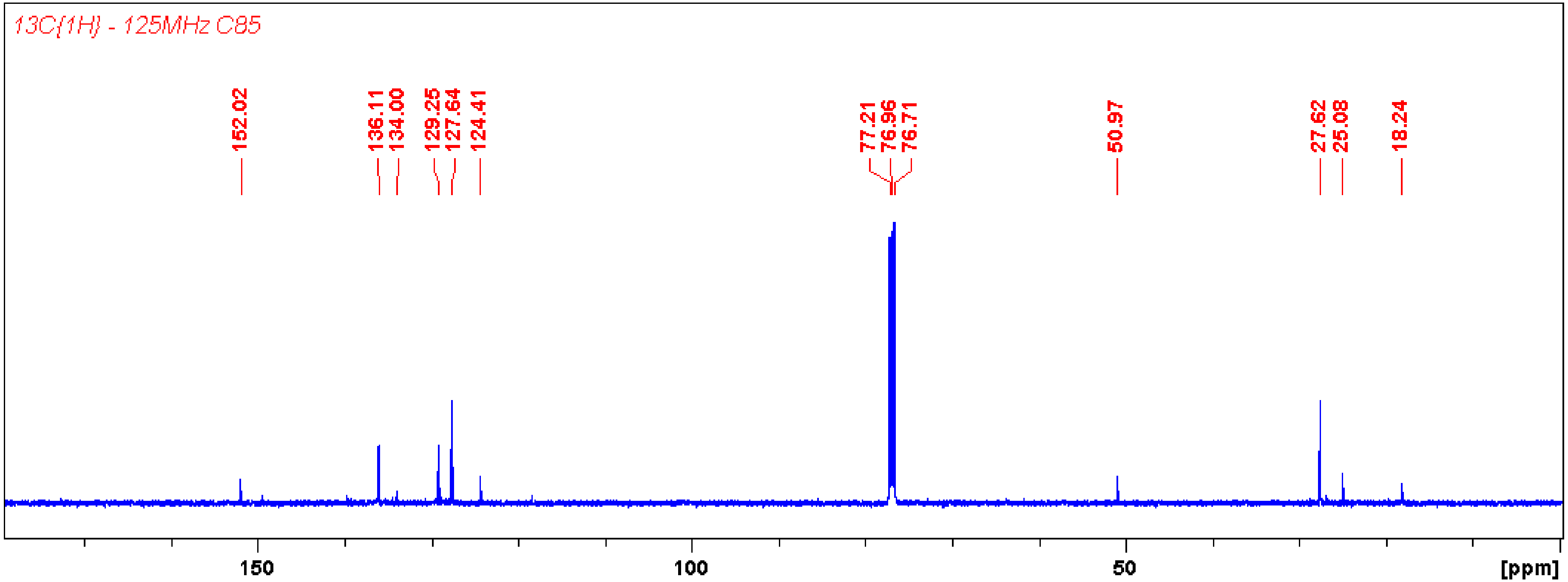

Figure S66. ^{29}Si DEPT NMR (99 MHz, $CDCl_3$, 298 K) of 5**a**.

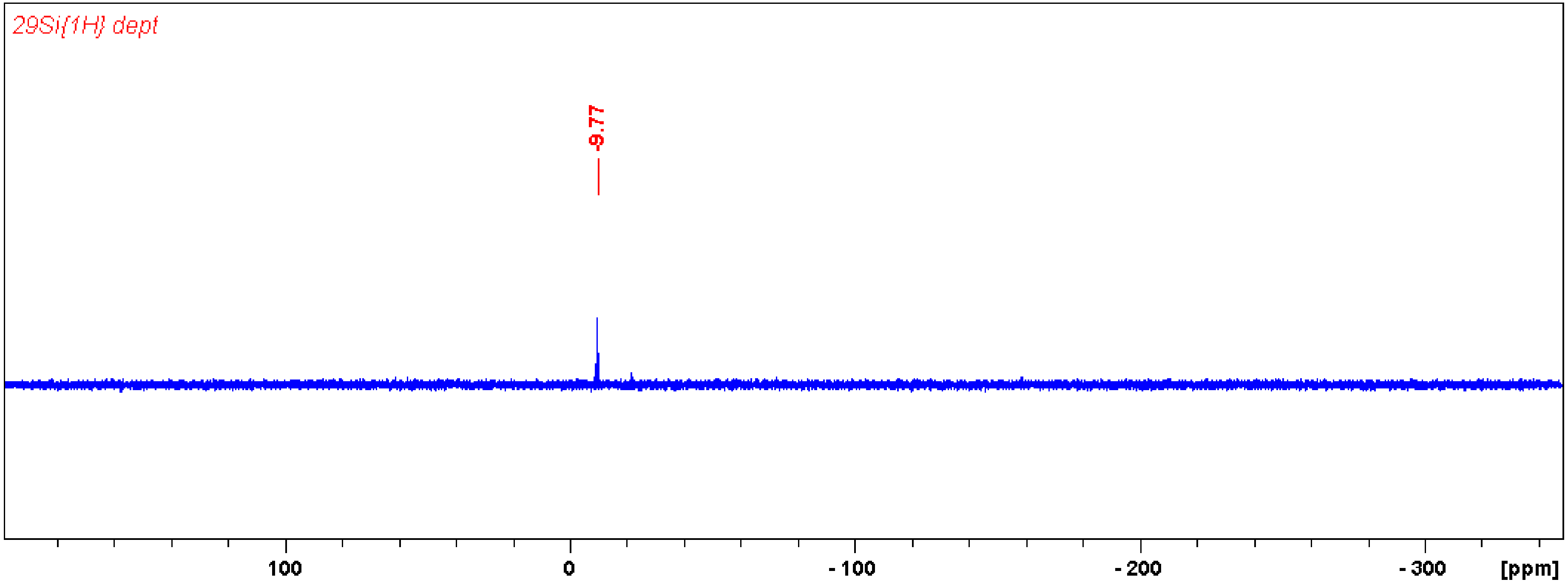

(2*E*)-(1-Bromobut-2-en-1-yl)trimethylsilane & **(1*E*)-(3-bromobut-2-en-1-yl)trimethylsilane** (**6a**):

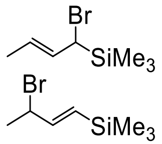

α:γ = 60:40

6a

Figure S67. ^{1}H NMR (500 MHz, $CDCl_3$, 298 K) of **6a**.

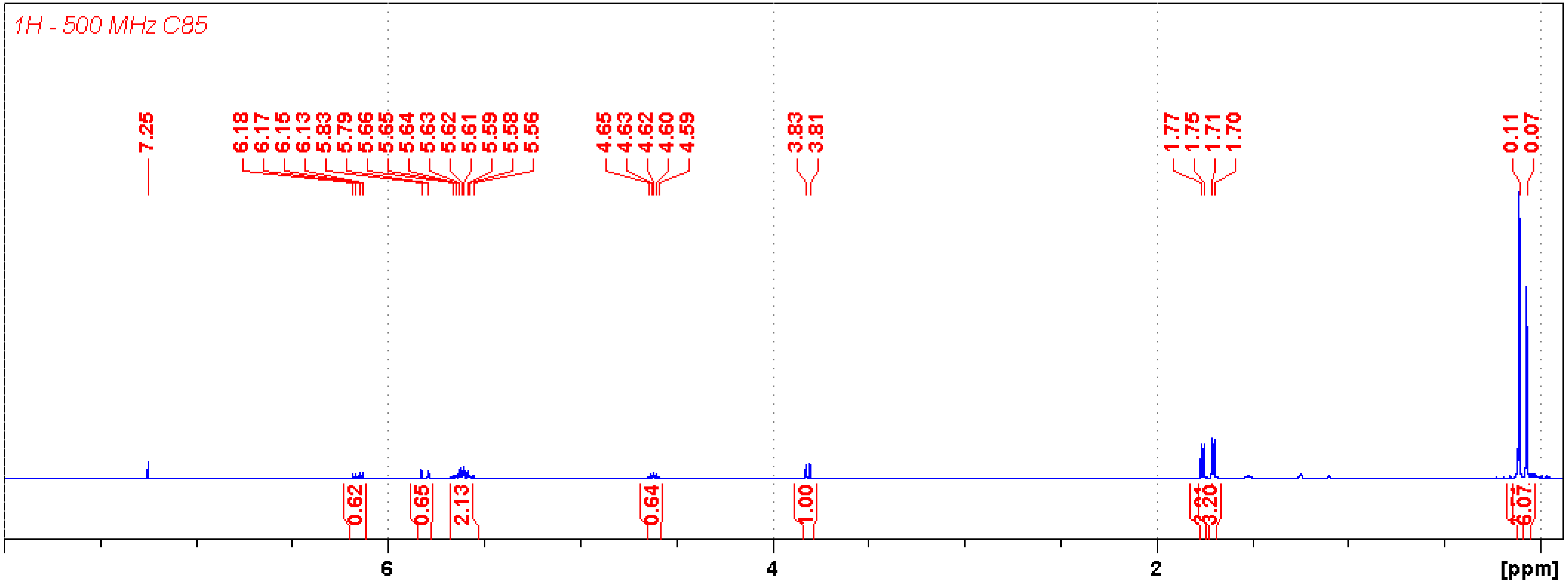

Figure S68. ^{13}C NMR (126 MHz, $CDCl_3$, 298 K) of **6a**.

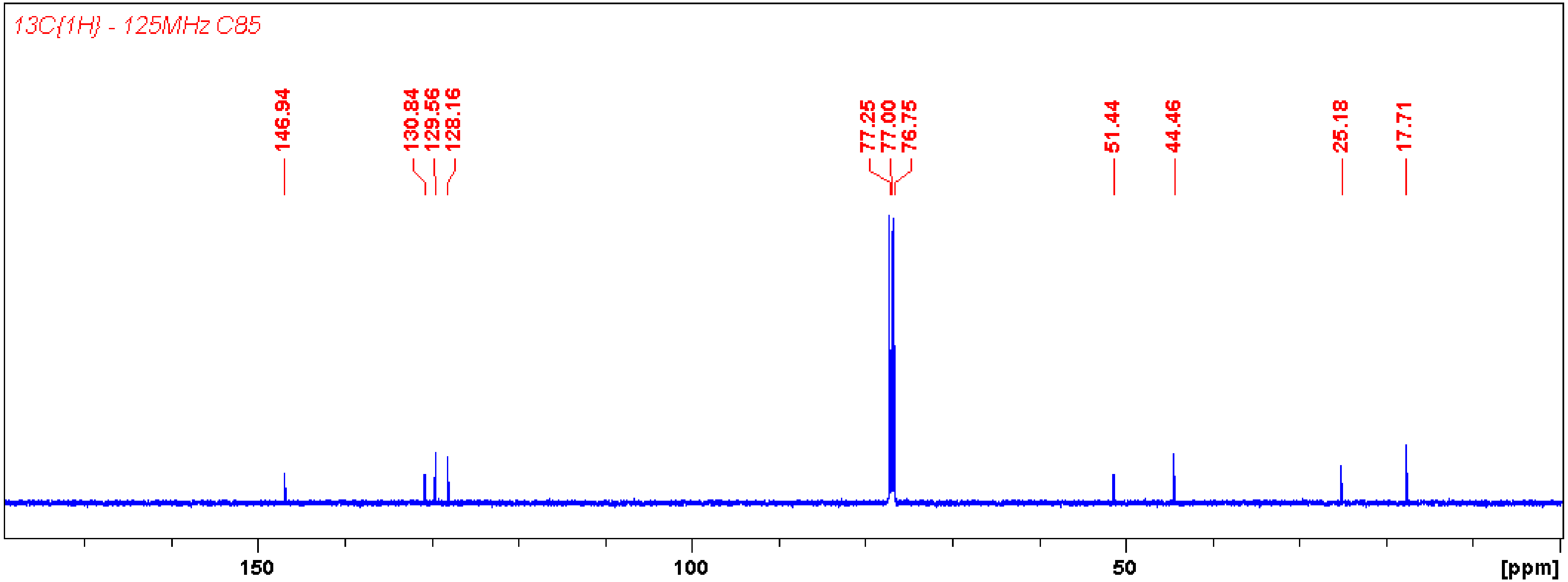

Figure S69. ^{29}Si DEPT NMR (99 MHz, $CDCl_3$, 298 K) of **6a**.

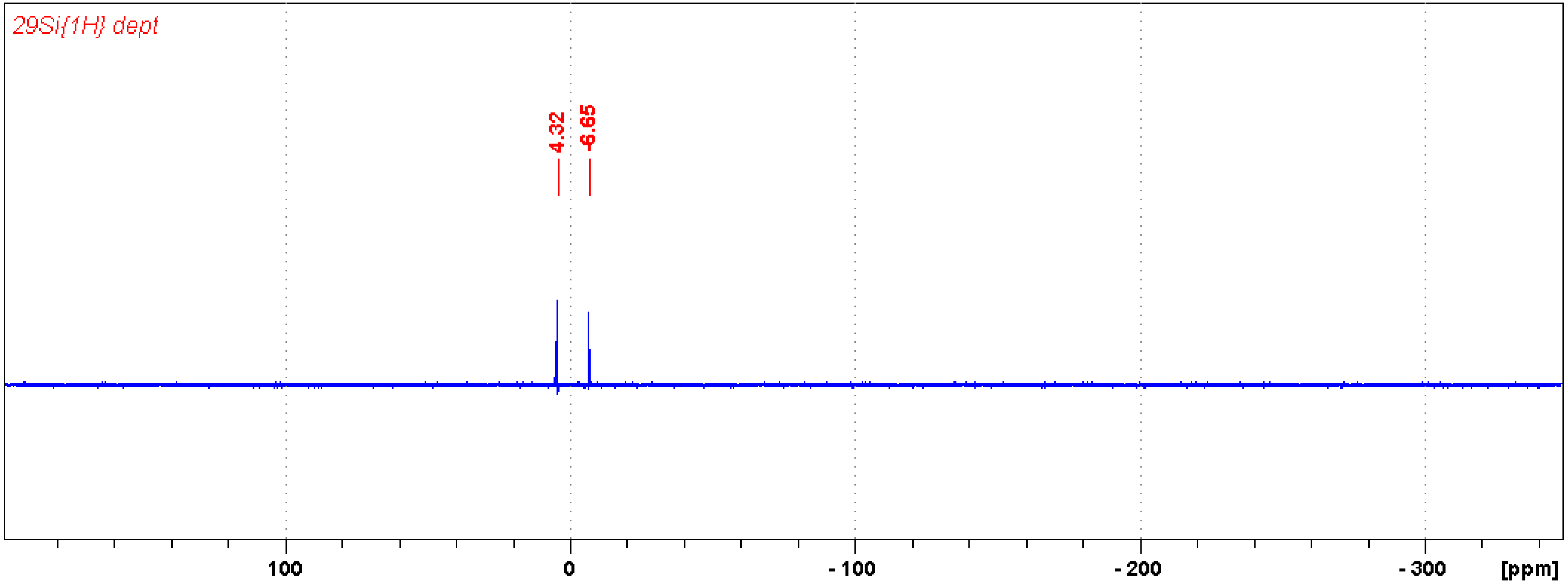

(2*E*)-benzyl(1-bromobut-2-en-1-yl)dimethylsilane & **(1*E*)-benzyl(1-bromobut-2-en-1-yl)dimethylsilane** (**7a**):

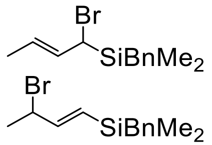

7a

Figure S70. ^{1}H NMR (500 MHz, $CDCl_3$, 298 K) of **7a**.

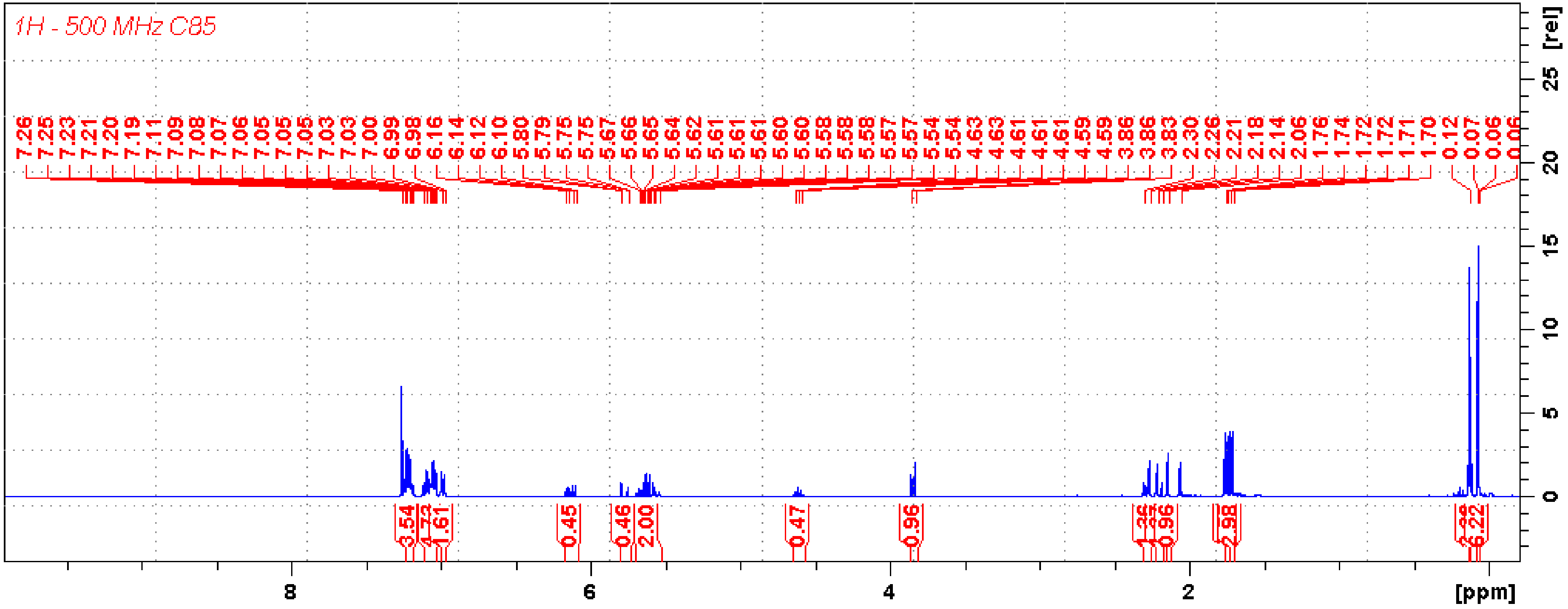

Figure S71. ^{13}C NMR (126 MHz, $CDCl_3$, 298 K) of **7a**.

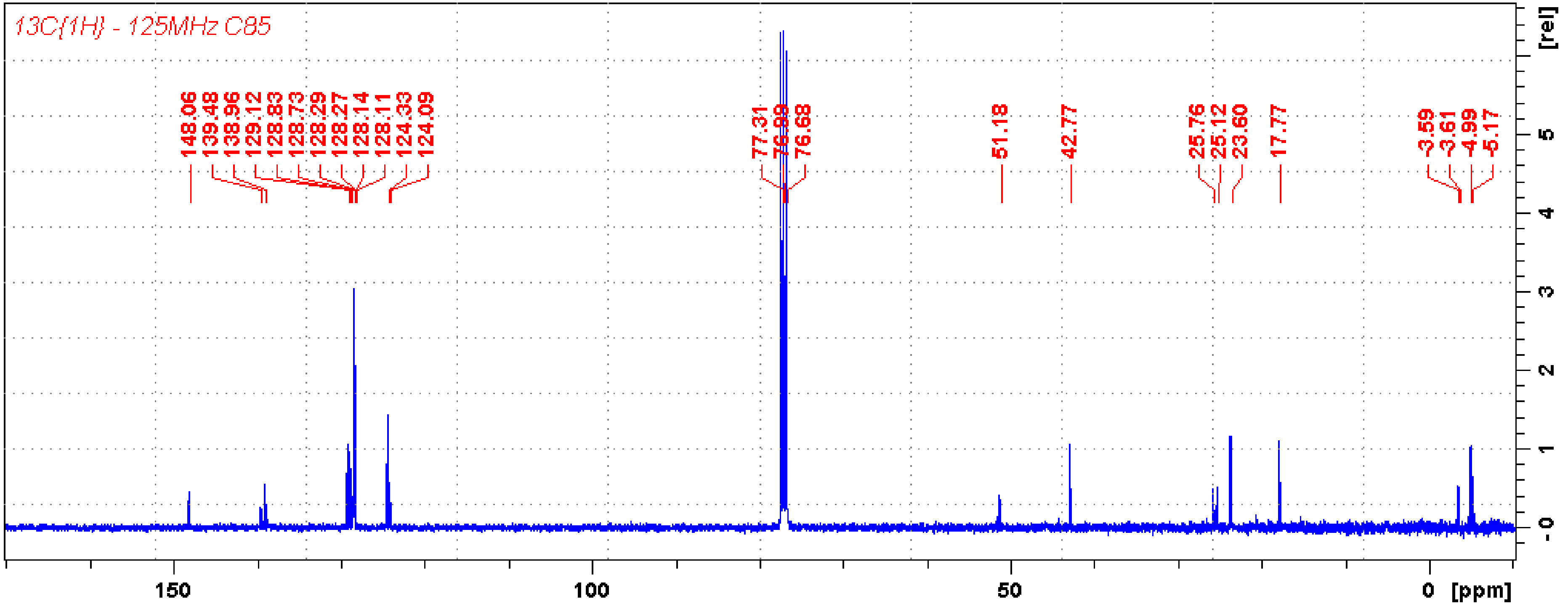

Figure S72. ^{29}Si DEPT NMR (99 MHz, $CDCl_3$, 298 K) of **7a**.

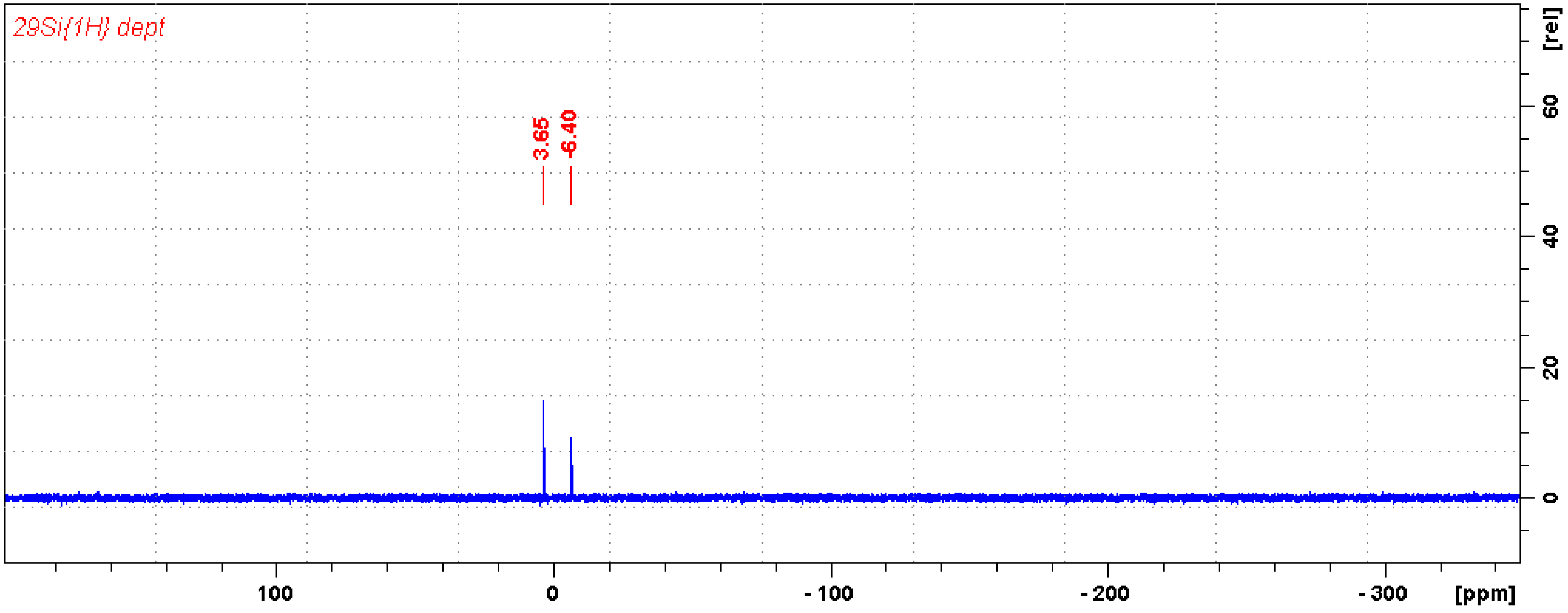

(2*E*)-Benzyl(1-bromohept-2-en-1-yl)dimethylsilane & ***rac*-(1*E*)-benzyl(3-bromohept-1-en-1-yl)dimethylsilane** (**7b**):

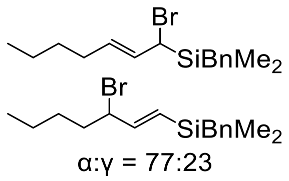

7b

Figure S73. ^{1}H NMR (500 MHz, $CDCl_3$, 298 K) of **7b**.

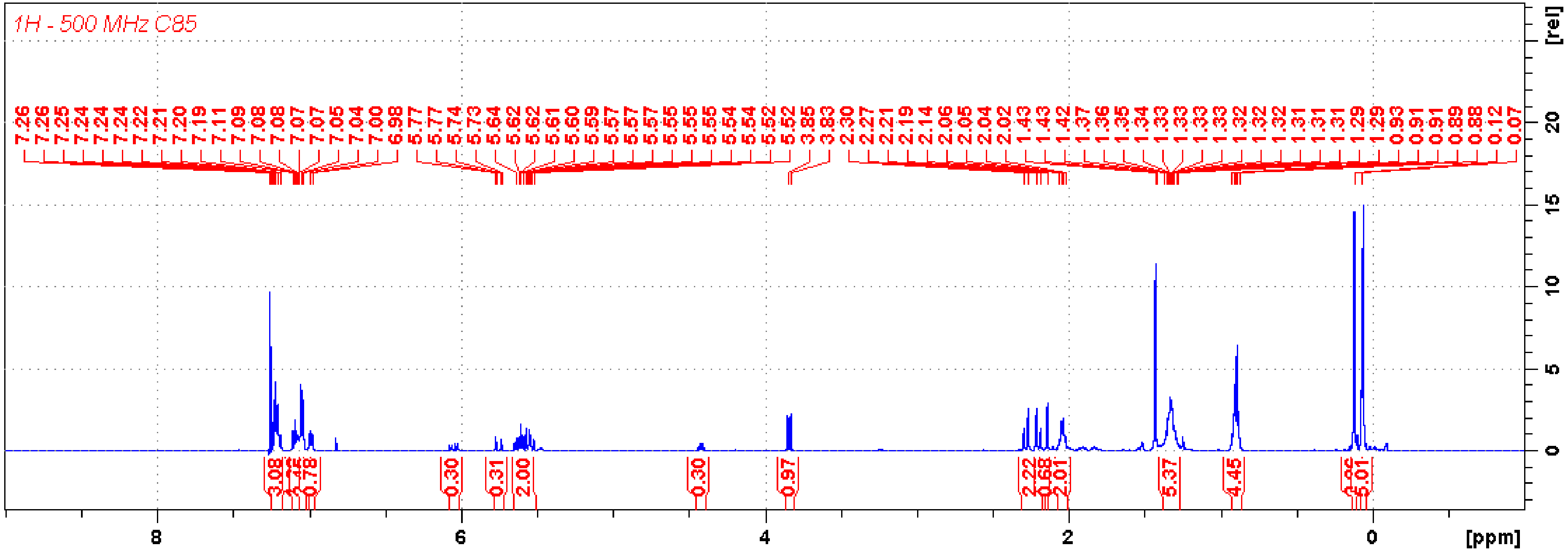

Figure S74. ^{13}C NMR (126 MHz, $CDCl_3$, 298 K) of **7b**.

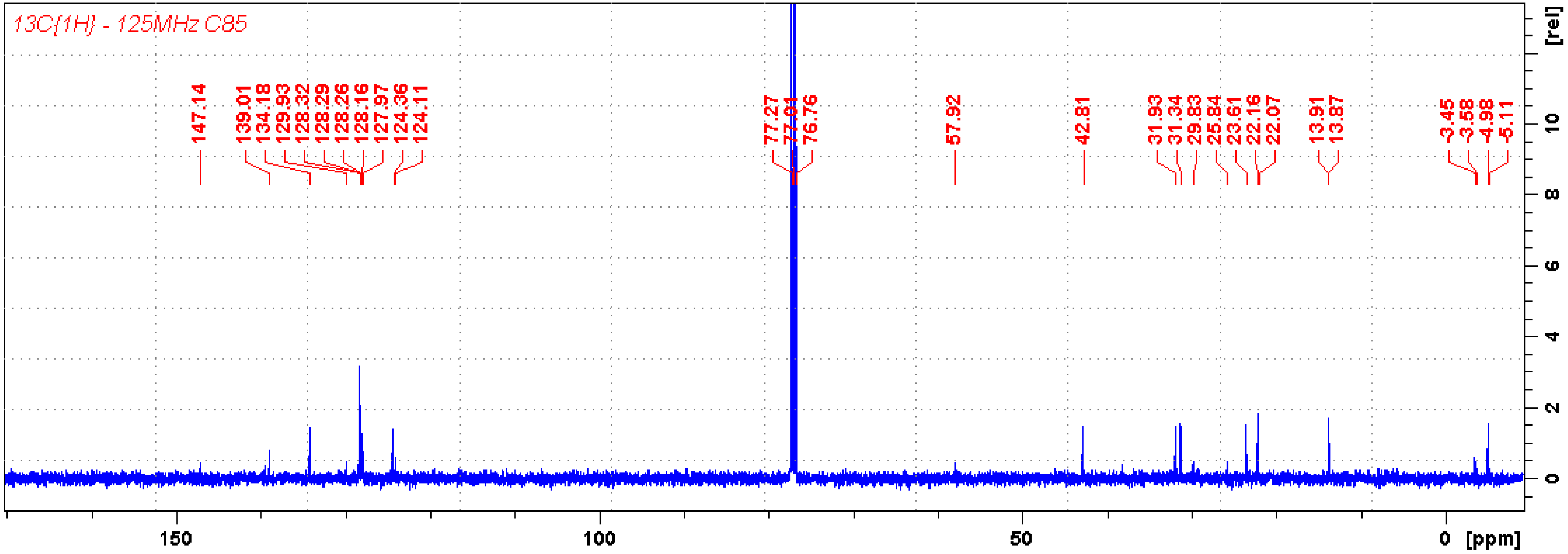

Figure S75. ^{29}Si DEPT NMR (99 MHz, $CDCl_3$, 298 K) of **7b**.

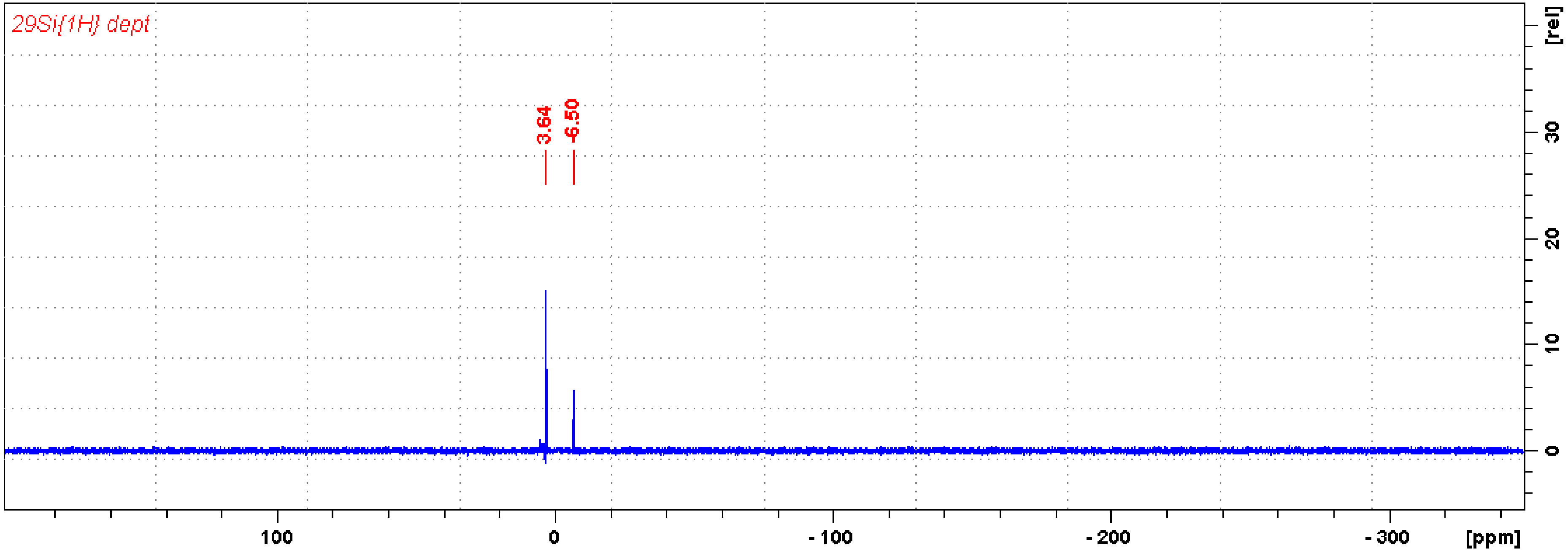

(1*E*)-(3-Chlorobut-1-en-1-yl)dimethyl(phenyl)silane (**S13a**):

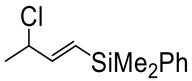

S13a

Figure S76. ^{1}H NMR (500 MHz, $CDCl_3$, 298 K) of **S13a**.

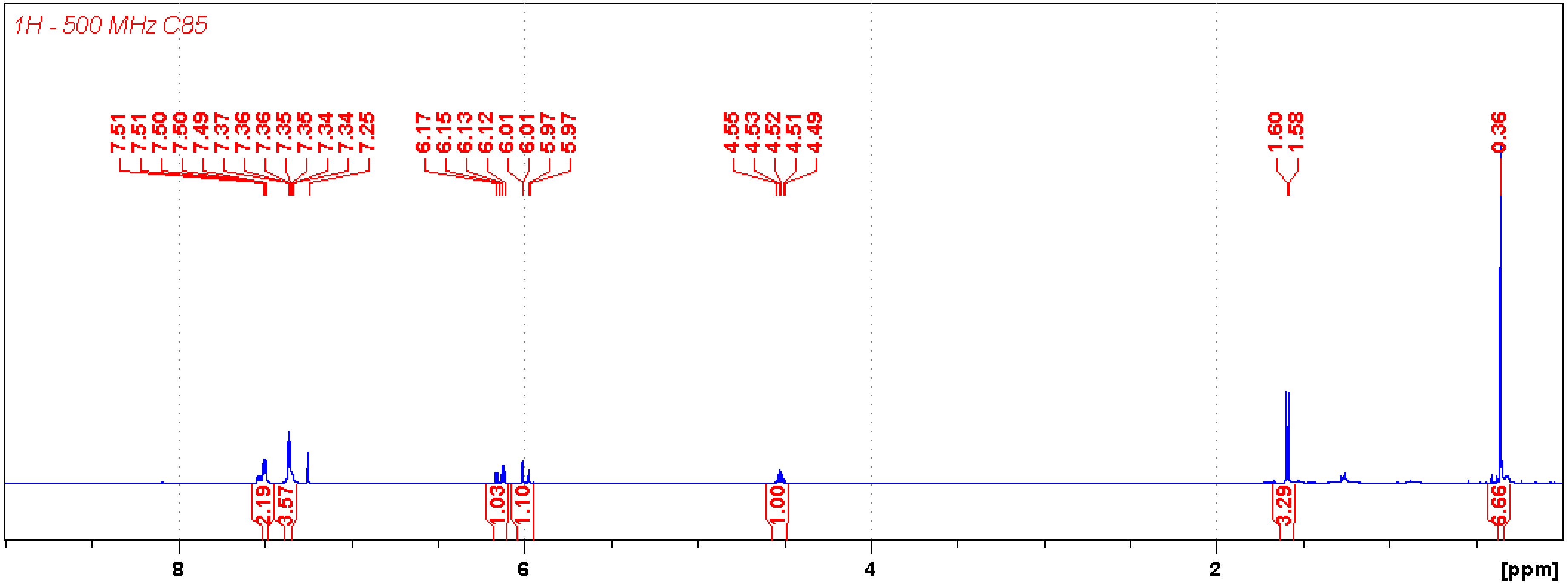

Figure S77. ^{13}C NMR (126 MHz, $CDCl_3$, 298 K) of **S13a**.

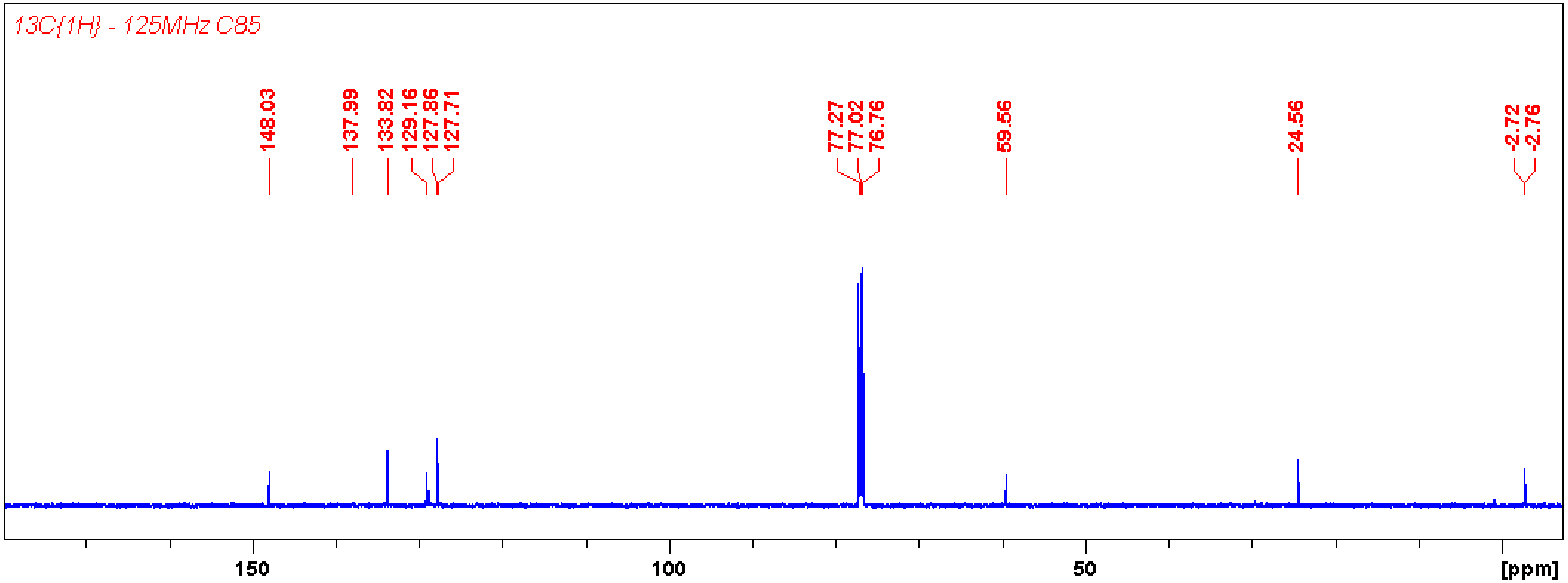

Figure S78. ^{29}Si DEPT NMR (99 MHz, $CDCl_3$, 298 K) of **S13a**.

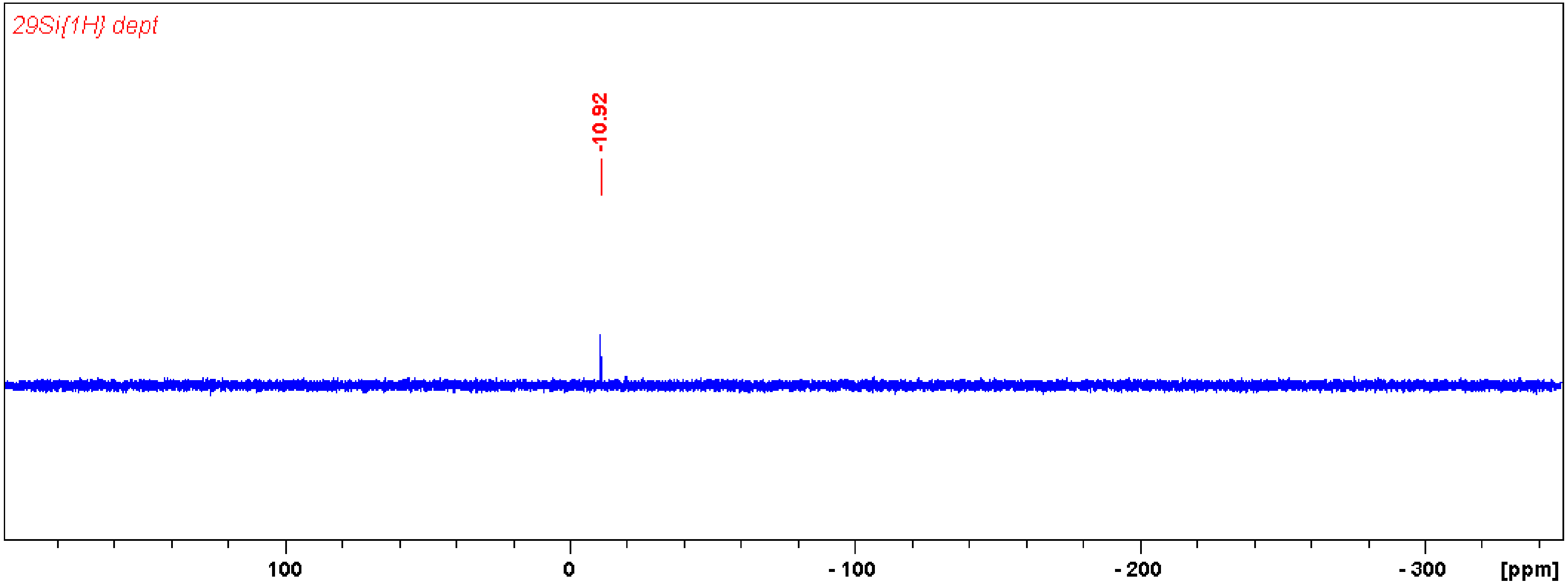

(1*E*)-(3-Acetoxy-1-en-1-yl)dimethyl(phenyl)silane (S14a):

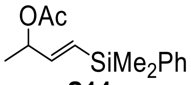

Figure S79. ^{1}H NMR (500 MHz, $CDCl_3$, 298 K) of **S14a**.

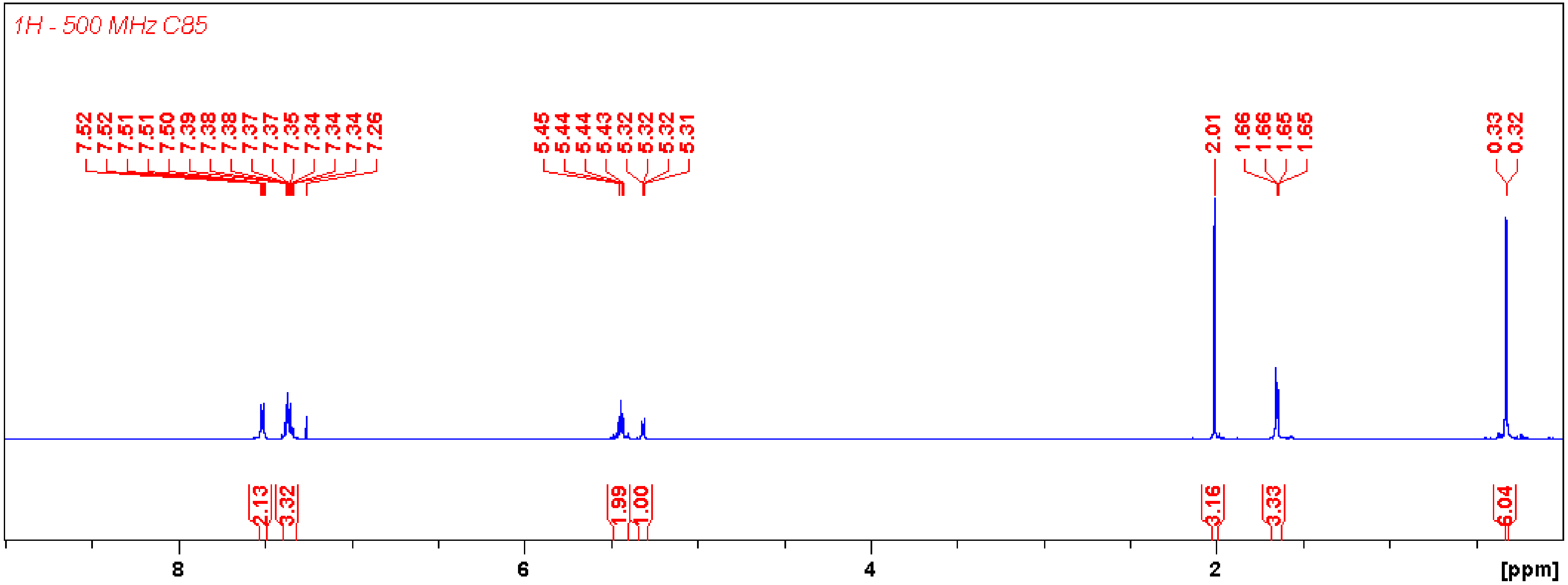

Figure S80. ^{13}C NMR (126 MHz, $CDCl_3$, 298 K) of **S14a**.

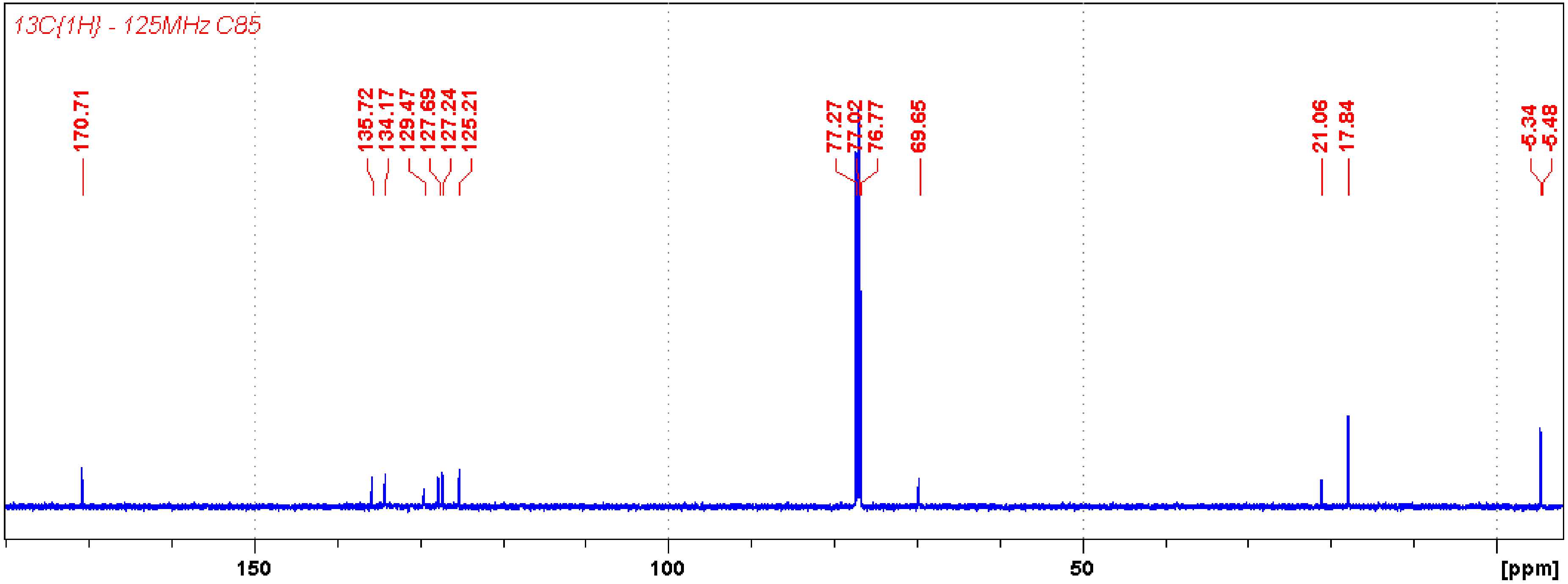

Figure S81. ^{29}Si DEPT NMR (99 MHz, $CDCl_3$, 298 K) of **S14a**.

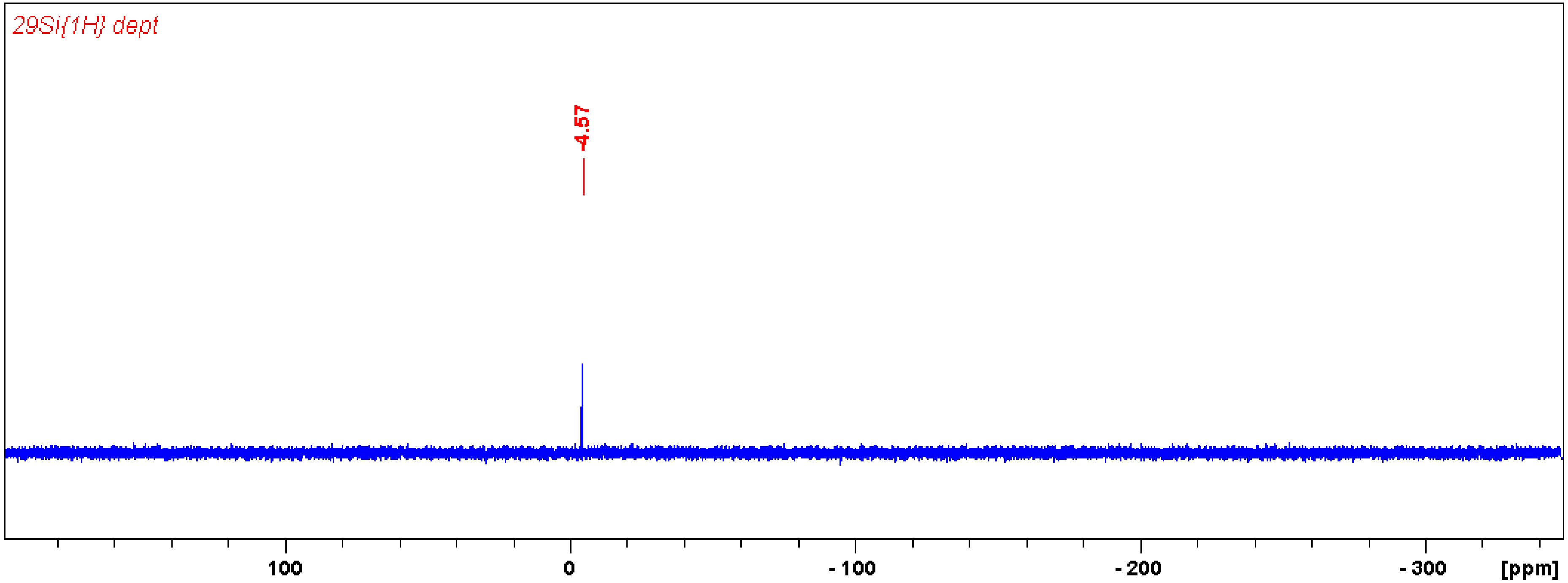

(1*E*)-(3-Chloro-2-methylbut-1-en-1-yl)(methyl)diphenylsilane (**12c**):

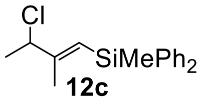

12c

Figure S82. ^1H NMR (500 MHz, $CDCl_3$, 298 K) of **12c**.

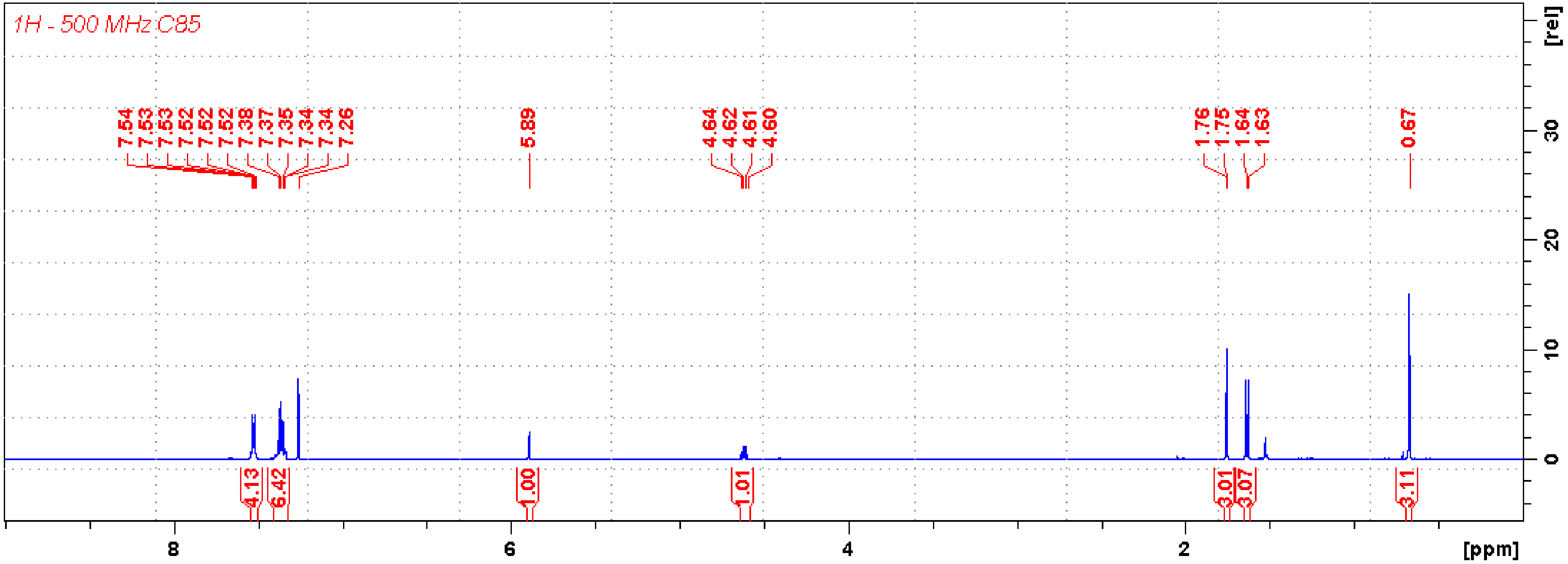

Figure S83. ^{13}C NMR (126 MHz, $CDCl_3$, 298 K) of **12c**.

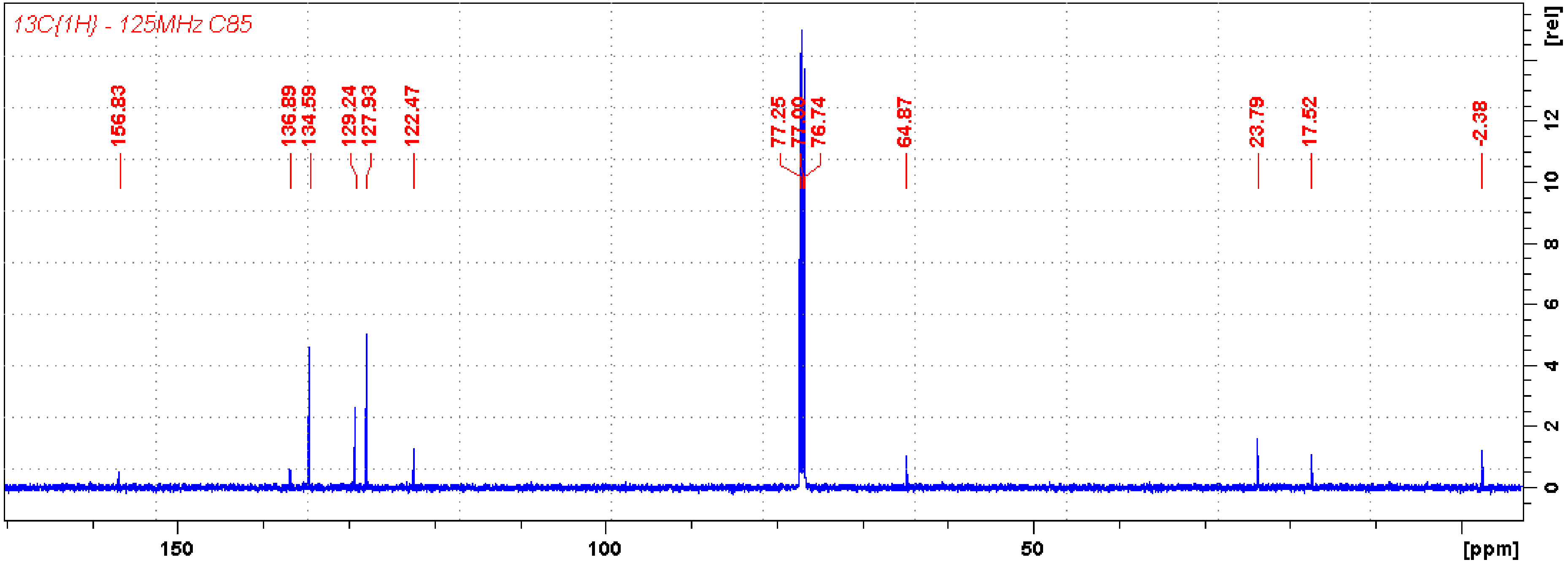

Figure S84. ^{29}Si DEPT NMR (99 MHz, $CDCl_3$, 298 K) of **12c**.

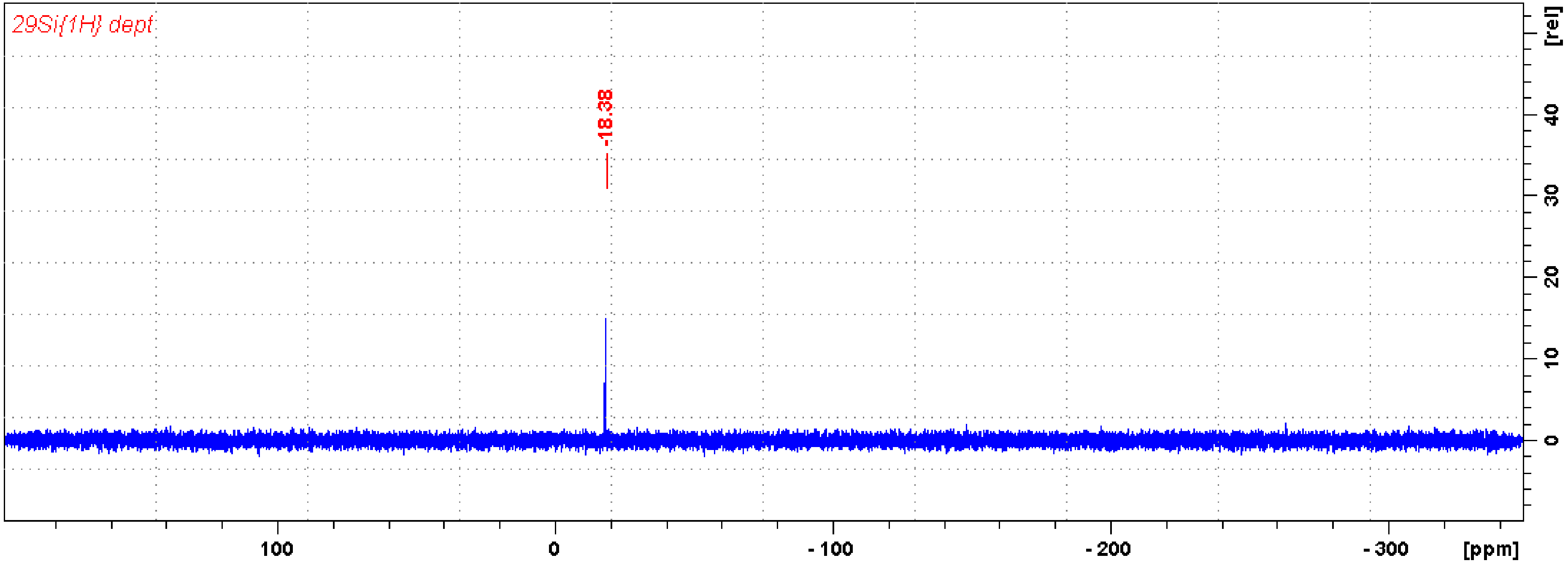

(1*E*)-(3-Chloro-2-methylhept-1-en-1-yl)(methyl)diphenylsilane (**12d**):

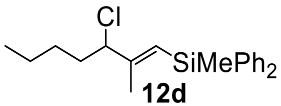

12d

Figure S85. ^{1}H NMR (500 MHz, $CDCl_3$, 298 K) of **12d**.

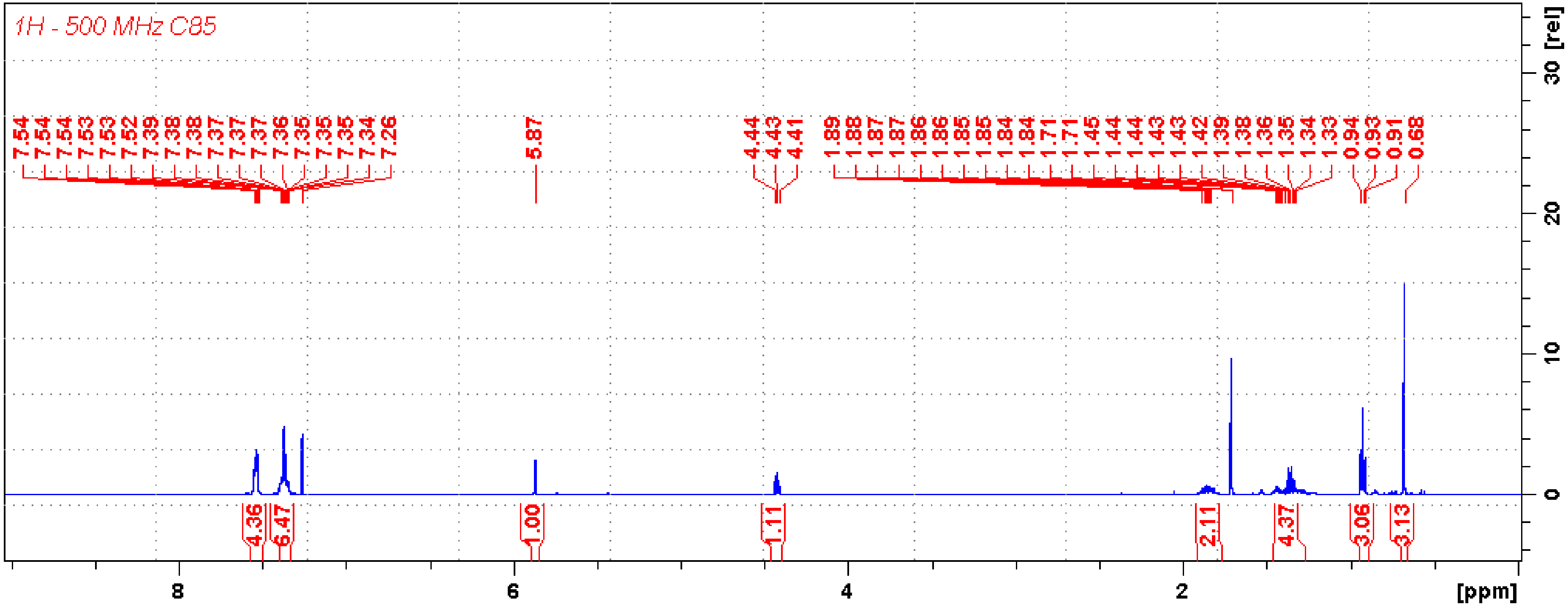

Figure S86. ^{13}C NMR (126 MHz, $CDCl_3$, 298 K) of **12d**.

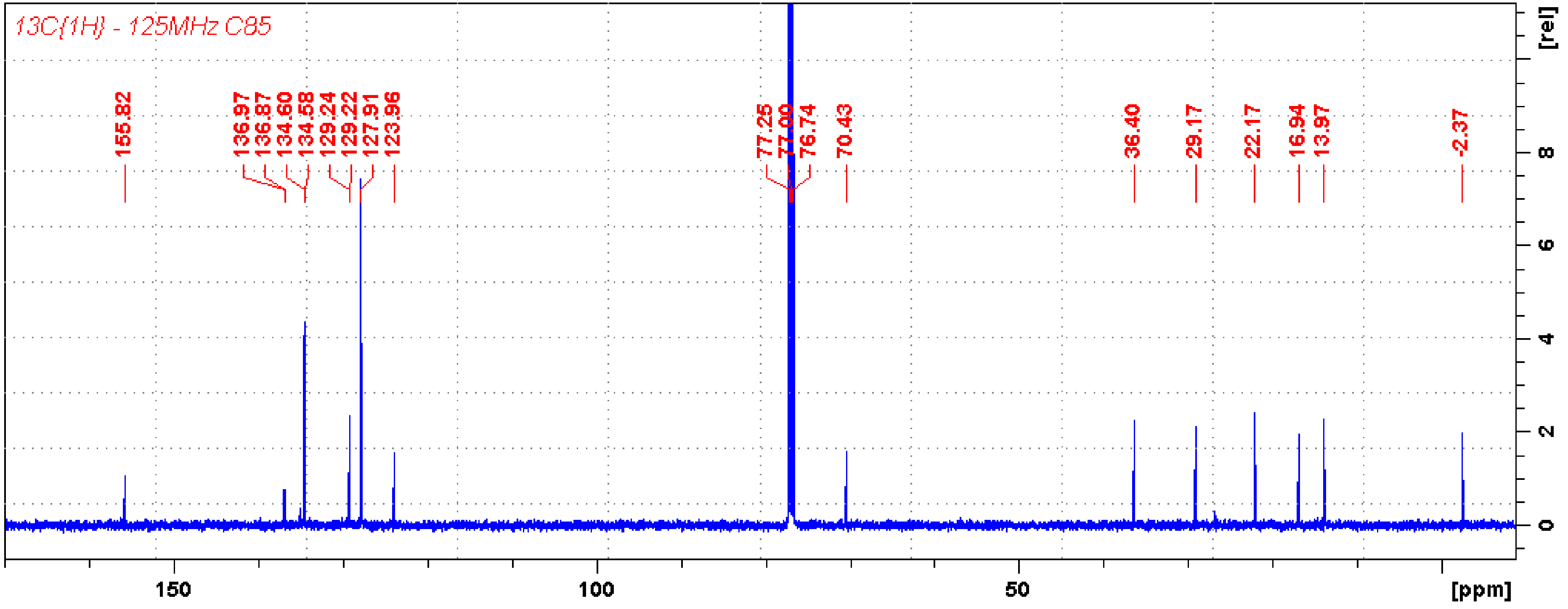

Figure S87. ^{29}Si DEPT NMR (99 MHz, $CDCl_3$, 298 K) of **12d**.

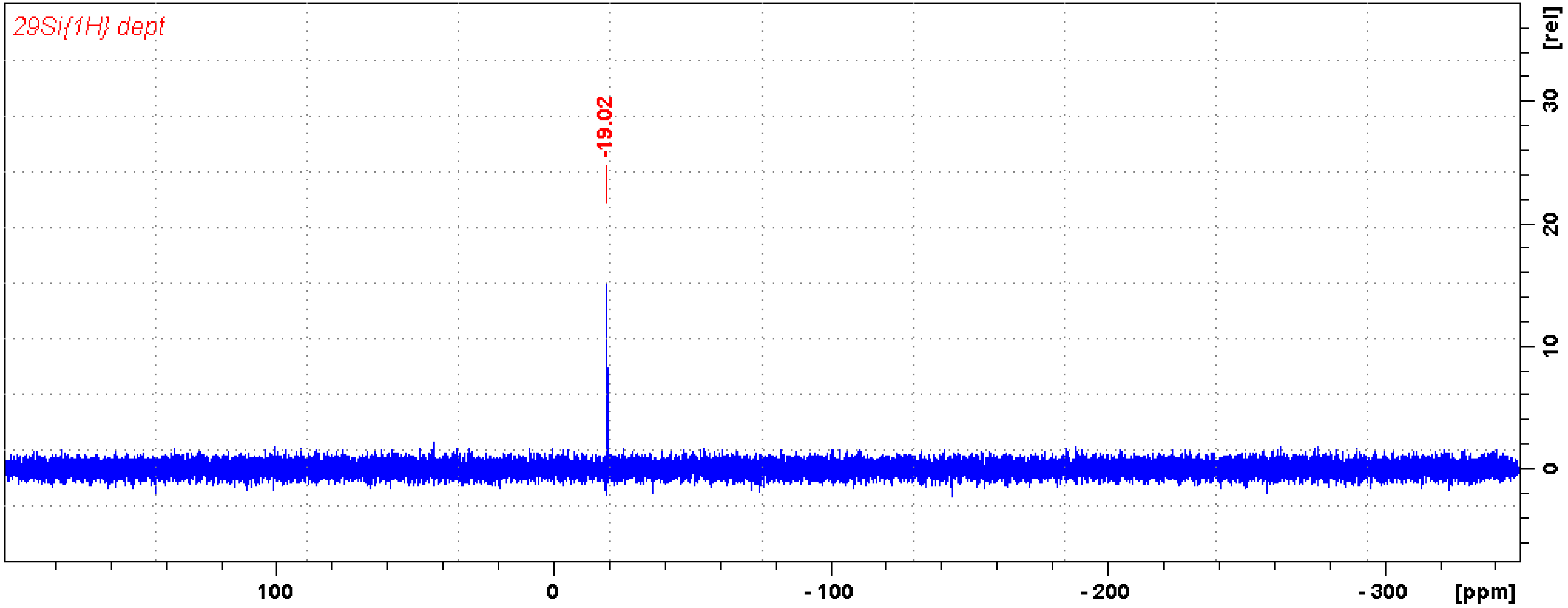

(3*R*,1*E*)-(5-(1,3-Dioxolan-2-yl)-3-methylpent-1-en-1-yl)dimethyl(phenyl)silane (**3aa**):

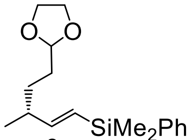

3aa

Figure S88. ^{1}H NMR (500 MHz, $CDCl_3$, 298 K) of **3aa**.

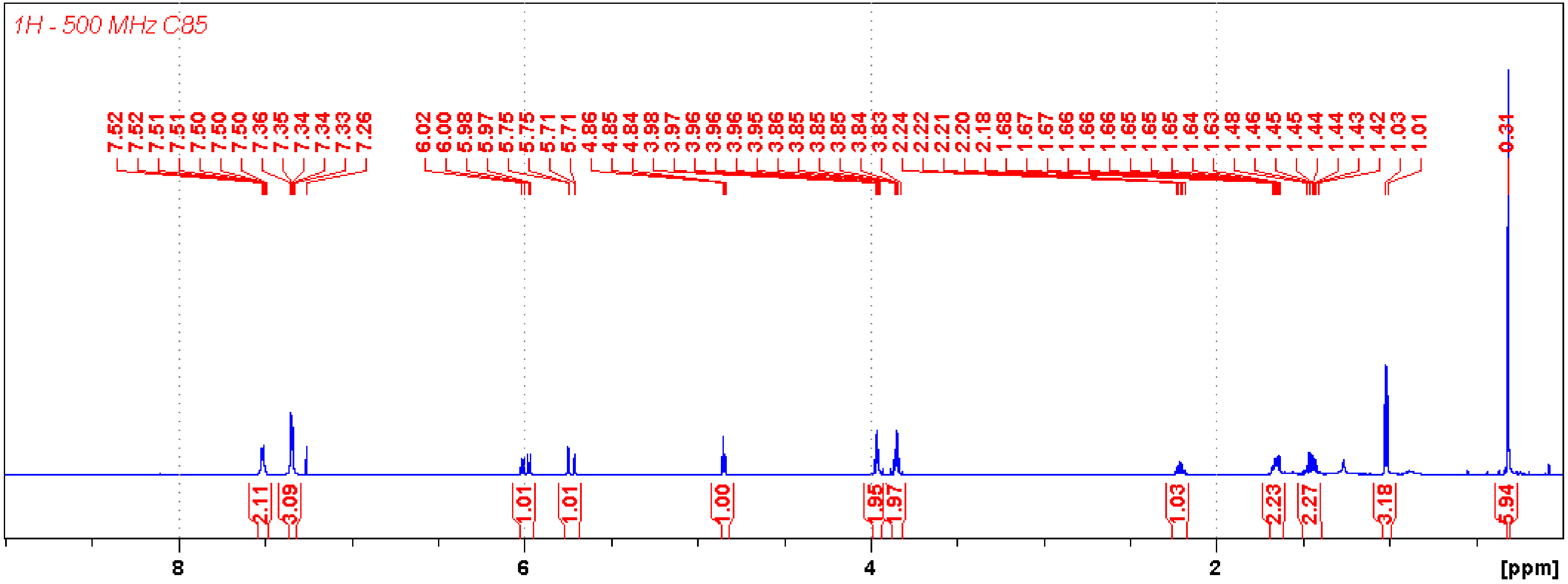

Figure S89. ^{13}C NMR (126 MHz, $CDCl_3$, 298 K) of **3aa**.

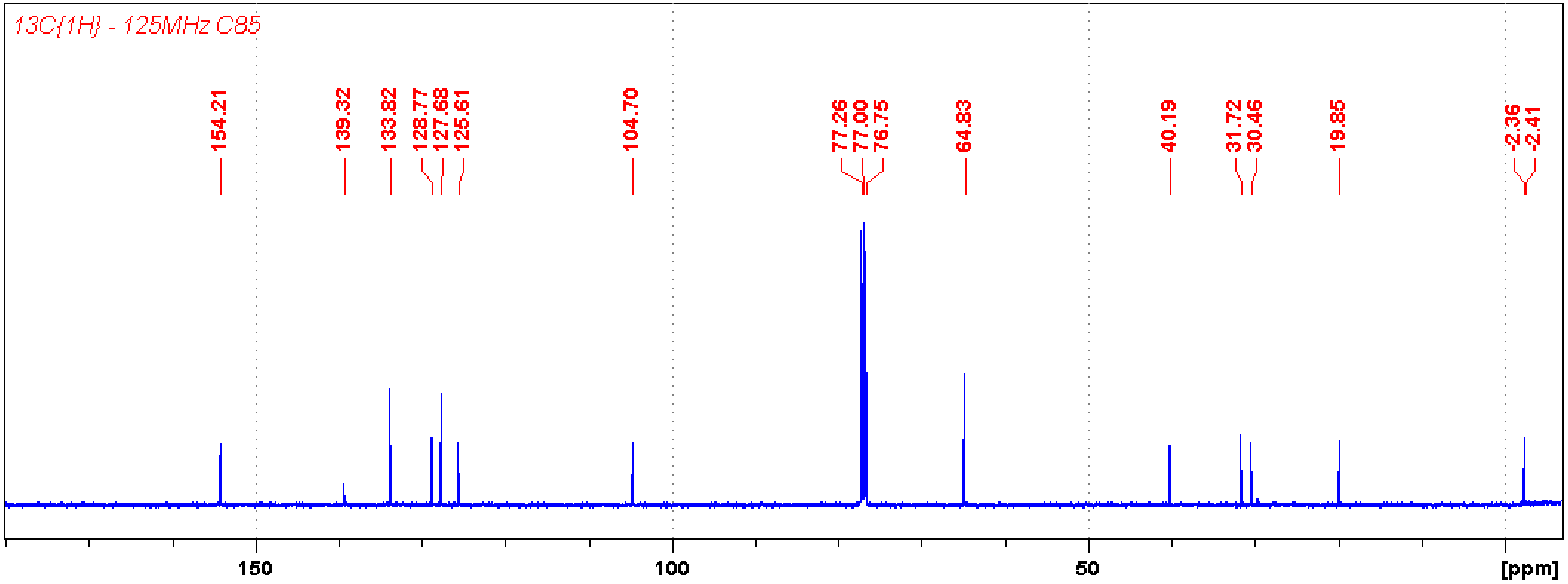

Figure S90. ^{29}Si DEPT NMR (99 MHz, $CDCl_3$, 298 K) of **3aa**.

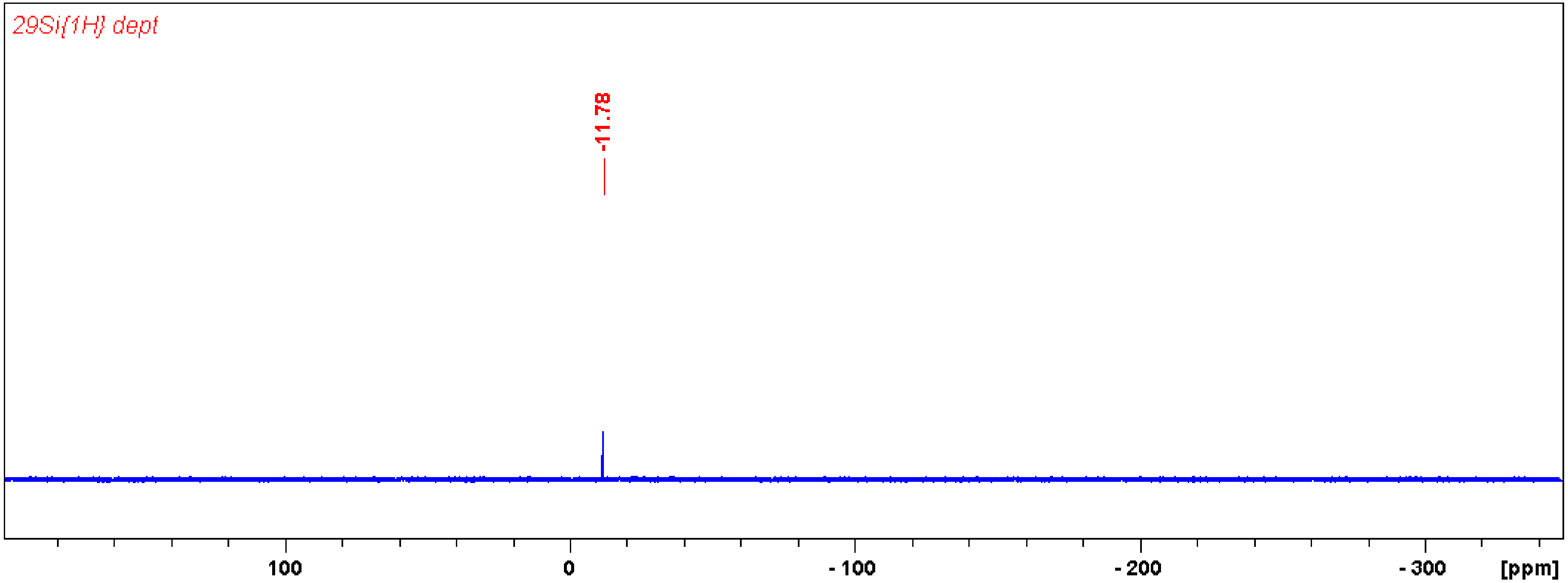

(3*R*,1*E*)-(5-(1,3-Dioxan-2-yl)-3-methylpent-1-en-1-yl)dimethyl(phenyl)silane (**3ab**):

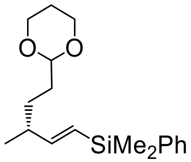

3ab

Figure S91. ^{1}H NMR (500 MHz, $CDCl_3$, 298 K) of **3ab**.

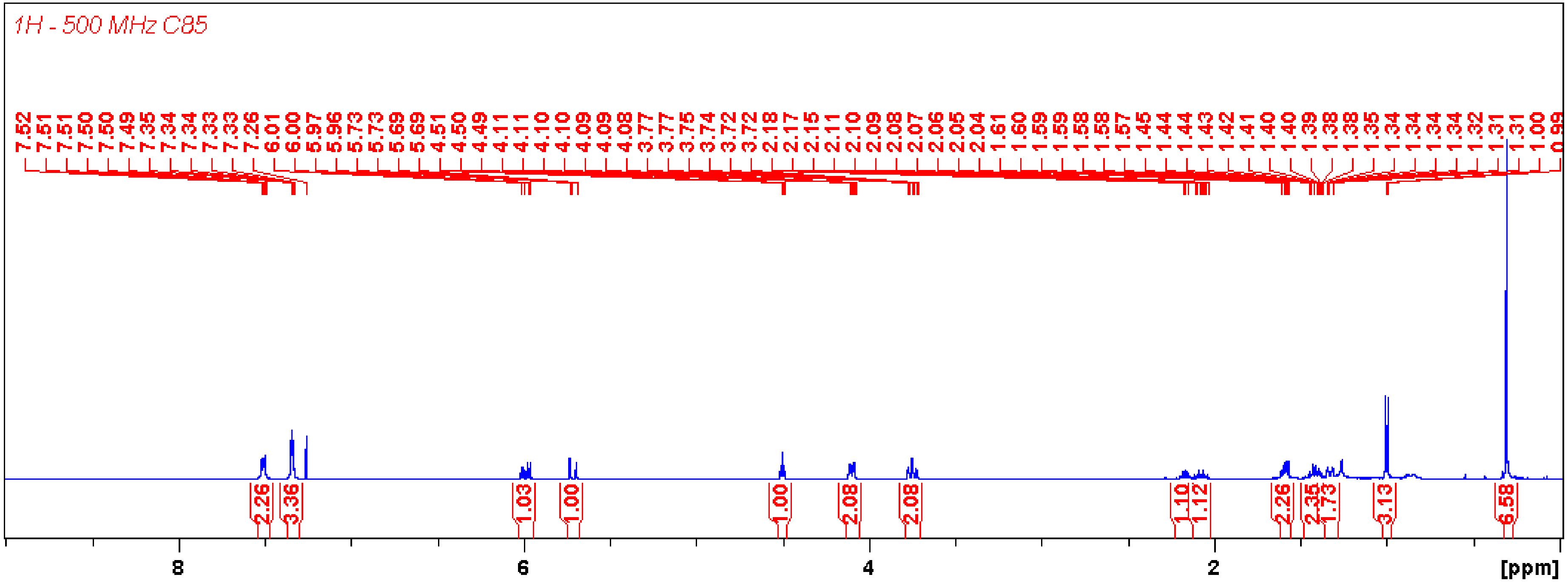

Figure S92. ^{13}C NMR (126 MHz, $CDCl_3$, 298 K) of **3ab**.

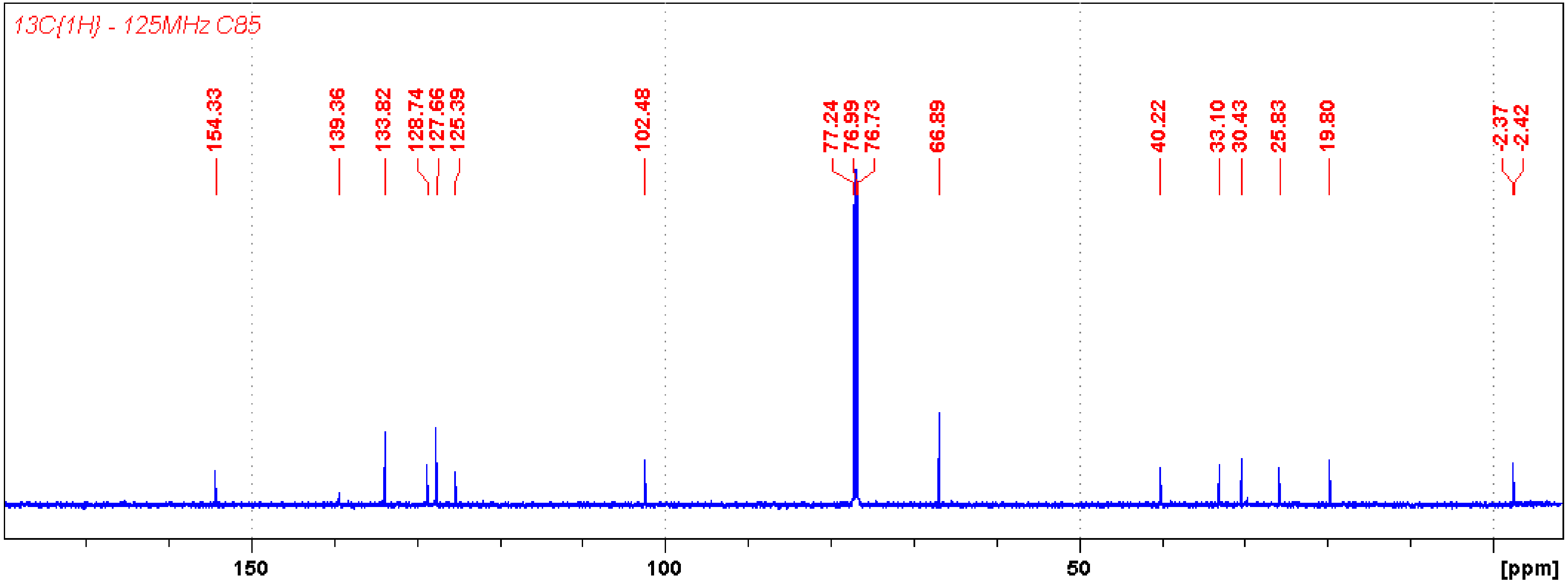

Figure S93. ^{29}Si DEPT NMR (99 MHz, $CDCl_3$, 298 K) of **3ab**.

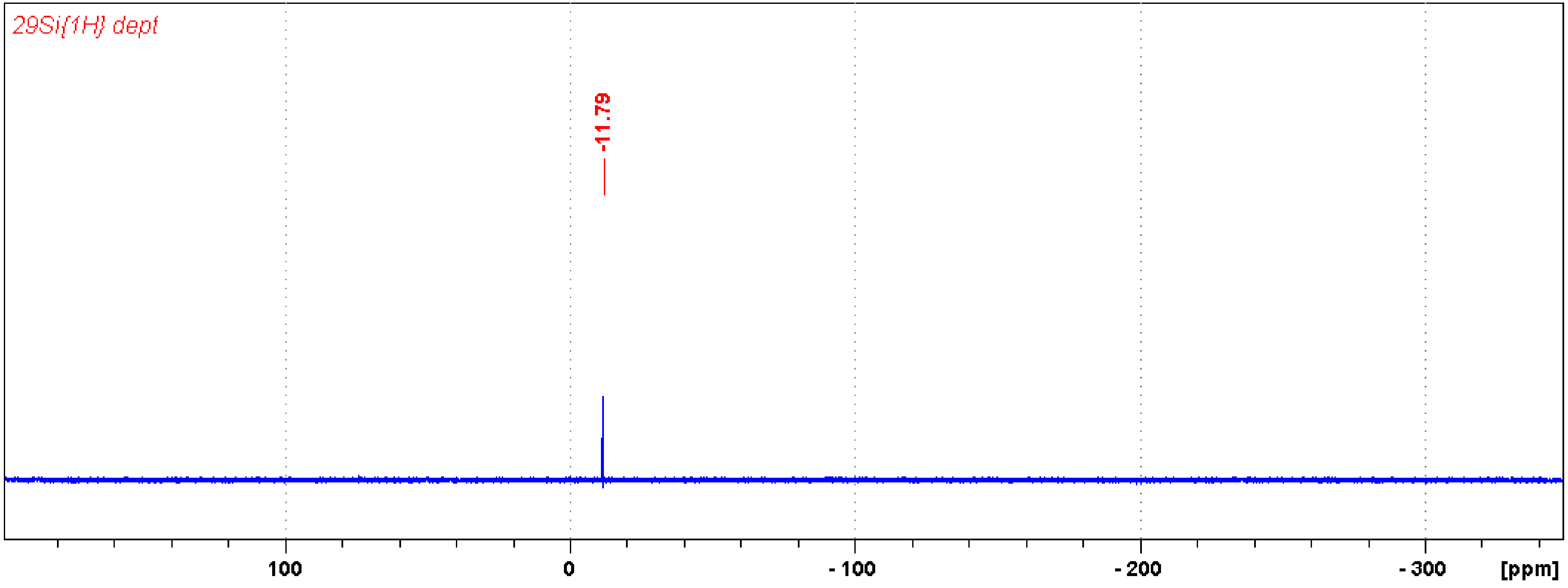

(3*R*,1*E*)-Dimethyl(3-methyl-6-phenoxyhex-1-en-1-yl)(phenyl)silane (**3ac**):

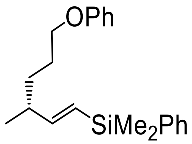

3ac

Figure S94. ^{1}H NMR (500 MHz, $CDCl_3$, 298 K) of **3ac**.

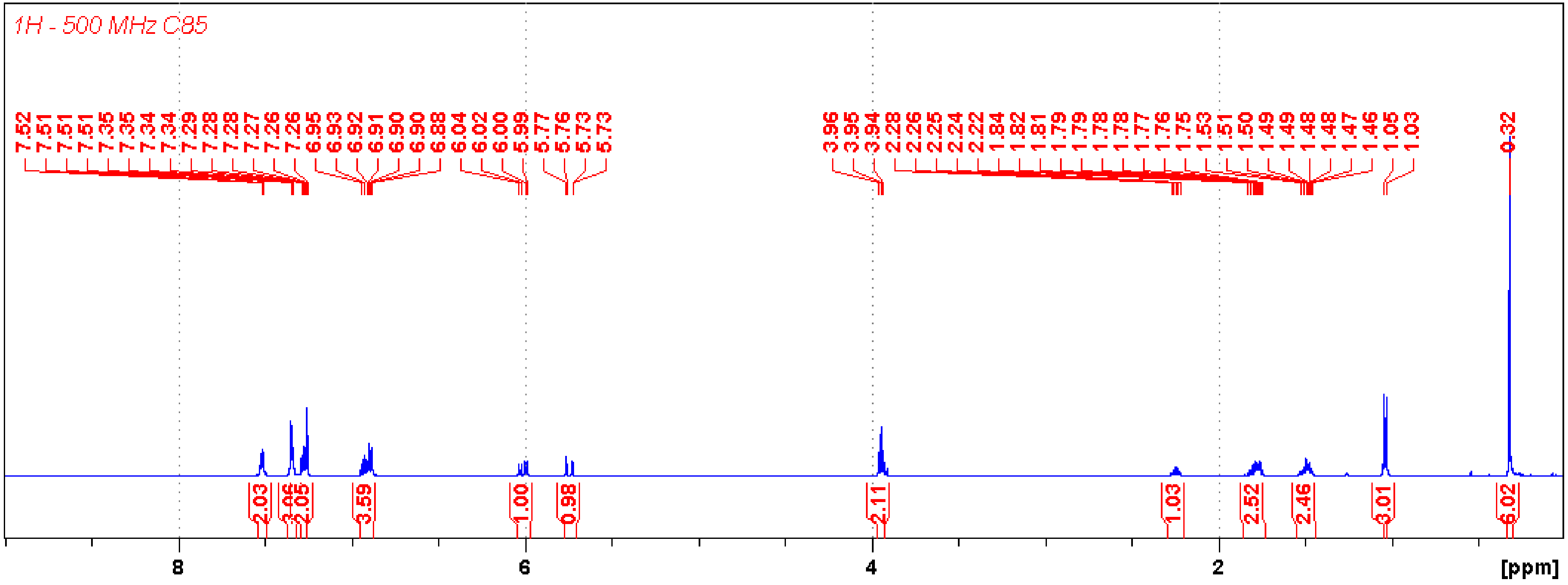

Figure S95. ^{13}C NMR (126 MHz, $CDCl_3$, 298 K) of **3ac**.

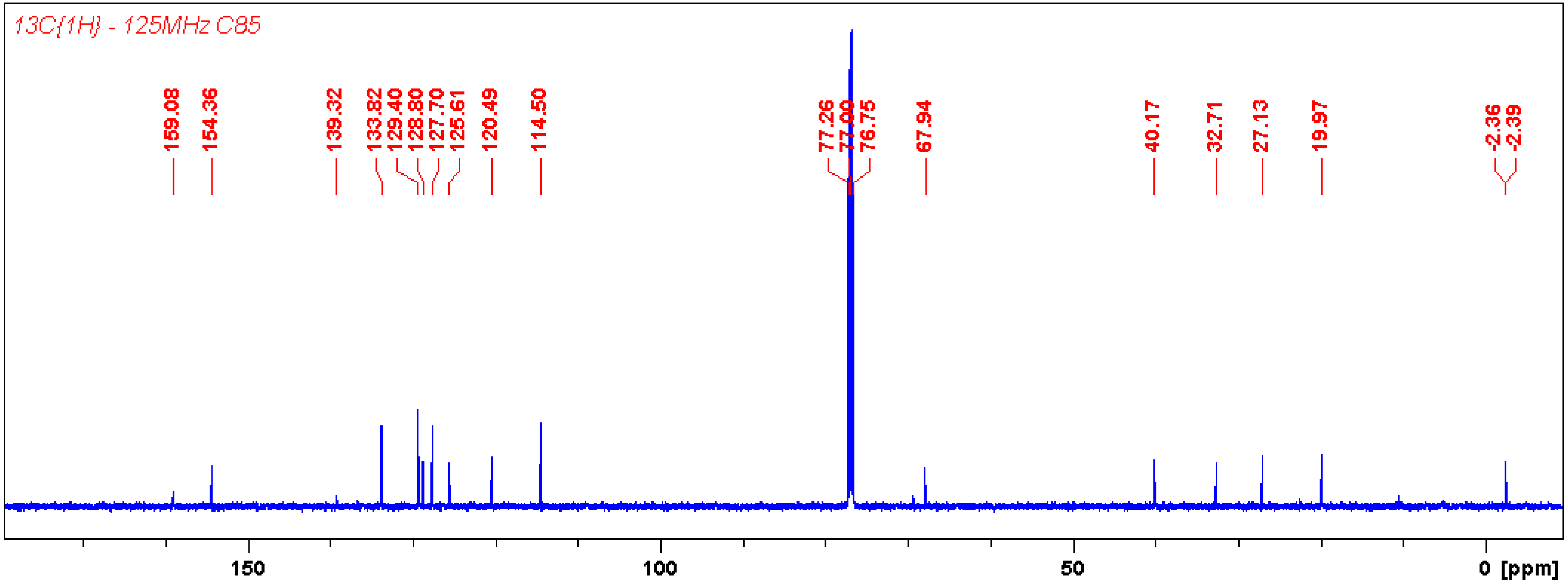

Figure S96. ^{29}Si DEPT NMR (99 MHz, $CDCl_3$, 298 K) of **3ac**.

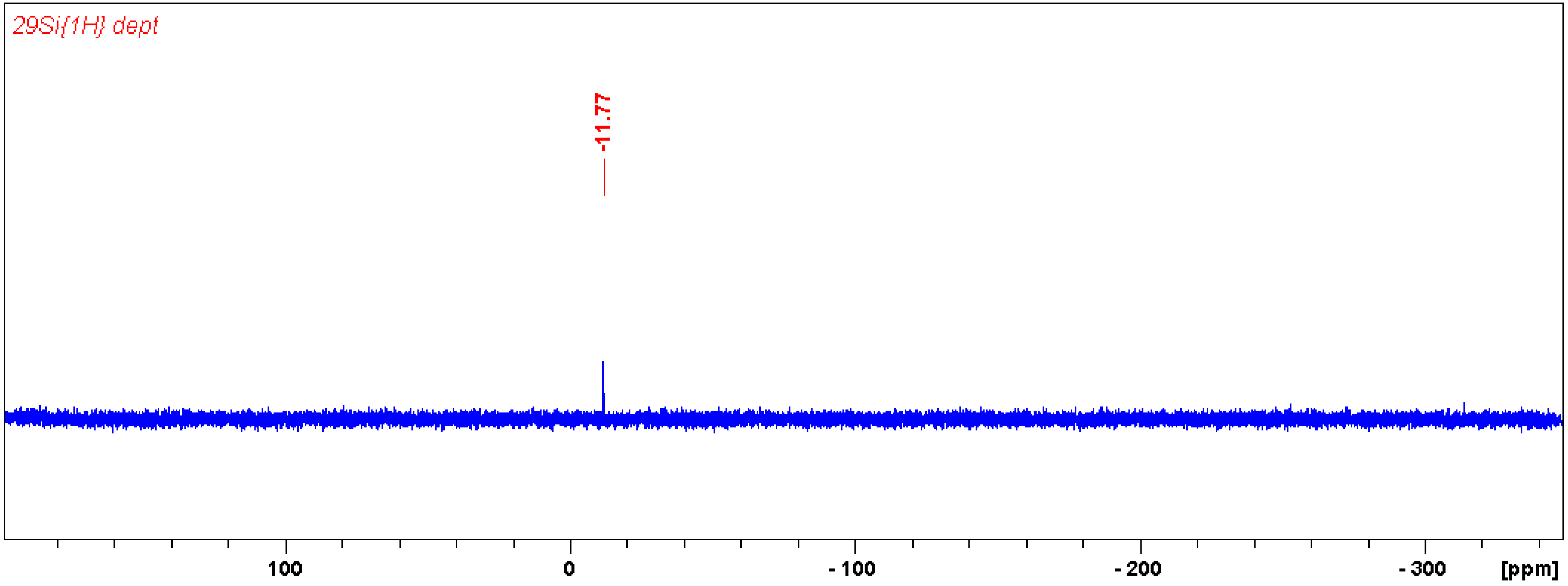

(3*R*,1*E*)-Dimethyl(3-methyl-6-phenylhex-1-en-1-yl)(phenyl)silane (**3ad**):

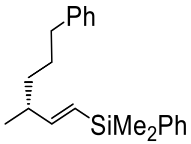

3ad

Figure S97. ^{1}H NMR (500 MHz, $CDCl_3$, 298 K) of **3ad**.

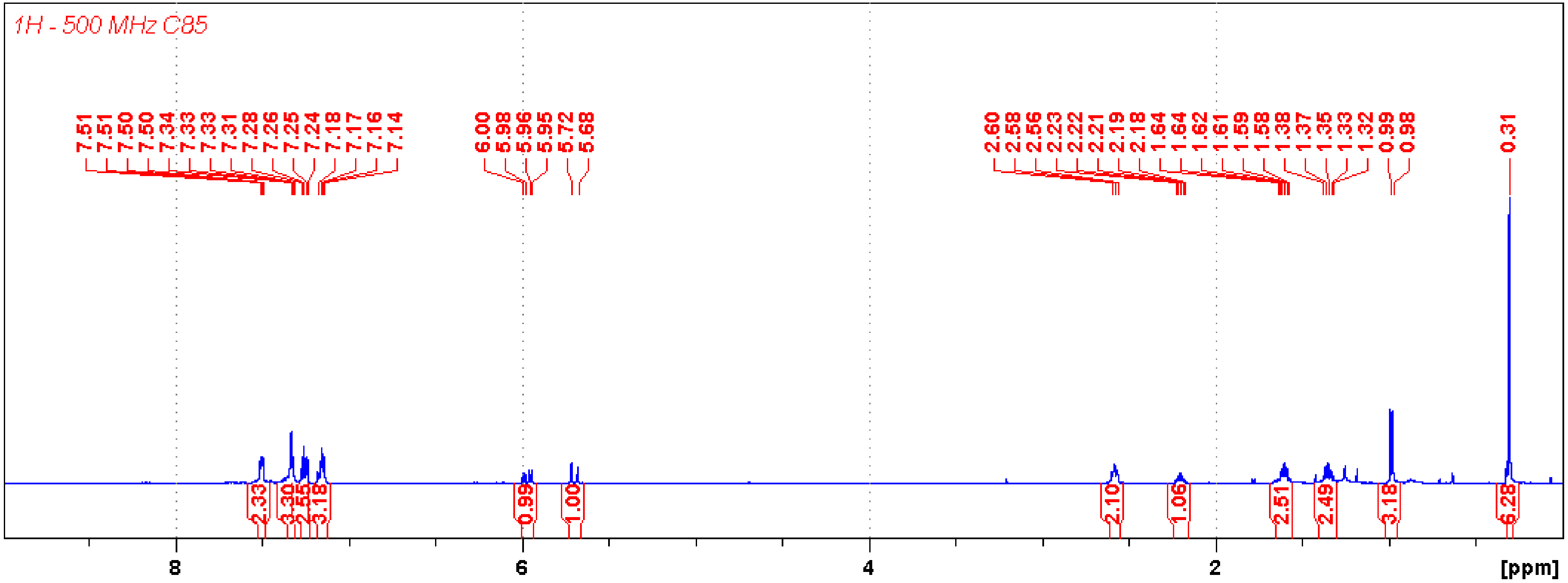

Figure S98. ^{13}C NMR (126 MHz, $CDCl_3$, 298 K) of **3ad**.

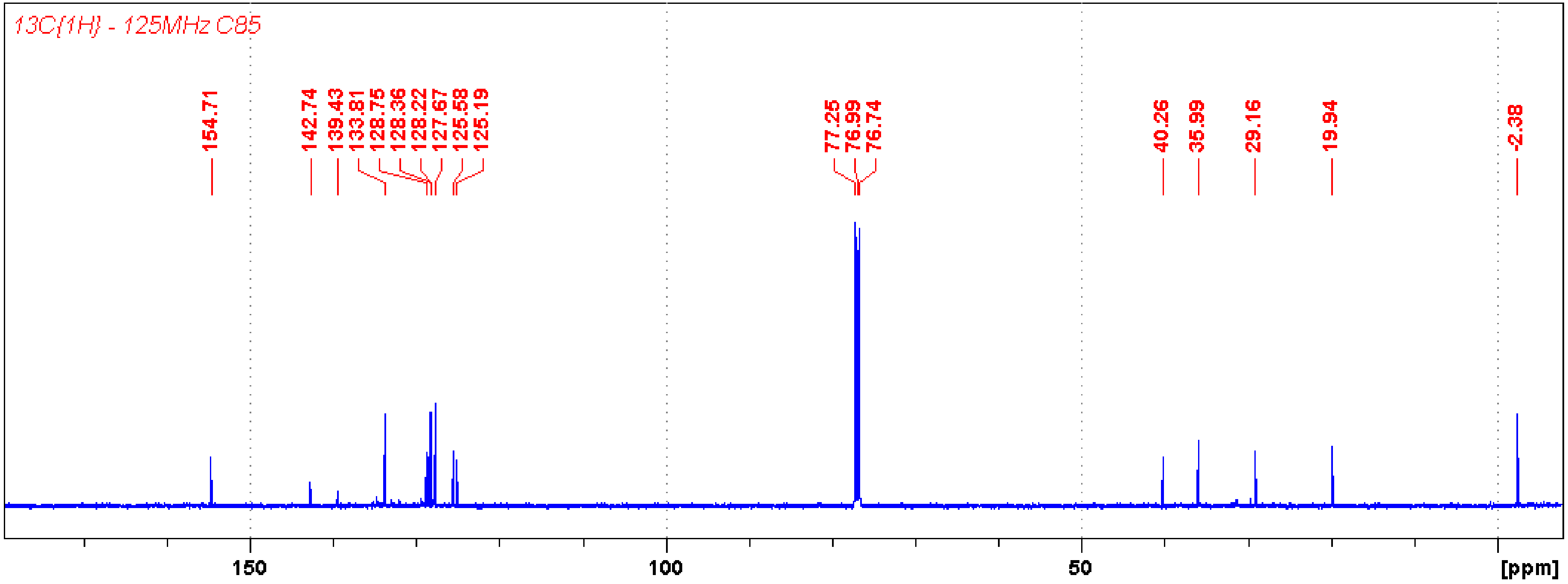

Figure S99. ^{29}Si DEPT NMR (99 MHz, $CDCl_3$, 298 K) of **3ad**.

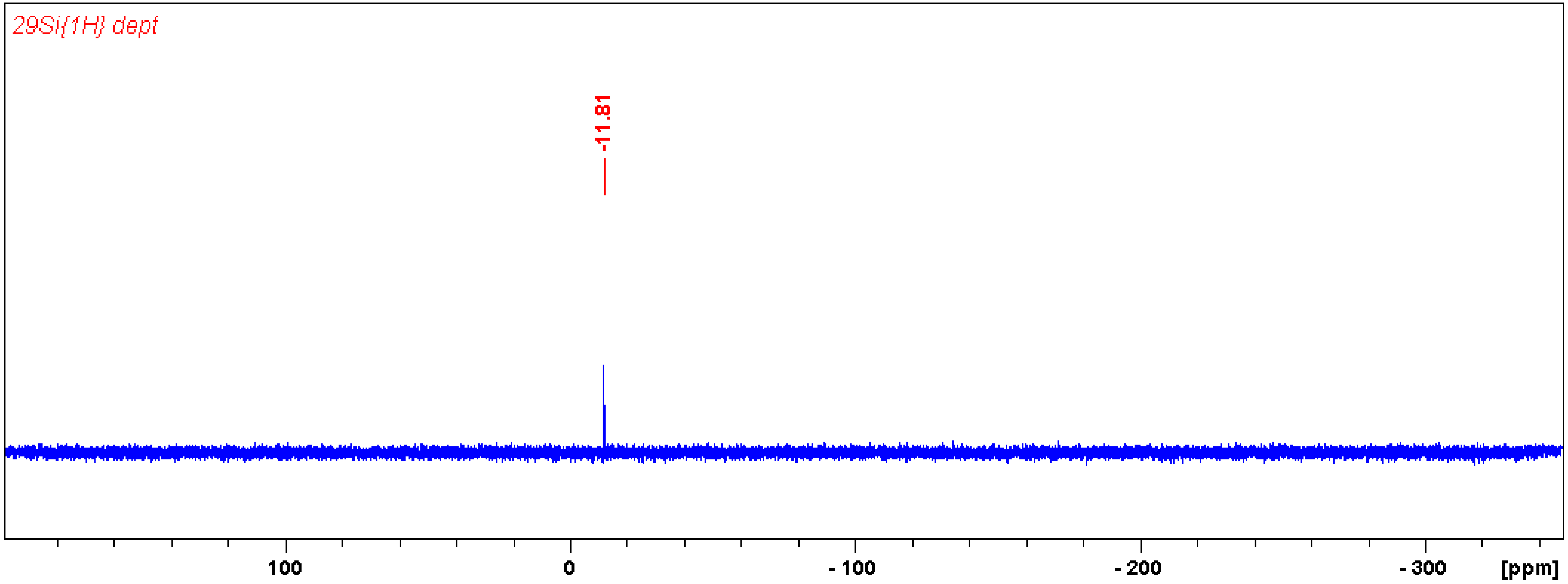

(3*R*,1*E*)-(6-Cyano-3-methylhex-1-en-1-yl)dimethyl(phenyl)silane (**3ae**):

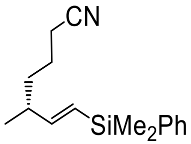

3ae

Figure S100. ^{1}H NMR (500 MHz, $CDCl_3$, 298 K) of **3ae**.

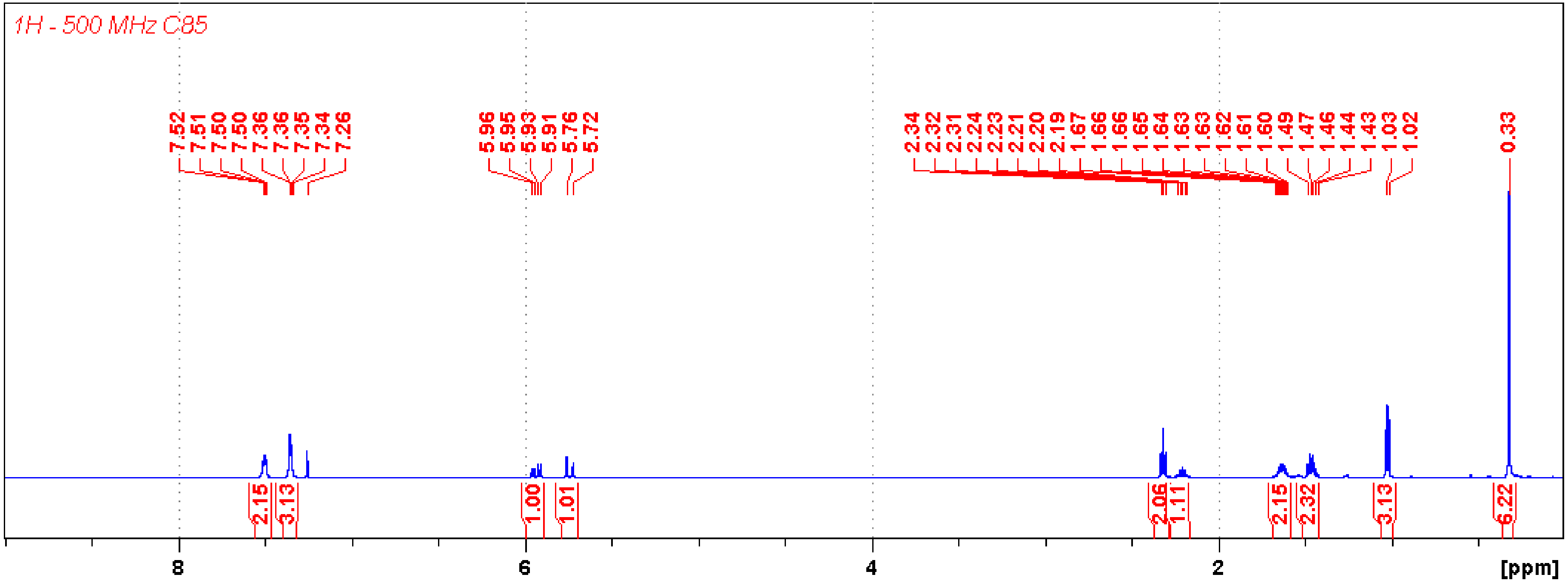

Figure S101. ^{13}C NMR (126 MHz, $CDCl_3$, 298 K) of **3ae**.

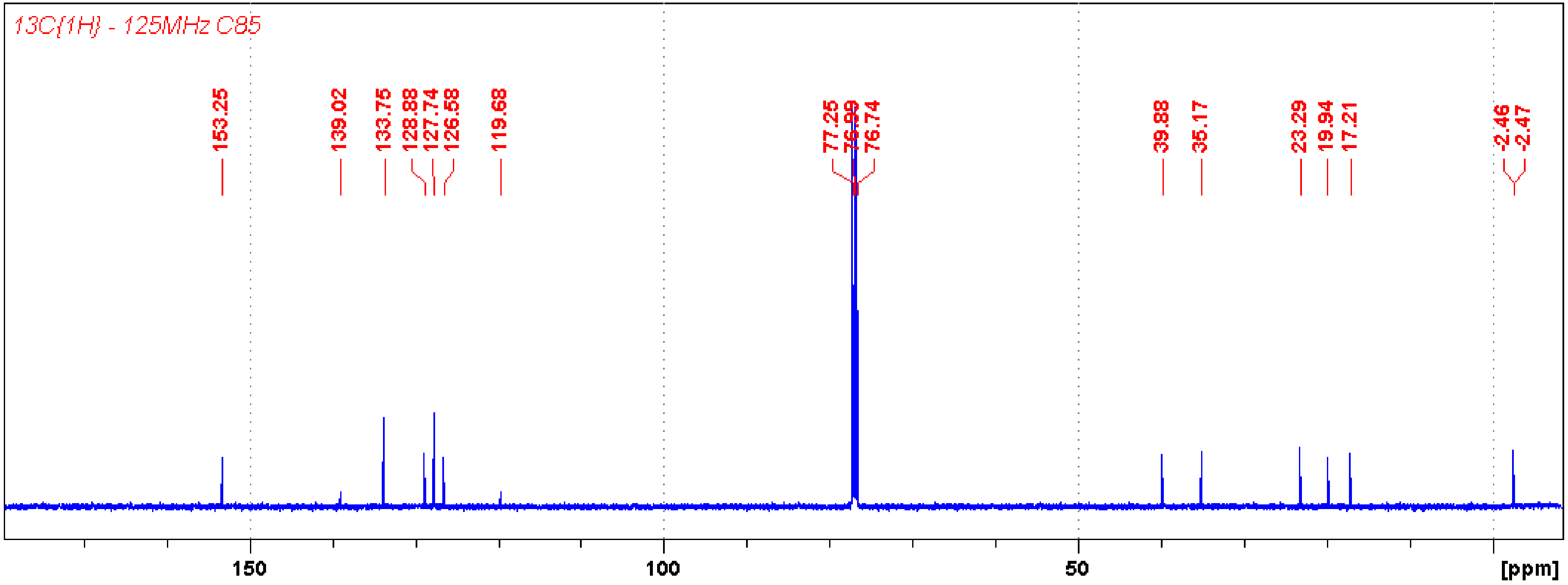

Figure S102. ^{29}Si DEPT NMR (99 MHz, $CDCl_3$, 298 K) of **3ae**.

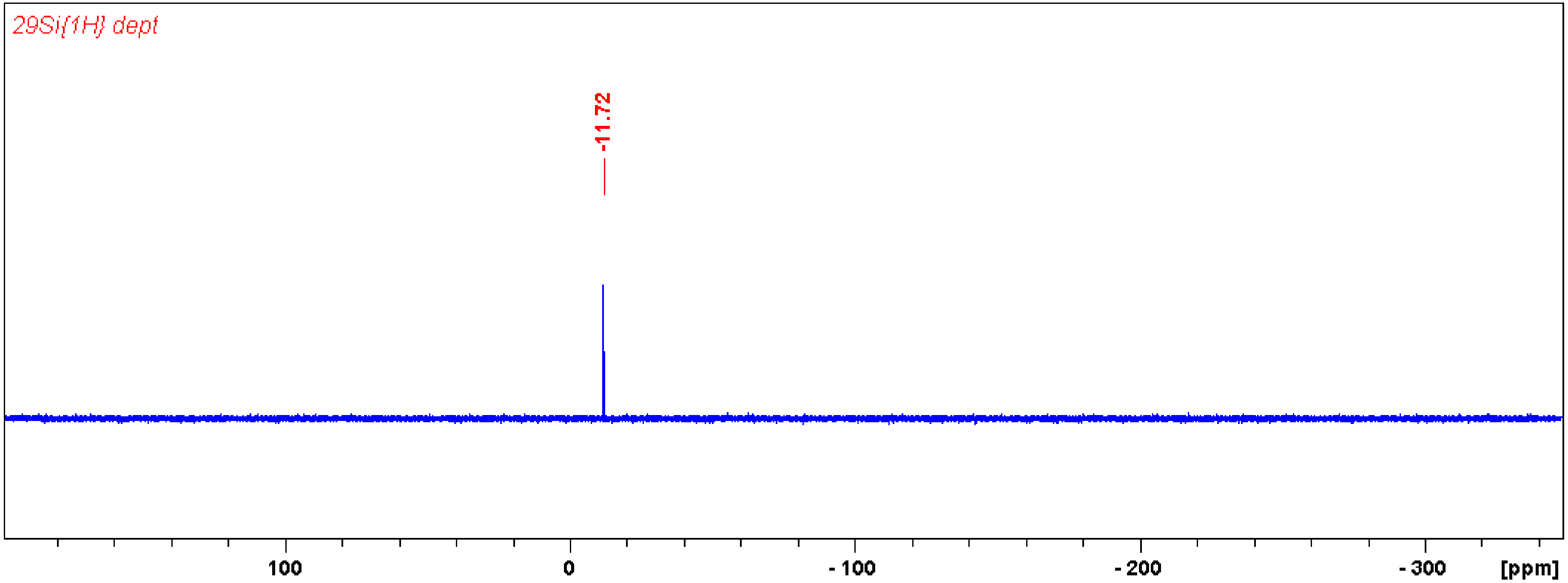

(3*R*,1*E*)-(7-Ethoxy-7-oxo-3-methylhept-1-en-1-yl)dimethyl(phenyl)silane (**3af**):

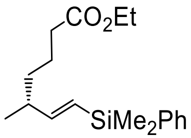

3af

Figure S103. ^{1}H NMR (500 MHz, $CDCl_3$, 298 K) of **3af**.

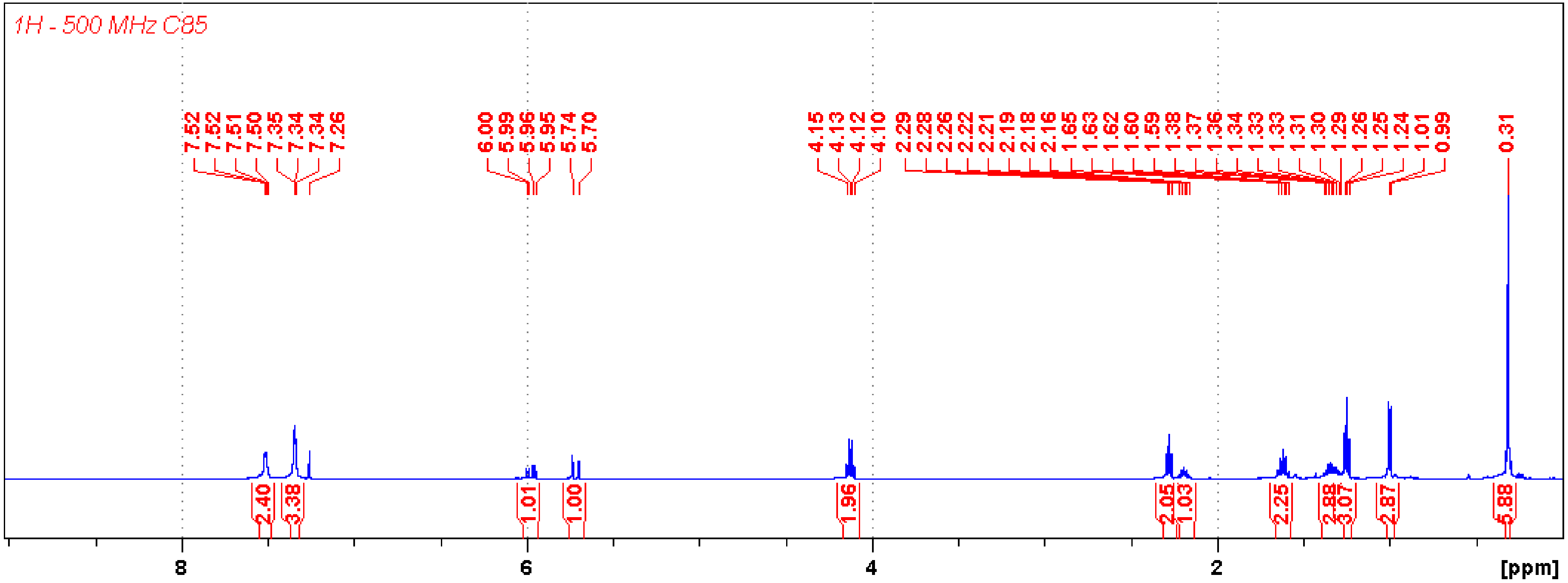

Figure S104. ^{13}C NMR (126 MHz, $CDCl_3$, 298 K) of **3af**.

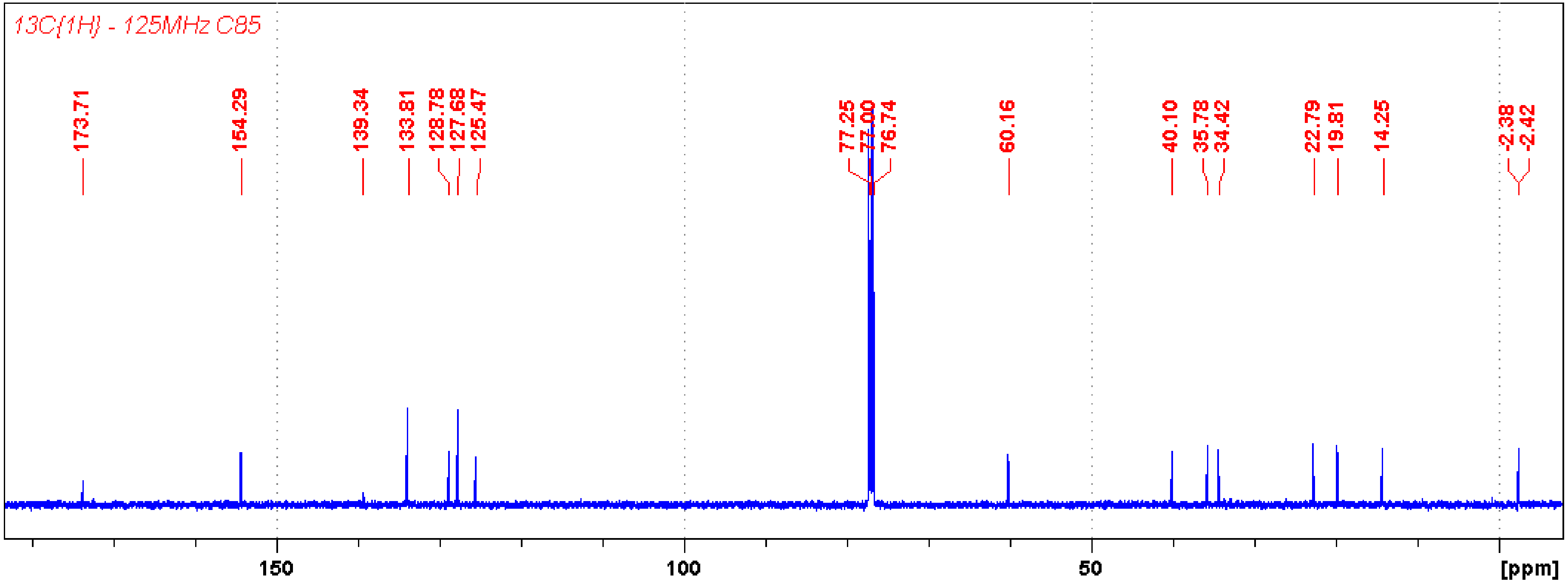

Figure S105. ^{29}Si DEPT NMR (99 MHz, $CDCl_3$, 298 K) of **3af**.

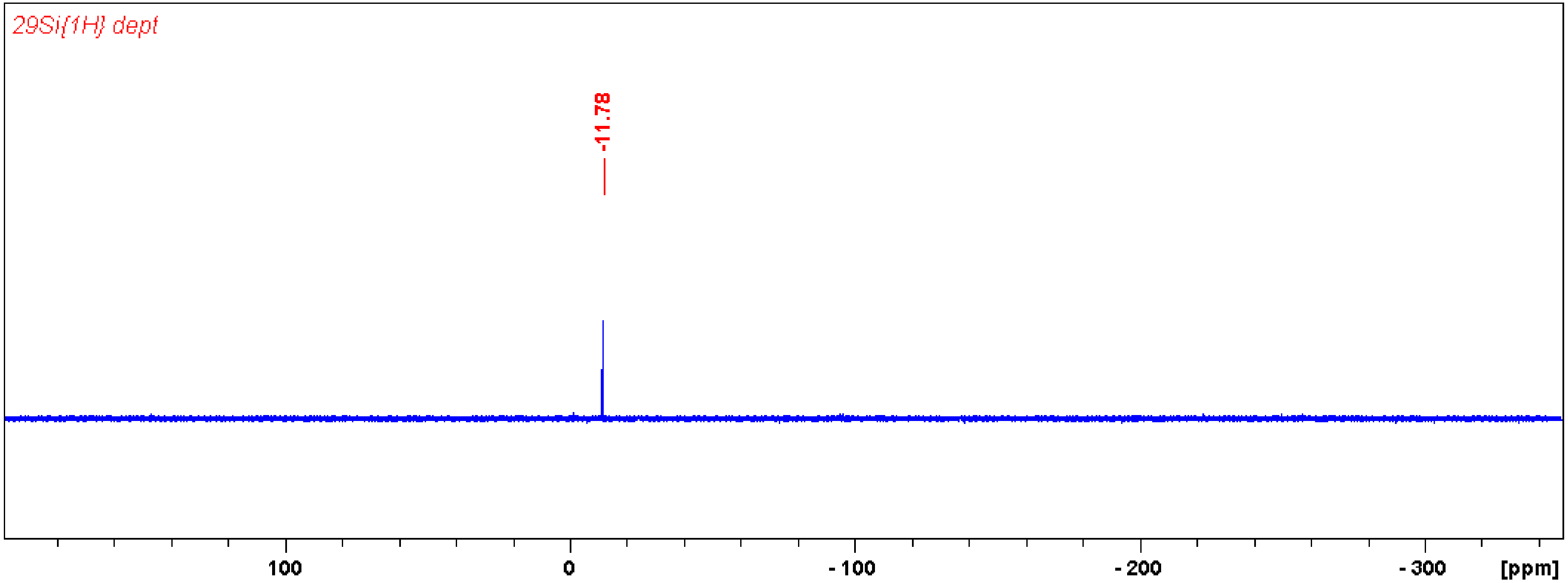

(3*R*,1*E*)-Dimethyl(3-methylnon-1-en-1-yl)(phenyl)silane (3ag):

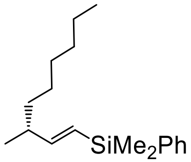

3ag

Figure S106. ^{1}H NMR (500 MHz, $CDCl_3$, 298 K) of **3ag**.

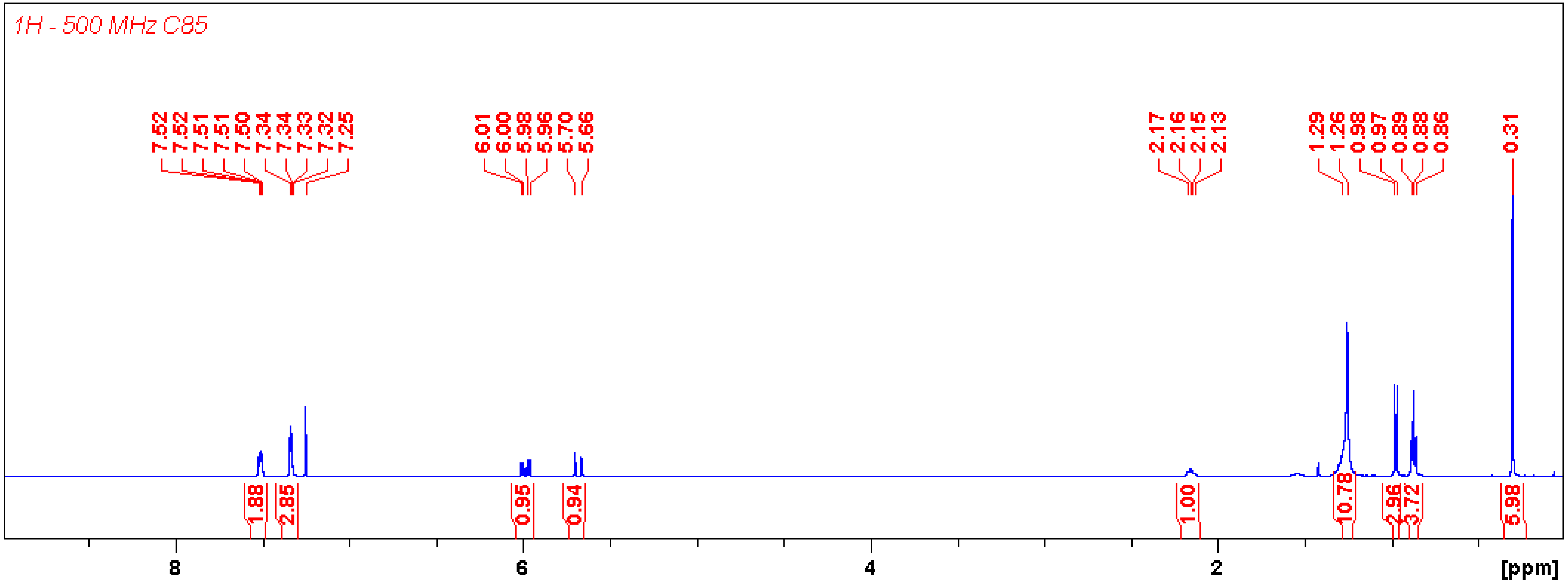

Figure S107. ^{13}C NMR (126 MHz, $CDCl_3$, 298 K) of **3ag**.

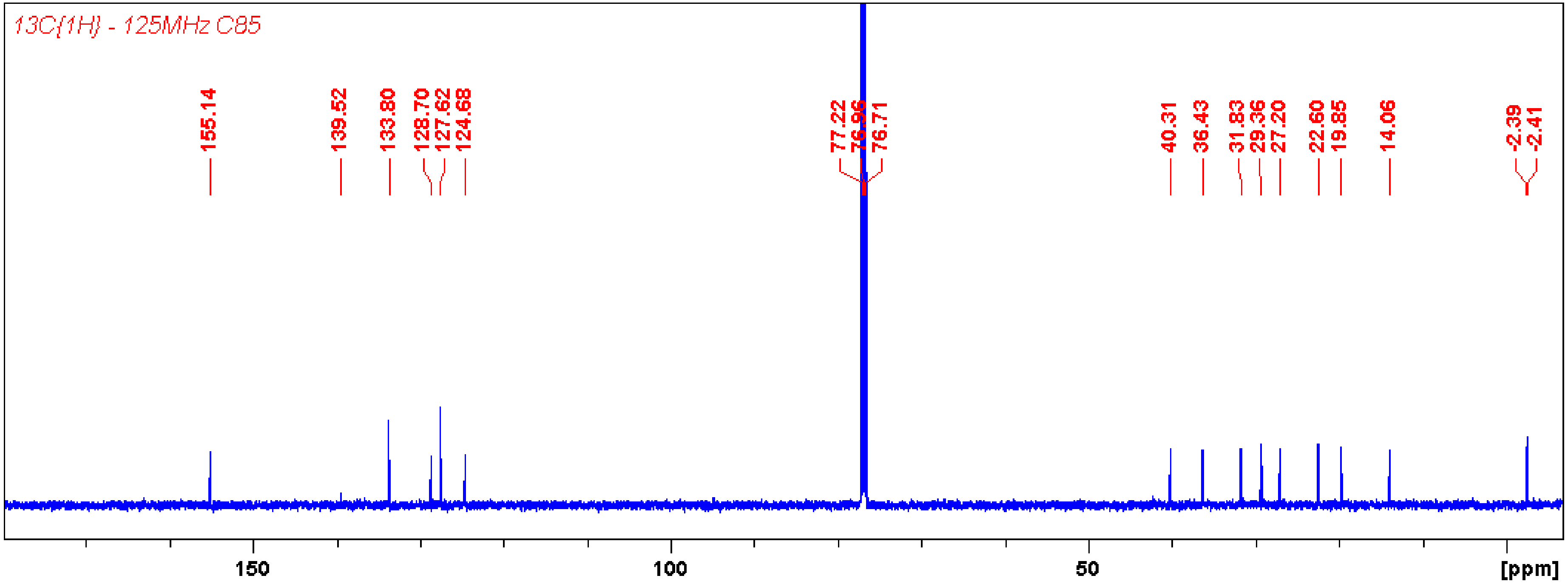

Figure S108. ^{29}Si DEPT NMR (99 MHz, $CDCl_3$, 298 K) of **3ag**.

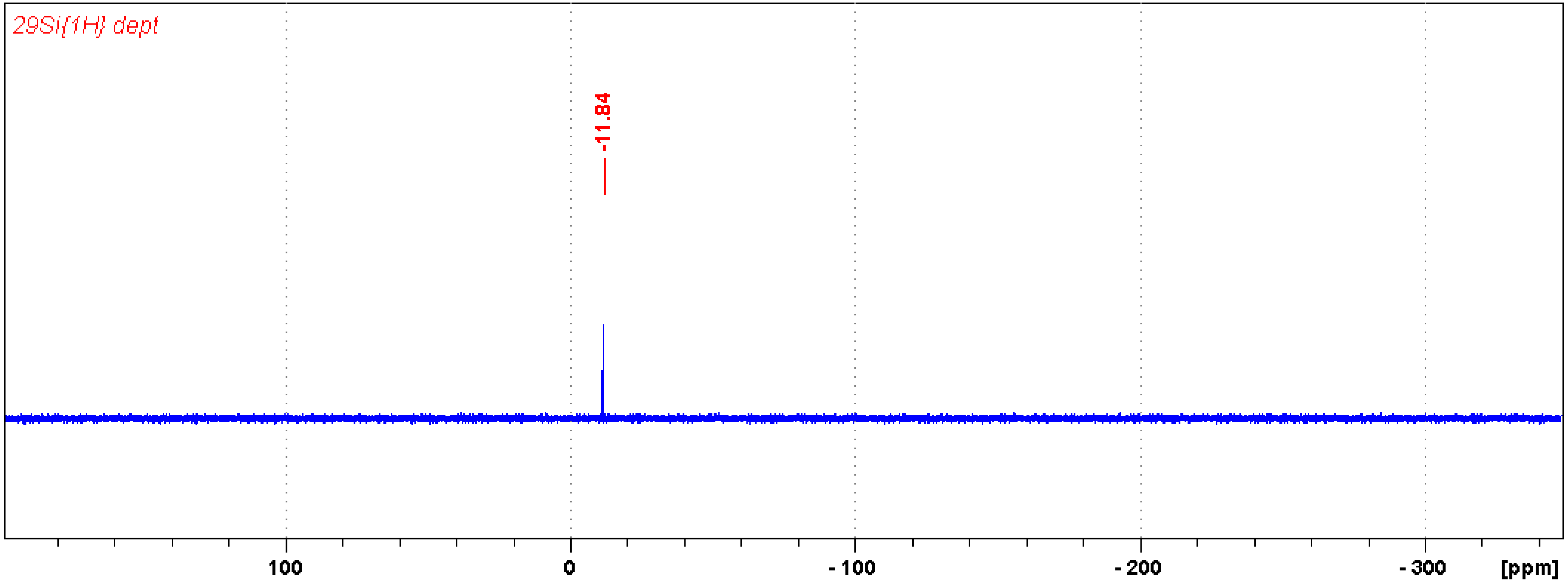

(3*R*,1*E*)-Dimethyl(3-methyloct-1-en-1-yl)(phenyl)silane (**3ah**):

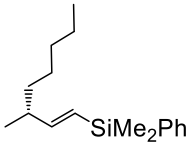

3ah

Figure S109. ^{1}H NMR (500 MHz, $CDCl_3$, 298 K) of **3ah**.

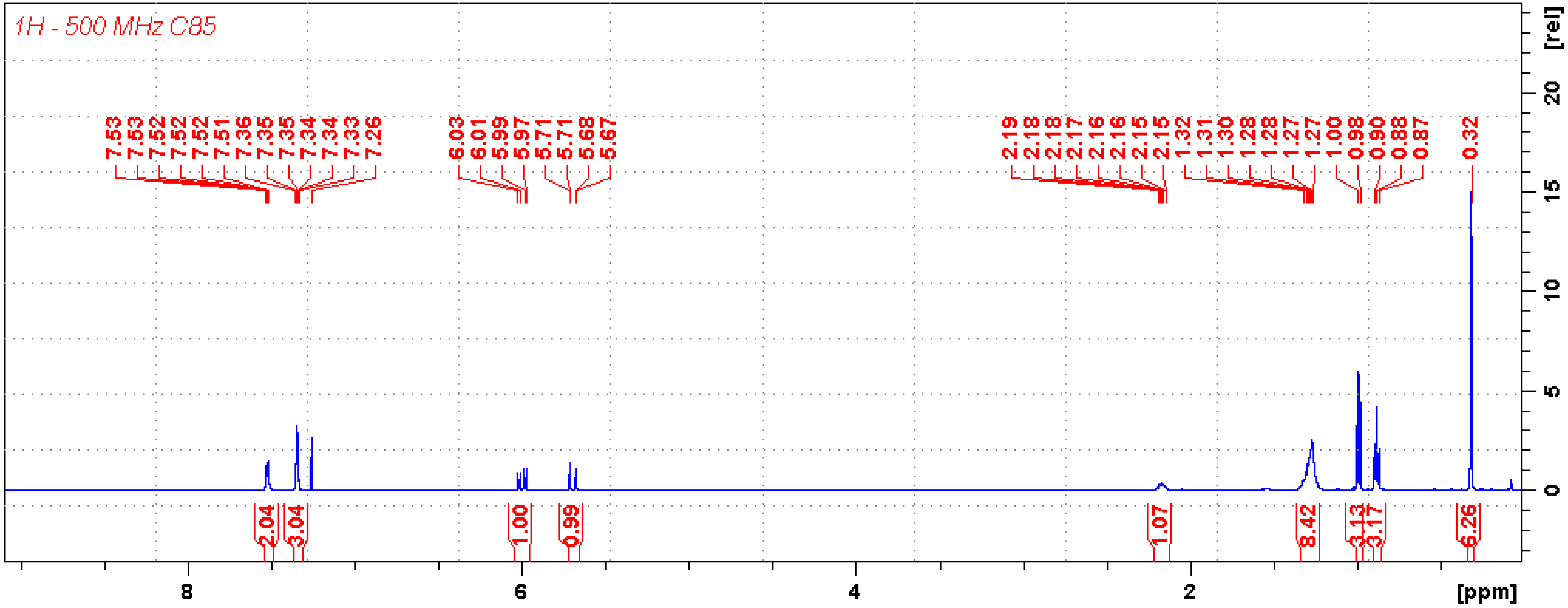

Figure S110. ^{13}C NMR (126 MHz, $CDCl_3$, 298 K) of **3ah**.

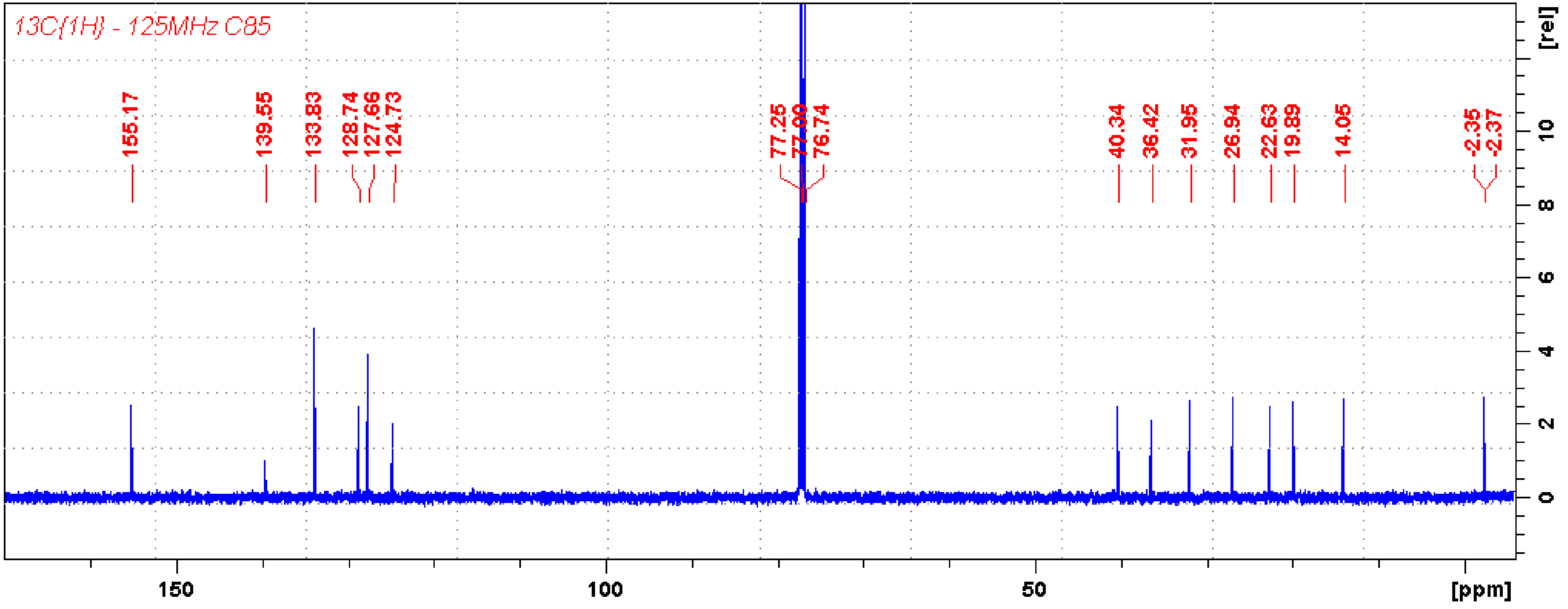

Figure S111. ^{29}Si DEPT NMR (99 MHz, $CDCl_3$, 298 K) of **3ah**.

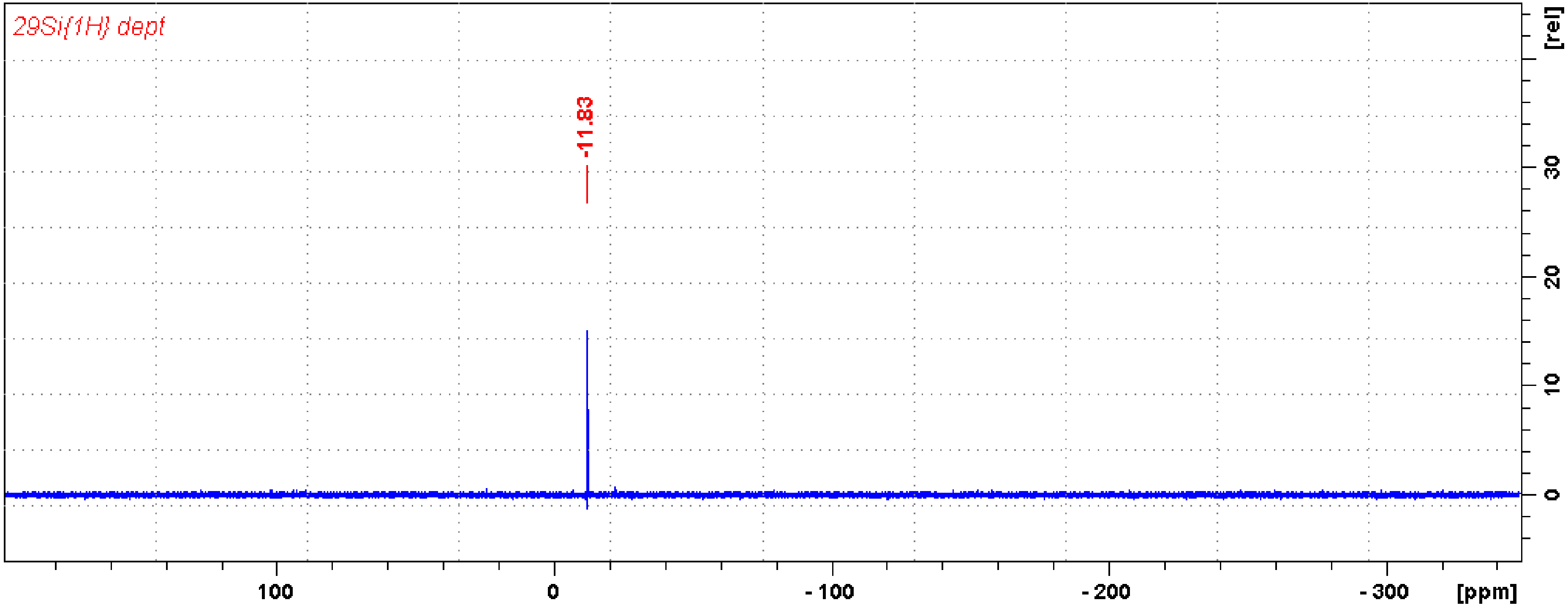

(3*R*,1*E*)-(3,7-Dimethylocta-1,6-dien-1-yl)dimethyl(phenyl)silane (3ai):

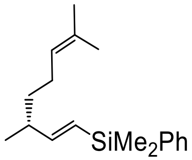

3ai

Figure S112. ^{1}H NMR (500 MHz, $CDCl_3$, 298 K) of **3ai**.

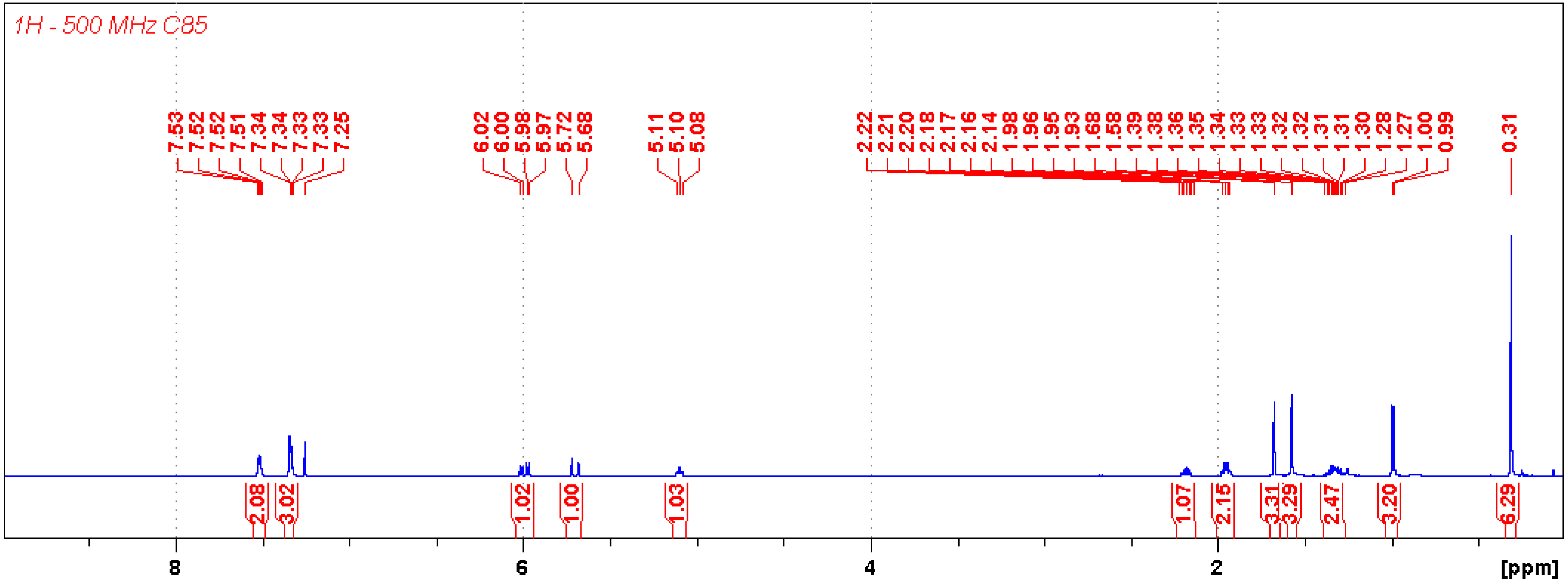

Figure S113. ^{13}C NMR (126 MHz, $CDCl_3$, 298 K) of **3ai**.

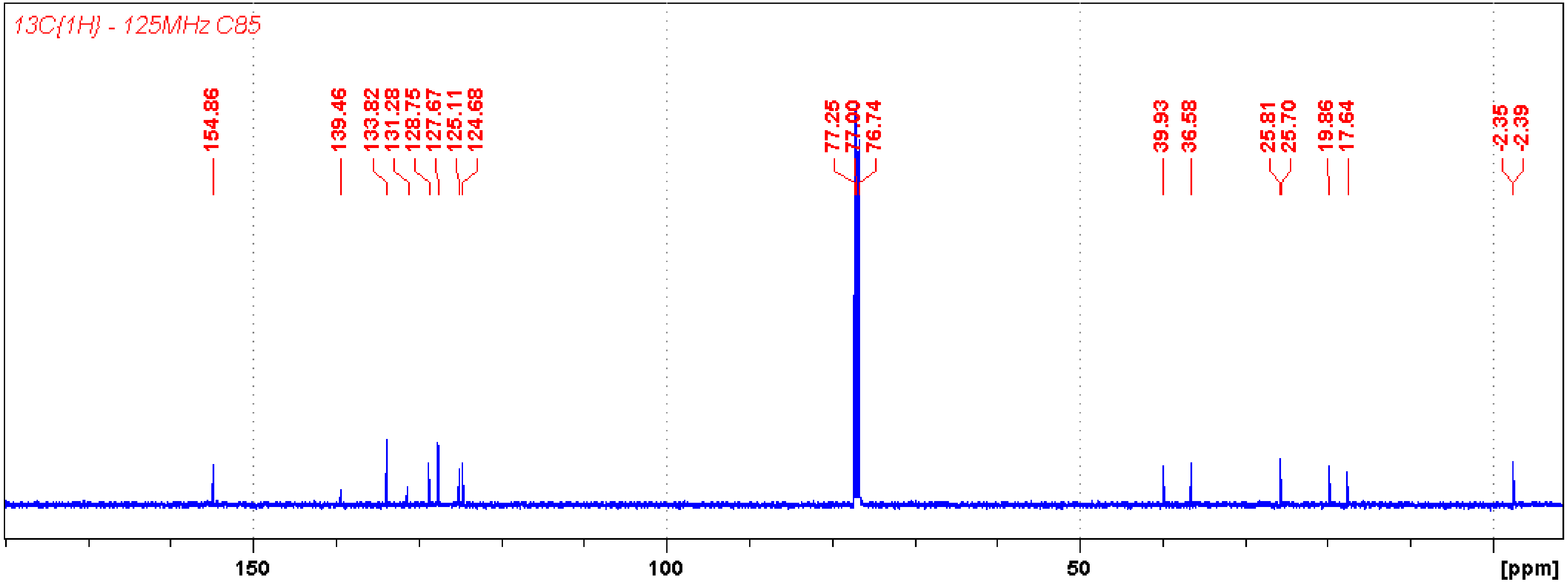

Figure S114. ^{29}Si DEPT NMR (99 MHz, $CDCl_3$, 298 K) of **3ai**.

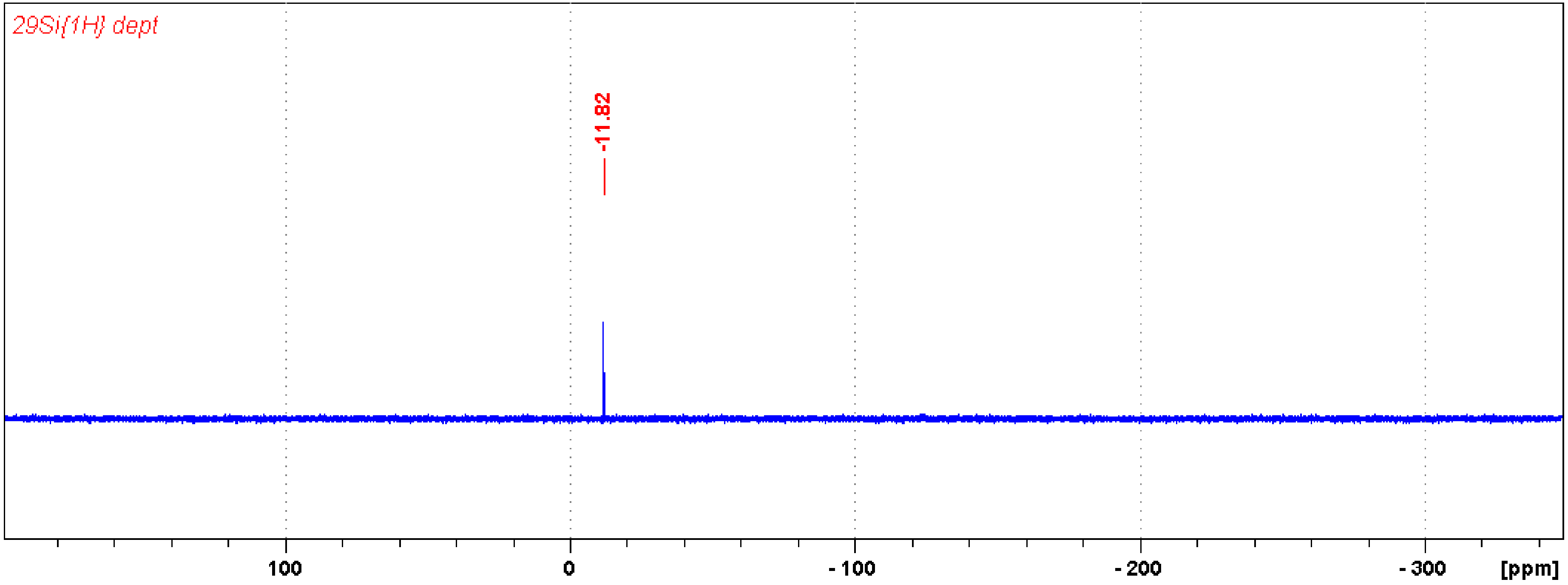

(3*R*,1*E*)-*tert*-Butyl((6-(dimethyl(phenyl)silyl)-4-methylhex-5-en-1-yl)oxy) dimethylsilane (3aj):

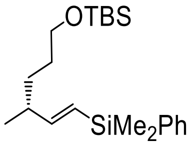

3aj

Figure S115. ^{1}H NMR (500 MHz, $CDCl_3$, 298 K) of **3aj**.

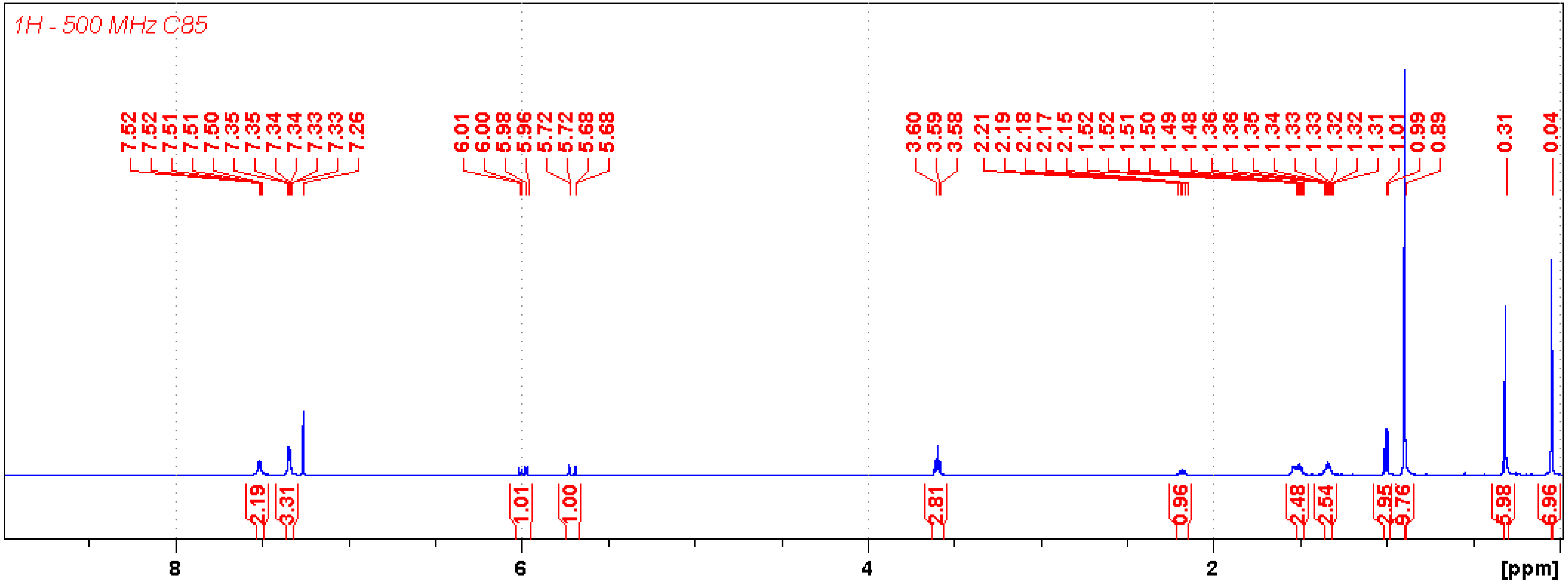

Figure S116. ^{13}C NMR (126 MHz, $CDCl_3$, 298 K) of **3aj**.

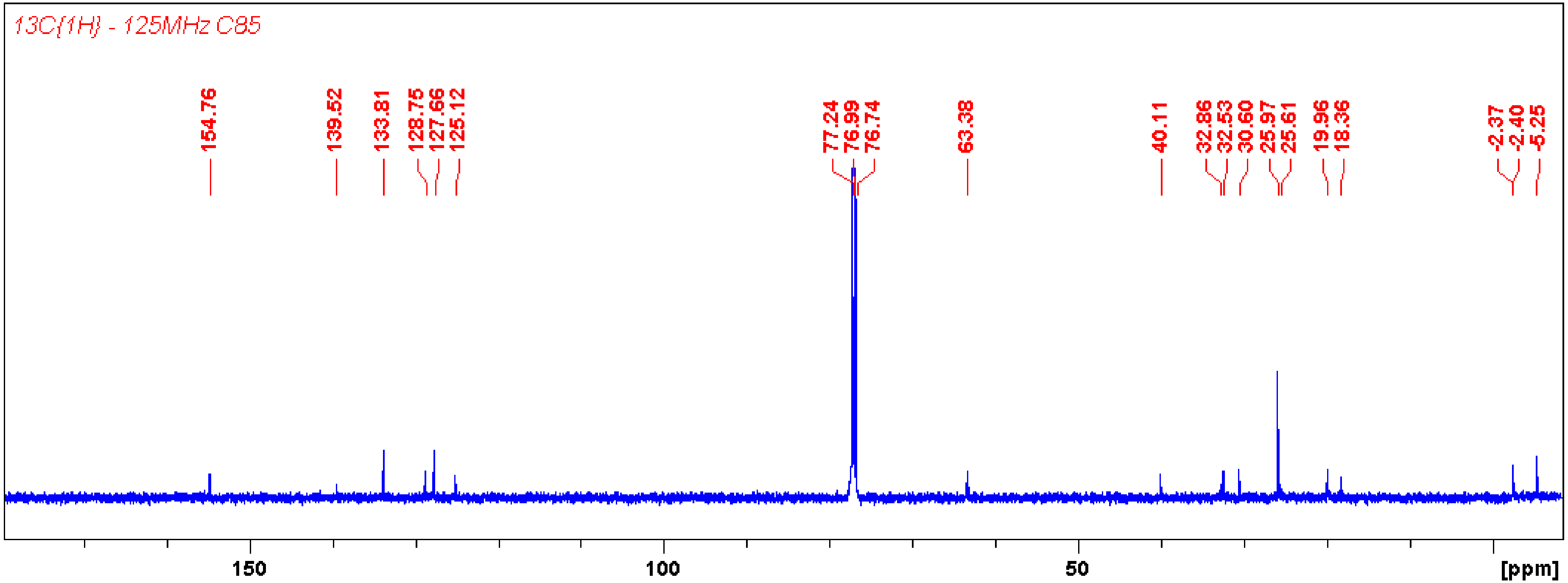

Figure S117. ^{29}Si DEPT NMR (99 MHz, $CDCl_3$, 298 K) of **3aj**.

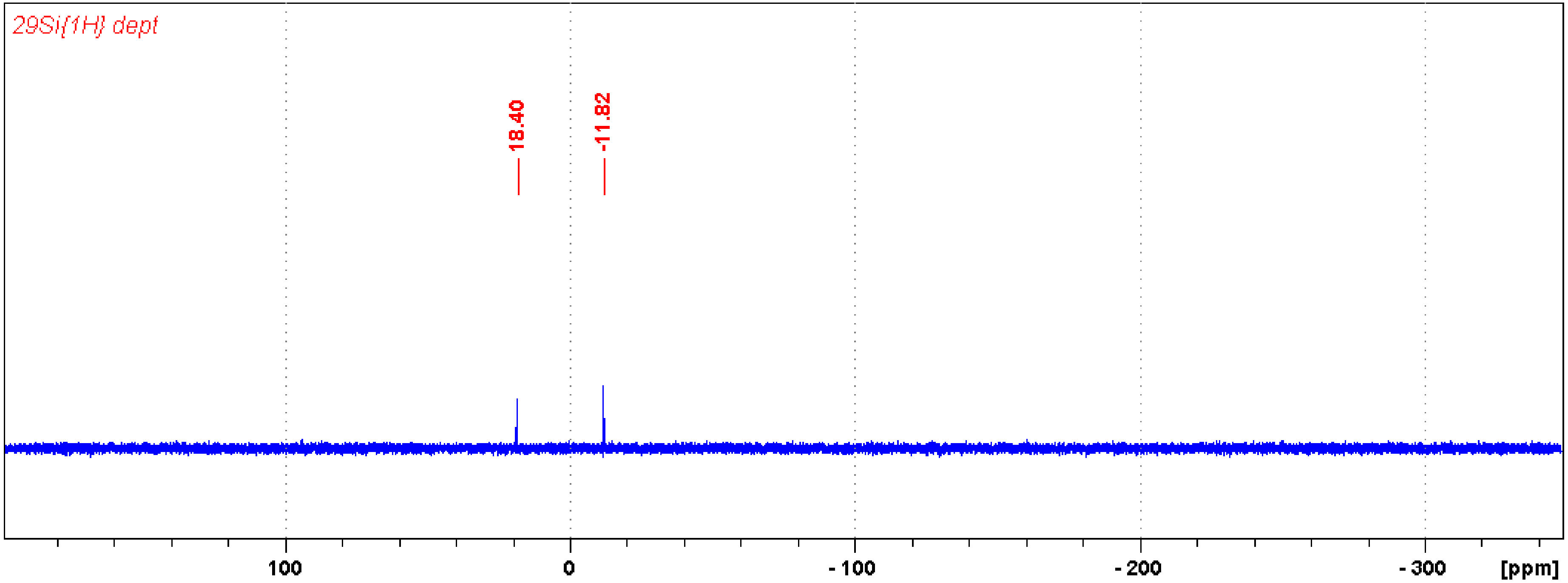

(3*R*,1*E*)-(5-(1,3-Dioxolan-2-yl)-3-methylpent-1-en-1-yl)(methyl)diphenylsilane (**8aa**):

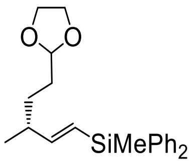

8aa

Figure S118. ^{1}H NMR (500 MHz, $CDCl_3$, 298 K) of **8aa**.

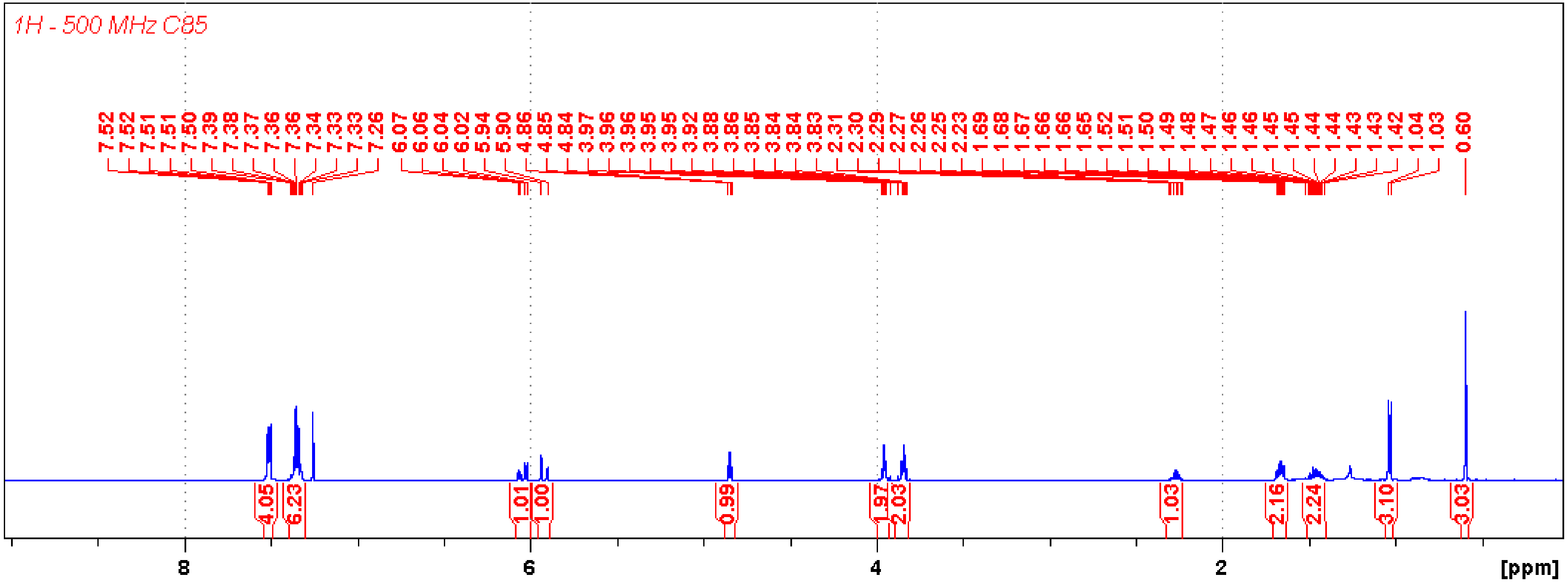

Figure S119. ^{13}C NMR (126 MHz, $CDCl_3$, 298 K) of **8aa**.

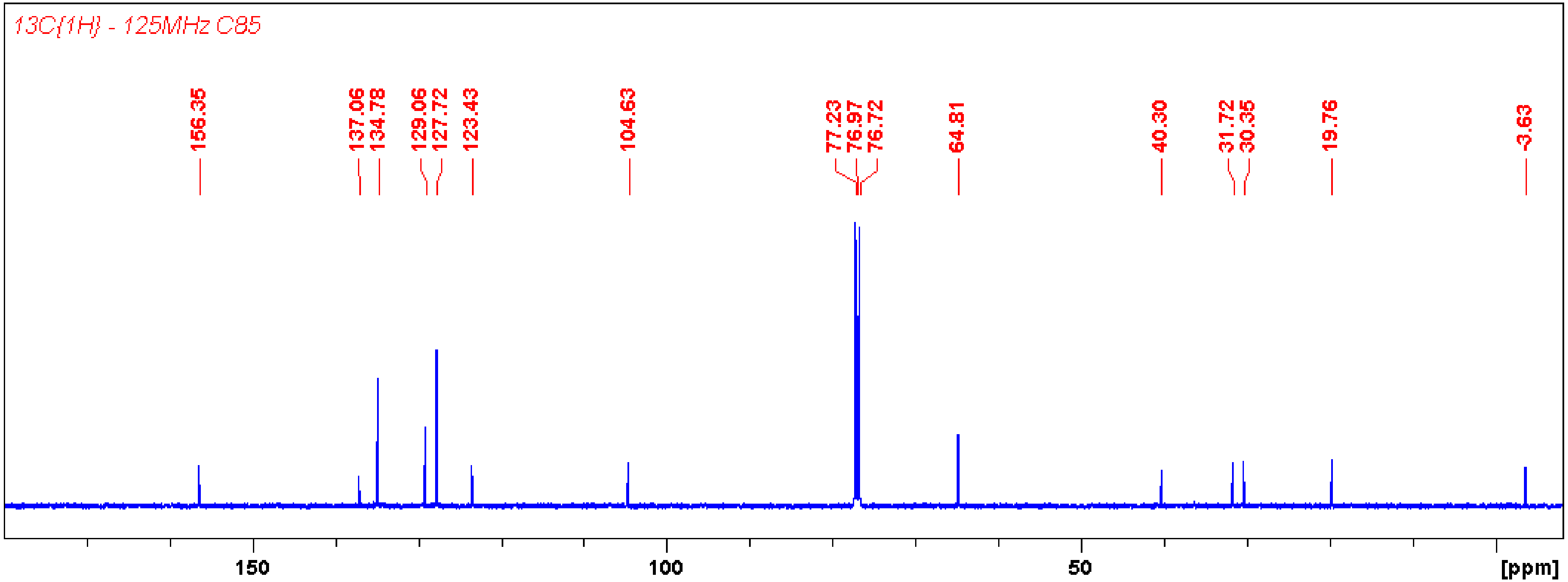

Figure S120. ^{29}Si DEPT NMR (99 MHz, $CDCl_3$, 298 K) of **8aa**.

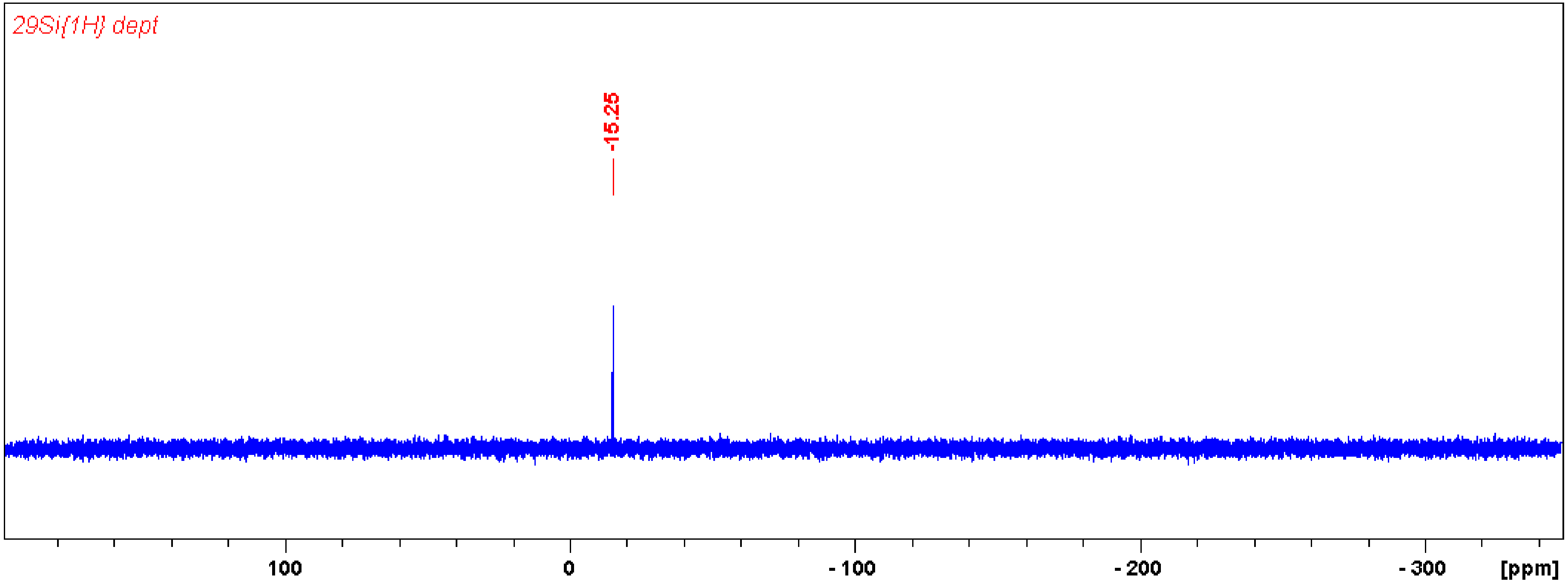

(3*R*,1*E*)-(3-(2-(1,3-Dioxolan-2-yl)ethyl)hex-1-en-1-yl)(methyl)diphenylsilane (**8ba**):

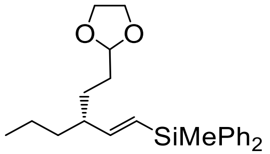

8ba

Figure S121. ^{1}H NMR (500 MHz, $CDCl_3$, 298 K) of **8ba**.

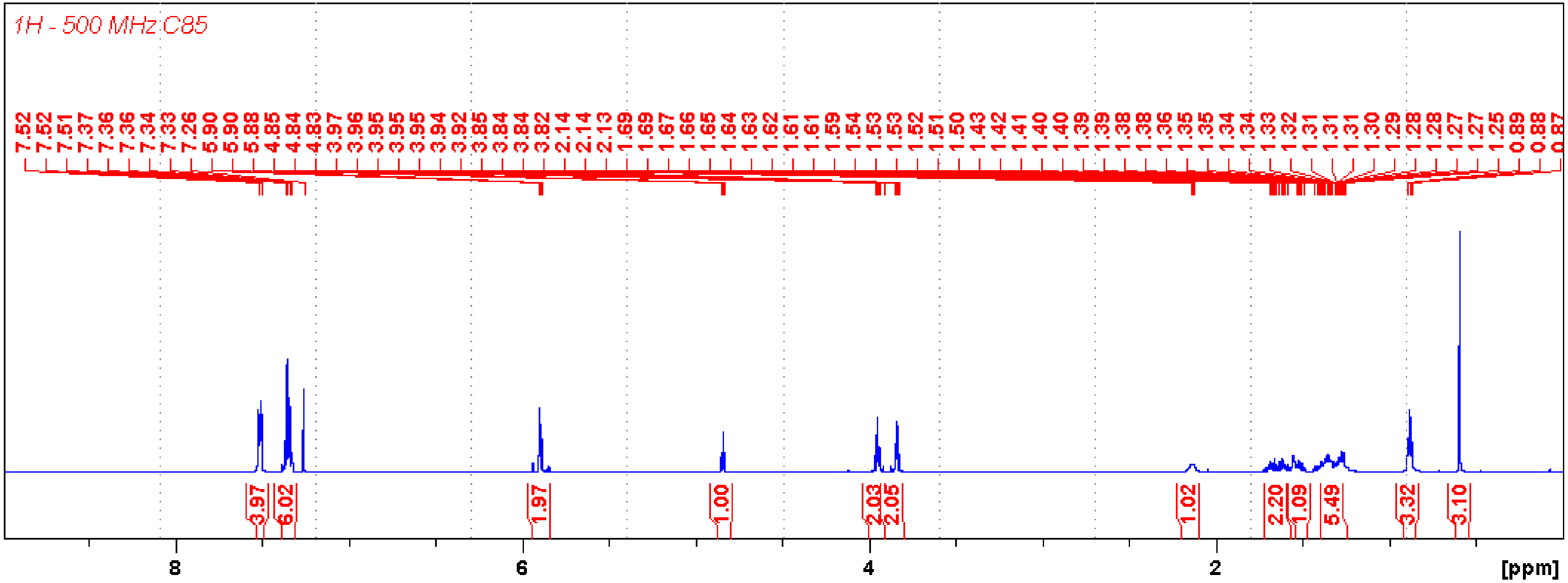

Figure S122. ^{13}C NMR (126 MHz, $CDCl_3$, 298 K) of **8ba**.

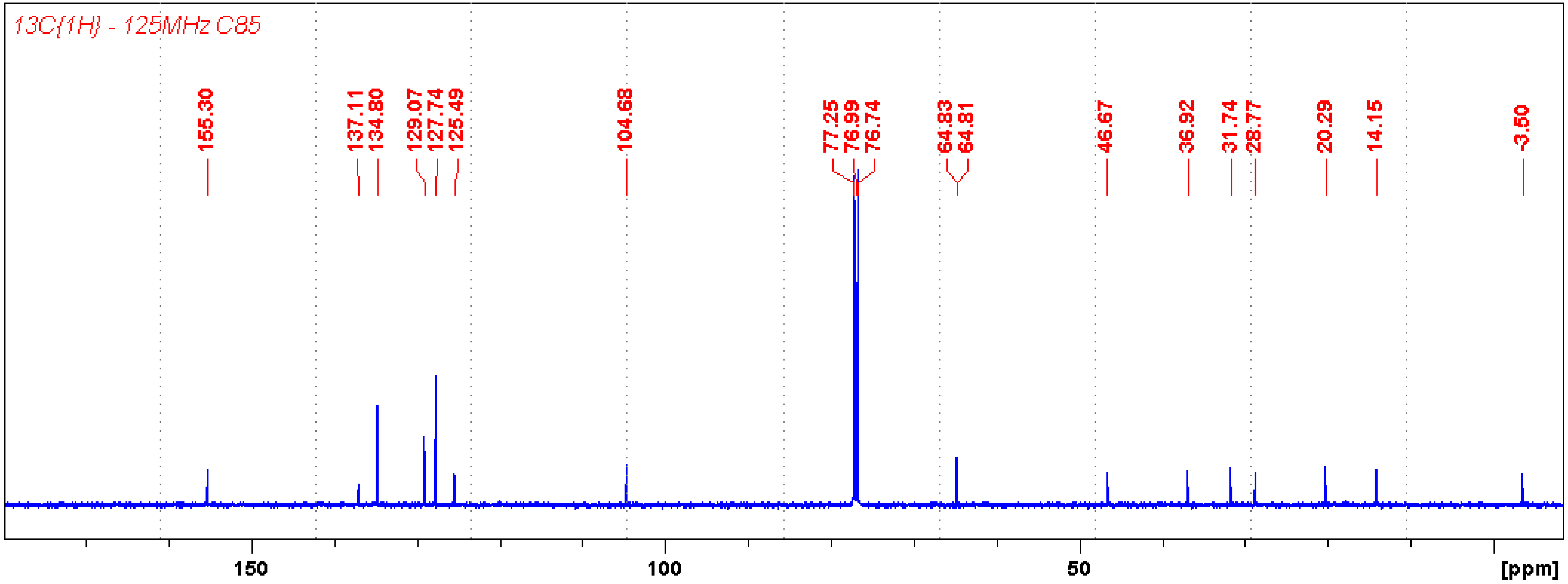

Figure S123. ^{29}Si DEPT NMR (99 MHz, $CDCl_3$, 298 K) of **8ba**.

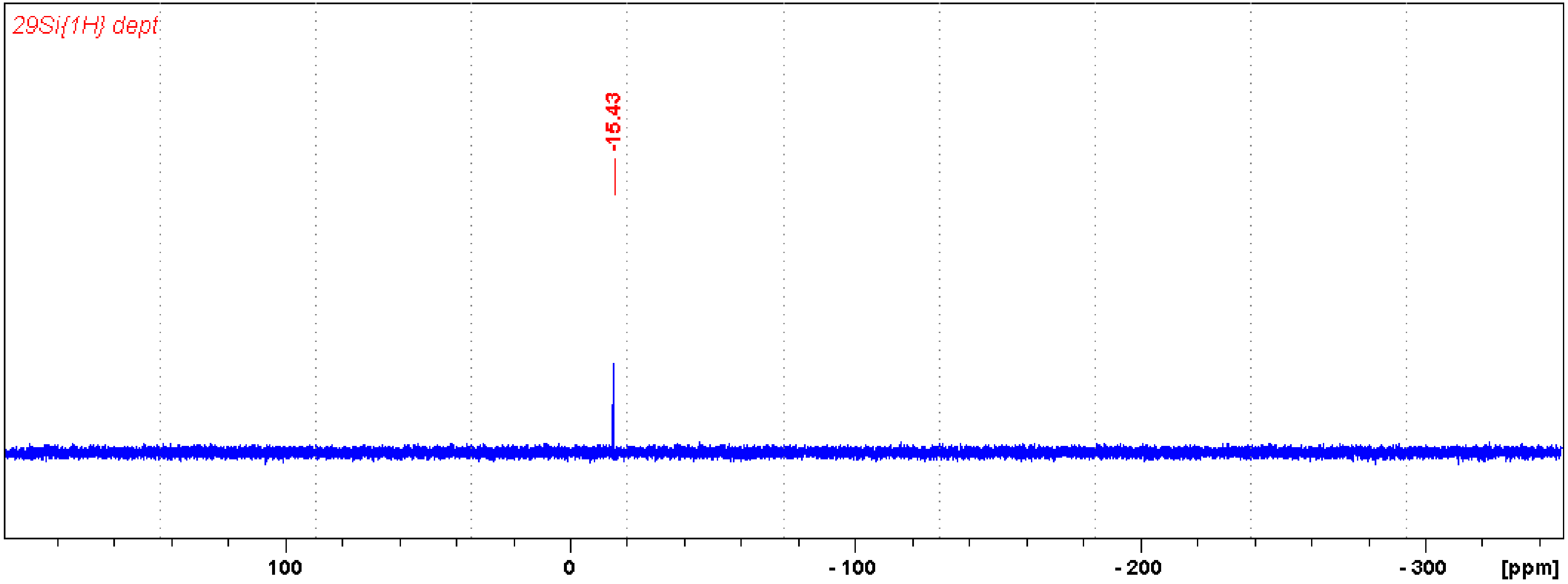

(3*R*,1*E*)-(5-(1,3-Dioxolan-2-yl)-3-methylpent-1-en-1-yl)(tert-butyl) diphenyl silane (9aa):

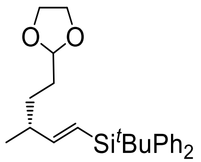

9aa

Figure S124. ^{1}H NMR (500 MHz, $CDCl_3$, 298 K) of **9aa**.

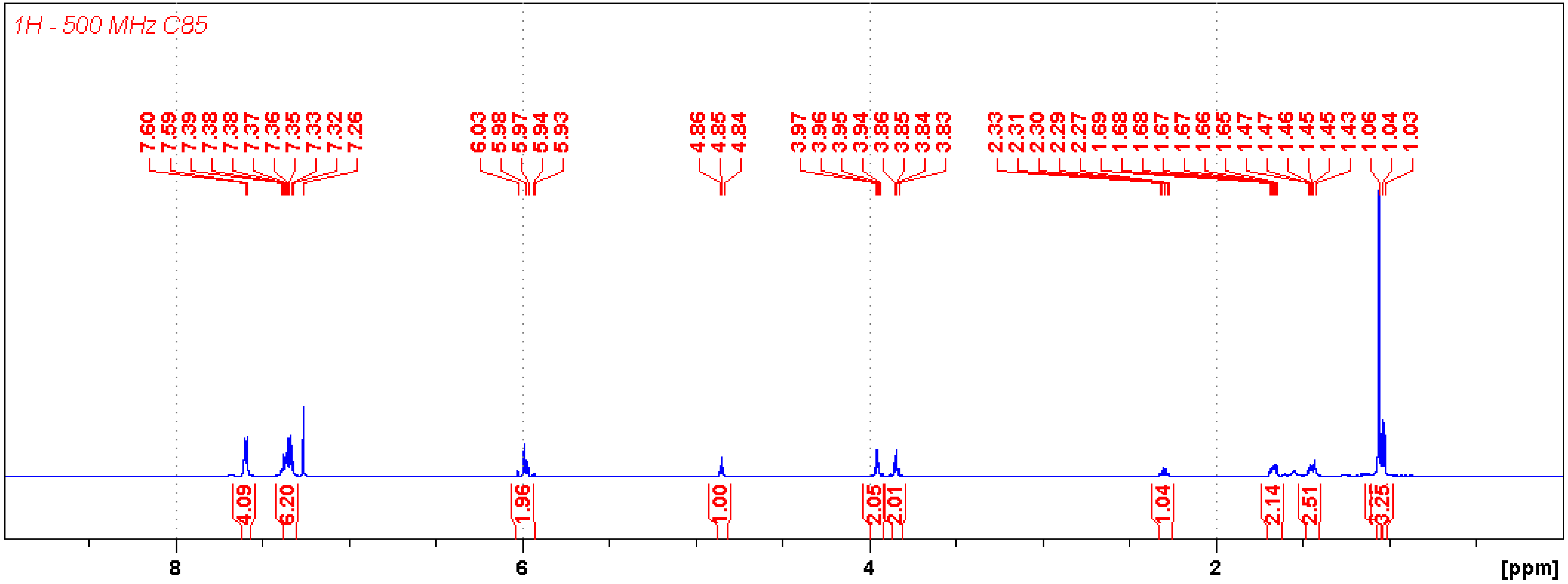

Figure S125. ^{13}C NMR (126 MHz, $CDCl_3$, 298 K) of **9aa**.

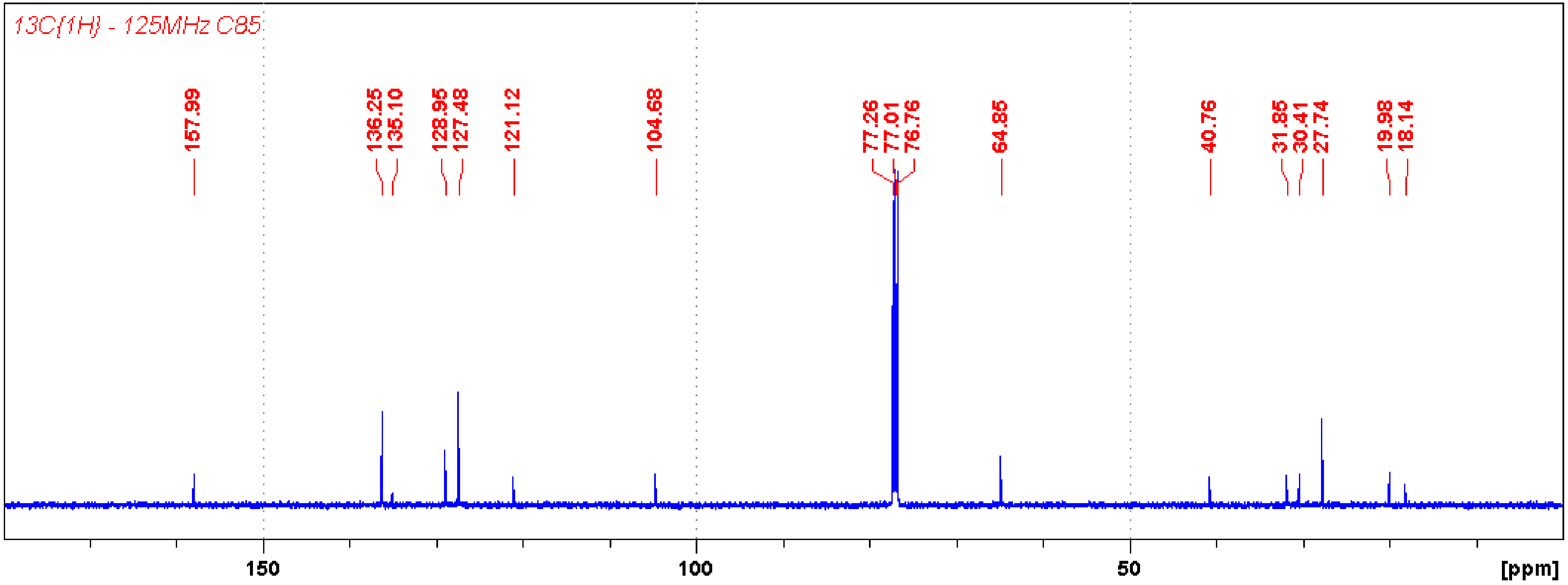

Figure S126. ^{29}Si DEPT NMR (99 MHz, $CDCl_3$, 298 K) of **9aa**.

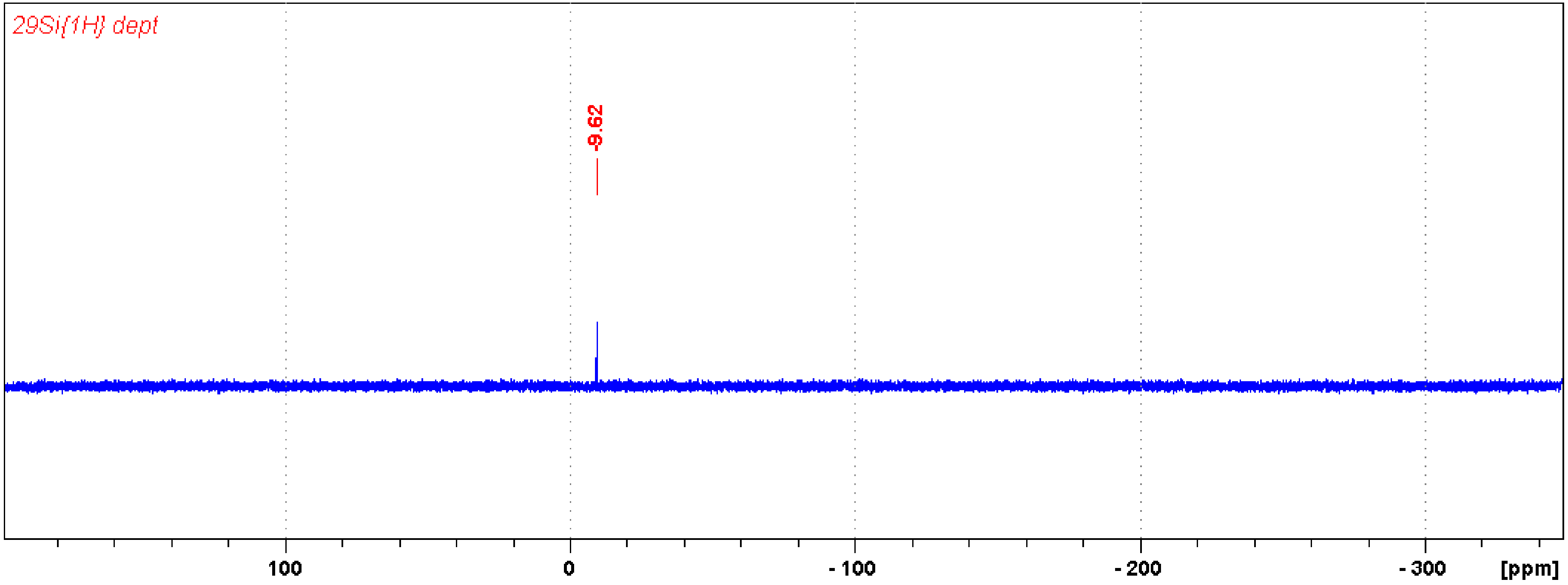

(3*R*,1*E*)-(5-(1,3-Dioxolan-2-yl)-3-methylpent-1-en-1-yl)trimethylsilane (10aa):

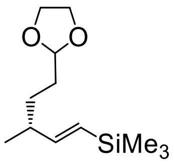

10aa

Figure S127. ^{1}H NMR (500 MHz, CDCl$_3$, 298 K) of **10aa**.

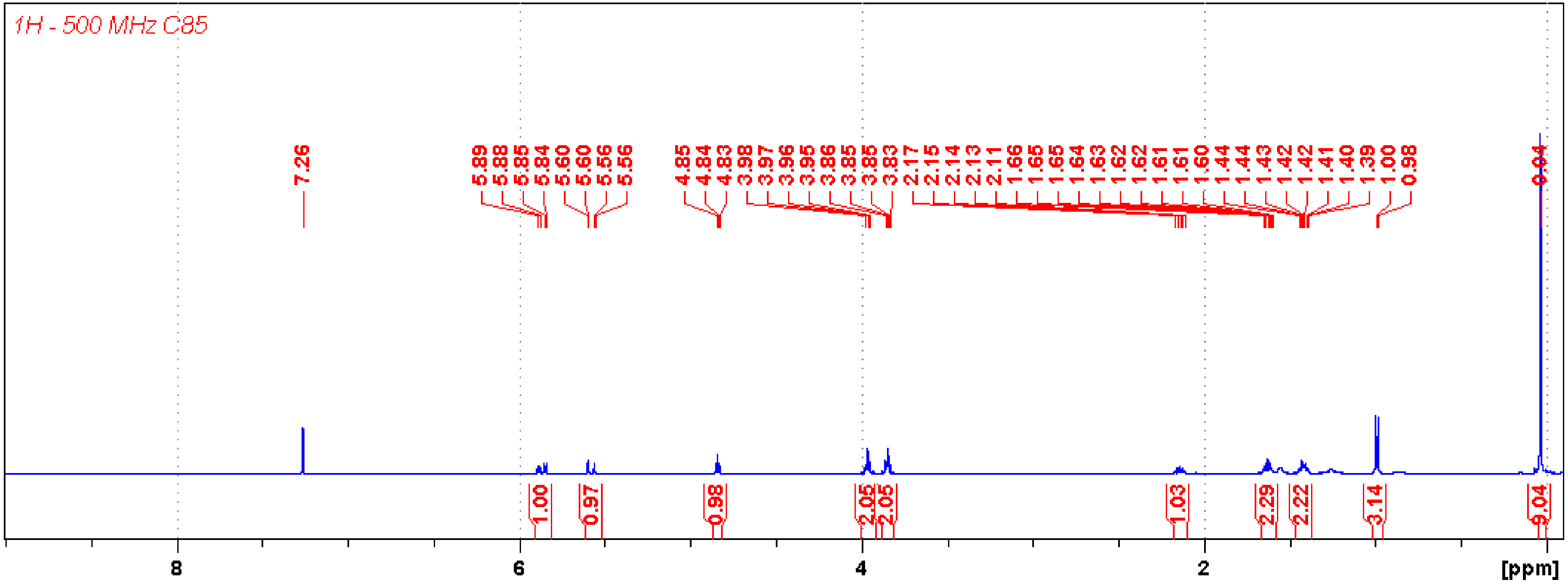

Figure S128. ^{13}C NMR (126 MHz, $CDCl_3$, 298 K) of **10aa**.

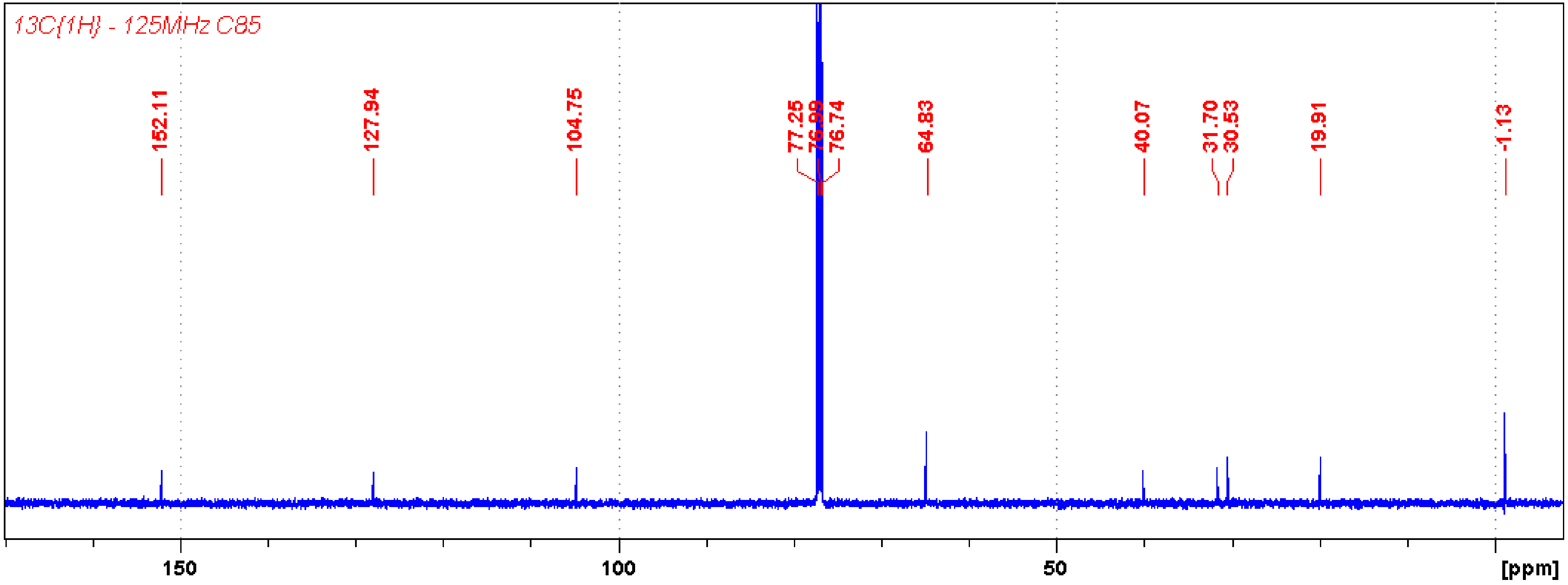

Figure S129. ^{29}Si DEPT NMR (99 MHz, $CDCl_3$, 298 K) of **10aa**.

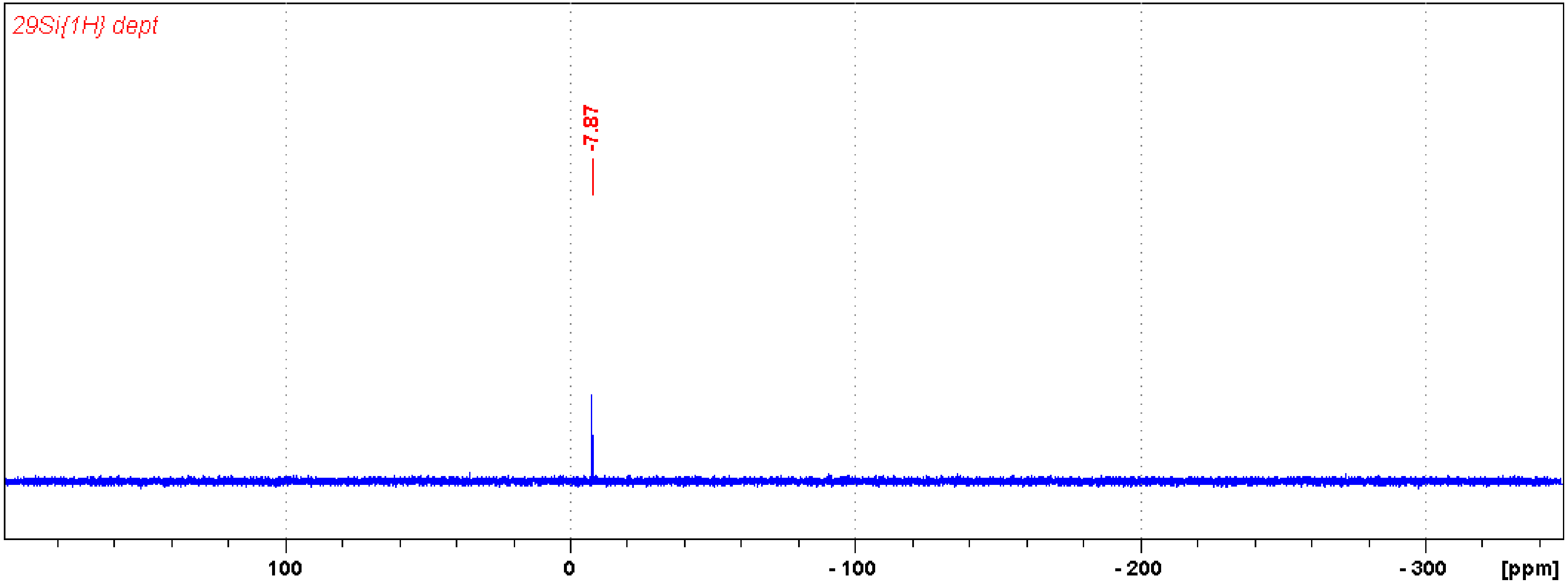

(3*R*,1*E*)- (5-(1,3-Dioxolan-2-yl)-3-methylpent-1-en-1-yl)(benzyl)dimethylsilane (**11aa**):

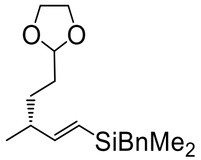

11aa

Figure S130. ^{1}H NMR (500 MHz, $CDCl_3$, 298 K) of **11aa**.

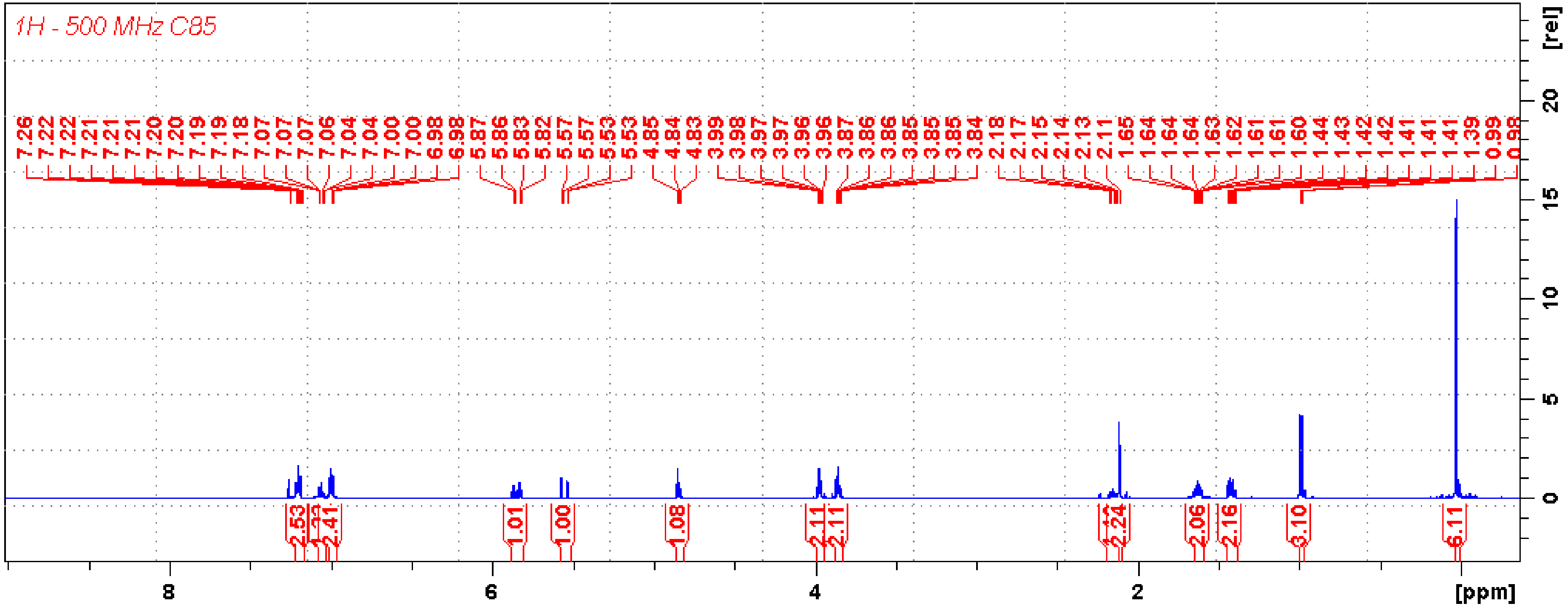

Figure S131. ^{13}C NMR (126 MHz, $CDCl_3$, 298 K) of **11aa**.

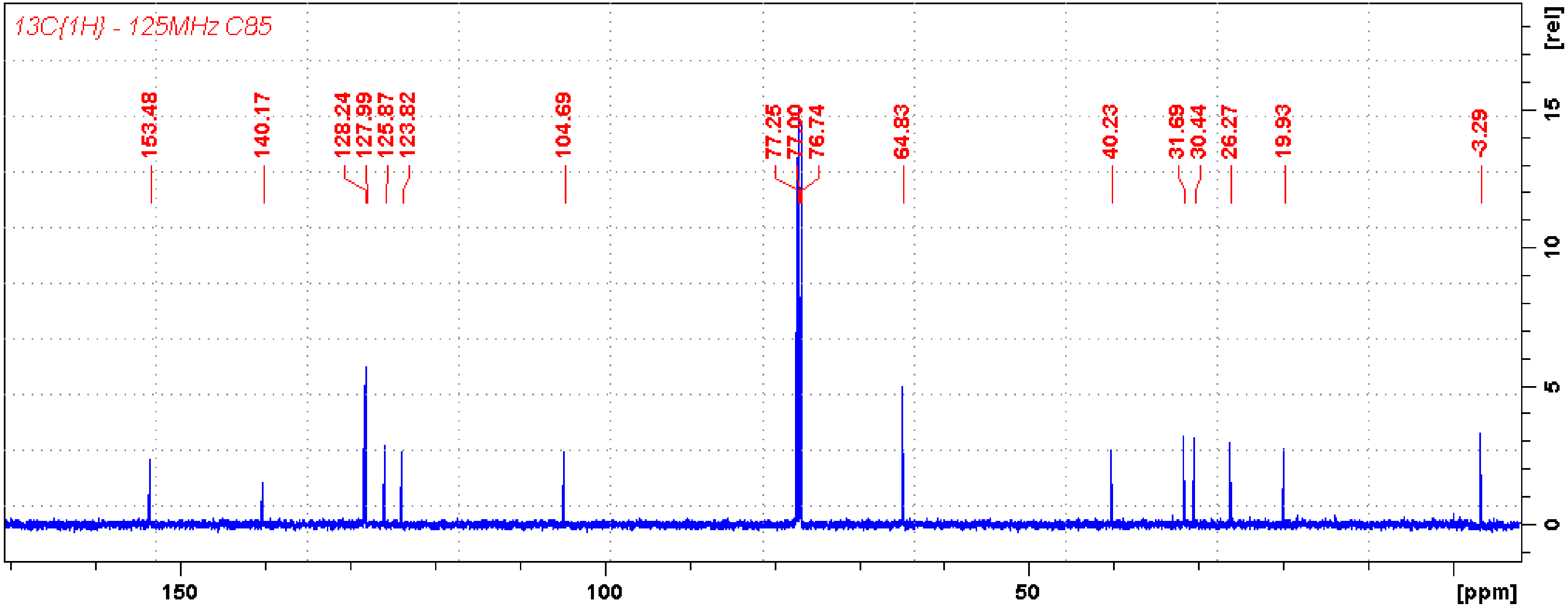

Figure S132. ^{29}Si DEPT NMR (99 MHz, $CDCl_3$, 298 K) of **11aa**.

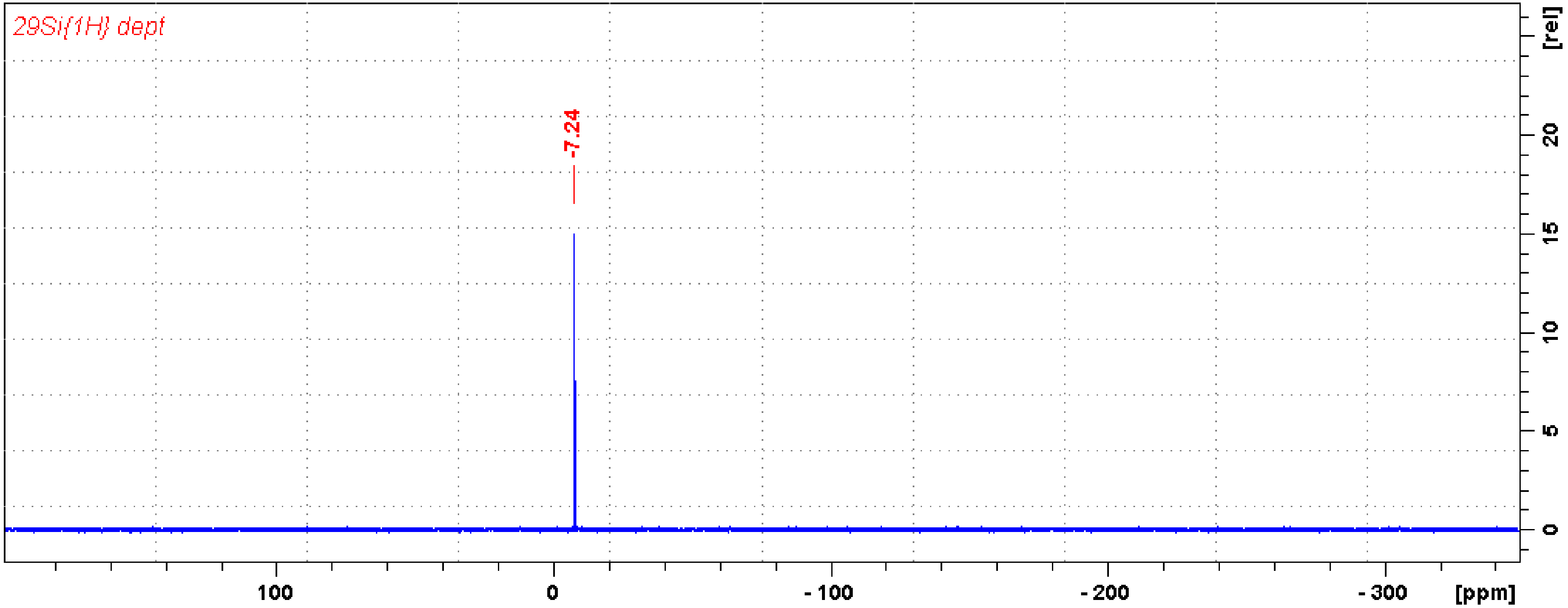

(3*R*,1*E*)-(3-(2-(1,3-Dioxolan-2-yl)ethyl)hept-1-en-1-yl) (benzyl)dimethylsilane (**11ba**):

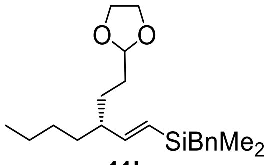

11ba

Figure S133. ^{1}H NMR (500 MHz, $CDCl_3$, 298 K) of **11ba**.

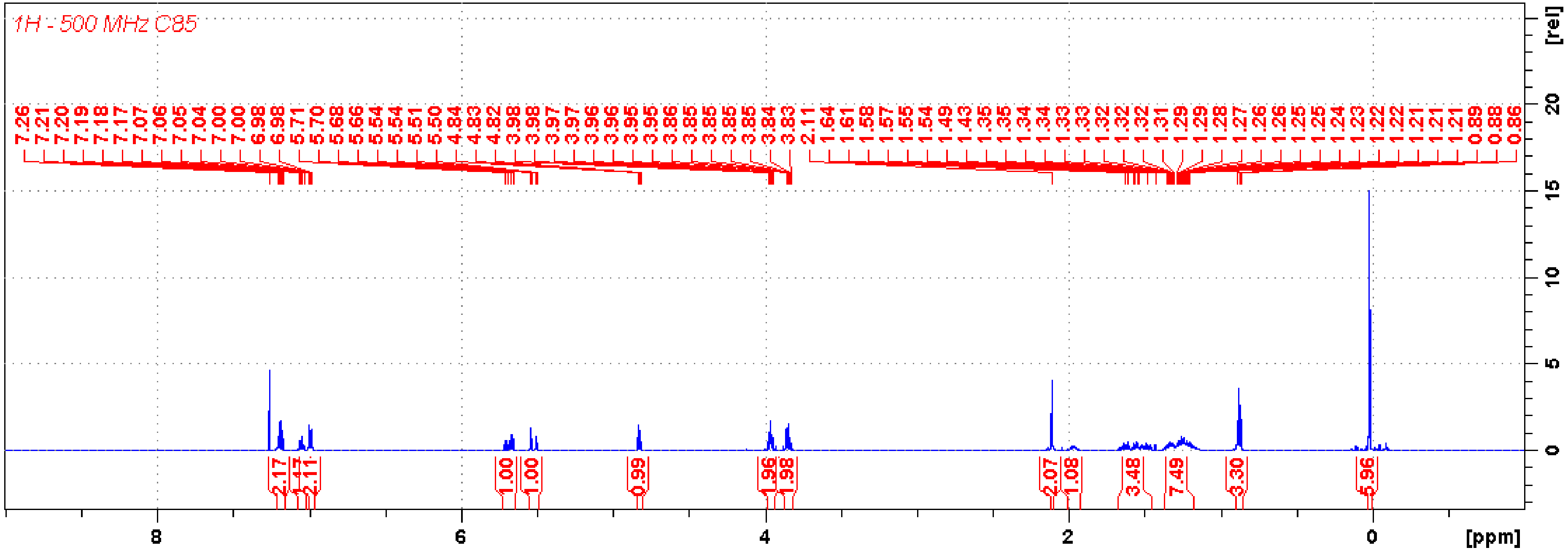

Figure S134. ^{13}C NMR (126 MHz, $CDCl_3$, 298 K) of **11ba**.

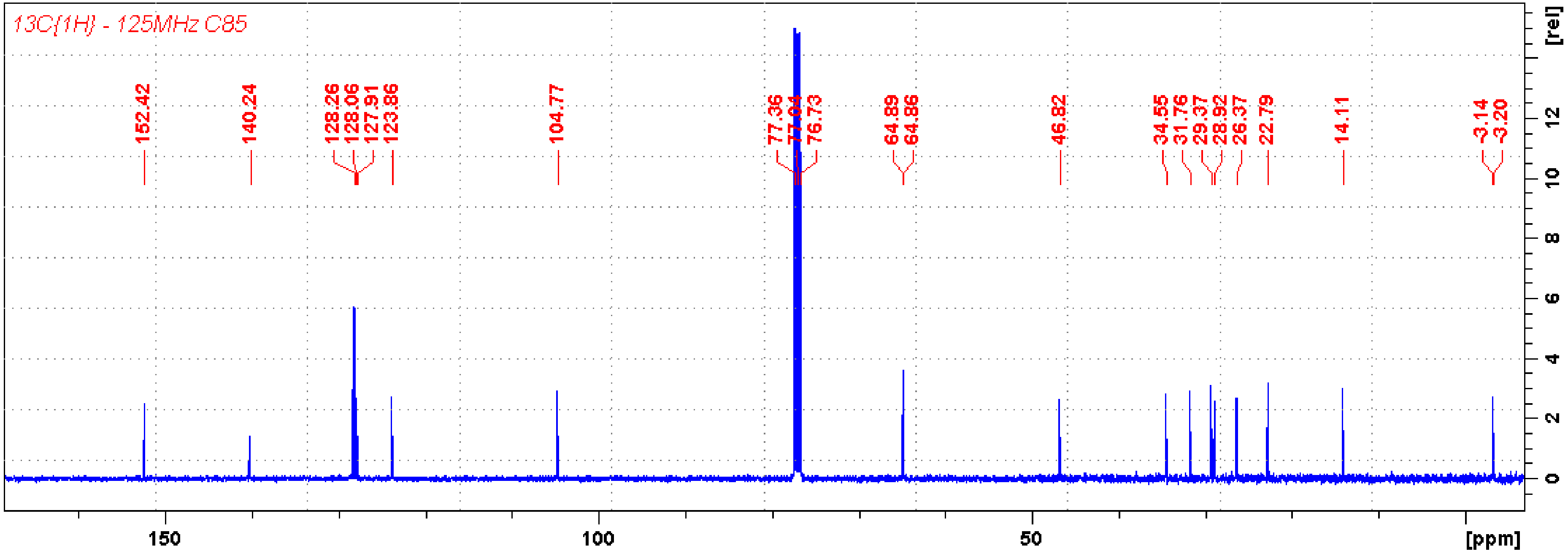

Figure S135. ^{29}Si DEPT NMR (99 MHz, $CDCl_3$, 298 K) of **11ba**.

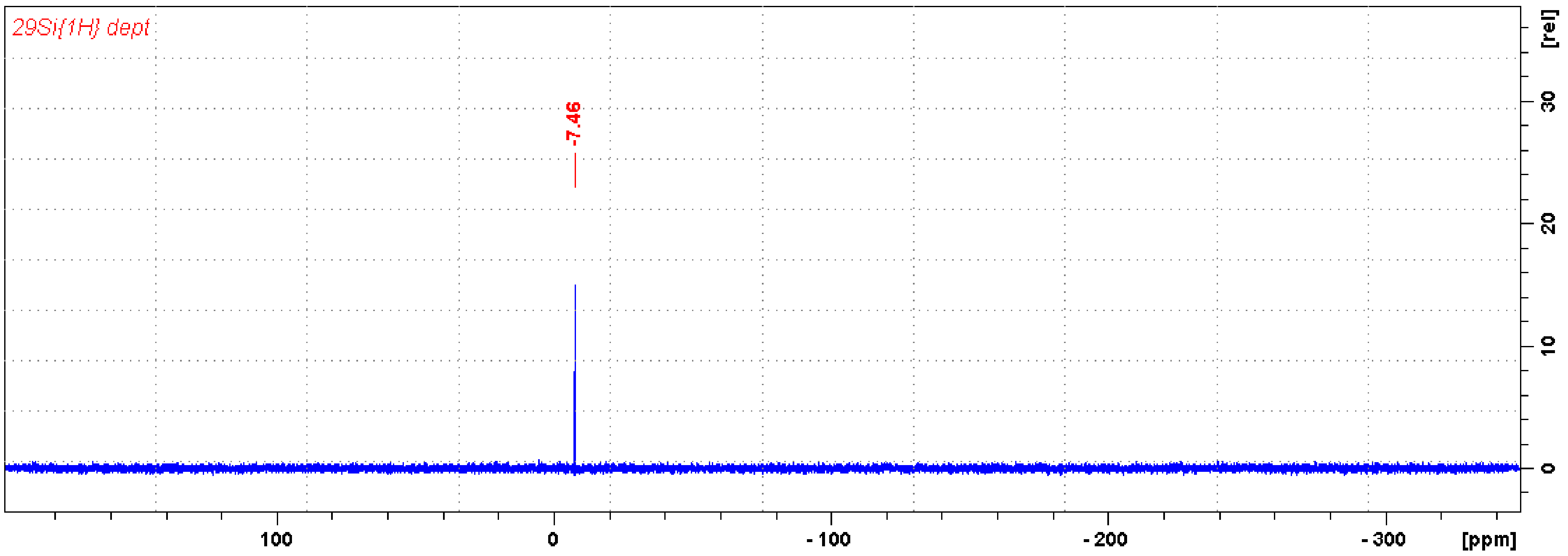

(3*R*,1*E*)-(5-(1,3-Dioxolan-2-yl)-2,3-dimethylpent-1-en-1-yl)(methyl)diphenylsilane (8ca):

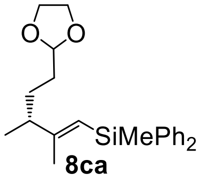

8ca

Figure S136. ^1H NMR (500 MHz, $CDCl_3$, 298 K) of **8ca**.

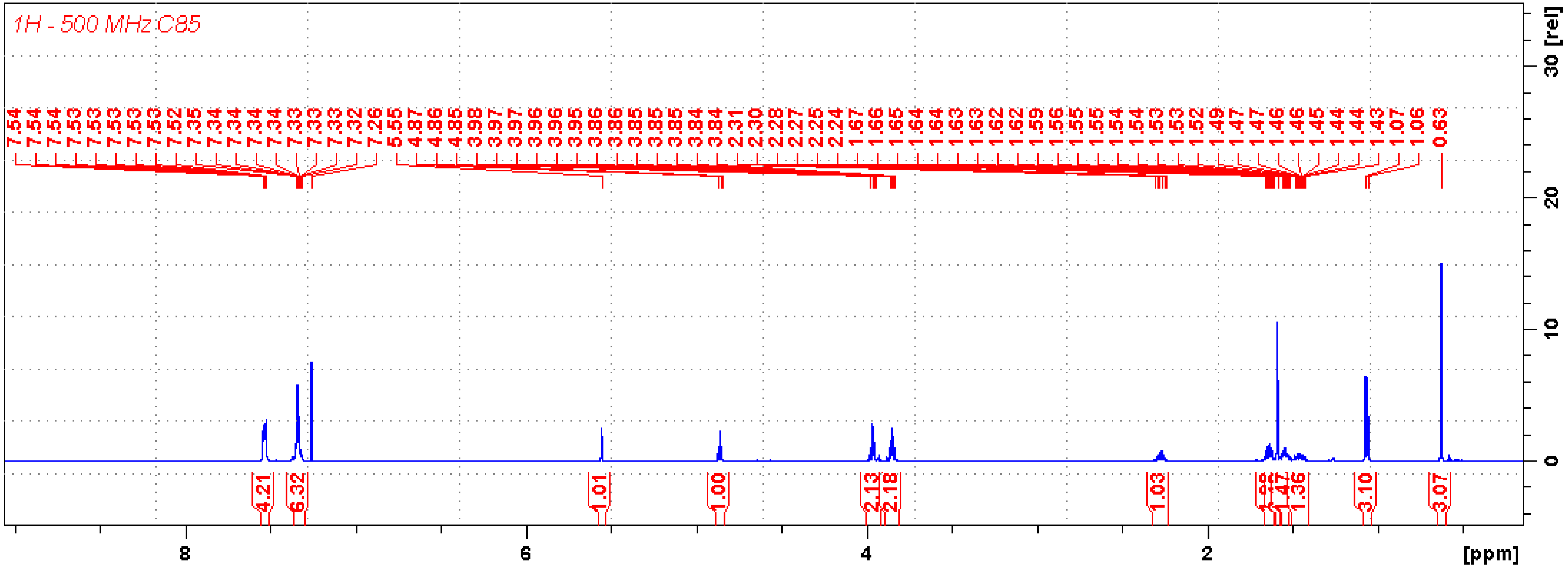

Figure S137. ^{13}C NMR (126 MHz, $CDCl_3$, 298 K) of **8ca**.

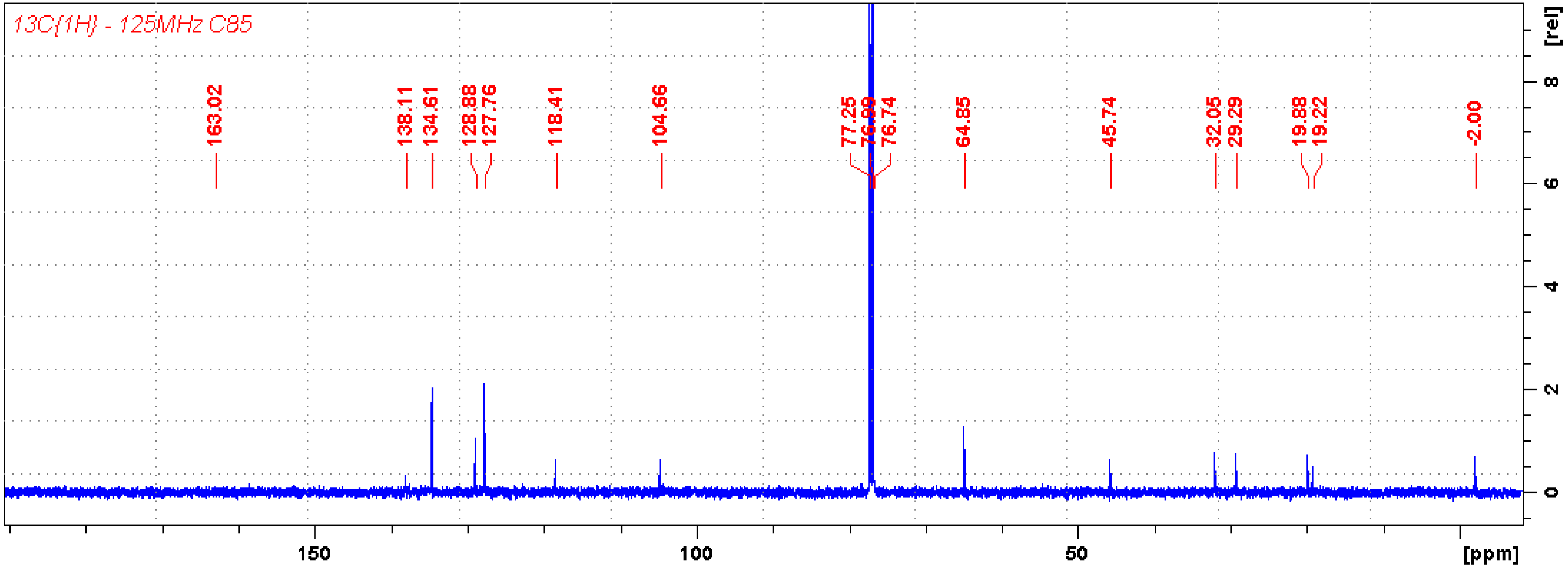

Figure S138. ^{29}Si DEPT NMR (99 MHz, $CDCl_3$, 298 K) of **8ca**.

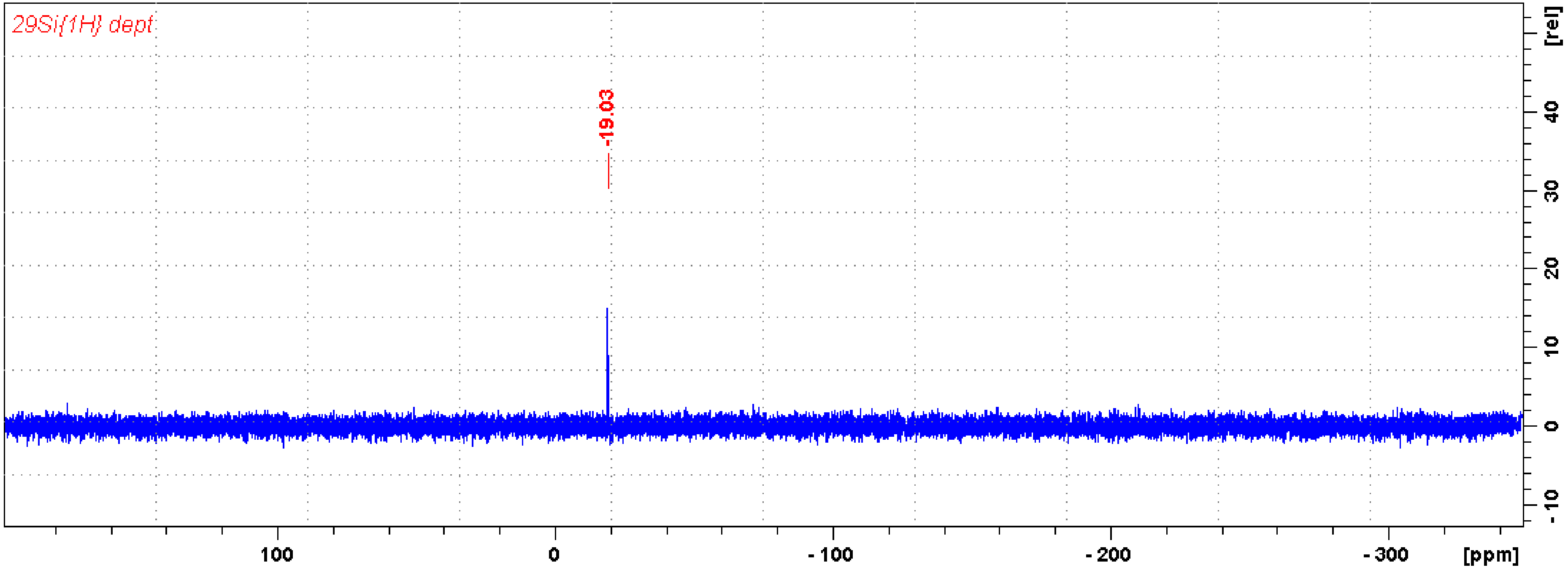

(3*R*,1*E*)-(5-(1,3-Dioxan-2-yl)-2,3-dimethylpent-1-en-1-yl)(methyl)diphenylsilane (**8cb**):

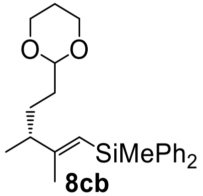

8cb

Figure S139. ^{1}H NMR (500 MHz, $CDCl_3$, 298 K) of **8cb**.

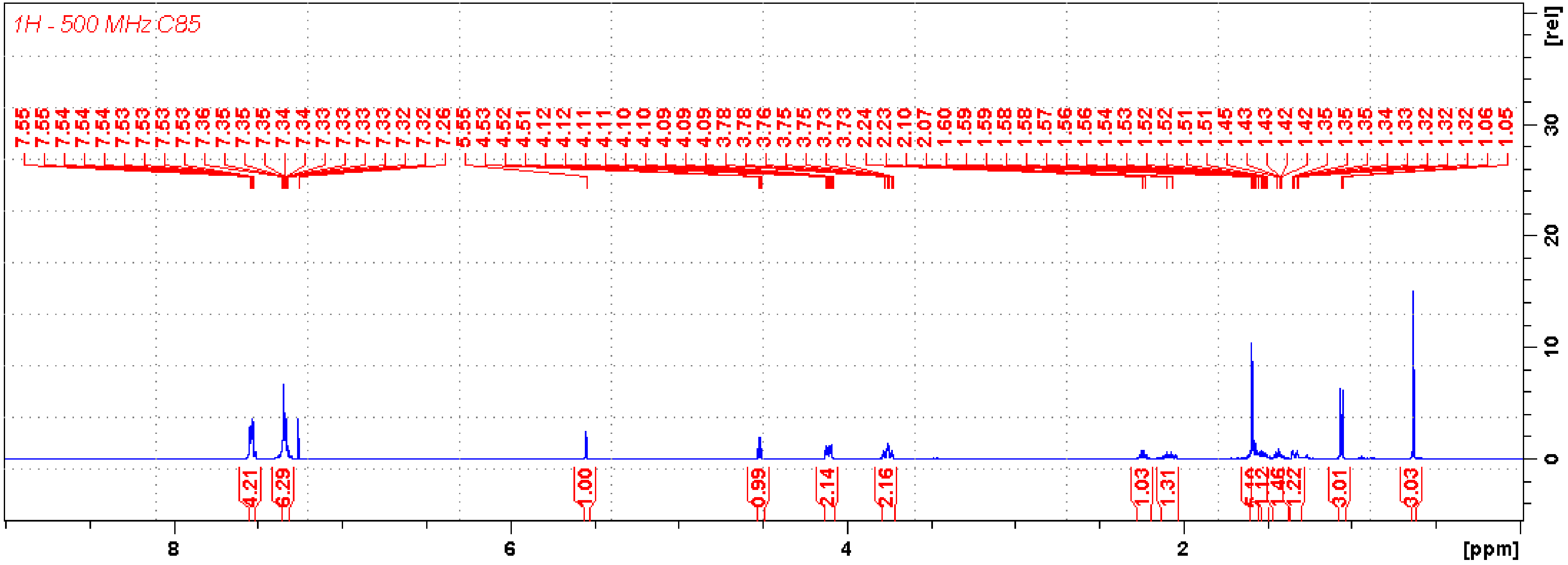

Figure S140. ^{13}C NMR (126 MHz, $CDCl_3$, 298 K) of **8cb**.

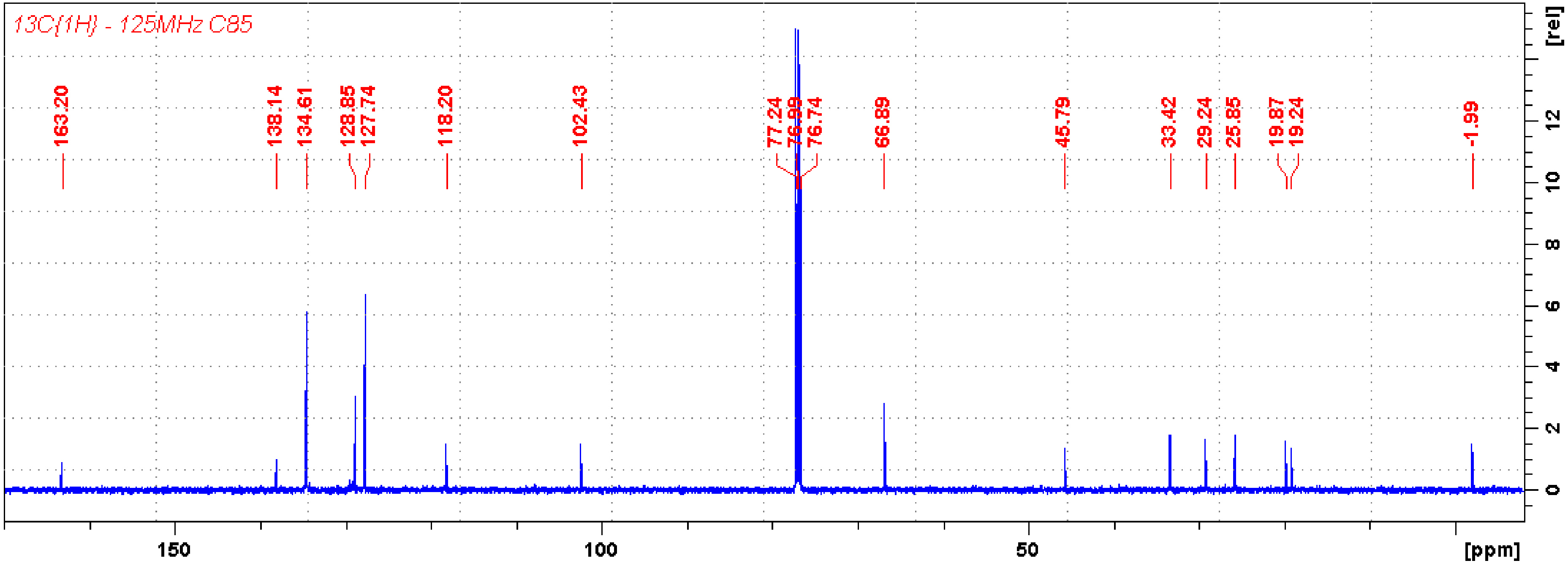

Figure S141. ^{29}Si DEPT NMR (99 MHz, $CDCl_3$, 298 K) of **8cb**.

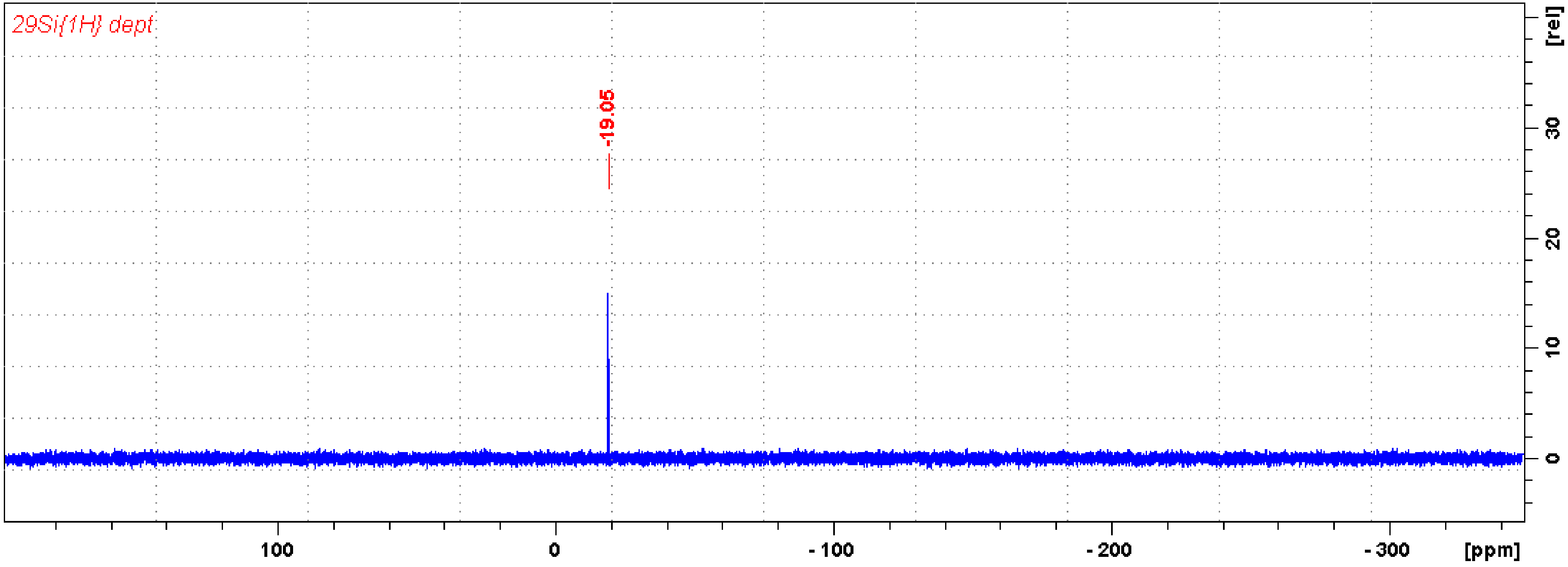

(3*R*,1*E*)-(2,3-Dimethyl-6-phenoxyhex-1-en-1-yl)(methyl)diphenylsilane (**8cc**):

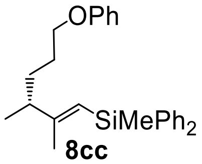

Figure S142. ^{1}H NMR (500 MHz, CDCl$_3$, 298 K) of **8cc**.

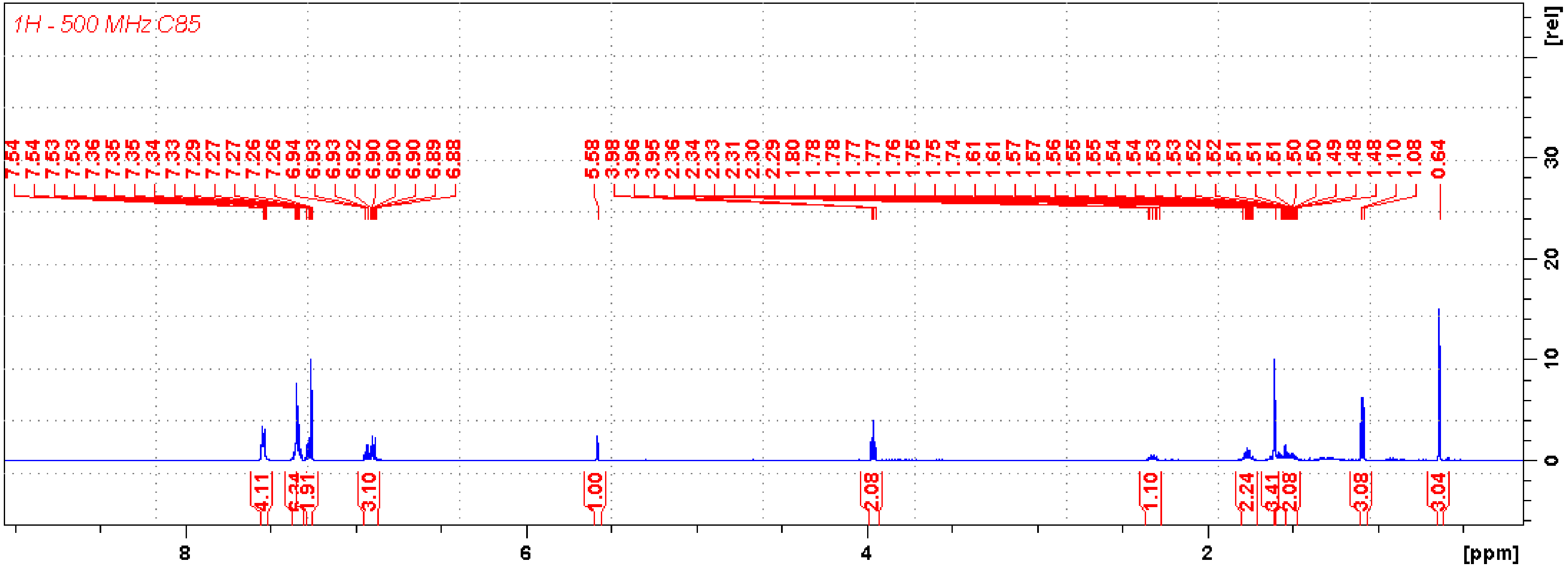

Figure S143. ^{13}C NMR (126 MHz, $CDCl_3$, 298 K) of **8cc**.

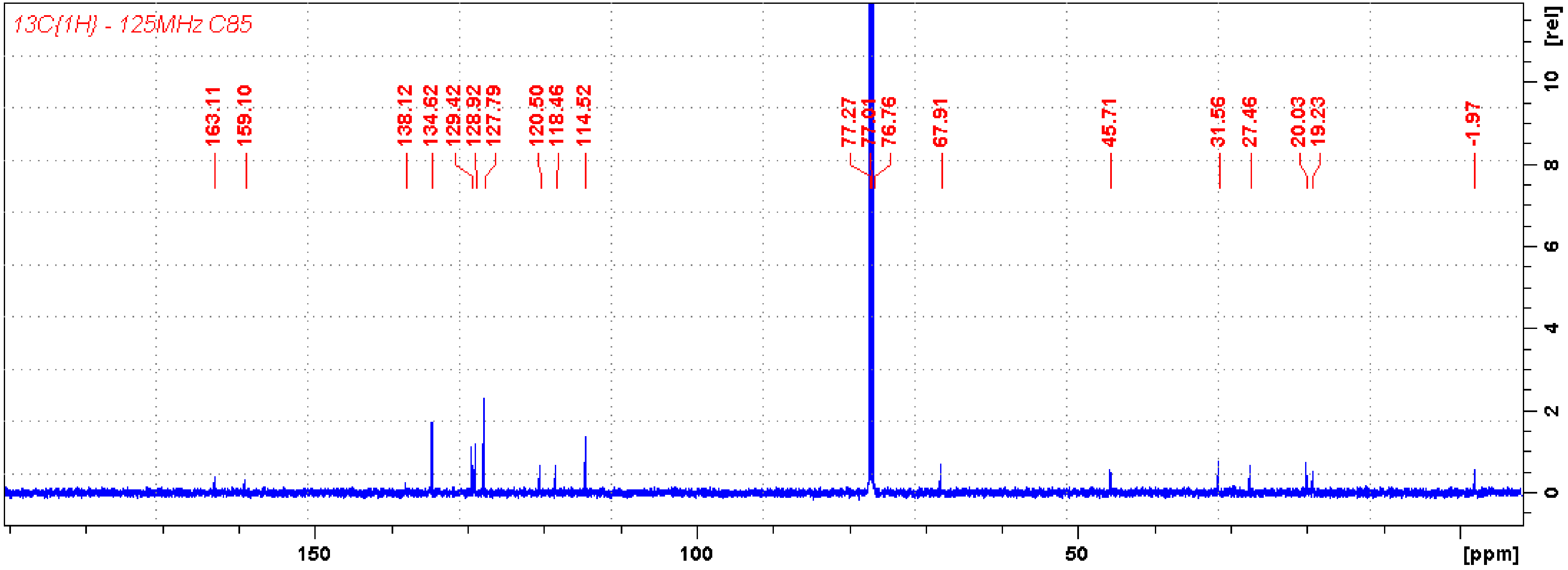

Figure S144. ^{29}Si DEPT NMR (99 MHz, $CDCl_3$, 298 K) of **8cc**.

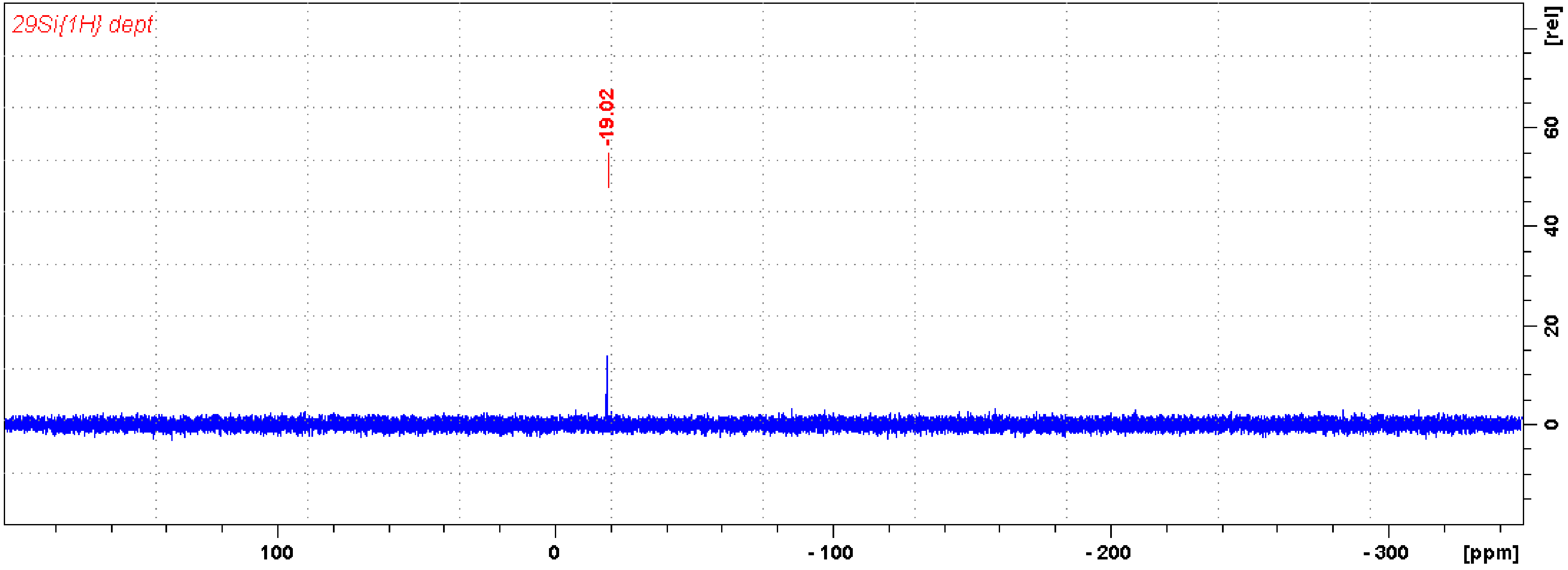

(3*R*,1*E*)-(2,3-Dimethyl-6-phenylhex-1-en-1-yl)(methyl)diphenylsilane (**8cd**):

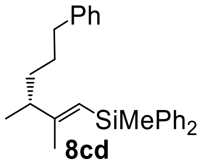

8cd

Figure S145. ^{1}H NMR (500 MHz, $CDCl_3$, 298 K) of **8cd**.

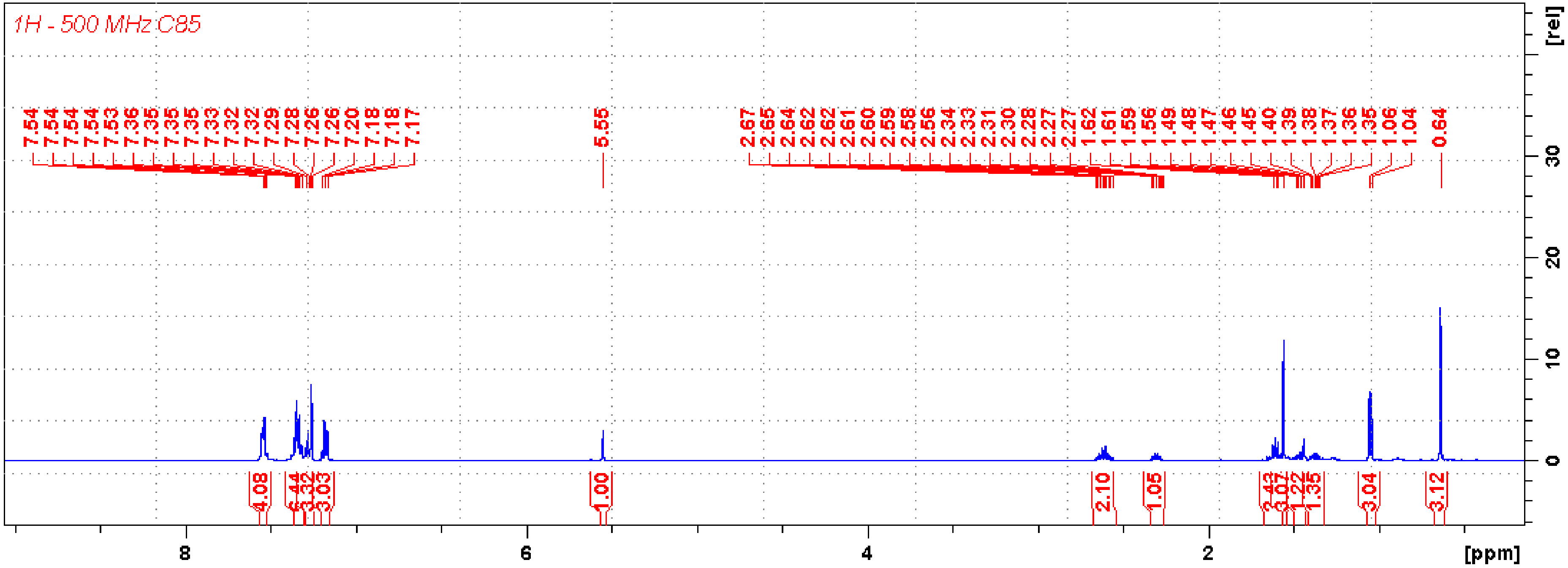

Figure S146. ^{13}C NMR (126 MHz, $CDCl_3$, 298 K) of **8cd**.

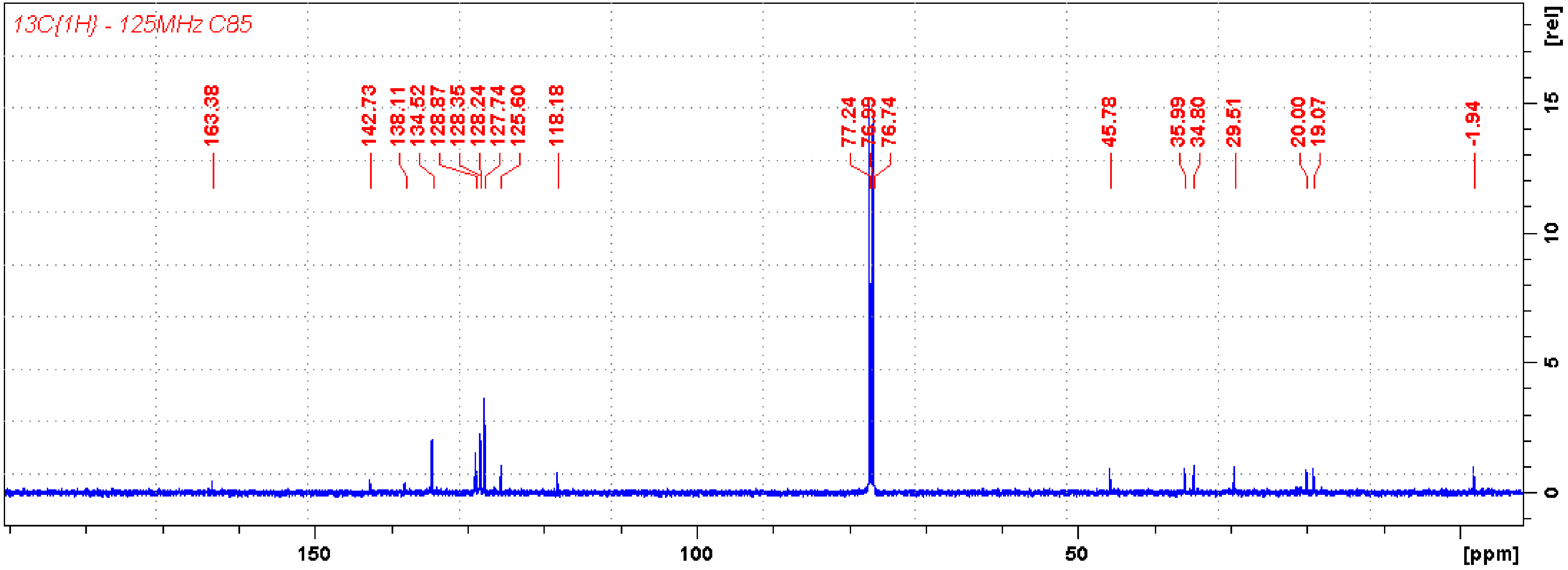

Figure S147. ^{29}Si DEPT NMR (99 MHz, $CDCl_3$, 298 K) of **8cd**.

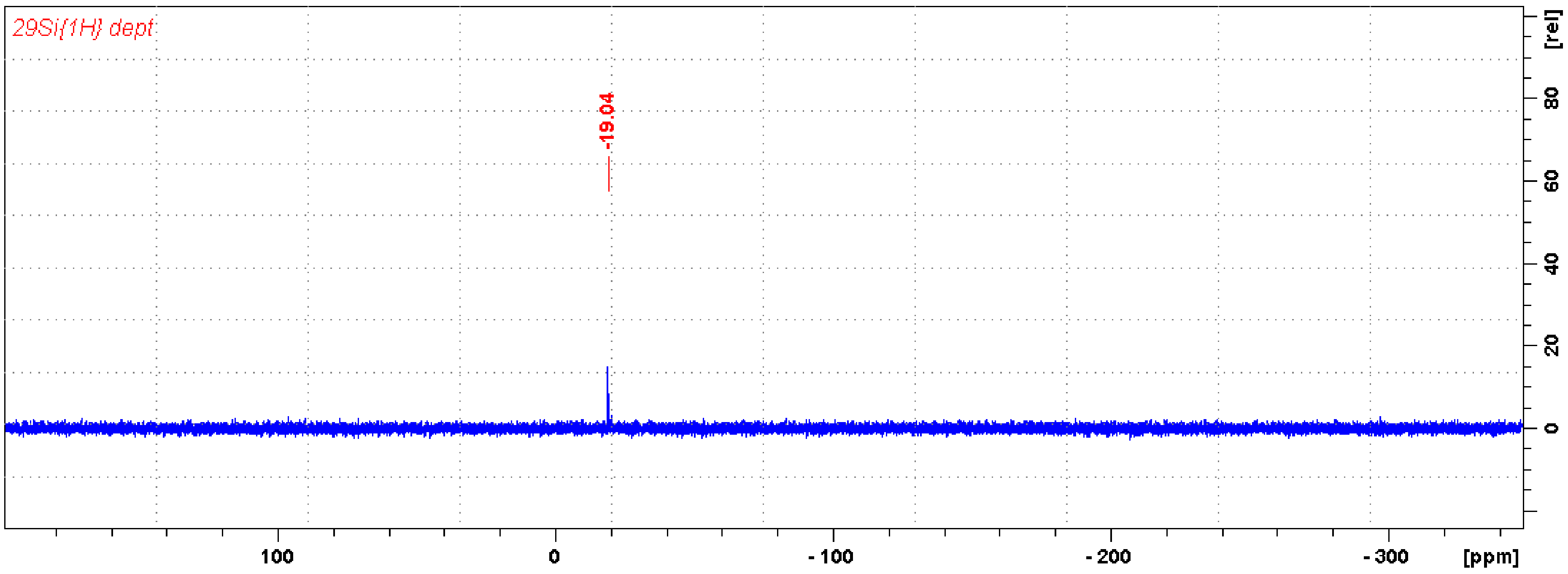

(3*R*,1*E*)-5,6-Dimethyl-7-(methyldiphenylsilyl)hept-6-enenitrile (**8ce**):

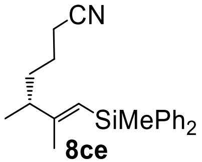

8ce

Figure S148. ^{1}H NMR (500 MHz, $CDCl_3$, 298 K) of **8ce**.

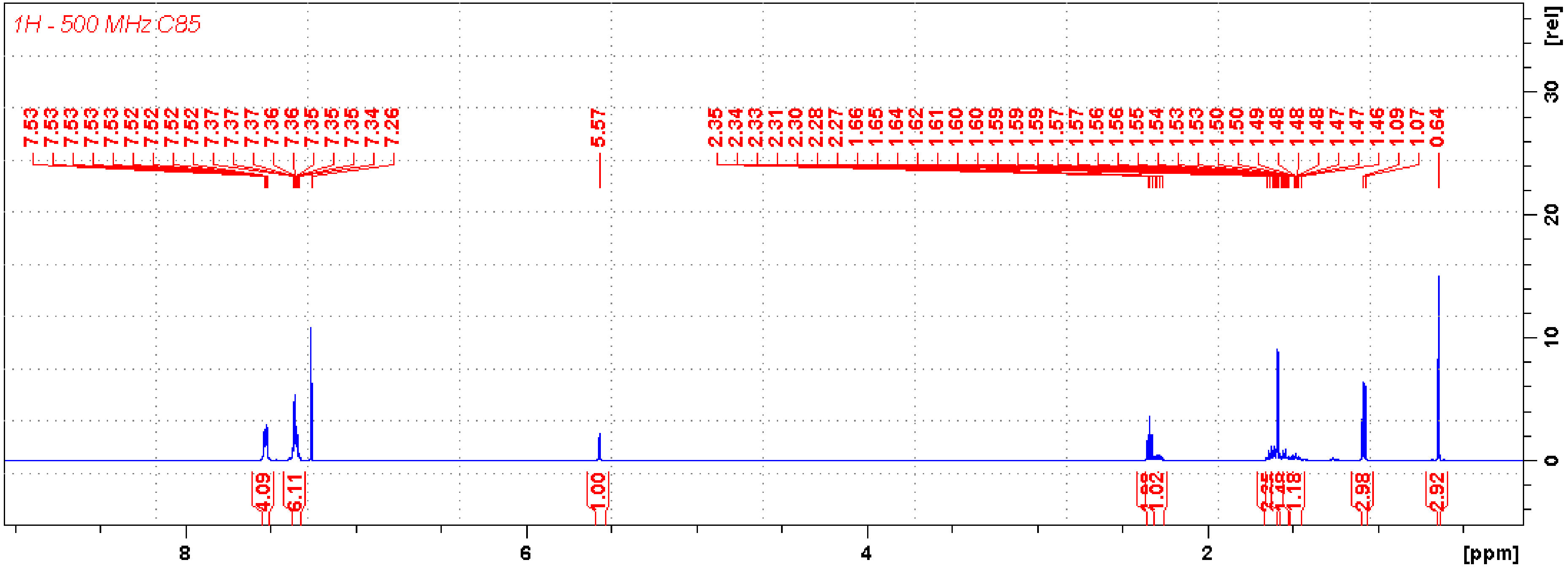

Figure S140. ^{13}C NMR (126 MHz, $CDCl_3$, 298 K) of **8ce**.

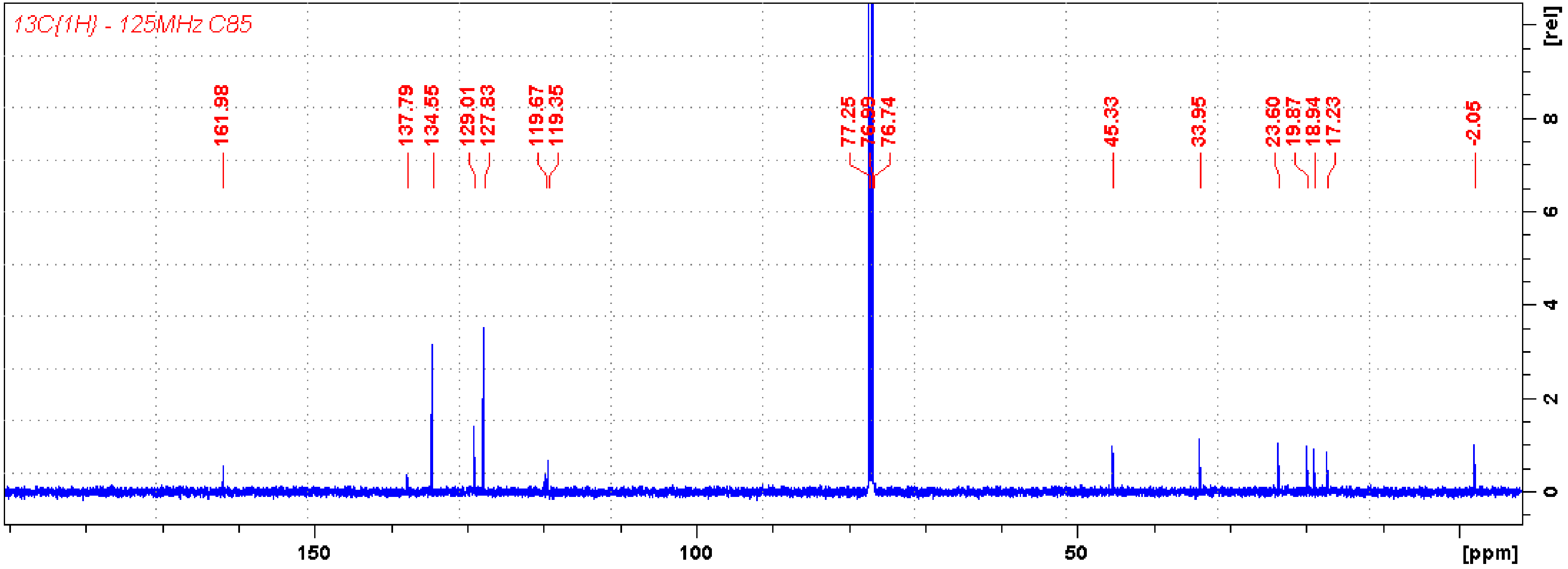

Figure S141. ^{29}Si DEPT NMR (99 MHz, $CDCl_3$, 298 K) of **8ce**.

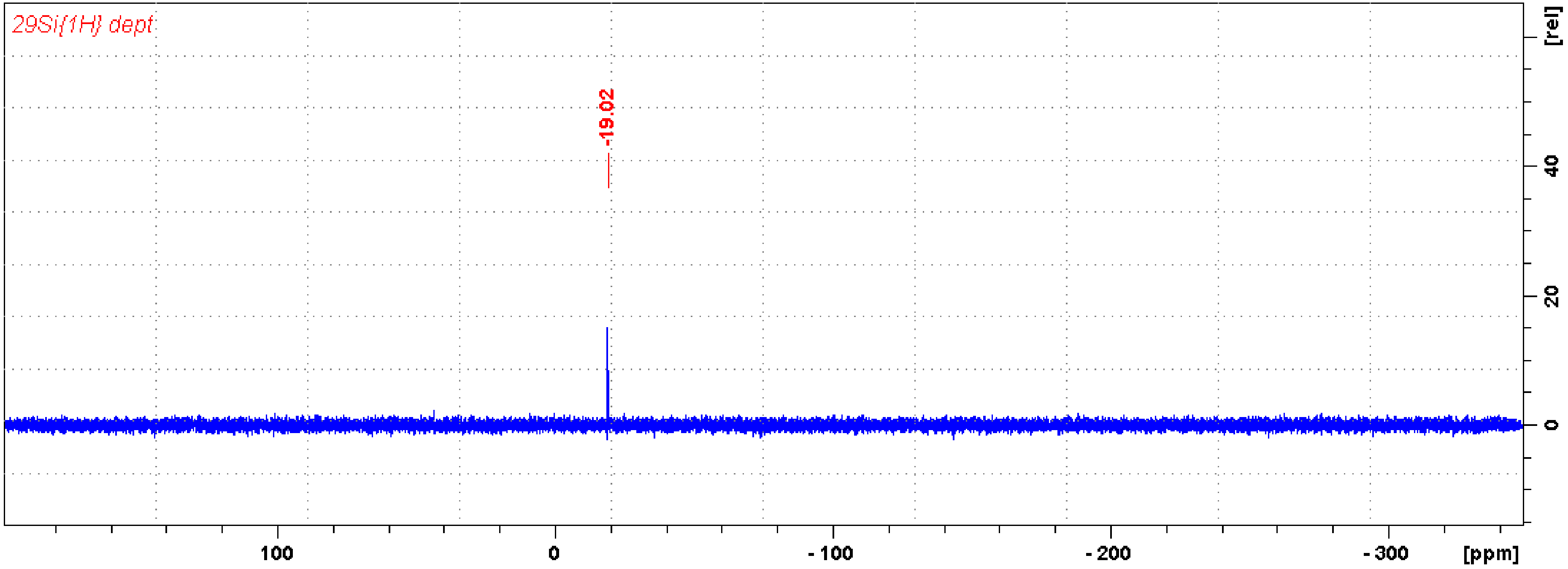

Ethyl (3*R*,1*E*)-5,6-dimethyl-7-(methyldiphenylsilyl)hept-6-enoate (8cf):

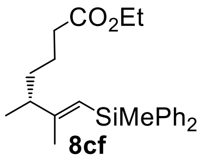

8cf

Figure S149. ^{1}H NMR (500 MHz, $CDCl_3$, 298 K) of **8cf**.

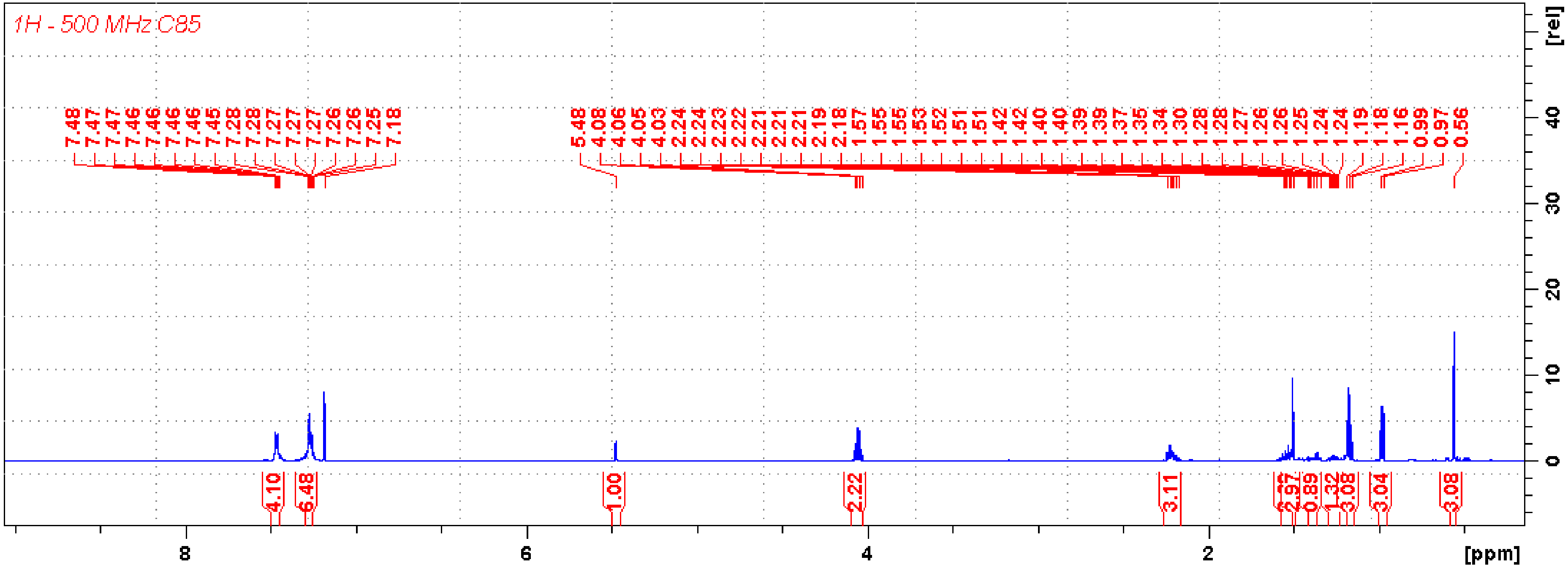

Figure S150. ^{13}C NMR (126 MHz, $CDCl_3$, 298 K) of **8cf**.

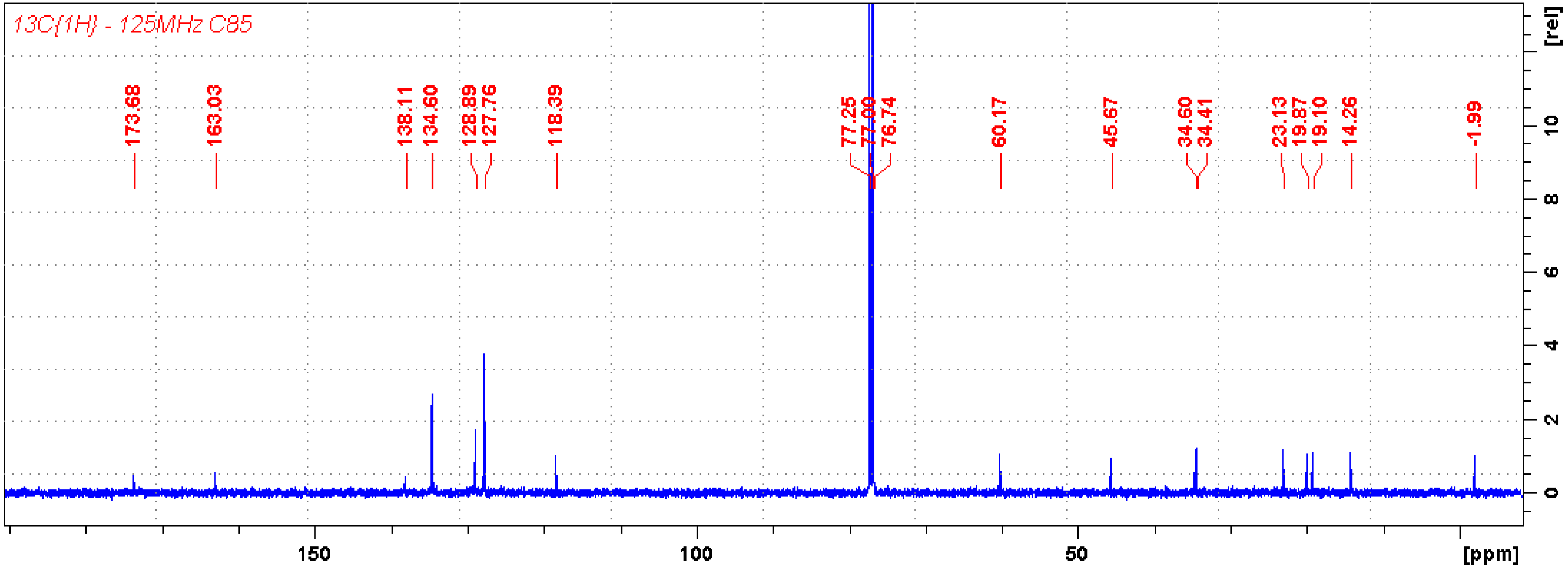

Figure S151. ^{29}Si DEPT NMR (99 MHz, $CDCl_3$, 298 K) of **8cf**.

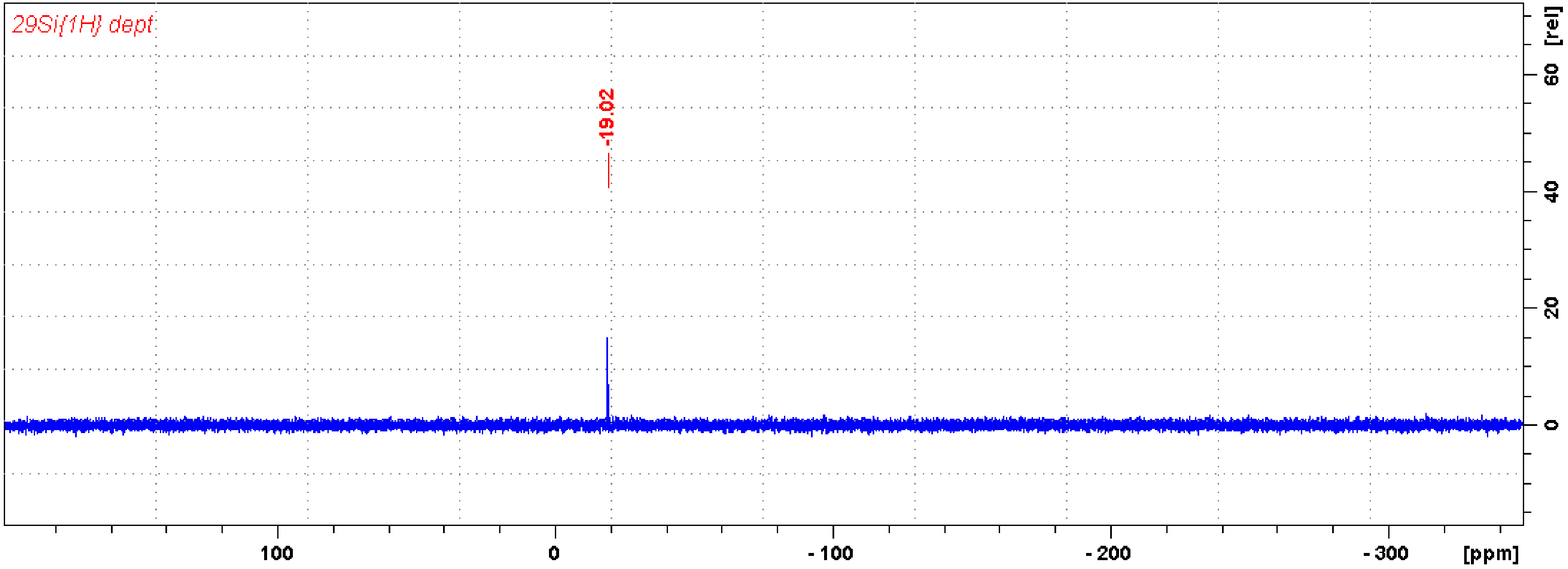

(3*R*,1*E*)-(2,3-Dimethylnon-1-en-1-yl)(methyl)diphenylsilane (**8cg**):

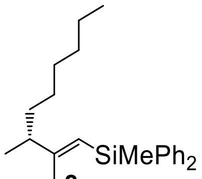

8cg

Figure S152. ^{1}H NMR (500 MHz, $CDCl_3$, 298 K) of **8cg**.

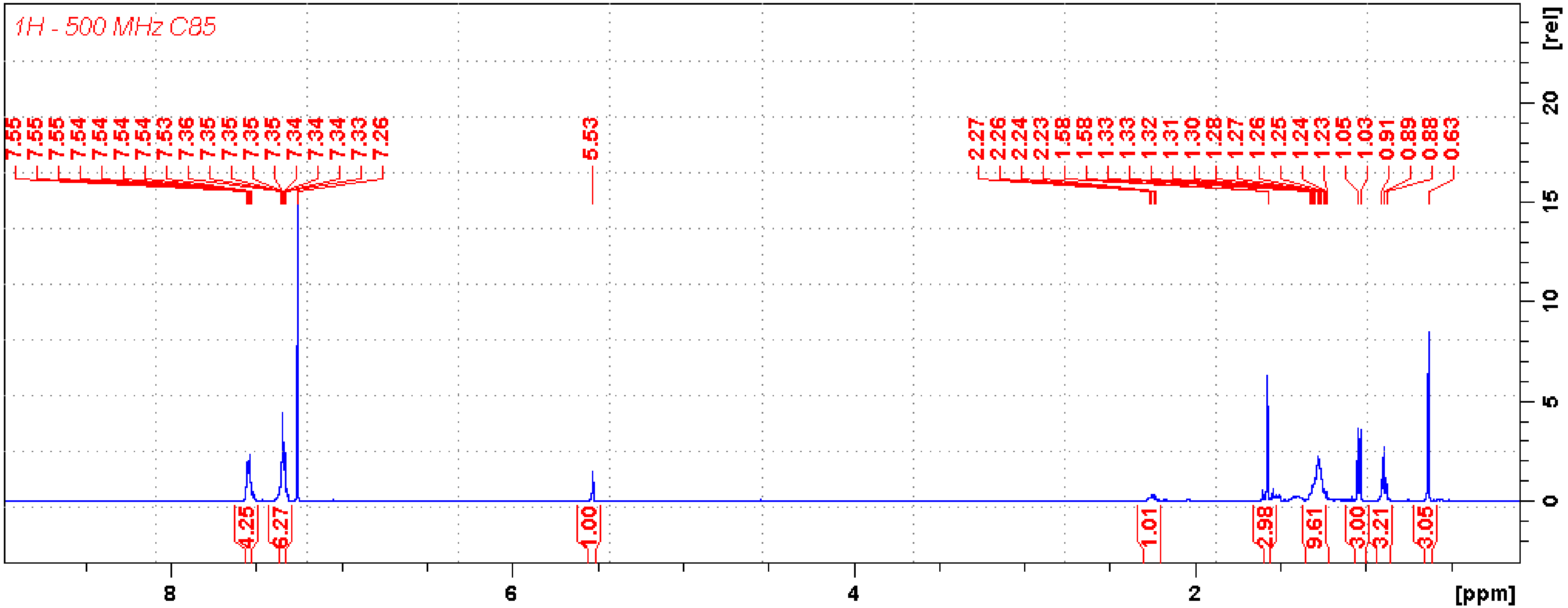

Figure S153. ^{13}C NMR (126 MHz, $CDCl_3$, 298 K) of **8cg**.

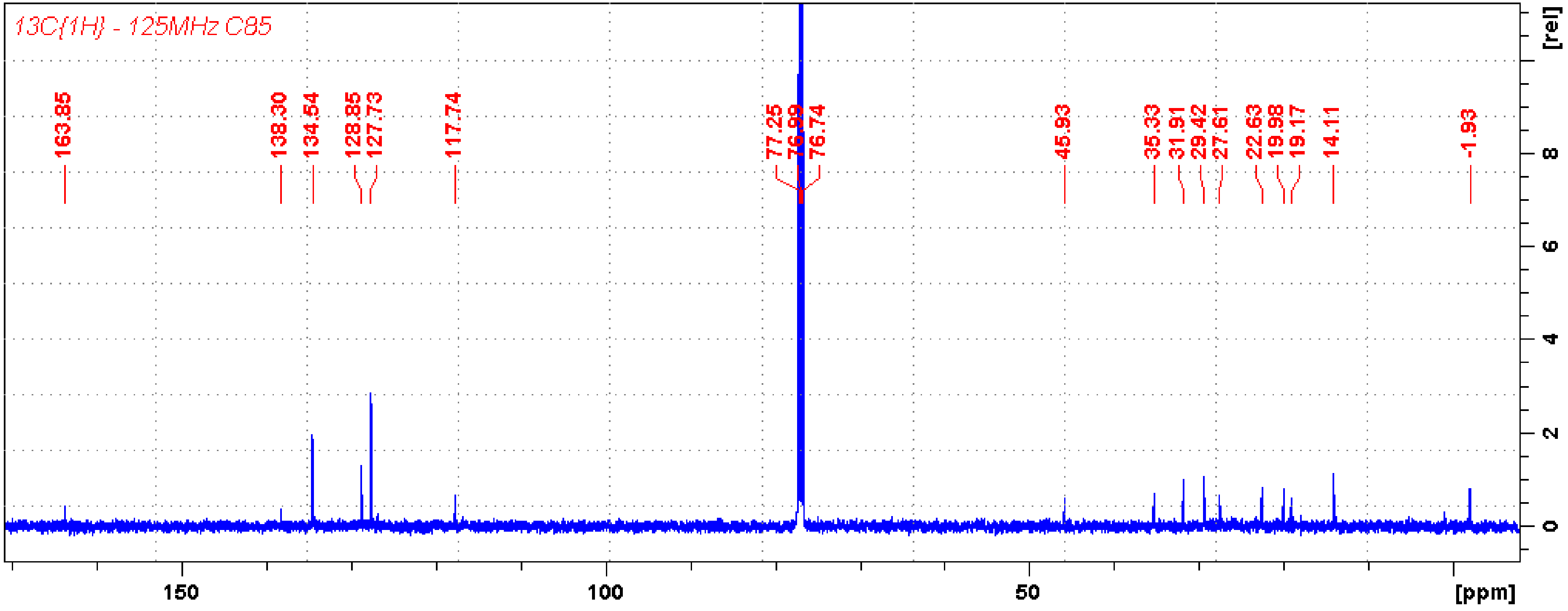

Figure S154. ^{29}Si DEPT NMR (99 MHz, $CDCl_3$, 298 K) of **8cg**.

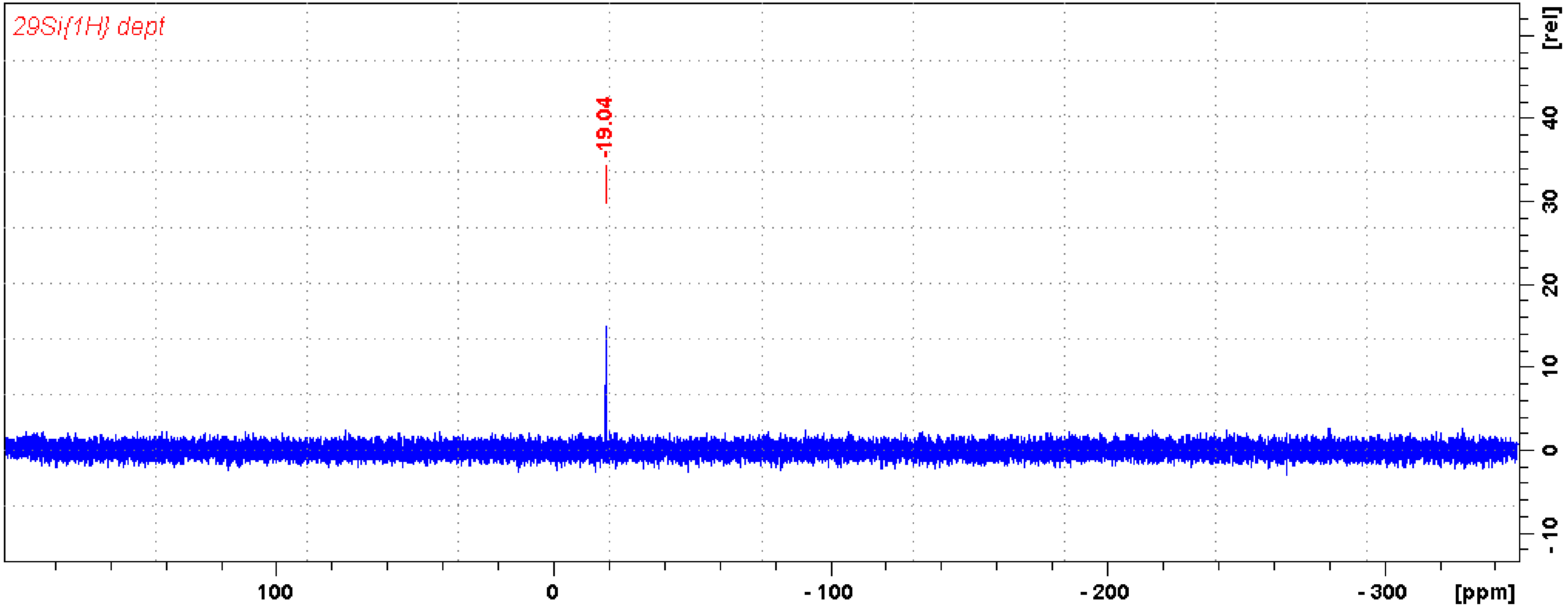

(3*R*,1*E*)-(3-(2-(1,3-Dioxolan-2-yl)ethyl)-2-methylhept-1-en-1-yl)(methyl)diphenylsilane (8da):

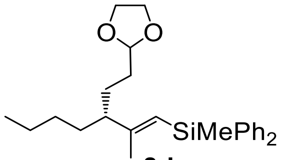

8da

Figure S155. ^{1}H NMR (500 MHz, $CDCl_3$, 298 K) of **8da**.

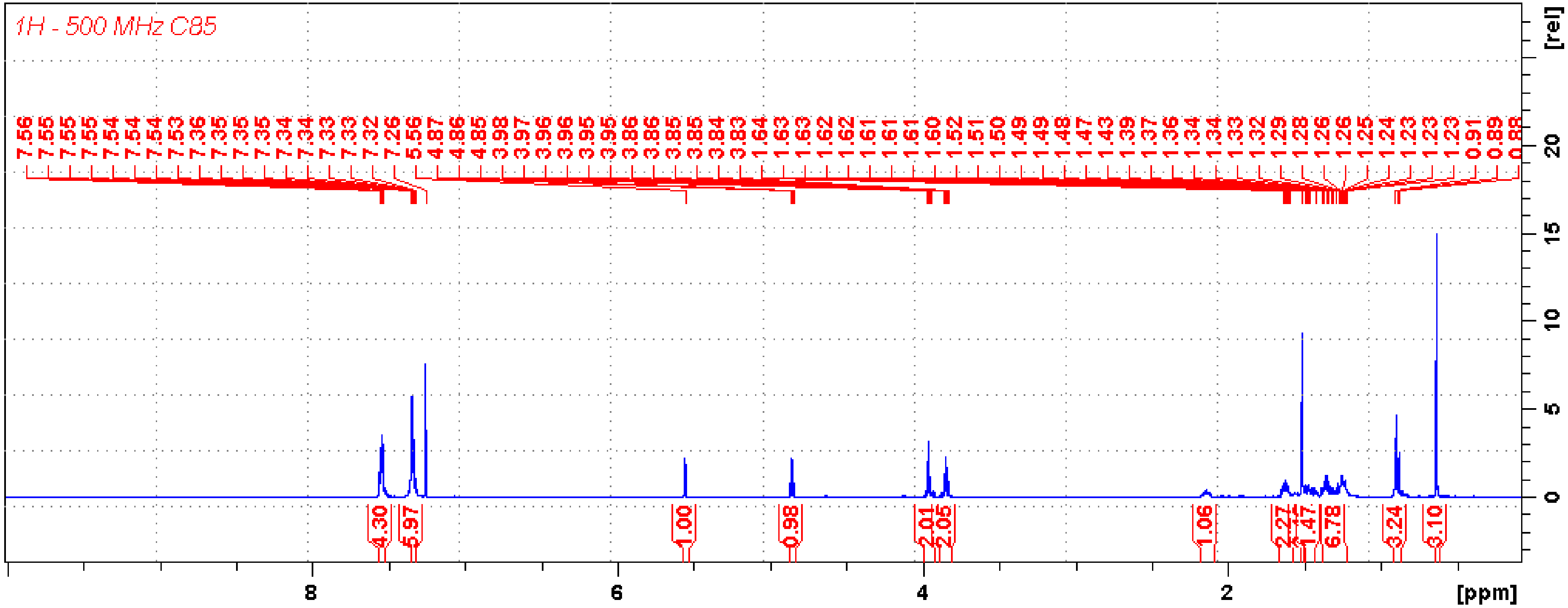

Figure S156. ^{13}C NMR (126 MHz, $CDCl_3$, 298 K) of **8da**.

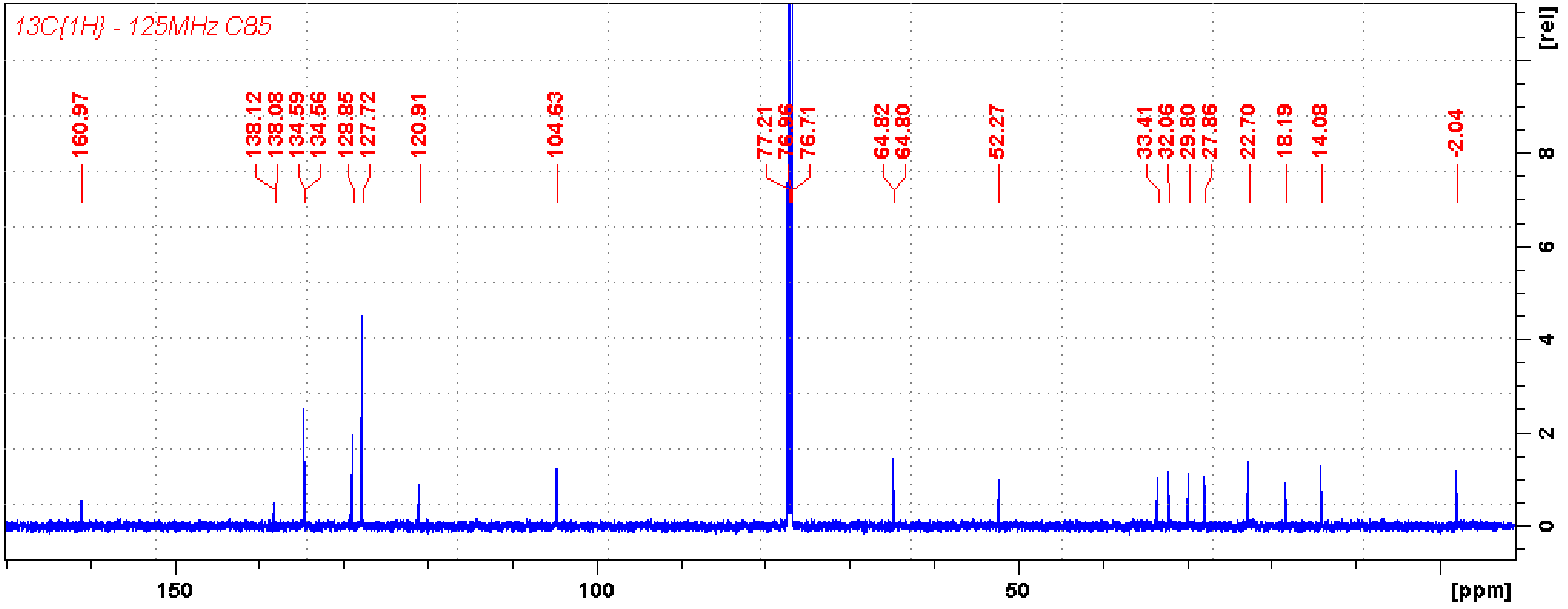

Figure S157. ^{29}Si DEPT NMR (99 MHz, $CDCl_3$, 298 K) of **8da**.

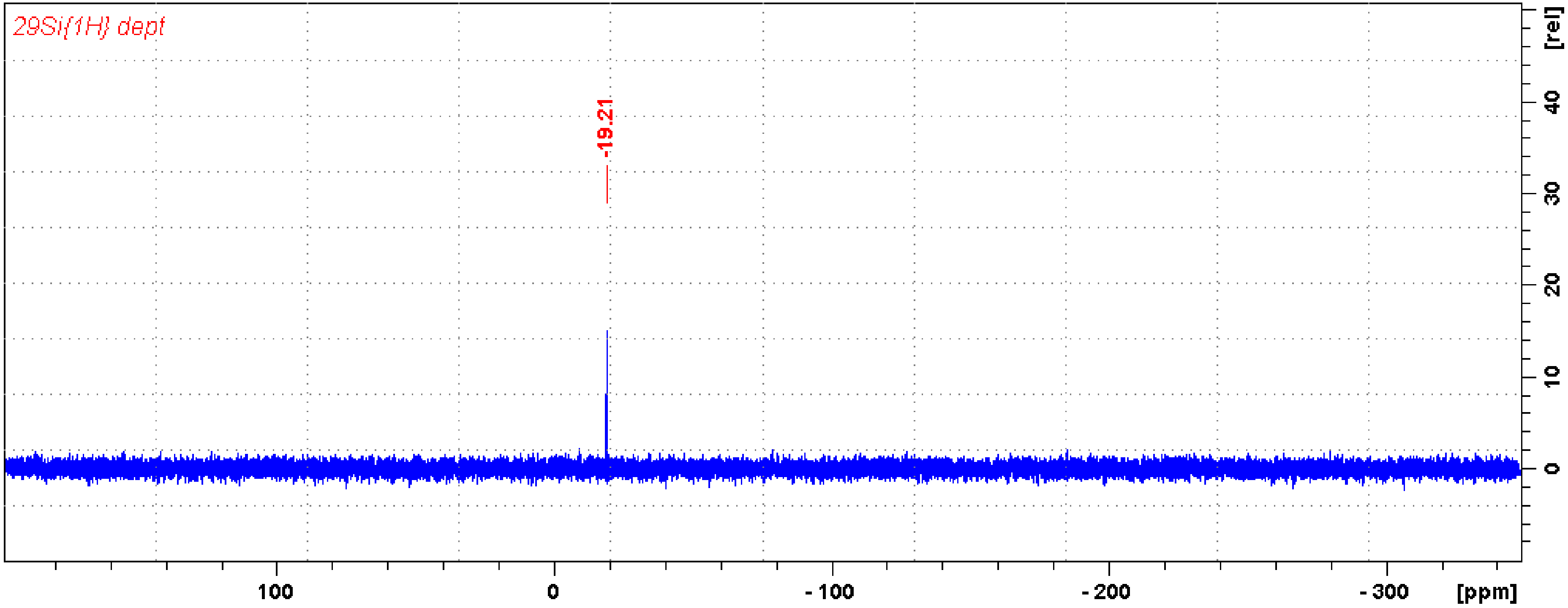

(2*R*,4*E*)-2-(3,7-Dimethylocta-4,7-dien-1-yl)-1,3-dioxolane (**14aaa**):

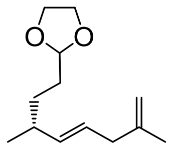

14aaa

Figure S158. ^{1}H NMR (400 MHz, $CDCl_3$, 298 K) of **14aaa**.

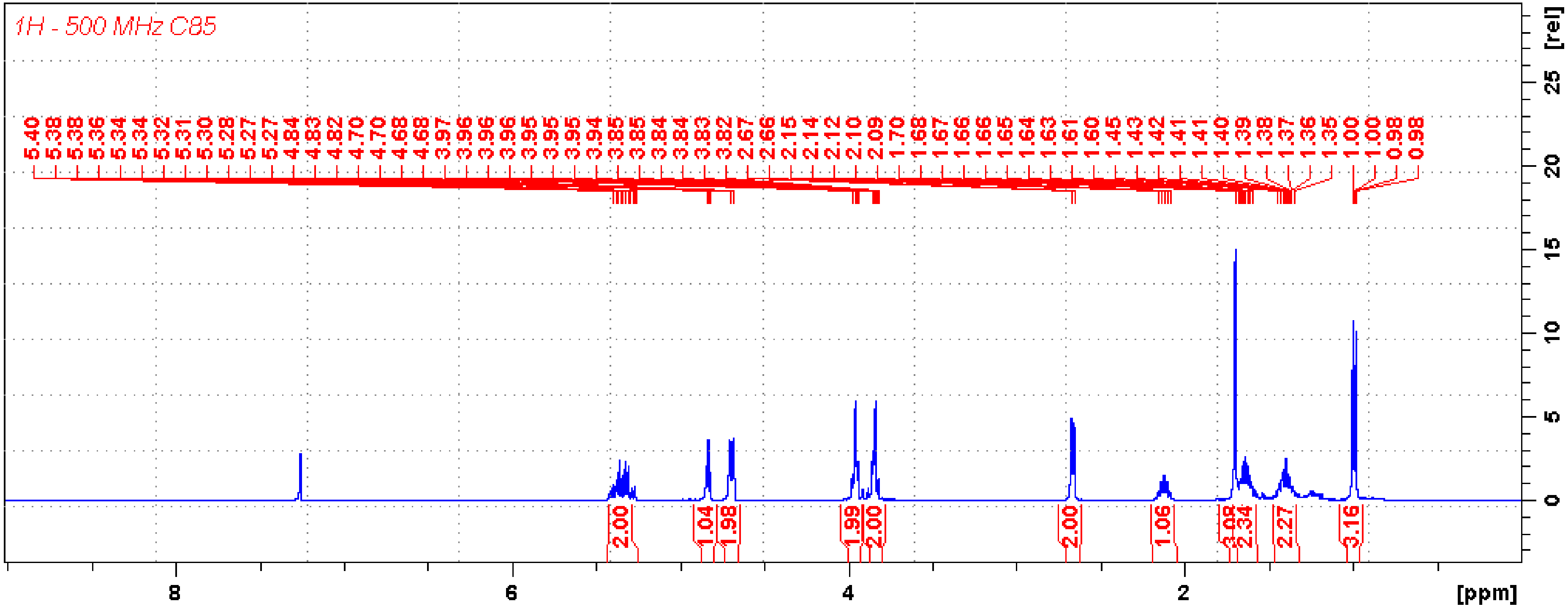

Figure S159. ^{13}C NMR (101 MHz, $CDCl_3$, 298 K) of **14aaa**.

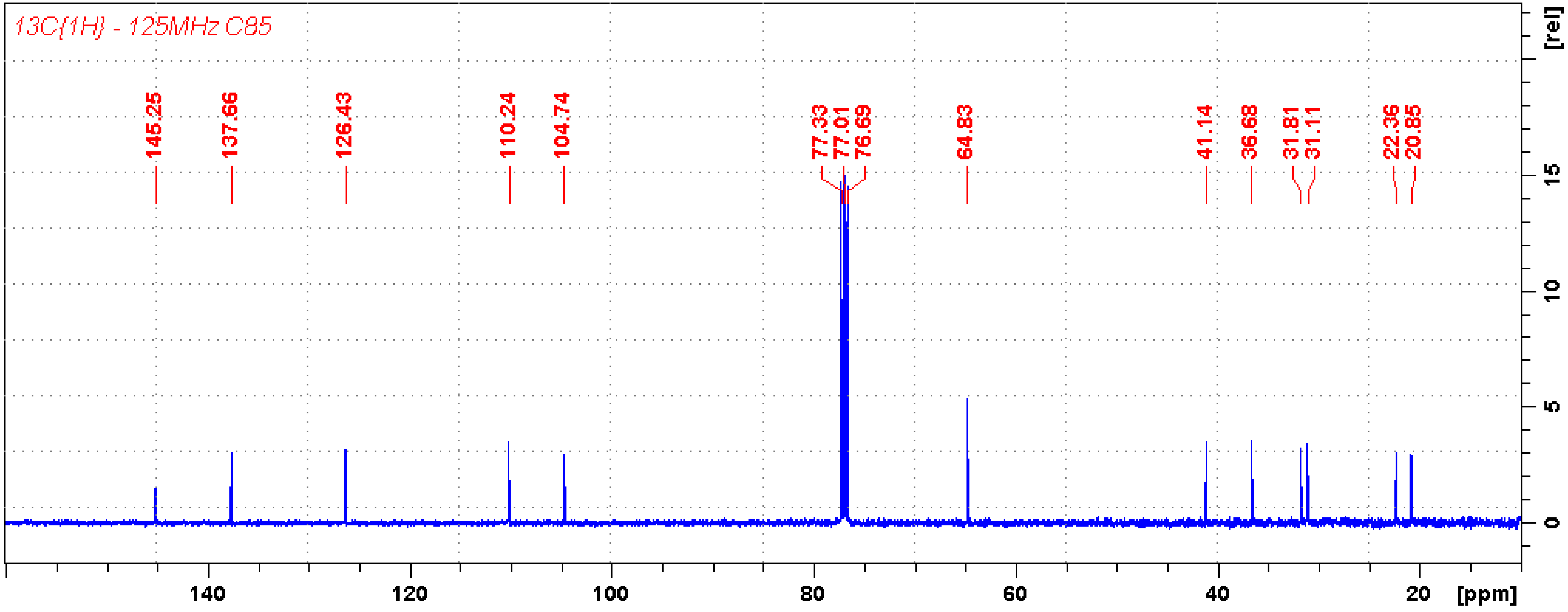

(2*R*,4*E*)-2-(3-Methylhex-4-en-1-yl)-1,3-dioxolane (**14aab**):

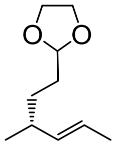

14aab

Figure S160. ^{1}H NMR (400 MHz, $CDCl_3$, 298 K) of **14aab**.

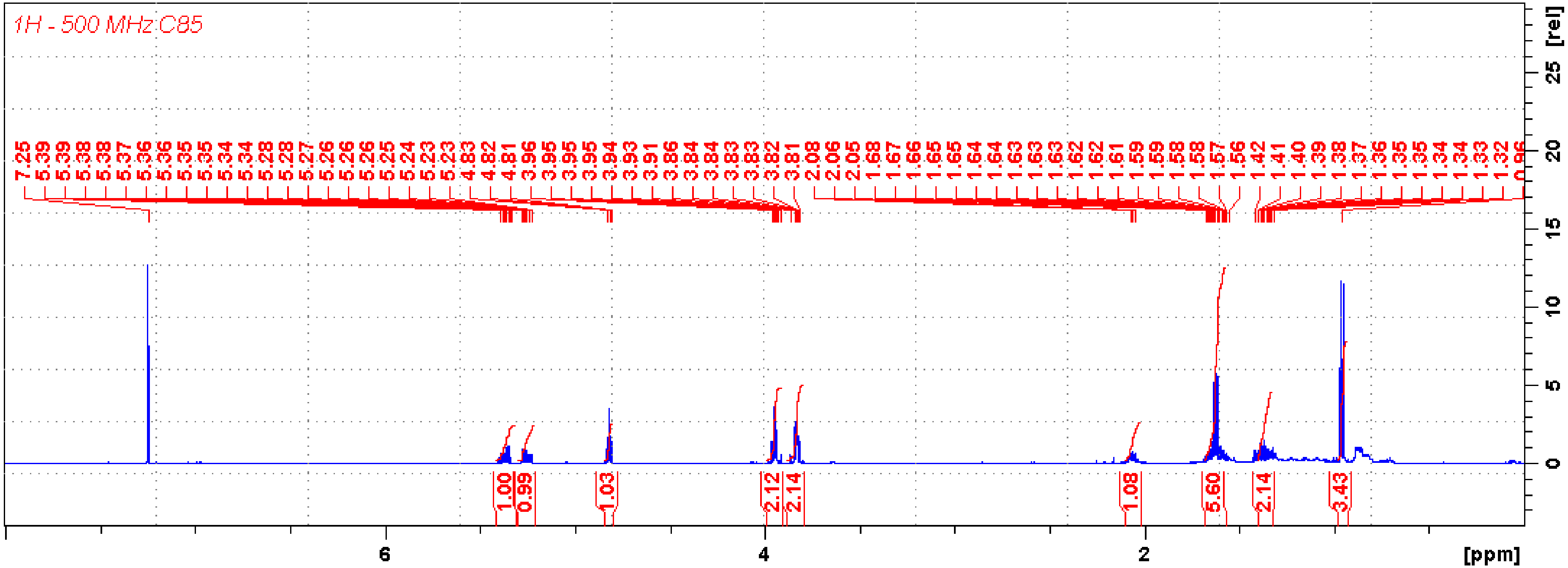

Figure S161. ^{13}C NMR (101 MHz, $CDCl_3$, 298 K) of **14aab**.

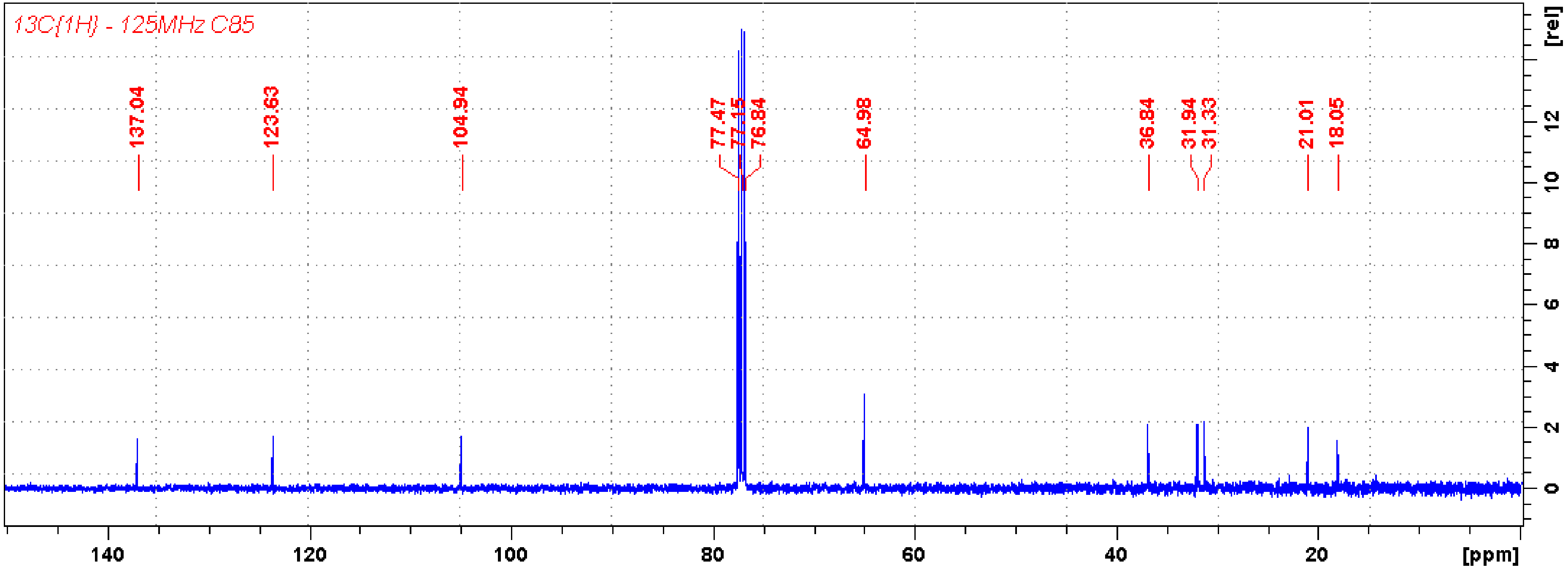

(2*R*,4*E*)-2-(3-(Prop-1-en-1-yl)heptyl)-1,3-dioxolane (**14bab**):

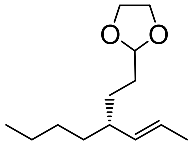

14bab

Figure S162. ^{1}H NMR (400 MHz, $CDCl_3$, 298 K) of **14bab**.

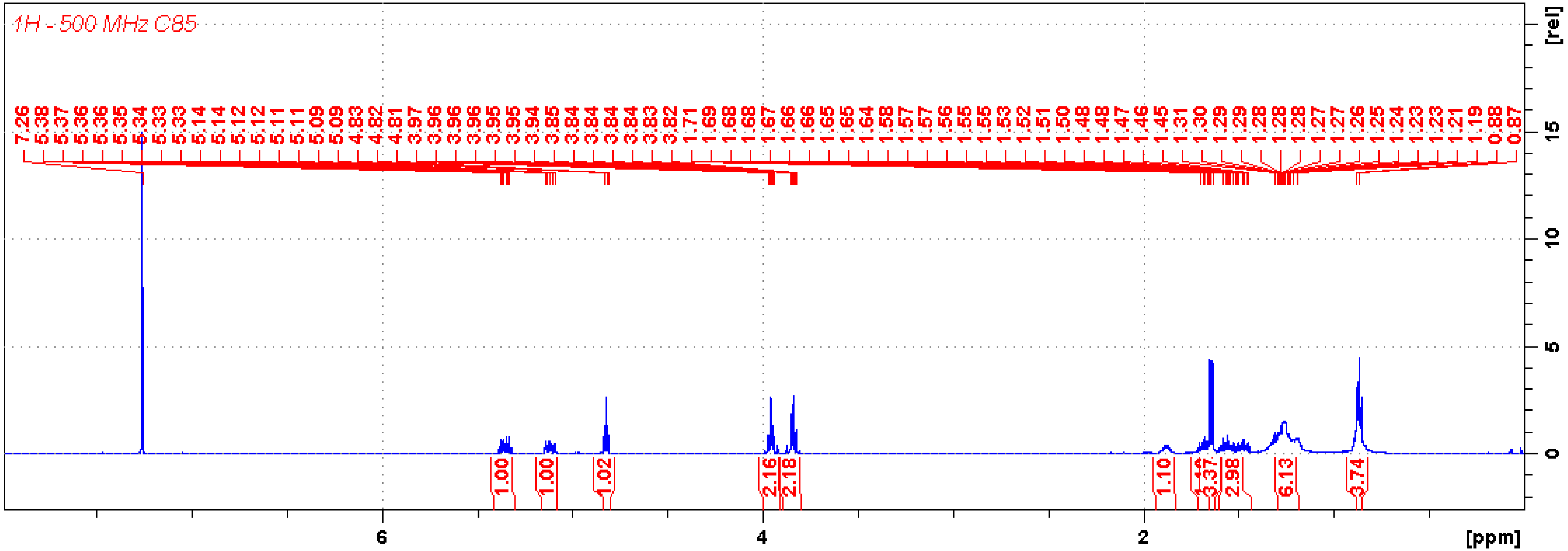

Figure S163. ${}^{13}C$ NMR (101 MHz, $CDCl_3$, 298 K) of **14bab**.

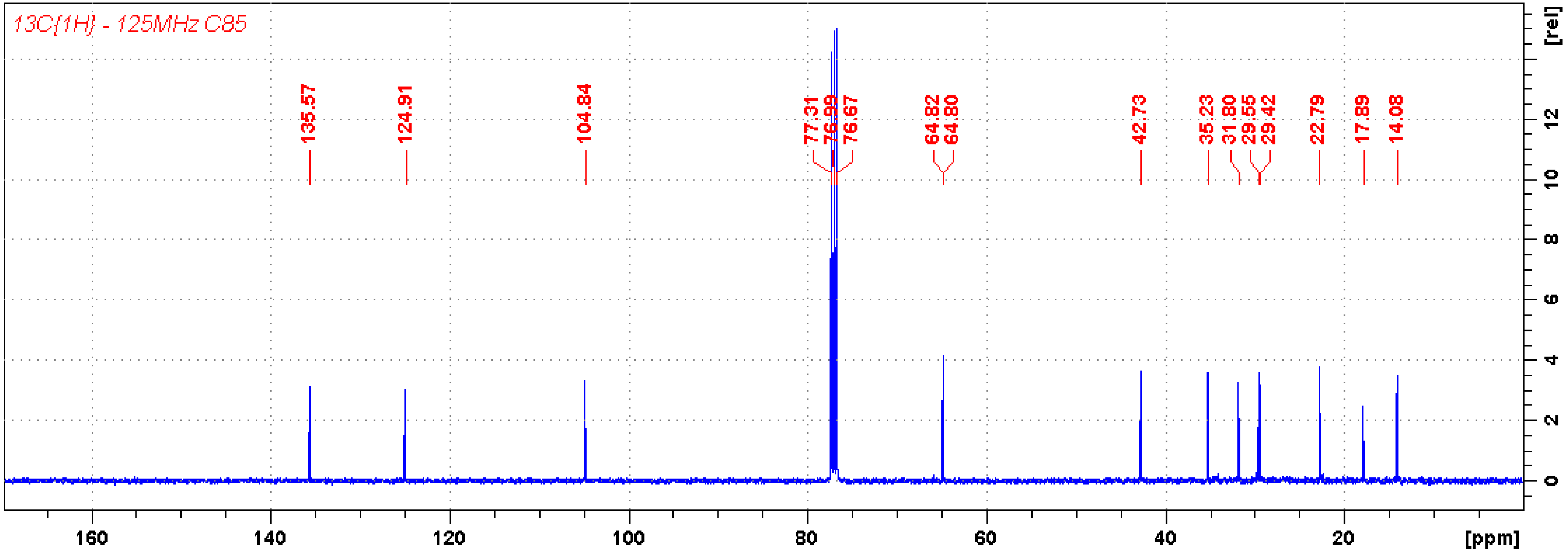

12. References

[S1] R. K. Harris, E. D. Becker, R. Cabral de Menezes, S. M. Goodfellow, P. Granger, *Pure Appl. Chem.* **2001**, *73*, 1795–1818.

[S2] J. Schmidt, J. Choi, A. T. Liu, M. Slusarczyk, G. C. Fu, *Science* **2016**, *354*, 1265–1269.

[S3] A. Romero, K. A. Woerpel, *Org. Lett.* **2006**, *8*, 10, 2127–2130.

[S4] P.-Y. Wang, G. Duret, I. Marek, *Angew. Chem. Int. Ed.* **2019**, *58*,14995–14999; *Angew. Chem.* **2019**, *131*, 15137–15141.

[S5] X. Gao, Y.-L. Xiao, X. Wan, X. Zhang, *Angew. Chem. Int. Ed.* **2018**, *57*, 3187–3191; *Angew. Chem.* **2018**, *130*, 3241–3245.

[S6] I. An, E. N. Onyeozili, R. E. Maleczka Jr., *Tetrahedron: Asymmetry* **2010**, *21*, 527–534.

[S7] M. Honda, W. Oguchi, M. Segi, T. Nakajima, *Tetrahedron* **2002**, *58*, 6815–6823.

[S8] R. K. Shiroodi, M. Sugawara, M. Ratushnyy, D. C. Yarbrough, D. J. Wink, V. Gevorgyan, *Org. Lett.* **2015**, *17*, 4062–4065.

[S9] M. Honda, T. Nakamura, T. Sumigawa, K.-K. Kunimoto, M. Segi, *Heteroatom Chem.* **2014**, *25*, 565–577.

[S10] J.-B. Langlois, A. Alexakis, *Angew. Chem. Int. Ed.* **2011**, *50*, 1877–1881; *Angew. Chem.* **2011**, *123*, 1917–1921.

[S11] J. S. Panek, P. F. Cirillo, *J. Am. Chem. Soc.* **1990**, *112*, 4873–4878.

[S12] W. Jin, J. D. Trzupek, T. J. Rayl, M. A. Broward, G. A. Vielhauer, S. J. Weir, I. Hwang, D. L. Boger, *J. Am. Chem. Soc.* **2007**, *129*, 15391–15397.

[S13] S. Perrone, P. Knochel, *Org. Lett.* **2007**, *9*, 1041–1044.

[S14] T. Takeda, R. Matsumura, H. Wasa, A. Tsubouchi, *Asian J. Org. Chem.* **2014**, *3*, 838–841.

[S15] S. Son, G. C. Fu, *J. Am. Chem. Soc.* **2008**, *130*, 2756–2757.
